# Barriers of last mile supply chain management and reproductive, maternal, new born, and child health product availability in Ethiopia

**DOI:** 10.1371/journal.pone.0346924

**Published:** 2026-05-14

**Authors:** Welelaw Necho Mulatu, Danielson K. Onyango, Happiness Mberesero, Gizachew Tadelle, Dessalew Emaway, Bezawit Mesfin, Tsegaye Shewangizaw, Chala Tesfaye, Andualem Ababu, Biruk Bogale, Kerubo Mogesi

**Affiliations:** 1 JSI, Addis Ababa, Ethiopia; 2 inSupply Health, Nairobi, Kenya; 3 inSupply Health, Dareselam, Tanzania; 4 inSupply Health, Addis Ababa, Ethiopia‌‌; LMU München: Ludwig-Maximilians-Universitat Munchen, NEPAL

## Abstract

**Background:**

Despite notable advancements in Ethiopia’s health supply chain, critical bottlenecks persist at the last mile, particularly within health centres and health posts. This study aimed to identify barriers affecting the availability of reproductive, maternal, new-born, and child health commodities and to explore barriers, and constraints that hinder effective data use for supply chain decision-making.

**Methods:**

This qualitative study employed key informant interviews with 23 participants, including logisticians, pharmacists, store managers, health extension workers, and health program experts. Data were analysed using Braun and Clarke’s thematic analysis framework.

**Results:**

Six key bottlenecks emerged: [1] inconsistent delivery systems; [2] poor data quality and limited use of Logistic Management Information System (LMIS); [3] inadequate inventory management; [4] weak quantification system; [5] staffing shortages; and [6] infrastructure barriers. In pastoral areas, poor road access, limited cold storage, and low eLMIS coverage hinder direct deliveries. Agrarian regions benefit from more direct delivery coverage, but they still face challenges, including aging vehicles and poor rural roads. Across both settings, health posts, the lowest level health facility, often experience inconsistent supply and resort to self-product collection. Indirect delivery via an added layer of woreda (district) offices poses delivery delays. Broader infrastructure issues, such as inadequate storage space, unreliable power, and weak internet connectivity, compound these problems. Data management system weaknesses, including poor reporting quality, limited interoperability, and minimal data use for decision-making, persist in both contexts. Human resource constraints such as shortages of skilled personnel, high turnover, and limited training further undermine supply chain performance.

**Conclusions:**

This assessment highlights interrelated bottlenecks affecting last-mile RMNCH product availability in both pastoral and agrarian areas of Ethiopia, though the severity and context-specific barriers vary. Addressing these issues requires coordinated, system-wide strategies that are tailored to regions with a focus on transportation, strengthening infrastructure, data systems, and workforce capacity.

## Background

To enhance health service delivery and ensure consistent product availability, the Ministry of Health (MOH) in Ethiopia has undertaken significant efforts to strengthen logistics integration, last-mile delivery, and the use of electronic Logistics Management Information Systems (eLMIS) at the facility level. The Ethiopian Pharmaceutical Supply Service (EPSS) plays a central role in managing the national health supply chain through the Integrated Pharmaceutical Logistics System (IPLS), working to ensure a continuous and reliable supply of health commodities to the last mile. Collaborative efforts have introduced tools to enhance data visibility and support informed decision-making. However, challenges such as limited system interoperability, capacity barriers, transportation issues, and inadequate data use continue to affect supply chain performance. A multi-country assessment in Sub-Saharan Africa further highlighted common bottlenecks in the availability of lifesaving health commodities, including insufficient staff training, weak supply chain systems, and stock-outs of priority commodities in approximately 40% of facilities on average [1–3].

Public health facilities in Ethiopia continue to face persistent challenges in ensuring the consistent availability of life-saving reproductive, maternal, new born, and child health (RMNCH) commodities [2]. These supply chain barriers have been linked to increased maternal and child mortality, as they hinder the timely treatment of common illnesses and the management of critical conditions such as eclampsia and postpartum haemorrhage [3]. Key contributing factors include inadequate infrastructure, limited human resources, weak record-keeping practices, and product wastage, all of which undermine the effectiveness of last-mile health service delivery.

A 2018 IPLS survey [4] highlighted significant disparities in commodity availability at different levels of the healthcare system. While 79% of health centres had essential commodities available, health posts lagged at 69.9%, indicating a critical barrier in last-mile service provision. Additionally, overstocking at health centres and hospitals, limited availability of functional cold chain equipment at health posts (only 18%), and the low uptake of digital logistics systems such as Dagu, a platform used to capture, track, and manage health commodity data, currently utilized by only 30% of facilities highlight systemic inefficiencies that must be addressed. The Ethiopian mini demography and health survey (EMDHS) 2019 [5] also showed disparities in RMNCH service utilization between agrarian and pastoralist regions, where commodity availability can be an important barrier. While existing literature, primarily quantitative in nature, confirms the existence and prevalence of stockouts and supply chain barriers, it often fails to uncover the process, contexts, and underlying root causes of implementation failures. There remains a critical barrier in systematic qualitative investigation into the structured barriers impeding effective last-mile commodity management and product availability. A full understanding of the complex, structural nature of these obstacles—such as bureaucratic resistance, poor data utilization culture, and specific human resource challenges—demands an exploratory qualitative approach.

This study intends to address this barrier by identifying key challenges, including those specific to agrarian and pastoral contexts. Exploring contextual differences between agrarian and pastoral regions is crucial to understand fundamentally different logistical and operational challenges for designing tailored interventions to improve commodity availability and healthcare delivery. This study, conducted as part of the Strengthening Service Delivery (SSD) Project in Ethiopia, sought to generate actionable insights into last-mile RMNCH commodity availability. Specifically, the study aimed to identify barriers to the availability of health commodities needed for primary health care within an overall service delivery intervention focused on improving RMNCH outcomes. and to assess the opportunities and challenges affecting healthcare workers and managers in using supply chain data as a lever for improving commodity availability.

## Methods

### Setting

Ethiopia’s health tier system consists of three levels: primary (health posts and centres), secondary (general hospitals), and tertiary (specialized and referral hospitals), all linked through a referral system. To support service delivery, health commodities are procured through the Ethiopian Pharmaceutical Supply Service (EPSS) from both international and local sources. Program commodities are centrally quantified, while Revolving Drug Fund (RDF) commodities are quantified in a decentralized manner by individual health facilities and submitted to EPSS for procurement, based on their needs and available budgets. Program commodities are distributed through the Integrated Pharmaceutical Logistics System (IPLS), with hospitals and health centers submitting requests and receiving resupply every two months from EPSA hubs. Health posts are resupplied monthly by their respective health centers. Reproductive, Maternal, New-born, and Child Health (RMNCH) commodities are accessed through both program and RDF mechanisms.

This qualitative study focused on last-mile healthcare supply chain dynamics within the Strengthening Service Delivery (SSD) project intervention areas in Ethiopia, covering 36 woredas (districts) across eight regions which are purposively selected based on programmatic considerations including collocating with existing projects and included both agrarian (largely agriculture-based subsistence) and pastoralist (nomadic to semi-nomadic) areas. The Woredas for this qualitative study were purposively sampled from the Project Area to ensure adequate representation of both the pastoral setting and the agrarian setting. This selection was strategically designed to explore context-specific supply chain challenges within settings that were the focus of a new programmatic intervention aimed at improving service delivery, as the qualitative findings were required to define the specific logistical and structural bottlenecks.

### Design

This study deployed a qualitative research technique using key informant interviews as a primary data collection method. The interview guides were developed with consideration of the Logistics System Assessment Tool (LSAT) and the Strategic Pathway to Reproductive Health Commodity Security (SPARHCS) frameworks to ensure comprehensive and structured data collection.

### Sampling

Purposive sampling was used to select 23 key informants with a minimum of 5 years of experience and expertise in the Ethiopian healthcare supply chain and primary healthcare systems, with interviews conducted until data saturation was reached. Participants included pharmacists, supply chain experts, Health Extension Workers, and RMNCH experts. For the purpose of this study, an **RMNCH Expert** was operationally defined as a health officer at the regional level who monitors **RMNCH programs** at the subnational level. Informants were drawn from both agrarian (Oromia and Sidama) and pastoralist (Somali) regions to ensure inclusion of diverse service delivery contexts and capture context-specific supply chain barriers. Five interviews (22%) were conducted in pastoralist settings. Data collection continued until thematic saturation was reached to ensure sufficient depth to capture barriers across settings.

### Data collection procedures and quality assurance

Key Informant Interviews (KIIs) were the primary method of data collection. Interview guides were tailored to participants’ roles and covered topics such as logistics operations, commodity management, data use, and supervision practices. Participants were drawn from Regional Health Bureaus (6), implementing partners (3), Zonal Health Departments (ZHDs – 1), Woreda Health Offices (WoHOs – 3), EPSS hubs (3), hospitals (2), health centers (3), and health posts (3).). Key informants were selected using a purposive, stratified rubric designed to ensure balanced representation across the three main functional nodes of the RMNCH supply chain. Participants were stratified into the following areas: oversight, and coordination (RHBs, ZHDs, and implementing partners); logistics and supply management (EPSS Hubs); and Last-Mile Service Delivery (WoHOs, Hospitals, Health Centers, and Health Posts). This strategy ensured that the voices of supplying entities and service providers at last mile were prioritized, with 14 of 23 key informants (60.8%) drawn directly from the supplying hubs, districts and facility levels, confirming a strong focus on the last-mile context. Key informants were included based on their demonstrated experience in last-mile commodity management within RMNCH programs, as well as their involvement in service delivery across various levels of the health system, including health posts, health centers, and hospitals. Data collectors underwent training on qualitative data collection techniques, transcription protocols, and ethical considerations. Role-playing exercises were used to familiarize research assistants with the guides. Besides, data collectors received **technical training on last mile commodity management** and specific instruction on the **interview guides** to ensure subject matter competency. Data were collected from August 6–16, 2024 which were audio-recorded to ensure accuracy. Two teams were deployed to collect the data composed of supply chain experts, public health professionals, data analysists as well as research assistants. Probing questions were used to explore participants’ experiences, challenges, and perceptions comprehensively, and audio recordings were transcribed verbatim for analysis. Research assistants took daily notes and observed participants’ non-verbal gestures in addition to making audio recordings. Research assistants took daily notes and observed participants’ non-verbal gestures in addition to making audio recordings. These notes were used to track the emergence of concepts and guide iterative adjustments to the interview guides. Saturation was monitored continuously during data collection through ongoing review of interview notes and emerging themes, allowing for iterative refinement of data collection. Saturation was then formally confirmed at the conclusion of data collection, when a final review of all transcripts indicated that no new substantive concepts or categories were emerging. To enhance the quality and accuracy of the data, the interview guides were translated into the local language during interviews and subsequently back-translated into English. Supervisors were responsible for ensuring that the data collection process adhered to the data collection guidelines and for leading the daily briefing sessions to maintain data quality

### Analysis

The data analysis followed Braun and Clarke’s thematic analysis framework which is a widely recognized and flexible method for identifying, analysing, and reporting patterns (themes) within qualitative data. The thematic analysis, allowed for gaining an in-depth, contextualized understanding of the experiences and perceptions of key informants, which is essential for developing effective, evidence-based, and tailored interventions. It systematically guides the researcher through six essential phases, from initial data familiarization and coding to the final definition and naming of themes [6], incorporating an additional validation phase to ensure credibility. Using Dedoose software, the process involved familiarization, coding, theme identification, review, refinement, naming, interpretation, and reporting [7,8]. The familiarization stage included repeated reviews of interview transcripts and field notes, organizing them by respondent type and region for contextual comparisons. Initial coding was conducted using both inductive and deductive approaches, with descriptive codes capturing key topics and interpretive codes uncovering deeper meanings and relationships within the data. These codes were systematically documented in a preliminary codebook.

Thematic development was structured around the Logistics Management Cycle (LMC), with codes grouped into broad themes such as quantification, procurement, inventory management, storage, distribution, LMIS, and supervision [9]. Relationships among themes were mapped to highlight interconnections. Candidate themes were reviewed to ensure they accurately reflected the data and aligned with study objectives, emphasizing key barriers and opportunities in last-mile supply chain management. The final six themes identified were commodity management, quantification and procurement, inventory management (storage and distribution), LMIS, organization and staffing, and monitoring and supervision, each of which was clearly defined with its relevance to supply chain management challenges outlined. The interpretation and reporting phase synthesized findings into a coherent narrative supported by illustrative participant quotes. A validation process was undertaken to enhance credibility through data triangulation, peer debriefing, audit trails, and the inclusion of verbatim quotes to ensure participant voices were authentically represented. Specifically, data triangulation was achieved by cross-referencing transcripts with field observation notes. Peer debriefing was conducted with the internal research team and technical experts from the Strengthening Service Delivery (SSD) Project teams to challenge and verify the emergent themes. These strategies strengthened the trustworthiness of the findings, ensuring that the study’s conclusions were firmly grounded in diverse participant perspectives.

### Ethical approval, permits, and authorization letters

Ethical approval for the study was obtained from the Institutional Review Board of the Ethiopian Public Health Association (Ref. #: EPHA/OG/382/24, June 28, 2024) as part of monitoring the effectiveness of service delivery project. Regional health bureaus issued letters of support to facilitate data collection. Participants provided written informed consent after being briefed on the study’s purpose, benefit, their role, and the confidentiality of their responses including their right to opt out and to respond to questions without consequences. Recruitment period for participants was from August 6–16, 2024. Ethical considerations were upheld throughout the study to ensure participant privacy and voluntary participation. The authors confirm that all methods were carried out in accordance with the relevant guidelines and regulations.

## Results

### Background characteristics of respondents

The study included 23 key informants from various levels of the healthcare supply chain system. The majority of participants (78%) were male, with roles spanning supply chain experts/pharmacists, RMNCH expert, pharmaceutical and medical device directors and service providers. Participants were drawn from Agrarian regions – Oromia & Sidama (65%), Pastoralist region – Somali (22%), and implementing partners from Addis Ababa (13%). On average, they had 14 years of professional experience as indicated in Table 1, contributing diverse insights into regional and contextual supply chain challenges.

**Table 1:** Demographic characteristics of key informants.

Demographic Variable	Participants N = 23
**Gender**	
Male	18 (78.3%)
Female	5 (21.7%)
**Geography**	
Agrarian (Oromia & Sidama)	15 (65%)
Pastoralist (Somali)	5 (22%)
Addis Ababa (Implementing partners)	3 (13%)
**Years of Experience**	
5–7 years	2 (8.7%)
8–10 years	4 (17.4%)
11–13 years	1(4.3%)
14–16 years	6 (26%)
17–20 years	1 (4.3%)
Missing	9 (39%)
**Role**	
RMNCH Director/expert	3 (13%)
RHB PMD Director/Expert	3 (13%)
EPSS hub distribution coordinators	3 (13%)
Zonal/Woreda Supply chain officers	3 (13%)
Primary Health Care Unit Directors	2(9%)
Facility Pharmacy head/Store manager	3 (13%)
Health Extension workers	3 (13%)
Supply chain Advisors (implementing partners)	3 (13%)

### Barriers of commodities management and product availability at the last mile

Six key themes emerged: (1) inconsistent and inefficient commodity management systems; (2) poor data quality and limited use of the Logistics Management Information System (LMIS); (3) inadequate inventory management; (4) weak quantification practices; (5) staffing shortages and coordination barriers; and (6) infrastructure limitations.

**Fig 1:**
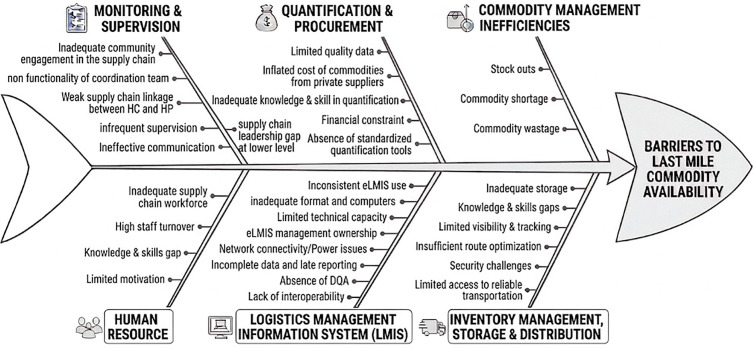
Barriers of last-mile commodity management and product availability. This fishbone diagram identifies barriers of last-mile product availability by categorizing the them into key areas: inventory management and distribution, quantification and procurement, Logistics Management Information Systems (LMIS), supervision and monitoring, and human resources and commodity management inefficiency.

The key barriers contributing to unavailability of commodities at the last mile are portrayed in the Fig 1 below:

#### Commodity management inefficiencies.

Frequent stockouts and wastage emerged as key indicators of commodity management inefficiencies. In both pastoral and agrarian regions, delayed deliveries, low refill rates, and lack of emergency ordering systems contributed to recurrent stockouts. Health posts, which depend on health centres for resupply, often receive inadequate supplies, particularly when health centres prioritize their own needs. In agrarian areas, overstocking and wastage were commonly linked to inaccurate demand forecasts during seasonal shifts.

*“The commodities delivered from EPSS (resupplying agency) might not be enough. For instance, they (Health facilities) may request 100 Depo, but they don’t get it. One health center will provide supplies to 5 health posts. But if the health centre is not getting enough numbers from here, it will be difficult to provide the health posts.” (KII_OA_15)*

On the other hand, overstocking, due to poor quantification practices and lack of accountability, leads to wastage. This issue was observed in both pastoral and agrarian regions.

#### Quantification and procurement barriers.

Common identified bottlenecks regarding quantification included limited availability of quality data, inadequate quantification knowledge and skills and absence of automated quantification tools. Identified root cause to unavailability of quality data was incomplete recording, outdated demographic data, and fragmented reporting systems.

*“We also face challenges to get data that we need for the quantification.” (KII_OA_14)*

Despite having access to data, the absence of a comprehensive tool for effective analysis and utilization poses a significant challenge. *“...we need a tool that can show the risk of stocking out of certain products. …. We are not using the tool to see the status by entering the data. We have too much data, but we do not have a tool that can show us even supply chain risk.”*

The findings also indicate a skills gap among staff in conducting accurate quantification, with many relying on assumptions rather than data-driven approaches as quoted by a supply chain expert:

*“I also believe there is a gap in expertise skills to do the quantification. There is a skill gap in doing quantification and forecasting based assumptions.” (KII_P_21)*

In both settings procurement major bottlenecks were **inadequate budget allocations**, **delay in reimbursement**, and **inflexibility of the budget**- and **inflated cost of commodities** from private suppliers. Inadequate budget poses a significant challenge procuring commodities according to the quantification needs. It often necessitates a budget-driven approach, where available funds dictate the procurement quantities rather than actual health needs. As highlighted by a logistician:

*“Budget shortage prevents you from following the standard. If you request to avail it based on case management, you will be rejected due to budget shortages.” (KII_OA_23)*

*However, procurement products for RMNCH using the RDF scheme is also stated as enabler to creat access to critical services.*

*‘’Already they are using the RDF and purchased Ceftriaxone, Gentamicin, Gloves, and others.” (KII_SP_19)*

#### Inventory management, storage and distribution.

Bottlenecks for inventory management were insufficient tracking mechanisms, poor storage practices, inadequate inventory management knowledge, and a lack of focus on monitoring, which led to commodity wastage and artificial shortages. Insufficient inventory tracking and visibility was reported in both pastoral and agrarian regions, significantly impacting the supply chain management in the health facilities and health posts. One of the primary issues identified in both regions was **failure to consistently update Bin cards** or utilize **automated systems** that provide real-time inventory status as noted by the key informant:

*“If you are not updating your BIN card or using an automated system…, you will face problems monitoring your status.” (KII_P_20)*

Key informants reported that poor coordination between storage units and service units contributed to poor inventory management as illustrated by a participant:

*“We got resources from the store that are out of stock in the service delivery rooms, but they are in the pharmacy. She said ‘I do not have it here, but we can find the supply in the store.”*

Moreover, some health facilities place orders reactively, in response to stockouts, rather than proactively based on anticipated needs as one participant explained:

*“Preventive action is not that much. It is mostly ordering due to stock out. This is the reality that happened. Data management for prevention and service continuity is a weak trend.” (KII_A_11)*

Barriers in proper storage included inadequate storage space, improper storage conditions, lack of power supply and lack of equipment like refrigerators. Moreover, last-mile commodity management is hampered by inadequate transportation infrastructure, limited access to reliable transport, geographical and distance barriers, lack of standardized distribution from woredas to health facilities and health posts, inconsistent delivery schedules, and insecurity. The transportation of commodities to last mile particularly to remote areas relies heavily on ambulances or vehicles provided by the woreda administration, which sometimes are old and not available consistently. One respondent explained:

*“Since the vehicle is provided by woreda, you may or may not get it. Such problems happen regarding transportation.” (KII_OA_05)*

The cost of transportation further compounds the problem, with some HEW paying out of pocket for private transportation to collect supplies, as explained by a participant:

*“If we face such a challenge, we go to the X health center using a private car by paying costs from my pocket. (KII_SP_17)*

#### Logistics management information system.

**Fig 2:**
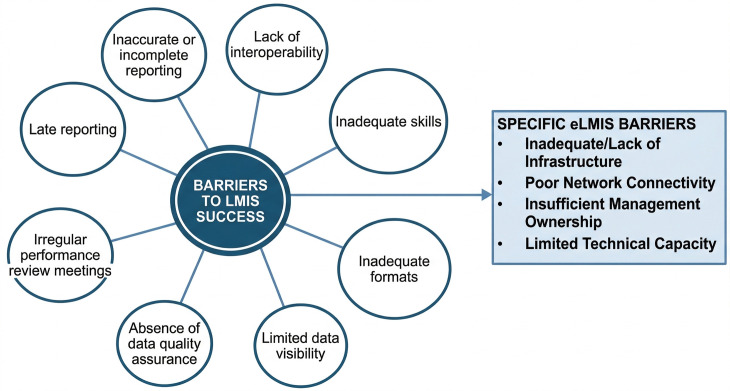
Barriers to the Logistics Management Information System (LMIS). This diagram summarizes the key challenges and root causes impacting the efficiency of the LMIS. The central circle outlines the main barriers, while the connected circles detail specific issues such as inaccurate or incomplete reporting, inadequate skills, limited data visibility, and a lack of interoperability. The accompanying list further expands on the infrastructure and technical limitations of the electronic LMIS (eLMIS).

As shown in **Fig 2**, our analysis revealed a variety of barriers impacting the LMIS, including those specific to both the overall system and its electronic components

Absence of complete recording and poor data quality of reports were the most significant challenges. As noted by a supply chain expert:

*“The RRF that is sent to EPSS is of poor quality most of the time. It shows a gap in data quality. It might be due to negligence and lack of commitment.” (KII_P_20)*

The reliance on paper-based systems in the pastoralist areas exacerbates these issues, making the process prone to errors and delays. The absence of an electronic system limits data visibility and creates additional barriers in reporting. As one participant mentioned:

*“Most of our reports are in hard copies, and it is better if they are replaced with an electronic system.” (KII_SP_01).*

Inadequate access to essential reporting formats was a common issue in both the pastoral and agrarian regions, significantly hindering the efficiency of the health supply chain data.

In the pastoral region, the lack of access to reporting formats such as Model 19 (Receiving Voucher), Model 22 (Issuing Voucher), the Reporting and Requisition Form (RRF), the Internal Facility Reporting and Resupply Form (IFRR), the Health Post Monthly Report and Resupply (HPMRR), and bin cards presented a major obstacle to effective data management. Health centers and health posts struggle to obtain these essential forms, which are crucial for accurate reporting and resupply. As one participant explained:

*“There are considerable challenges in accessing Model 19 and Model 22.” (KII_SP_19).*

Moreover, the report findings indicated a significant barrier in the formal assessment of data quality at the health centre and health post. There was a notable absence of dedicated teams or committees specifically tasked with monitoring and improving the quality of supply chain data in both agrarian and pastoral regions. As noted by the logistician:

*“We did not formally formulate specific teams for quality issues, and for report purposes; We do not have a team or committee formation for such an issue.” (KII_SP_19)*

The findings highlight a lack of interoperability and harmonization of reporting timeliness for logistics and service reports. A supply chain expert noted:

*“For instance, Dagu is used for warehouse monitoring and inventory, Mbrana is used for vaccines, and selected KPIs are available through District health information system(DHIS2), but these systems operate independently without a cohesive framework.” (KII_P_20)*

This qualitative finding highlighted that the lack of interoperability led to discrepancies, as seen in the case of malaria, where consumption data filled in the RRF did not match service delivery data. The logistics management information system is detached from service delivery data, making it difficult to accurately assess stock levels and service requirements. As explained by a participant:

*“We need amendments [on IPLS] as additional requirements are coming frequently. Some fabricated stockouts are coming, I think we have to do more on triangulating the service data with the consumption.” (KII_P_21)*

Limited network connectivity, technical capacity challenges, insufficient management ownership and inconsistence use of LMIS were major obstacles identified for eLMIS implementation. A participant tried to explain impact of using both manual and eLMIS:

*“We are not progressing to the level we want due to this internet connectivity problem.” (KII_OA_15)*

A supply chain expert added that generally without strong leadership acceptance and clear accountability structures then the system might not operate optimally:

*“Since this system is a transactional system and needs consistent monitoring, it cannot be stable as long as the leadership and accountability properly exists.” (KII_P_12)*

#### Organization and staffing barriers.

This qualitative research insights revealed that stock availability at the last mile is significantly impacted by: shortages in the health supply chain workforce, lack of motivation and commitment among staff, negative staff attitudes, high staff turnover, insufficient induction and skills transfer for new staff, training and skills gap, and workloads. Below are notes from key informants:

*“Pharmacists are rarely found in rural areas as they are often not inclined to live there. There are no applicants for pharmacist vacancies in these areas.” (KII_A_22)*

Furthermore, the findings revealed limited staff commitment to supply chain tasks, particularly among non-pharmacy professionals who perceive these responsibilities as outside their area of expertise. Respondents expressed their concerns as follows:

*“I was not interested in quitting my profession, as I am clinical. I want to work on that rather than being a pharmacist.” (KII_A_09)*

Combined with a lack of formal training, many staff members feel ill-equipped to handle the responsibilities related to supply management, which contributes to delays and inefficiencies in the system. New staff and those in the system complained of not receiving sufficient induction nor skill transfer.

#### Supply chain coordination and communication barriers.

Key areas of concern that emerged were: ineffective communication, the absence of a zonal structure in Somali, deficiencies in leadership and governance, weak linkages between health centres and health posts, inadequate feedback from partners, conflicts of interest among partners, centralized decision-making in commodity allocation, the lack of dedicated coordination teams, and infrequent supervision.

Key informants reported that the absence of functional internal coordination mechanisms, such as Technical Working Groups (TWGs), limited the ability to regularly discuss challenges, identify solutions, and escalate issues requiring external support. They also noted that weak enforcement from facility management contributed to poor compliance with supply chain procedures. As explained:

*“The health centre management should enforce if the dispensing unit is not using IFRR. Weak management and lack of enforcement at facility level is a problem.” (KII_P_20)*

Although there are linkages between health posts and health centres for service delivery, there is still a need for stronger coordination and support related to product management, as noted by a participant:

*“There are linkages between health posts and health centres in terms of services, but more activities are required for product-related support.” (KII_A_11)*

#### Monitoring and supervision.

This theme highlighted barriers to effective monitoring and supervision, including infrequent supervisory visits, weak communication channels, and the absence of regular mentorship programs.. A respondent explained the situation as follows:

*“There is no mentorship program that specifically focuses on the supply chain.”*

Key informants identified inadequate budget as a key factor contributing to infrequent supervision, noting that limited resources hinder routine visits from health centers to health posts, where supervision is expected to occur monthly or quarterly. As highlighted by a respondent:

*“There is a gap in regard to frequency of supervision and feedback systems since health facilities should visit the health posts monthly and quarterly.” (KII_SP_01)”*

### Barriers to using supply chain data at last mile

The study found that healthcare workers and managers in Ethiopia’s agrarian and pastoral regions face multiple barriers to effective supply chain data use. These include lack of data quality, absence of data review system, workloads, low motivation, limited Information Communication Technology (ICT) infrastructure, poor internet connectivity, and a shortage of trained personnel as portrayed in **Table 2**. Respondents also reported restricted access to key systems such as DHIS2, which hindered data reconciliation and evidence-based decision-making. Additionally, the absence of standardized procedures and supportive policies contributed to inconsistent practices and limited the use of data for supply chain planning and performance monitoring.

**Table 2:** Barriers and opportunities to utilizing supply chain data.

Barriers	Opportunities
Workload and availability of inadequate supply chain officers	A system for interoperability
Lack of motivation and commitment	A system for data quality assurance
Inadequate supportive supervision and mentorship	Capacity building and training
Lack of power supply, ICT equipment and internet connectivity	Social media Platform for data sharing
Lack of SOPs on supply chain data use	
Limited skill and data literacy	
Lack of quality data and culture of not utilizing data	
Limited access to data and inadequate system interoperability practice	
Lack of performance-based incentives	
Irregular performance and data review meetings	

Furthermore, A key issue overemphasized was the ineffective management structure, particularly within drug and therapeutics committee(DTC) and Performance Monitoring Teams (PMTs), which results in a lack of clear guidance and oversight for data utilization. This is further compounded by a prevailing culture where data is not seen as a valuable tool for decision-making, leading to its underutilization. As highlighted in the following quotes.

*“The big problem that we recently learned is that a performance team is not checking and monitoring pharmacy indicators, both supply chain, pharmacy services and medical device indicators.” (KII_OA_14)*

There are also opportunities where facilities use social platforms such as WhatsApp and Telegram channels to share reports for resupply purposes. A key informant described how social media is being used to support data use:

*“We have created a WhatsApp group and each health center posts its own report there. We communicate with EPSS, and if there is a late facility that did not report, we can see it there.” (KII_SP_19)*

## Discussion

This study identified a range of interconnected bottlenecks hindering last-mile supply chain performance and the availability of health commodities needed for primary health care within an overall service delivery intervention focused on improving RMNCH outcomes. While both pastoral and agrarian regions shared systemic challenges—such as gaps in quantification and budget limitations for procurement—qualitative insights specifically highlighted unique logistical constraints within pastoral contexts. These included challenges with road infrastructure, limited cold chain capacity, and low coverage of electronic logistics management information systems (eLMIS), reflecting the distinct geographical and infrastructural landscape of those regions.

This study highlights poor data quality and limited quantification knowledge as key barriers, similar to findings in Nigeria where inadequate human resources hinder accurate forecasting and procurement of family planning commodities [10]. Similarly, studies conducted in Ethiopia have identified inadequate capacity and critical budget shortages for pharmaceutical procurement as major bottlenecks affecting product availability at the facility level [11]. The prevailing financial limitations that hinder facility level procurement activities, often necessitate a budget-driven approach, where available funds dictate the quantities rather than actual health needs. The situation needs a more efficient utilization of available funds through capacitating facilities on forecasting and supply planning. Inadequacy of budget was mentioned as causes of RMNCH product stockouts [10,11]. Besides, installation of standardized tools for quantification of health products procured through the revolving drug fund mechanisms has paramount importance in the demand planning process. It is evident that poor quantification significantly contributes to stock-outs and wastage of essential health products, as highlighted by a study conducted in Uganda [12]. Another study conducted in Nigeria, emphasized that effective oversight of the procurement of family planning (FP) commodities can significantly reduce frequent stock-outs. This can be achieved through regular needs-based procurement, effective supply planning, and improved use of logistics data [13].

This study identifies transportation constraints as a major barrier to effective product delivery. As detailed under the Inventory Management and Distribution theme in Fig 1, where inadequate transportation, insufficient route optimization, and security challenges are identified as root causes. Contributing factors include inadequate transportation infrastructure, limited access to reliable transport options, geographical and distance-related challenges, the absence of a standardized commodity distribution system from health facilities to health posts, inconsistent delivery schedules, and security concerns. Similar studies conducted in Ethiopia have also highlighted transportation infrastructure limitations as key obstacles to product delivery, ultimately affecting last-mile product availability [14]. Addressing these transportation challenges requires more innovative and context-specific solutions. Strategies such as route optimization can enhance the efficient use of available vehicles. Additionally, developing a transportation model tailored to last-mile delivery—such as introducing motorcycle-based delivery systems, either through third-party logistics providers (3PLs), facility-owned vehicles, or voluntary community groups—may offer practical alternatives. However, any proposed model must be carefully evaluated based on total cost of ownership to ensure long-term sustainability.

This study revealed several key barriers that hinder the effective implementation and operation of Logistics Management Information Systems (LMIS) within health supply chains. These include inadequate data management, lack of appropriate tools, limited technical capacity, poor system interoperability, insufficient quality assurance mechanisms, poor connectivity, and a lack of ownership. Collectively, these issues contribute to inefficiencies in logistics operations and disrupt the consistent availability of essential health commodities. Inadequacy of reporting tools, low reporting rate as well as low data utilization were more prevalent in pastoralist areas as compared with agrarian. Global progress updates and the results of a multicounty assessment conducted by the UN High Commission identified supply chain weaknesses—particularly within LMIS—as major obstacles to ensuring the availability of 13 priority RMNCH products (2). Addressing this requires expanding eLMIS coverage with strict enforcement from administrative units and building staff capacity in supply chain data management. The redesign and implementation of the supply chain modules within the Ethiopian Commodity Health Information System (eCHIS) present an important opportunity to strengthen last mile reporting. Once fully operational, these modules—particularly when accessed through mobile applications—can enable health posts to submit reports online to their respective health centers. This can enhance the timeliness and accuracy of data reporting and improve the overall resupply process at the primary health care level.

Barriers to effective supply chain data utilization in Ethiopia are deeply rooted in systemic challenges such as inadequate infrastructure, limited technical capacity, and insufficient institutional support. These findings are consistent with similar studies in Ethiopia [15–17], which identified poor data quality, low staff motivation, and limited analytical capacity as key constraints to health data use. Similar challenges have been reported in other African countries, where insufficient supervision, lack of feedback, and unclear roles have undermined logistics data utilization in primary health care. For instance, Musa et al. (2023) highlight that health data availability in Africa is both limited and frequently of poor quality, with factors such as inadequate infrastructure and a shortage of resources contributing to this situation [18]. Additionally, a study by the African Health Initiative (2022) found that leadership engagement and available resources at the facility and subnational levels significantly influence the readiness to implement data-driven decision-making processes [19]. Addressing these barriers requires a coordinated approach that includes strengthening infrastructure, investing in staff capacity, and cultivating a culture of data ownership and accountability at all levels of the health system. Addressing the human resource obstacles of low motivation and high workload is crucial for strengthening last-mile commodity management. This requires the strategic deployment of low-cost recognition mechanisms to boost staff morale, coupled with advocacy for evidence-based pharmacy workforce expansion—specifically, recruiting additional staff informed by rigorous workload analysis throughout the health system. Moreover, establishing a Pharmacy Performance Management Team (pPMT) presents a valuable opportunity to strengthen data use at the pharmacy department level. By enabling systematic review of KPIs, pinpointing to causes, and designing targeted interventions, the pPMT can significantly enhance pharmacy performance and contribute to overall supply chain improvements.

Effective coordination and communication are vital for resilient health supply chains; however, this study highlight issues such as fragmented communication between health centres and regional bureaus, absence of zonal structures, and inactive coordination teams, all of which hinder efficient operations. These challenges are compounded by leadership deficiencies and inadequate community engagement. Similar issues have been observed in other African contexts, such as Uganda, where ineffective planning structures and workforce shortages disrupt supply chain performance. Addressing these systemic barriers necessitates establishing clear coordination frameworks, strengthening leadership and governance structures, and implementing regular supervision and community engagement strategies to enhance the resilience and responsiveness of the health supply chain [18–20]. Enhancing existing mechanisms such as the use of WhatsApp in Somali and Telegram in Oromia and Sidama for supply chain communication and information sharing can strengthen coordination mechanisms and improve supply chain performance

The strength of this study lies in allowing participants to share their perspectives in their own words, which enhances authenticity. It involved diverse key informants including managers, providers, and advisors, adding depth to the findings. This study will contribute to the existing body of knowledge, as there is a limitation in the availability of information on last-mile commodity management and product availability. However, purposively selecting only three of the eight project-targeted regions in a country with 14 administrative regions may limit generalizability to other regions and the national context. This study intentionally sampled from both agrarian and pastoralist settings to identify context-specific barriers. However, the analysis revealed significant thematic convergence across most core functions, leading to the application of a unified thematic framework for these shared systemic bottlenecks. While distinct divergences emerged regarding infrastructure, cold chain, and LMIS functionality—which were more pronounced in pastoralist narratives—the study was not designed as a formal comparative assessment. The lack of independent, stratified segmentation for all themes precludes a definitive comparative merit and may have limited the exploration of more subtle, setting-specific nuances.

Addressing these multifaceted challenges requires integrated, context-specific strategies that strengthen infrastructure, human capacity, and data systems while fostering strong coordination and leadership across all levels. Leveraging existing communication platforms and innovative initiatives offers promising pathways to enhance supply chain resilience and ensure sustained availability of essential health commodities at last mile.

## Conclusions

This study highlights interconnected bottlenecks that undermine last-mile supply chain management and uninterrupted product availability in Ethiopia’s pastoral and agrarian settings. Weak demand planning, poor recording and reporting, lack of dedicated pharmaceutical transport, and workforce shortages hinder supply reliability. These are compounded by limited financing, infrastructure barriers, and poor system interoperability. While digital tools like eLMIS offer potential solutions, their impact is limited by low data use and insufficient ownership. Addressing the challenges in the last-mile health supply chain management requires a coordinated, system-wide, and context-tailored response. This approach should integrate technological innovation, infrastructure development, and capacity building, while leveraging digital tools, adopting innovative delivery methods, and fostering cross-sector collaboration to improve the availability and accessibility of essential health commodities and ultimately enhance health outcomes in Ethiopia.

## Supporting information

S1 Fig Barriers to supply chain management. (TIF)

S2 File Data.‌‌ (PDF)
