# Supplementary material for: Barriers of last mile supply chain management and reproductive, maternal, new born, and child health product availability in Ethiopia
Source: PLoS One. 2026 May 14;21(5):e0346924. doi: 10.1371/journal.pone.0346924 (PMC13175355; doi:10.1371/journal.pone.0346924)
Supplement: S2 File — (PDF) [file pone.0346924.s002.pdf]

Transcript 1

**Name of Project:**	[project]
**ID Code**	KII - OA -23
**Type of interview**	KII
**Type of informant**	[Sex]
**Region**	[region]
**Role of the Interviewee**	[coordinator]
**Years of experience**	
**Name of Transcriber:**	[Lecturer]
**Date of Transcription**	August 2024
**Length of interview**	Start time: 0**2: 05 PM** End time: **03: 25 PM**
**Names of Reviewers:**	

Start of interview

I: Can you describe your role and responsibilities within the health supply n system?

R: I started working as a pharmacist in a dispensary unit. Then I attended clinical pharmacy at [University] and established it here. I have been doing drug evaluations. I have been working as a head for a year. I have resigned as we are not agreeing on many things. Now I am working in a dispensary and trying to initiate the clinical pharmacy again. I have been working on supplies for a year. Let me tell you the problems starting from the top. From the drug we requested, we only get 19 or 20%. I can show you the plan. There is an evaluation which shows how much money of the drug we get above 80% and which one is below 80%. We get a maximum of 26 % and minimum of 19%. When there is a stockout, we collect it to present to the management. The current availability of the drug is between 64% and 69% as a country. There are basic drugs that should always be available in the [health facility]. You report it every quarter and receive it at the end of the year. APTS does everything including service reports. We also report all transactions every quarter. The supplies were not provided when they were updating their system. It is becoming very challenging following the malaria outbreak. The [facility] is working very well. It collected around 15 million birr last year. There is a challenge with insurance. Services provided for children under five years are free. The government provides cotrimoxazole, Zink and ORS as program drugs. However, children are being provided Augmentin and Amoxa. The cost is around 400,000 every quarter. These are some of the challenges related to supply. Budget shortage prevents you to follow the standard. If you request to avail it based on case management, you will be rejected due to

budget shortages. If there is no money, you will face stockout. If there is no supply, you cannot get the products based on your monthly consumption. What I have been using as a solution was model pharmacy. We started with a capital of 500,000 birr. During stockout I will request from [resupplying hub] at any time to use it here. Due to this we do not face stockout most of the time. The challenges that are preventing us from reaching the community are; budget, availability of the products at [resupplying hub]. IFRR and HPMRR are being filled out correctly. But you will say it is fine when it is linked to availability. These things prevent you from working based on the standard.

I: You have raised many important things. What about the workforce here? Is that enough? What is their capacity and motivation?

R: now it is good. Previously it was very difficult. If workforce is not sufficient, it will be difficult. Now it is enough. Skill gap is the main problem now. Training is not given on IPLS including [eLMIS] and APTS. Most of us like softcopy and we are training to learn in our own way. However, there is a big difference between getting the training in another place and trying to learn it on your own. We have started implementing [eLMIS]. We are sending the reports using [eLMIS]. There is a skill gap. We are being evaluated without getting the training on how to do it. APTS is being implemented in this [health facility] but I only took the training. Others are using it based on what I tell them.

I: Do you give induction training for new staff?

R: We will discuss this as pharmacy staff. Once they are done with HR. we will give orientation at département level. To start a duty program, he has to know how to do the initiatives. If he is not able to do those things, it will be a risk for him. We will touch these key things during orientation. Last time I requested to have everything digitalized including transactions like [Facility]. For now, workforce is enough. Seventeen pharmacist is required for [Facility] and we are 17 now. The problem is the skill gap. We are trying to overcome it by discussing on it and reading guidelines.

I: Is there any recognition mechanism that is provided as a team?

R: We have it as a pharmacy unit.

I: What do you do? Please tell me about it.

R: we thank each other during our meeting. We will discuss and give comments on every gap. We give recognition twice a year as a role model. Staff will nominate anyone to undertake voting. But it is challenging to do such a thing in the organization. The management body does not like to do anything that is not the guideline. We are always in dispute with them due to such challenges. If you are working on health issues, you have to keep your political role aside and work on your health. Here we are working to save lives and you will be audited and face problems if you are not working properly. As a pharmacy, we are motivating and respecting each other. But there are challenges as a [health facility].

I: Who is this year's role model?

R: (laugh) it is not selected since I resigned. They are suggesting that it should be me. But I am did not agree with that as someone who is fired from his position cannot be a role model. For us, positive thinking is a big award. It is difficult when you bring individual issue to work place.

I: How do you evaluate data quality?

R: Data quality is very important for assuring availability. As I have told you, training is very important. We reached here on our way. You can use this year's data for next year's plan. Training was not provided. No orientation was given. You will bring good things as you learn from challenges. We are doing well with data quality. However, we are facing problems when there are new cases. Our store man tool IPLS training recently.

I: Do you do the quantification every year?

R: Yes

I: what data do you use? How about data quality?

R: Our data source was Bincard and IFRR when we plan for 2016. Let's take central, I will count the number issued over a year from the bind card. We do quantification of each drug. It takes many days. We will count the number and how many times ceftra is issued over the year. Based on that, we use +/- 25 % considering additional cases may exist as emergencies. We use HPMRR which is submitted every 2 months for program drugs. We have a slip that we collected from [resupplying hub]. The system does it for us based on our consumption.

I: What challenge have experienced when doing this quantification?

R: It takes time and you will be very busy. Sometimes you face challenges when bincard is not available for some drugs. If you don't have the bincard, the data for that drug will be false and the accuracy will be lower.

I: have you trained on the quantification tool?

R: No

I: **Who participated in quantification?**

R: Store man, medical director and myself have participated in quantification. I will explain to the finance head about the budget for the plan.

I: **Who approves the quantification?**

R: Medical director will approve it.

I: Is DTC functional?
R: to tell you the truth DTC is not functional.

I: what is the reason?

R: the members are not around most of the time. It is not possible to go for procurement unless DTC agrees on it. You will find only a few individuals. We will discuss if the medical director, matron and HR are available. They say it is due to workload. There must be someone who enforces them to take part. As a pharmacy, the head call and r the meeting. You will be demoralized when they are not coming for 2 and round 3 of the meeting schedule. We have an annual plan. I disseminated it. What to do every quarter, including quantification and what to do in clinical pharmacy are included in the annual plan. If they understand it, DTC is like cornerstone of a house. Nothing is done except DTC. You cannot do quantification and purchase it without the involvement of DTC. I think this is due to misunderstanding. They do not have hint of DTC. I tried to call for the meeting 3 times. I then posted it on the group. I Posted DTC principle considering they may read and understand the concept. I submit KPIs every quarter. To present it, DTC must first evaluate it before presenting it to staff. Thy are not understanding its importance. If they understand it, they will engage more in the activities.

I: What about the budget for procurement?

R: we better not talk about it.

I: How much is budgeted for this year?

R: I don’t know. No one knows. I know the management will be called and informed about the budget from [region] and the internal revenue. Then the total budget will be told to the management. No such practice. We hear from other third parties. There is no transparency. I can also tell them. There is no transparency when it comes to budgets. The federal guideline says, 60 to 70 of internal revenue must be used for drugs. But it is inverse here. Only 30% is used to purchase drugs. This will demoralize you. It is better if you are regularly informed about your budget and how much you have used.

I: What should be done differently to improve quantification and procurement?

R: quantification is the responsibility of pharmacy head, store man, dispensary. Finance will take a big role from DTC members. Medical director should take part as this is what is going to be done in the facility. Laboratory, delivery and OR should also be involved. Quantification is not the role of 3 or 4 people. I plan to purchase the drug and it must match the case that the OR department is planning to do. Quantification must be done when all these things are fulfilled. Finance is a must; you can take pharmacy as the motor of the [health facility] and finance as a fuel. Pharmacy can plan what has to be purchased. However, it is zero if it has no budget for purchasing it. you will be informed as there is no money available when you request for procurement. It is better to work on life saving. There are cases that are done without a plan. For instance, purchasing drugs must be prioritized over constructing buildings. People are not understanding this. They are focusing on politics. If you purchase accordingly to your plan, you can assure availability. Unless you assure this availability, you cannot talk about saving lives.

I: How is transportation and distribution of RMNCH commodities?

R: logistic system is very low. The challenge we are facing here also exists there too. I can show you the HPMRR. We were struggling with them to address. I don't know the reason but, the standard and what we are doing is different. They are not providing you based on your request, you fill out an HPMRR indicating what you have consumed and what you need for the next 2 months. What you filled in the HPMRR is different from what they provided you. It is not available there. For example, the HPMRR which shows the artesunate we consumed based on the case which is reported through DHIS2. You can ask for 1000 but they give you 150 say it was not available. This is what you request as an emergency order. They will supply you after 20 or 25 days. There is such a problem from [resupplying hub] side. The one you requested using HPMRR and [eLMIS] is different from what they supply you. For instance, under five TB drugs are not available. I don't know whether they are using the adult's drug. However, it is available from the health centers. It is overstocked there. This happens as you requested without quantification or plan. You find in health center while it is stockout from here and [RESUPPLYING HUB]. I use any means to

increase its availability. I called the health centers to ask them for availability of the drugs. They say it has been a year since it is in stock.

I: Are they bringing it here or you go to collect it?

R: They will bring it. we collect it if it is an emergency. We go for Coartem and Artesunate. [RESUPPLYING HUB]. We were going for it repeatedly. We face more stockout of Coartem, artesunate and primaquine. The requested amount was different from what they provide you.

I: How do you evaluate the storage capacity?

R: I asked for the store with a letter. I explained it to people from [region] and zone. They responded we have planned to work on it. if you check it is overcrowded and does not the standard.

I: What challenge do you experience to transport?

R: There is no big challenge. I will go and collect it if they are not bringing it. sometimes you cannot get a car and should wait until you get a car.

I: Is there any shortage of LMIS implementation formats like Bincard, HPMRR IFRR?

R: Bincard is provided from [RESUPPLYING HUB]. They also give us stock cards. We print the IFRR.

I: What about its use in units including pharmacy units. Is the family and MCH filling out IFRR properly? Are they sticking to the schedule?

R: IFRR is filled in dispensary, emergency, OPD and IPD. IFRR is being filled in with other units like the laboratory. The problem is a bincard. They are not filled out. What we don’t have to hide is, there is work load at dispensary and laboratory unit. The standard indicates that every drug should match with bincard.

I: Do you approve of the IFRR?

R: Yes, they approve of it?

I: Do they request based on the schedule? how do you see the quality?

R: the problem is with availability. You can provide what they requested when you have the drug. If you are not giving, they what they are requesting, they are not asking for it. The facility is not purchasing it based on our request. I only provide them available things. They are coming based on their schedule but the drug is not available. It was challenging previously. We discussed it at the management level and now it is corrected. Now, the challenge is availability of drugs.

I: Do you support the units on how to fill IFRR and bincard?

R: We will give them orientation on IFRR.

I: Do you do stock status analysis to minimize wastage and stock out? You told us you implemented APTS. So, you are doing inventory every month or 3 months. Do you do stack status analysis based on that?

R: we do our stock status analysis. When we have near expire drugs, we use FIFO to use those drugs first. W also discuss with GPs to give priority to those drugs. We give them the list those drug to order before it is expired. We will discuss on it during the morning session.

I: What about the transfer and exchange of the drug?

R: we do have such practices with other facilities. If we have drugs that are near expire, we will transfer it using model. If it is more consumed by other facilities the drug that is over here, we will transfer it. there are drugs that are not used here but the health centers using it more. There are program drugs that come over. They will bring those drugs without your request. If you say no,

they will complain as if you refused to accept it. if you don't accept it, they will make it challenging when you ask them for the next time. They will not give you the products on time. We accept it and then transfer it to health centers using a formal format and model.

I: Is there any drug which has expired as a result of overstock?

R: I think it is IUCD. IUCD was overstock and became expired. We didn't request for it. (pause) some people gave it to the CEO and he brought it here. Only a few people use it here. First of all, you have to know how this thing is accepted in the community before ordering it. I tried not to receive it but was not successful. The community uses the short term most of the time.

I: This can be a push system. What other reason can you mention?

R: There might be a problem with the quantification. If there is quantification error and low data quality, we may face that challenge. Our work process might be another problem. Ordering the same thing repeatedly can cause it.

I: Is the electronics LMIS you are using is [eLMIS 1]?

R: Yes

I: what are the its implementation challenges? Are there any challenges with its functionality and infrastructure?

R: No problem with the implementation of [eLMIS]. You can observe it. I will call (his colleague's name) to show you. The only challenge is its not matching with the bincard. We do inventory and send reports. The problem is with bincard. I informed them about the matching and it will be resolved in the future. It might be solved now. I am telling you about the time when I am in the position.

I: You told us that you are reporting supply performance and pharmacy activities as M&E. Is there a platform you use to review this data?

R: There is PMT.

I: were a member?

R: Yes

I: How often do you have meetings?

R: Monthly

I: Are pharmacy indicators reviewed.

R: Yes. But sometimes we face problems. You will be asked to report false data. They are asking you to include what is not done. I refuse to do so as I will be responsible for that false data if cross-checking is done. I don’t have to report what is not done.

I: Do you report all pharmacy indicators through DHIS 2?

R: Yes, there are KPIs

I: What about the M&E? to whom do you report it?

R: To the zone. The DHIS2 Is reported monthly.

I: DO you present these indicators to PMT?

R: You cannot present it to PMT. That is the role of HMIS.

I: What do you do you think should be improved. What we expect from PMT is you review the performance and identify problems. Then you prioritize those problems, design solution and take action on that problem after developing action plan. Do you do it this way?

R: It is not this way. We discussed on the identified problem but, taking action after developing an action plan is not practiced.

I: Is there any support provided from the management body to strengthen supply n activities? it may be training, recognition or reviewing the performance.

R: They will only check it when you beg them to see our performance.

I: Is there any additional major challenges that you can mention on generating and using the supply data?

R: I am happy to show you. we have all the data. There are action plans and principles. At least people need to give you, their ear. They may not accept your idea. They are not happy to hear from us.

I: What about the technical support provided by zone, region or partners?

R: It is very good. people from zone are supporting us. They give us comments and tell us how to correct the problems. [region] is also supporting us. They understand your idea of not getting acceptance here. They understand how improving this activity is important. You need to understand more before deciding to take action. The facility needs someone who has detailed knowledge about health. He will know the value of drugs and their consumption. It should not be focusing on construction. The two units are very important for the availability of the drugs.

I: Can you tell us any best practices that can be scaled?

R: To increase availability, we will transfer the drugs with all health centers in (specific Woreda) and other woredas. In addition to increasing availability, we give and take near expiry from them to reduce the wastage. We also conducted rational drug use. We did a drug use evaluation including the level of antibiotic use, injection status and availability at each department. Using a model can be considered as best practice. Model pharmacy is helping this facility stand strong. If there is stockout here, we use from the model pharmacy. We buy stockout drugs using a model pharmacy to make it available for the community. We then return to the model pharmacy based on its price. This is what we have been doing to make availability increased.

I: Thank you very much. Thank you for your time and contribution.

End of interview

Transcript (2)

**Name of Project:**	[project]
**ID Code**	KII_P-12
**Type of interview**	KII
**Type of informant**	[sex]
**Region**	[region]
**Role of the Interviewee**	Advisor
**Years of experience**	
**Name of Transcriber:**	(Lecturer]
**Date of Transcription**	August 2024
**Length of interview**	Start time: 9:05 End time: 11:09
**Names of Reviewers:**	

Start of interview

I: Could you describe your role and responsibilities within the health supply n system?

R: I oversee program activities for as a senior technical advisor. Although it is a kind of intervention, at the end of the day, it will improve the utilization of digital health. Thus, similar to marketing, my job is to observe customer demand from the customer's perspective. Some activities can be practical and optimized from the program side. It also includes addressing what the customer requested. Generally speaking, my job is to provide technical support and match program requirements with available technologies. Furthermore, by offering leadership and technical oversight for the essential skills of supply n operations, such as LMIS and the entire supply n, I oversee supply n interventions for the project.

I: How do you perceive the overall effectiveness of the supply management system?

R: Since it might lead to an external entity, it is challenging to say whether it works or not. Really, how are we going to quantify it? I was part of the IPLS inception team, I am afraid of creating bias. In general, the flow of financial resources, information, and commodities is what the supply n is all about. I think we are talking about the public sector.

I: Yes

R: Well, based on historical evidence, IPLS is applied and [SUPPLYING HUB] is established. Demand-driven program supplies are being provided, and IPLS is currently being implemented. I

can say that we were successful because we made significant strides toward making our products available. That is to say, goods—particularly program goods—are getting to patients and healthcare facilities. However, we need to go a long way if we only take the necessities. For our service to be considered effective, optimization efforts must be made. We are in good status, at least from the program side.

I: What about timely delivery of commodities, facility readiness in reporting, and data quality?

R: Obviously, there is a system challenge. This system challenge affects the entire health system as well as the supply [partner. Service delivery, supply n management, and all other activities have challenges. To see these thematically, there are challenges from people's sides. It includes motivation, and sometimes capacity. After the capacity problem is resolved, turnover occurs. problem. Second, there are issues related to the procedure. Is the process that we have implemented optimal? Is it progressing to the expected level? The other comes from the realm of technology. Is the technology implemented optimally and with consideration for the needs of the customer? This should be evaluated. The other is related to the infrastructure. Is the infrastructure of the nation suitable for supply management and procurement? All of these things, including its governance, ought to be examined. To claim that it is delivered on time, we must establish a benchmark based on international standards. As a country, there are multiple challenges. Currency and policy issues are included. I answer "no" if you inquire about effectiveness in terms of timely delivery. However, based on the existing context, it can be close to efficiency, and to be effective, we have a long way to go**.**

I: What about supply n workforce staffing and capacity? Presence of logistic teams, adequacy of human resources?

R: Obviously, it is not. The big issue for me is, that I don’t think understanding, prioritizing, and budgeting of logistic contributions and logistics systems at a higher level is enough and has an impact. For example, if there is no infrastructure, you cannot provide service if there is no logistics. There is a big challenge in terms of recruiting a logistics workforce. So, we need to have a mechanism to advocate for sufficient resources to assign logistics professionals at all levels. So, there are huge challenges from the leadership side in terms of prioritizing and assigning

professionals. Secondly, those who are on the job are not capacitated. If you ask me whether there is a set up for those who started working after graduating, there might be facilities that are doing well. But as a system, I think it is not good enough now. There are challenges in terms of capacitating the workforce by giving priority. It is also not possible to say the setup is enough. Another thing is we manage the supply by giving it an autonomous entity. Using that entity consistently has helped us a lot in creating a specialized workforce by getting support from donors and implementing partners. From that angle, it is also promoting other sectors. For example, the presence of [SUPPLYING HUB] is recognized by the ministry after confirming its importance. But if we take other countries as the benchmark, we are not done yet about supply n management.

I: What about mentorship programs, performance monitoring, and recognition mechanisms?

R: It is similar to other service delivery programs. The capacity-building activities may not be to the level that is required for supply management. There is a situation in which the mentorship programs for service delivery also incorporate the supply.

I: What about the management support?

R: For me, it is comsi comsa. Things are remaining. There is a logistics department, but to say there is management support, the management understands the role of supply management in service delivery, prioritizes things, and makes the worker accountable for the activities. If this thing is very narrow, When IPLS was designed, it suggested a pharmacy head sign and the facility head confirmed it. This is how to make someone accountable for the problem that happens as a result of the filled HPMRR. Does he know what he is signing? If we make such accountability from top to lower level and start to recognize that, it will be better. The logistic issue is only remembered when there is a stockout. For instance, you may avail 1000 items, but as far as the service being provided no one can remember it. It might be the nature of the business. They will remember you only when there is a stockout. You see my point. They say you are not availing of the products, what are you doing? No one recognizes the steps you went through to make it available. That is a big challenge. So, it is difficult as far as we cannot make what it takes to avail even one individual product starting from demand planning to supply fulfillment. As far as we

cannot make what demand planning means to people, they going to take it for granted. This is a tricky business. People remember you only when there is a problem. We need to work hard to change that mindset.

I: Can you describe the current quantification and procurement process for RMNCH commodities?

R: If we talk about quantification technically, it has a preparation phase, forecasting phase, and supply plan phase. You have to check and clean the data and plan your demand based on program targets. You will plan for the amount you need based on the assumption and the budget you need for that amount of product. You will consider the lead time. You will do the warehouse capacity. But, RMNCH commodities are not like other programs, it is mixed. Some groups of products such as family planning products are fully supplied even when donor support is reduced. These products are provided to clients free of charge. These products are costing the country including the transportation cost. Commodity quantification is done centrally based on the aggregated demand, and there is no facility contribution for such procurement. There are also non-full supplies. For these non-full supplies, the facility does its demand plan and buys itself. The facility buys these products following the same procedure and forecasting it. Problems happen at this place as the facilities are in resource constraints. Everywhere, full supply is difficult. Even it is not possible in America and developed European countries, We need to prioritize things; it needs to follow technical procedures. There are problems with technical procedures and prioritizing, and I think it is the area that needs to be worked on. I don't even think the methodology we are following is the right one. I don't think only giving TOT and sharing the Excel with them will bring change. I think we need to have a mechanism to overcome this. We can do this based on historical demand and service level agreement. We can use a mechanism of delegating for some of them and make others do it on their own. The big challenge is in this area. So, the process is already there, it is centralized for some and decentralized for the other one. There should be a clear assessment of the demand for no full supplies of RMNCH commodities.

I: What about the challenges related to the quality of the data??

R: it is similar to what is mentioned earlier. It is related to manpower, process, technology, and other things. When we say human power aspect, it also includes the funny business. I mean, we

include it here if you are making the data quality poor intentionally. For me, everything is garbage unless and otherwise you are accounted to the system that allows you to collect, aggregate, and report logistics data that are required for decision-making, it depends on the LMIS. So if you are not well accounted for the LMIS system you are using, you are doing that thing based on your interest. When we say well-accounted, we mean that you should be responsible if you do the wrong thing. There should be a monitoring mechanism. If you are not accountable for it, what you forecast will be garbage in garbage out. There are sufficient resources but stockouts are being reported. Wastages are being reported. This is due to the system that is not accounted for. You have to develop a hook and work on capacity building. I am not saying there is no capacity-building activity but it has to be checked. So, there should be a well-accounted system that allows you to capture the right information for decision making. If the problem is from the data source, at the end of the day, the forecasting will be poor. If there is no quality data, forecasting accuracy will be very low, even if you use AI (Artificial Intelligence).

I: What about budgeting issues?

R: Honestly, I do not think the budget can be a major issue this time around as long as we can prioritize and defend it. As previously stated, resource constraints are present even in the United States. The nation has many priorities, so there are not enough resources to make everything available. Are we making efficient use of the resources at our disposal? should be the question. We are not using the data, in my opinion—another person may disagree. We don’t even know our demand. Given that I am in charge of the facility, I have to be aware of the requirements for every service. Following that, as an entity, determine the facility's service level, priority, and country, and then have a service level agreement. This is what we call ABC analysis. The idea behind internalizing service level agreements is that I can use all of this group of products within our budget and, after using the product, reduce the safety stock level by 80% of the time. So, I will be evaluated based on these criteria. I will be asked if I have a stockout of less than 80% after planning for that percentage. That means 80% is 100% for that group of products. If that kind of prioritization and analysis is not done, we should not associate it with a budget shortage.

I: **Let us talk about transportation and distribution. How does the transportation and distribution system for RMNCH commodities?**

R: This is a trick question. I know [SUPPLYING HUB] has a transportation service. I mean, I do not address the question of whether or not they would find the business model advantageous. Do we really need to buy all of these vehicles, keep them all maintained, and pay for their depreciation? Since it was not accounting, its efficiency and optimality are questionable. There are options, like outsourcing it and focusing on contract management. I prefer that we view it from the top down. Is it optimal to buy all those cars and do the distribution? This type of logistics system business model seems unlikely to me. Anyway, in terms of using the existing system optimally, we are not using it optimally, there is room for improvement even in this setup. They should enhance it and adopt a different perspective on the business model, incorporating some outsourcing, since they have made significant progress in comparison to earlier times. I think they are performing better than any other sector in terms of reaching the facilities. Although whether they have fulfilled customer service requirements is another question, there is a practice that even other countries can learn from. I mean, there are best practices here and there. The transportation issue comes after that. For instance, when we talk about preventive service, it takes place in a lower-level setup. Once the product reaches the health center, it is very challenging to reach the health post. The product reaches there sometimes using donkeys, or motorbikes, or volunteers. There is big challenges and gaps in terms of creating a modality that allows her to use products. There are facilities that [SUPPLYING HUB] reaches directly or indirectly. Once the indirect product reaches the woreda, there is no standard practice. In some places, the woredas distribute it at their convenience. at some place, it will expire at Woreda. It is easy to say, no transportation system at the facility level. But there are best practices, such as motorcycle initiatives. The transportation capacity and availing transportation mechanisms are at a lower level.

I: what do you recommend in this regard?

R: If possible, it is better not to include the woreda in this system and facilitate direct delivery. I was engaging in the early design of IPLS and the role of the woredas was to oversee and supervise it. The woreda has no role in the direct delivery of commodities. We introduced the concept of woreda passthrough. That means, they collect, aggregate, and send facility information, then [SUPPLYING HUB] will send the packed products for each facility, and their role is just transferring it. There were no interventions and programs that considered the woredas, even when multiple partners had been supporting them. The support was at the facility and [SUPPLYING

HUB] level. Woredas are using the warehouse of the nearby health facility, which is compromising the facility's inventory management and business process. I think the last mile should push the current speed. With the existing transportation facility, it can reach many facilities through last-mile delivery. One thing that has to be known is, we don't have to use the current strategy for last-mile delivery. Most of the time in the supply, it is a trade-off. The moment you increase distribution frequency, you reduce warehouse costs and increase transportation costs. However, you have to see what effect it is bringing on overall supply management. Whatever you do as part of a last-mile delivery strategy, emanates from the overall strategy. That means it should be done in a way that cannot compromise overall supply management. For the health post, there are multiple initiatives. For example, currently, we have three categories of health posts; comprehensive, basic, and merged. The mentality of the previous time, when the informed push model was implemented for health posts, is different now. For the compressive health post, it is not clear whether we have to use HPMRR, which is through the inform push model, or not. We need to reframe that since they might have a pharmacy and a pharmacy store manager. Other than this, for others, there are so many best practices. There is leveraging of the private sector; through motorcycle initiatives and others. So, we need to have this list in a table and apply it based on the context. As a single shirt is not for everyone, we have to adopt multiple scenarios according to the context. When we adopt this scenario, we have to look at the resources of the context. If the resource of a specific context is not known, it will lead to a significant loss of commodities. For instance, if we say Woreda X or Zone X should use this strategy to deliver to the health post, it should be planned, budgeted, implemented, and monitored.

I: We need to hear more about private sector engagement, and motorcycle initiatives; it is blurred for us. I am happy if you list them down and tell us in detail. Last time we were in {zone} it was very difficult to reach the health center if it was rainy. Reaching the health post is unthinkable.

R: You know what, For such a problem, a human-centered approach matter. I can say let me design a variable inventory control and inventory management system that allows them to collect supplies for six months for the woredas that have such problems. Another person may say Why don't we send it to them by using a drone. Here, you are asking me two things at once. You are saying they have no storage capacity and access. Here are the options: let's build a store for them, send the

products using a drone, or distribute them and let them do whatever they like. There are multiple scenarios if you are asking me about how to improve IPLS. Because certain facilities have capacity gaps, we might use vendor-managed inventory for them. vendor-managed inventory I can store and control, but 3PL providers can do the inventory just like Walmart. That can be one option, as it was tried in Zambia and became successful. It can be a variable inventory. policy. The country's inventory policy says the facility requests it every two months. But if we take facilities like {Bigger facilities), we may request them every two weeks based on their storage capacity and services. You might offer certain types of facilities once every three months. That means it varies. Regarding health posts, it will change according to the situation. It can engage small-scale enterprises as PLCs. This requires multisectoral involvement. It has many risks. Health care providers are stealing goods these days, and giving them to a third party to carry it a lot of risks. You know what, It needs to do a risk-benefit analysis. Outsourcing is the ideal solution when you have systems in place to track and monitor the delivery of services and you have a distinct entity that cannot disappear with the goods. I feel it differs based on the context. For instance, what should be done to the issue you have raised about [zone]? It may be making motorcycle deliveries to the health post, or thinking about using drone technology without limiting our minds.

I: Don't forget about the inventory. Is it feasible to optimize the inventory activity of health posts?

R: No. By design, the health post-resupply is an informed push model. The document states that the informed push model should be used in the following situations: 1) when there are many health professionals and there are insufficient resources to train them all to improve the health supply when that person's core competency is not in the area of the supply . When these two things happen, you will take the supply role to a higher level. The HPMRR says the health posts report, and the health center calculates the resupply based on the information provided by the health posts. The HEW has this minimum level of training for every inventory activity, and she may be busy with other activities. Her level of knowledge can also relate to this. Therefore, it is also better if you give them a small amount of the commodity in volume. As far as possible, it is preferable to keep commodity volume and commodity management activities using the current methodology. We have to optimize efficiency, including her linkage with the store manager. A considerable portion of health posts will be comprehensive, so they must be regarded as health centers. For basic health

posts, it is better if we continue by maintaining the existing procedure. The strategy was designed considering the health extension workers collect their salary from the health center before it is paid through the bank. So, one of the requirements to get her salary was to report to the HPMRR. We were trying to use her salary as a hook to get a health post supply report. It worked, but it was not very good. Thus, we need to consider alternative mechanisms, like assuming the role of supervisor. If she brought the HPMRR, and collected her salary, but provided no transportation for the commodity, this would not add any value. So, we need to have another strategy where the health center is accounted for, planned, and budgeted to directly deliver to the health posts. It can be done within this basic reporting. Even within this, we can also think about segmenting. There are various segmentations and optimizations of a system that need to be taken into account.

I: I was about to say I am done but you are raising interesting points at the end. Can you explain a little bit about the segmentation?

R: It is a good concept. I report HPMRR, so there are different groups of products from that health center. Most of the time, due to sensitive product nature, there are products that we need not to carry. There are products that we don't need them keep due to their bulkiness. We have to identify products she has to collect every month based on her report. There are products that we don't want; they have to carry them. I am happy not to talk about this. To be honest, such things need independent projects that can be implemented rather than within other activity. I am happy not to raise it here.

I: What are the main implementation challenges of the IPLS?

R: I remember IPLS was launched at the end of 2009 at the [Hotel] after giving TOT. even the it was named by that time. first, it was called NIPLS, second, it was called NPLS, and thirdly it was decided to call it IPLS. More than 50 thousand individuals were trained, and many reporting tools were printed and distributed, and included in health science and university curricula. Just like HSDP and HSTP, IPLS was a common term used in health facility. It is about how someone who has been a part of the health system for the last five, ten, fifteen, or twenty years assesses IPLS. As we compare it with the time, we first implemented it with this time we have gone better. There is good improvement, but there is still room to improve it more. IPLS is all about optimizing the logistic system, including recording and reporting. For me, it is laughable. to hear when people

say it is difficult. A person who completes high school does it. It is a simple addition-and-subtraction activity. Although there is a capacity issue, I feel it is minimal. In terms of optimizing commodity flow, it is questionable. Still, there is HPMRR and commodity resupply data quality issue. The data quality issue is not only the issue of IPLS. HMIS and other health information systems also have data quality issues. It also has behavioral issues, facility-level issues, and accountability issues, just like any other system. As a system, it is recognized and accepted. IPLS elements are also seen in the performance monitoring framework. Although there are challenges with generating quality data, it is in good status in terms of implementation. However, the health post part is removed from IPLS system since there is no hook. The facility reports HPMRR to [SUPPLYING HUB] and gets the supplies. So, bringing that HPMRR they will implement IPLS. Whether they report false data or not depends on people's level of commitment. I see IPLS like this. You may see it differently; it depends on whether you joined the health system in the last 5 10 or 15 years.

I: what are challenges with its implementation?

R: It can be from HR challenges, including commitment, the culture of data use for decision-making, and funny business. Is that not? There are issues related to the process. Does it make sense to provide supplies for four months every two months to (Bigger Hospital]? So, it needs optimization and segmentation. There are issues of reporting, technologies, and infrastructure. There are many challenges, just like other systems, and it needs to be contextualized. The big challenge is related to accountability and leadership.

R: eLMIS is related to the technology we have raised earlier. When we talk about LMIS, it is about a system that includes paper, and technology that enables proper data collection, aggregation, and reporting. eLMIS is the way we have a mechanism to leverage proper technology to optimize business processes and feasibility. So, if we take eLMIS we started it before many African countries but, now we are behind those countries that learned from us. When we see the eLMIS, we have upstream and downstream eLMS, …. we have visibility. platform. So, the upstream is the one that is called [eLMIS at (RESUPPLYING HUB)] and supports the activity of [SUPPLYING HUB]. Vitus application has been using its own process with multiple enhancements, and [SUPPLYING HUB] has been using it very well. Now, to improve

organizational maturity, it is in transition to use another system. So, we can see the implementation of eLMIS and the supply n in this way at the upper stream. There were multiple applications at a lower level based on the context. If we take [eLMIS] was first implemented at [Hospital] in 2009. It started with a small application and grew from time to time, now it is being used as a web-based application. So, it was designed to optimize the facility-based inventory management system. As you know software upgraded based on requirements. It is being upgraded in a way that cannot affect the requirements. Currently, in terms of deployment and implementation, it is deployed in around 1300 health facilities. However, due to the issue I have raised earlier; HR, commitment, turnover, change management, leadership and accountability, the approach we have used to implement it, and how the leadership acceptance can make its implementation vary. Its implementation can be good in some facility, it can be on and off in another facility, while it not implemented in other facilities. So, just like other systems, its implementation level varies. Since this system is a transactional system and need consistent monitoring, it cannot be stable as long as the leadership and accountability is properly exist. For example, DHIS 2 is a reporting system and you enter the data from different places once a month. For this one you to enter the data daily, otherwise, the data will not the correct. So, we have to enforce that practice. So, we have to identify the hooks and work on digital HPMRR, and digital voucher.

I**: How effective is the inventory management system in woredas, health facilities and health posts?**

R: it is similar to the earlier points on IPLS implementation status. It varies among the facilities. It depends on facility demand, commitments, and capacities. For example, if don't have sufficient storage but, want to manage it optimally, it's going to be difficult. The storage premise should be good and have capacity, commitment, and need. Implementing inventory management at big hospitals and lower-level health centers varies. It varies based on need, premises, and capacity, and leadership accountability. It varies based on set up where some facilities do better while others do not.

I: what about supportive supervision?

R: Supportive supervision is also being provided. However, as long as there is no accountability, such supervision and mentorship cannot bring about change. He can do it today and stop it the next

day. There are multiple supports for supportive supervision. Many facilities are working better following the supportive supervision. There are facilities that are not doing anything at all. The big thing is working on leadership... leadership.

I: what about the understanding of HEWs on the stock-out triggering points?

R: Honestly speaking, it is what I said earlier. By design, when IPLS introduces informed push model**,** commodity management practice should be minimal of minimal at the health post level. That means they will be resupplied every month. The way they think about emergencies… for example if there is a malaria outbreak, she may be informed to layman level to request for 8 commodities weekly by checking the trend to see if she had been receiving 4 items during the normal time. We are not expecting them to have perfect management. We just want them to have basic of basic knowledge. We inform her to keep the commodities from dust and sunlight, and keep it locked. They will also be informed to request their commodities frequently. There is a standard for health posts in the IPLS and based on that standard, the health extension worker supervisor visits them with store manager and advise them on how to use their stock.

I: What are the barriers and opportunities to strengthen the health post-resupply system

R: it is making health extension workers know more about supply n management. The other important opportunity is the module on supply management in eCHIS. So, it needs to facilitate facilities that implement eHIS use the module. The other thing is to strengthen the IPLS strategy that recommends resupply management by translating the tools into the local language and making them learn about them. It needs to devise a supporting mechanism in which any person who provides community-based support can have budgeted intervention for optimizing supply management as part of compressive package.

I: What are the internal reporting and resupply system's challenges with the facility?

R: Still this is also connected with IPLS implementation. IPLS has three points; information optimization, storage management practices, and internal business processes. So, part of IPLS implementation is about introducing store managers, pharmacy, and dispensing units come to gather and decide who should request every week or month by filling IFRR. Here important thing is making the management accept it and work on it very well. Big dispensing units do not have big

sore and it can be difficult to ask for reports and collect 100 products every week. So, the segmentation technique we discussed on health post resupply can be applied here by informing them you will collect these products weekly and the other will be collected after bringing the report. Secondly, it also needs to automate this reporting system. Such are existing at many places where Excel is used. It is better if we enter the data, then it calculates by itself and we can do the resupply. The third one is promoting the implementation of APTS in which IFRR automatically enters into the system. Generally, the system should first be organized according to what is recommended in IPLS. Then you will work on optimizing that system, including mini-automation and compressive automation

I: **How are supply data generated, shared, and utilized for decision-making within the health administrative units and facilities?**

R: we are talking about supply. It also depends also level of maturity. Some facilities are implementing IPLS very well. They are using their data very well. Whether the data is collected manually or eLMIS, it is being used for decision-making to improve the availability. In terms of making the data institutionalized, you already know a PMT system. It is a performance management system at institution that should be leveraged, even when practice is good or not good. When the practice is not good, we can enforce the PMT or DTC. In facilities that implement [eLMIS], the store manager reports near-expiring products to his leader. He then checks it and say let's redistribute it different unit and inform them to give priority for prescribing those products. But we can enforce PMT to use this information at the institution level to make this data used continuously. Its practice varies from facility to facility. In some places, the practice is comsi comsa.

I: How can we know the facilities? Are they health center or hospitals?

R: It varies? Sometimes you can find a better health center or the worst hospital. It depends on the accountability of a person. As far as I can send the HPMRR, no one can ask me. I don't if things are changing now. Is anyone who can ask me if I am not doing it? No one can. However, level of data use depends on the facility. At the hospital level where the flow is high, it may be higher. Most of the time it depends on individuals, and that is what you have to bring to leadership and make them accountable.

I: What are the most successful examples of best practices in the supply n that could be scaled or replicated?

R: One more challenge that I forgot. In terms of RDF commodities, whether it is in IPLS or not, the information flow should be checked very well. There is a big gap with that one. You can continue now.

I: **What are best practices in the supply n that could be scaled or replicated?**

R: This is one of it. We have to see and implement the gained improvement to the RDF and others. There are initiatives here and there. So, we have to think about how we can use them comprehensively. We have to see market sensing very well. we have to see the concept of one shelf for everyone, we have to think about a segmented approach and differentiated delivery modalities. Having the practice at hand we have to see the mechanism by which optimizing are optimized. Even when we talk about the best practices, we have to see them from the top, including globally and in Africa. We have to work on it by having benchmarks at each level. We have to think about having proper hooks and bringing accountability on the implementation of LMIS. It needs to set a thematic area, budget and plan and implement it. We have no problem with planning, the problem is when we cascade to the lower level. I mean we have the recommendations in everywhere. If you open documents, you will see interesting recommendations. The question should be whether we have the capacity to implement it or not at lower level.

End of interview

Transcript 3

**Name of Project:**	[project]
**ID Code**	KII -OA -02
**Type of interview**	KII
**Type of informant**	[sex]
**Region**	[region]
**Role of the Interviewee**	HEW
**Years of experience**	15

**Name of Transcriber:**	[lecturer]
**Date of Transcription**	August 2024
**Names of Reviewers:**	

Start of interview

I: **Can you describe your role and responsibilities within the health post?**

R: I am health extension worker at [Health Post}. I have worked as a level 4 health extension worker for about 15 years.

I: **As we have tried to introduce about the project, the objective of this study is to assess bottlenecks for supply management. Regarding this, how do you perceive the overall effectiveness of the supply n management system?**

R: There are many problems regarding the availability of supplies. First, we are not getting what we have requested. Secondly, we are not getting what we have asked for on time. Such a situation is affecting our work. For example, if we take family planning, a woman can be pregnant without a plan when we are not providing it to her. If we take EPI, a mother brings her 45-day-old baby for immunization, and due to the unavailability of a vaccine, she returns home without vaccinating her child.

I: How do you see your facility's readiness to report supply n reports and data quality?

R: There are also problems from our side. We cannot say all health extension workers are the same. There is a problem with requesting the commodities using HPMRR. It is our responsibility to request before stockout, this is also our problem. The main problem is the unavailability of supplies at the facilities. For instance, this week, we are facing a shortage of malaria kits and coartem, while malaria is a current problem as a country. We were informed to do a test [malaria test] for 21 people per day. To do so, we at least have to receive that number of tubes. However, we are only getting 50 of the tubes. So, what we are doing is only doing the test for those we consider to be very sick. We are doing the test for only people who feel to have malaria. So, in addition to our problem with requests on time, there are also problems with the availability of the supplies.

I: what is the main reason for your side?

R: We sometimes may forget to do it. I can also say the problem of knowing how to request it. For instance, it's supposed to be requested before it is finished. This may be due to a knowledge gap. There is also knowledge gap with filling the HPMRR form. Everyone may not how to answer HPMRR questions. It is difficult. … if we use it all the time it becomes easy. We are not using it continuously, as here (Health Center) is also giving as the commodities without HPMRR. They ask for HPMRR rarely. If they refuse to provide any supply without HPMRR, everyone will be experienced in using it and it became easy. Previously we were receiving it after filling out the form. Now, the pharmacist here is not giving as based on our request without asking HPMRR. So, there is problem with filling the HPMRR. The form asks to fill A, B, C and there is a problem of understanding and filling it. Previously they had been showing us how to fill it. But the new pharmacist is not asking for it. Yesterday I received 5 pills and 5 Iron from this health center, I didn't sign anything or register. This will create forgetting to use HPMRR over time. So, the problems with health extension workers are, failing to request on time, and not filling HPMRR.

I: Has he asked you for HPMRR yesterday?

R: no

I: Have you filled it and brought here?

R: no, we don’t have the form.

I: Is this gap also among other health extension workers working with you?

R: you mean problem of filling the HPMRR?

I: Yes

R: yes, there is a gap. Everyone can understand how to fill out the HPMRR. I had started to understand it by asking, but now I am forgetting how to use it. As I see others, they are facing difficulty to fill it.

I: Who showed you how to use HPMRR?

R: A pharmacist who had been working here. I asked him how to fill it. I had been filling it

regularly. When we here monthly with the form, and we collect the supplies after making it signed by the director. Now we are receiving without any report.

I: when was the last time you filled the HPMRR?

R: Now it is about a year.

I: How does the transportation and distribution system for RMNCH commodities, including vaccines at the health post?

I: have you received support at your health post on how to fill bin card, HPMRR?

R: No

I: What about the pad? Is it available?

R: yes, there is shortage. We don't have it now.

I: was the form you filled being approved?

R: yes it will be checked and approved by the pharmacist.

I: Have you received any training on this issue?

R: we haven't received any training.

I: How does the transportation and distribution system for RMNCH commodities, including vaccines at the health post?

R: we are facing big problem with transportation. Our health post was supposed to be under Seka health center. I have asked this at woreda level in the presence of our woreda health office head. Coming here is difficult for us, we pay 80 birr.

I: you mean for a round trip?

R: Yes round. We pay 40 birrs when come here and 4o birr when we return. We pay 20 birr for motorbike to come to (woreda), then we pay 20 birrs for Bajaj to arrive here. Then 40 birr to return

to the health post. So, we spend 80 birr to come here and return back to the health hosp. additionally, we are also wasting our time which is more important than that money. If we stay here for about 5 hours, we are wasting our time than that 80 birr. I don't know the reason, but our health post is supposed to be under [health facility]. Our health post is next to [health facility].. Receiving everything from here is tiresome and time-consuming for us.

I: Are they directly delivering the supplies to your health post?

R: No one has brought it so far. We come here and collect it. For example, if our health post is next to a road, they might have provided us our share and pass.

I: are you receiving the supplies monthly?

R: As I told you earlier, we came here to submit the report on 20 (date). We arrange it with the reporting date, we prefer to take the supplies at this time. Previously, after a meeting on our report, we took our supplies including cards like family planning and EPI cards. This means there was a schedule. Nowadays, we are not filling HPMRR and getting the supplies we request. For instance, yesterday I was here to receive family planning card and EPI vaccines. I have collected the cards but, not the vaccines.

I: what should be done to improve this?

R: It is better to inform the pharmacist correctly. This can be done by [head] or another responsible person. He has to provide us with all the commodities that we use monthly. We don't have to beg for it while we are working. We were being considered as if we are working for our business. I need to have all the supplies to do all the activities I am responsible for. I don't have to beg for DEPO and courtem every time. I have to receive test kits for daily consumption to be evaluated for not doing 21 tests daily. Last time people from [project] asked me about this. I told them about the problem from both the health center and our side. We don't have to beg all the time to get the supplies. Such thing makes you not to do what you are supposed to do and it also affects your motivation. So, he has to be well informed. If it is me I will tell him to make HPMRR filled monthly and arrange the schedule. He has to stop saying I am off. For instance, if the date is 20 and he is on duty last night, he can give us the supplies when we come for reporting and can break another day. I believe this problem can be solved if we get the supplies when we come to submit

the report. Secondly, the problem from our side such as failing to fill HPMRR and requesting supplies before stockout should be addressed.

I: you are raising very important thing, aligning it with HMIS report is good thing. But don't you think you will have overloaded? Aren't what you report too many?

R: yes, it is too many.

I: yes, you report many things. When we take HPMRR, it is about one page. How do you see the quality of the data? I accept your suggestion; my fear is about the challenge with reporting too many things at once.

R: it might be compromised, but what I am thinking is not to come another day to receive the supplies.

I: are other HEW also help you with preparing HMIS report?

R: Yes.

I: What about HPMRR? Do they also know how to fill it?

R: they may not understand it. Now I am forgetting how to do it. It is in English but you can understand it if you read it. We support each other while compiling the report. It takes us about three days to compile and it will be more than that when it is quarter report.

I: are you using the bin card?

R: I am not using it but it is available. My colleagues took orientation about it when I was on leave. but they are not able to inform me how to use it. I asked them to show me but it was not clear to them too. We are not using it.

I: what about HPMRR form? Is it being available now?

R: No, it is not available.

I: What alternative transportation methods could be deployed to enhance commodities delivery from health center and health post?

: For [health post], it will be very important if our health post is included under Seka Health Center. This will save our time and money. [health facility] is next to the bus station and passes next to the that health center when we go to our health post. This is the best option that has no alternative. Secondly, having a schedule here (at the health center) whether on the 20th or 30 and not make us, again and again, ask for the community.

I: What support does the health center provide to health posts to strengthen data use?

R: we never received any support or feedback.

I: do you have a shelf in your store? How many rooms does the health post have?

R: three rooms. One is used for delivery, the other one is where ANC follow-up is done, and third one is where we provide customer service.

I: **where do you store the drugs?**

R: in the third room where we service to the customers.

I: Do you have refrigerator?

R: yes, we have.

I: Is Oxytocin available?

R: no

I: Are the existing platforms such as PMT available at HP?

R: Yes, I am secretariat

I: can you tell us about it?

R: [project] has given us training on it. We meet and discuss with Kebele representatives. If you ask me about its regularity, it is not regular. This happens due to workload. Another problem is people who engage in the meeting always expect some payment for every activity related to health. Whenever you call them for a meeting, they ask for money. Due to this the meeting is not regular.

I: when was the last time you had a meeting?

R: it was about three months ago.

I: What were the main agenda of your discussion?

R: Our discussion was during the first month of the fourth quarter. We discuss on health performance of the health post. [region] Development Association has been supporting us by providing training on how to discuss with the community about the performance of the health post. Discussion was also focus on the use of ambulances. We also discuss the quality of service provided at this PHCU. This team also have meeting with a community from Gare (small community structure) every three months. During this meeting we give them different cards with color, then we ask them how satisfied with a specific service and raise the card. For example, if they are very satisfied with a given service, they show up the green card. We may ask them how satisfied they are with ambulance service and they may raise the red card. We then calculate the number of red and green colors and make a report. So, every three months we conduct it at Gare level, the one with eight member is conducted every month and discusses health, education, and other issues.

I: what about discussing with health center? Have you discussed about the issues you have told us earlier?

R: we bring the points raised at health post level as report and discuss it here. The committee includes agriculture, teachers and religious leader. Those committee members will come and check at health center whether the points raised on the health center is correct or not. For instance, community may give a red card on certain service, then the committee have to come and check whether that problem really exists or not in health center. However, we never come and check it this year. No visit was carried out.

I: What support does the health center provide to health posts to strengthen data use?

R: There is no such support.

I: What is the main reason you haven't used the bin card?

R: The reason that I haven't used it yet is, I don't know how to use it.

I: what should be done to overcome the problem of unavailability of the supplies at lower a level? What support should be given, including capacity building and other support?

R: Training is important. Someone may forget how to do things over time. We took many trainings; I am not saying we were not trained. We may forget things over time. If there is training in that area it is important. Monitoring is also very important. If the body that provided training is not follow the status of the activity, ask for the progress, ask for areas that is difficult to do, and appreciate for what is done better. Most of the time we take the training, get back to home and it is over. So, training is needed and monitoring of the implementation of the training is important. Supervision is very important. No one ever come to our health post for supervision. No one came and say you are doing this right or wrong. The problem is our health post is not on the roadside. But we feel bad when we see kebeles which were not better than ours is being visited several times. We can work better if there is someone who come and support us.

I: **Thank you so much for sharing with us valuable information. We will work on the information you provided us and come with solution in the future.**

End of interview

Transcript (4)

**Name of Project:**	[project]
**ID Code**	KII-OA -04
**Type of interview**	KII
**Type of informant**	[sex]
**Region**	[region]
**Role of the Interviewee**	[coordinator]
**Years of experience**	8
**Name of Transcriber:**	[lecturer]
**Date of Transcription**	August 2024

Length of interview	Start time: **2: 06 PM** End time: **4: 01 PM**
**Names of Reviewers:**	

Start of interview

I: Can you describe your role and responsibilities within the [ORGANIZATION]?

R1: My name is (participant name) and I am the [coordinator] at [organization]. I have 8 years of work experience. We can discuss my experience during these eight years.
R2: My name is (participant name), I am a delegate [store manager]. I have more than 8 years of work experience.

I: Our main discussion point is on supply n management. How do you perceive the overall effectiveness of the supply n management system?

R: [ORGANIZATION] has been working with the support of many partners. Although there are no clear research findings available, I think [ORGANIZATION] has been successful in more than 90 % of its activities. We deliver the products to the facility level. During this we face different challenges and also have the technique to overcome those challenges. There are things that prevent us from going far when there are seasonal variations. It can be the capacity of the car. It can be the service year of the car. With this challenge, we are reaching the facilities directly that are supposed to be reached through direct delivery and others through indirect delivery. Our future plan is to make 100% direct delivery. Our current direct delivery is around 82%. The remaining is provided through woreda. For example, a vaccine is directly reaching 137 facilities. Since the vaccine needs care, we are reaching these number of facilities. We can also deliver to other facilities like the vaccine, but how ready are the facilities? Availability of the storage place prevents us a little bit. Some partners are providing solar fridges. It is possible to install a solar fridge but it requires a budget. If there is a partner who supports the fridge, the vaccine can reach the facility level without losing its potency. We improved this 82% from 72%. Moreover, the number of health centers is increasing. We are servicing a total of 337 health facilities; 27 hospitals and 310 health centers. Two new hospitals have started working, and health posts are being upgraded to health centers.

We are working with one organization on the number of facilities we are going to reach in 2017. There is a problem with the cars. The age of the vehicle is too old. The road infrastructure is not good enough in rural areas. We face big challenges during the rainy season. Other than this, we are successfully delivering the product to the facilities. We cannot do anything when there is stockout as a nation. There is no product which has reached here and we didn't deliver to the facilities. Further data can be found in HMIS data which was implemented by . You can find out for which facility the product is delivered, and which facility requested didn't get the product. There might be some complaints as everything cannot be smooth. There is a data quality problem. For example, when RRF is filled, there might be quality of the filled number, timeliness is checked. Completeness, its visibility and all 5 components should be checked. If they fill 00 where it is not supposed to be filled like that, we will face problem to send the products. Although they request for 500 items, we cannot send 500 of the items. We do ration. We cannot send what they have requested. The first reason is, it will be abused and increase wastage. It can expire if they take over. There is a responsible body which provides training on this. Even [ORGANIZATION] has been providing training. [ORGANIZATION] has been providing IPLS training. The role was then given to the Ministry of health. The Ministry of health provided the training at some place and stopped it. Now, one reason for poor data quality is due to high staff turnover. They are doing what they never get trained on. Newly recruited staff need someone who can aware them how to use the IPLS. So far, IPLS stands strong and is making sure work is done properly. As it provides direct training related to what is supposed to be done. Newly recruited health workers should get enough information about the service they provide. I have worked here for a long period of time but there might be some activity that I do not know about. There is a big problem is on ware house management of the facility due to turnover. The other problem is communication. Woreda heads, facility heads and health workers at lower-levels may leave the facility due to different cases. Unless those who leave that position can properly tell they newly assigned person, we will face big problem. When we try to reach out, we will be informed as that person has already left that position. The newly assigned person says I don't have information about it. This kind of communication creates problems. There is also a problem with reporting. Once they get a service, there is a trend of bypassing. While it is easy to discuss with [ORGANIZATION] and solve any problem, they call the ministry of health and political wing. When things that can be solved at (organization] level is bypassed and discussed with a person, things get complicated. It will demo

(demoralize?) someone who is working well. In such situations the community is affected. To overcome such things, communication is a very important thing. The reason why we are always in rush and have tough activity is that we don’t have information about the products. Let me tell you what happened yesterday. The product is available here. We will keep it if we are provided with extra products. Most of on time, reports or feedback comes indirectly. We heard there is cholera outbreak. The RDT was transported yesterday. We can do better if the information is disseminated by triangulating it with a responsible body. To do this, how connected are zones with woredas, woreda with health centers, and health centers with health posts is very important. If the information is provided by keeping its n the product will be delivered properly. So, if we work hard on this area, supply n can be easily delivered. The [ORGANIZATION] branch which is called “[organization]” is not 100%. They are not better than us since they deliver up to [district] by passing through Gurage. The topography is the same and it cannot be 100%. We go to [district] passing through [zone] region and deliver the products. It would be better if the partners could work on this.

I: Can you make the route clearer?

R: Which one?

I: The one you explained lastly.

R: If you [district), it is 150 Kilometer far from here. There is a place called “(facility]”. It is 17 Kilometers far from [district]. Next to that, there is a new woreda called [district]. This [district]is 37 kilometers far from [supplying hub]. But from here it is 150 plus 76 kilometers far.

I: Now it is clear

I: What about facility readiness in terms of reporting? Anything about emergency situations.

R: it is similar to what I was saying earlier. Previously they used the hard copy provided by . We are giving them the soft copy now. There is also [eLMIS]. Emergency orders happen not only due

to our problem. When they bring poor quality data, we may give it as a ration. In between, those products will be out of stock and this will lead to an emergency. The facility has no readness on reporting quality data. If there is no new added patient, what product is used in a month is known as TB, HIV and family planning. unless there is a new user, if the Depo provera is for a woman in July, that women will come back at the end of September. So, for that woman, it is requested for the September. There are also those who use monthly. For these people, it will be requested to consider monthly consumption considering new users. Once you know the presence of an additional 1 or 2 person, you request the supplies included for them. The big question is who will assess and do this? Most of the time only pharmacists fill the RRF. We recently visited the facility for direct delivery. We asked for the form that each ward requested, but it is not available. If it is not available, how can it be functional? Unless the ward knows what, it has used, how can they manage it? If you check the filled form, everything is zero, but last part is filled. The IPLS used to show how this thing is done. In the future, if the partners or the ministry of health provide IPLS training to newly employed workers, supply n management will be better. Then the wastage will be reduced, stockout will be reduced. Emergency will also be reduced. He knows what he used, what he has at hand and he knows what he has to request. This will reduce [ORGANIZATION] burden and poor reporting rate there. ((*phone ringing*). This happens when IPLS training is provided appropriately. Other than training, joint supportive supervision should be provided. Still, it has no importance unless supportive supervision is provided. There is an organization called . They have a group that check the store status and give feedback. These people have provided IPLS training. There is a high turnover among those who get the training. There is a condition in which they never get one person after training all of the staff. It is better if the partners create a means by which the staff become stable and stay longer in the facility. Previously, there was DTC. These DTC committees determine the right product they need and collect reports from wards and request the purchase. It also has its own training. The drug and therapeutic committee established by including every department as member. Even the finance unit is a member. Only those who participated in supportive supervision can know its status now. When you ask them whether |DTC is present or not. They say yes, when asked to show you the evidence, they may or may not show you. It is possible to identify someone who is trained or not trained on IPLS. There is data of those who get training that is provided by . There is also which started working about 5 or 6 months ago. The project is available here. They are working on route optimization. It is identifying which

way of delivering products can minimize cost and time.

I: Who is doing that?

R: provided training for some individuals.

I: Is there anything you can say about workforce capacity?

R: there are health center with only one pharmacy professional. We categorized the health centers as high, medium and low based on the patient's daily flow. So, only one pharmacist may be present in the one we call high. A nurse may take responsibility for the store in an additional role. So, he may not have enough time to show each and everything that is supposed to be filled. As everyone has their own profession that we can be effective, it is difficult. Unless I show the nurse how the RRF is filled, I cannot say it is your mandate to do this. But if he gets the training, he can do that report. But it is not possible to make them this without making him aware about it. Additionally, … we expect there is a skill gap among the professionals. Recently we went to Bonga to give IPLS training, through . There is one professional who is employed a month ago. He has to be trained. How ever, there are individual who served 12, 10 or 6 years and who come to be trained for the 3rd time. how can we identify such thing. I am not saying awareness is not important. I am not saying why he was taking the training. For whom should the priority be the main question? If there is only one pharmacist in a certain health center and the facility, I requested to send one person for IPLS training, that individual come, we cannot do anything. If the person working on the warehouse come and get training and the one who work on dispensary come another time, they support each other. Most of the time [ORGANIZATION] is not engaged in many activities of the partner. Supply n is a team work and with whom that team will be organized? Malaria is now prevalent. They were in the field for 20 days and identified the gap. Who is responsible for following the distributed products? (*phone ringing in the background*). The main thing is not only distributing the supply from here. I am responsible for distributing the products, checking arrival, and receiving model 19. He has to bring and submit the model. Otherwise, he hasn't delivered the products. So, I asked the person who took the products to the facility. It is not only filling and providing the model 19. There should be a responsible body which follows the proper utilization

of that product. We need to do a follow up at least every quarter in a team. So, there is no partner who gave us feedback. In general, such things will lead to self-protection. For instance, I receive the filled model 19, but the product is not reaching there. So, can I be accountable for do this? Who is responsible for this? we are happy if we are asked “We are skeptic about the product you have sent last time. can we check it to gather?”

I**: Can you describe the current quantification and procurement process for RMNCH commodities, including vaccines?**

R: Most of the time it is at the reporting level. [ORGANIZATION] can only request based on the report it gets from the facilities. I am not bringing it without it. Even for RDF: Revolving drug funds. Not only the program drugs, which is provided by donation, but purchased drugs are only requested when hospitals and health centers ask for them. I asked them once when we met in the review meeting. If you are not requesting it properly, it will be difficult to forecast to a higher level. You have to explain the amount you need. For this, it is not only the pharmacy profession responsibility, every health professional should be involved. It should not depend only on the consumption. Morbidity should be known. It is also important to understand what problems can happen as an epidemic. We asked them if they have this data. But the trend is, they give the consumption they need for this year, that is all. This is why we become distributed when we hear about the occurrence of the epidemic in certain areas. That is why we need to estimates the occurrence of certain diseases at certain times. The person who treats the morbidity is responsible for doing this. we hope there are health professionals who can say the prevalence of this disease may be higher at this time. sometimes, we here as there is family planning shortage in some areas. There is a gap when the one they received from us and their survey is compared. No one can give you an answer when you ask for the difference between the amount you provided and the service they gave. (*drop his pen on the table*). Special it is associated with malaria drugs. We heard theft has happened in a southern region. The product we provided was found in the private pharmacy when assessed for the complain about the theft. But no action was taken on the thing. Whether it is at woreda level, hospital level or health center level, the regulatory status should be checked. Regulatory should ask for everything.

I: How about the forecasting, data quality and emergency request of family planning,

maternal and child health commodities?

R: we have raised about this earlier. Data quality will create an emergency request. This is due to unawareness of our reporting system. For instance, if 100 mothers are using the service they provide, they do not specify which product they use, whether it is a Depo or long acting. They just request for 100 products without specifying them. At this time, we send the products based on their last year consumption. Requesting without considering the will create problems in the forecasting. Although I am not working on the forecasting, average monthly consumption should be checked. This average monthly consumption will give direction for what we have to request. It has a formula just like RRF. For the months of August and September, it should be requested in July. For October and November, it should be requested in September. Is it every other month? We you provide for this month you keep for the next month. Our distribution is also classified as odd and even. Odd and even means, for instance, July is odd, August is even, September is odd, October is even. In such a way they will receive six times in a year. Those who requested it this month will receive it in August and those who requested it in August will receive it in September. For that we request from them a higher level based on for the average monthly consumption. There can be shortage from our side. It is not due to failing to do procurement. This may be due to a delay at the port. As we are land locked, customs clearance in our country is a little bit difficult. If they family planning product is allowed for a week, we expect pregnancy to happen during that week. We can reduce this if the correct data is provided. This is what our forecasting the higher-level looks like.

I: How does the transportation and distribution system for RMNCH commodities, including vaccines, function across different settings? Including routine optimization.

R: our routine optimization is done based on the topography. We have about 9 zones. If you go to the west there are [zone 1] and [zone 2). [zone 3] is obviously in the center. {zone 4], [zone 5], [zone 6] and [zone 7] are in the south. Our route optimization is done considering which route is best to reach the facilities. We do it in a way that prevents repetition of the route. Based on this, there are woredas where all the facilities are being reached directly. There are places which are totally impossible to reach directly. We have data for this. For those facilities, Woreda will take it and use ambulances as an option. We also have “long base” cars that can be used to deliver in such

areas. We classified it as; all type of vehicle, {NAME OF A VEHICLE] only, long base only.

I: You mean the places?

R: yes, the places. So if we send {NAME OF A VEHICLE] to a place that is identified for long base, it is on us. However, for places that were considered for {NAME OF A VEHICLE] may not be possible during the rainy season. If you know [district] which is found in southern region, there is [district] which is 15 kilometers from [district]. There is also a place called [district]. To reach [district] from [district], it is very challenging as it has been raining for all 12 months. In such places, the road will accidentally close due to land slide. In such a situation, we will communicate with zones and arrange for the ambulances to pick it up. Last time, people from [partners] were here. We asked them for cars. As the age of vehicles increase, fuel consumption increases and its capacity decreases. Its force decreases while its fuel consumption increases. There has been also cars shortage when there have been epidemics and campaigns. When there is pandemic like COVID 19, cars shortage will happen. As an option, we ask the zones and woredas to come and collect it using the car they have. No zone or woreda refused to collaborate during any campaign. We deliver to facilities that our cars are able to reach and they will come and take for the remote facilities. For a car, it is not reaching the facility, it needs maintenance when it returns. That means they are old. This time, only long base is a new model, but you cannot reach all the places with that car. You have to consider the volume when you assign a long base to a facility which is 300 kilometers far from here. It may drop to facilities in between when it travels 300 kilometers, but it takes a total of three days when it returns. It may also take up to four days if they face problems on the road. These are the main challenges for the supply . Transportation is a corner stone for the supply . Road infrastructure is another challenge when there is seasonal variation. Any car can reach the place where the road is constructed.

I: What about the transportation from health center to health posts?

R: We deliver only up to the health center level. Health posts receive it from health center. You are talking about an important issue. Product stockout, overstock request and things leading to emergency requests might happen due to health posts failing to report to the health centers. If they

are not filling the IFRR and reporting it to the health center, what data can the health center use to provide the products? As we request for the consumption from woreda, the health center should fill the RRF and request from woreda after receiving the IRFF, I mean HPMRR.

I: Why are they not filling? What is the reason behind this?

R: this can be answered by the health center and woreda. We don't know, but it might be negligence. There might be a transport problem as they are supposed to go there in person. We are not sure about this; the health center and the catchment area know this. HPMRR must monthly arrive at the health center. If they reported it to the health center and the health center forget to report, it is a health center problem. It is not health posts problem. If the post is not reporting it, the problem is theirs. I was with people who came from [regional town] before the establishment of the region and visited them. They are doing well in some places and while it is not good in others. We also found someone who says "they have no place to keep their registry" as they are using the kebele as their working place. this can be another big problem.

I: What about back-order management?
R: Pack?

I: back, back-order?

R: Back log, okay.

I: how about it?

R: Back-log is done if and only if we are stockout. As I said earlier, this can happen if the custom clearing is not done on time and the product is not coming. For example, we provided it last week and it came after it were done. We checked the RRF again and sent it as a back log. Depo Provera and Implanon was stockout for about 2 or 3 months recently. We informed them to use it by borrowing from the hospital or any facility, and we delivered it when it arrived here. The question must not be whether we have delivered the back log or not. It must be whether that mother who

missed it a month ago has returned and got the depo injection. Was the product properly used? Is there any responsible body who monitored it to be used for the next when there is a shortage? That drug should keep us in reserve. How can this back log be managed? We will deliver the back logs for the next 2 months based on the data they sent to us. How can the past 15 days be managed?

I: You are stating very important points that we can also check when we go there. To identify areas that this project can support. If it is needed to scale up direct delivery, what is the capacity of [ORGANIZATION], [resupplying hub]? What do you do if their back order is too high in terms of manpower, vehicle and budget? Is there any means of third-party involvement? Including lead time? what is peculiar in the areas of direct and indirect delivery?

R: [resupplying hub] distributes for 82% as direct delivery and the remaining is indirect. This is what we officially report. (*Long pause*)

I: ya.

R: it is better if training is provided not only for [ORGANIZATION], but also for hospitals, health centers and woredas. They have to be aware. The other thing is … has provided two cars to Hawassa hub and they have easily increased the facility they reach. If we get a car which has a capacity, we can reach facilities that are challenging to reach.

I: So, you need a vehicle for direct delivery. At this time, you have {NAME OF A VEHICLE] and long base.

R: there is a place where all cars can reach.

I: what types of cars do you have?

R: We have FSR, {NAME OF A VEHICLE] and a long base. It is a van. I hope you travelled in this area. Starting from here to [district] and (district], there are a lot of facilities from left and

right. Any car can reach these areas. There is no problem with reaching 2 or 3 kilometers from the main road. We will send a bigger car. But, when we enter more from the main road, for instance areas like [district] and [district], it is also difficult. There are very challenging woredas in [zone]. For these areas, we use {NAME OF A VEHICLE] up to it is able to reach and use long base for the remaining. For example, {NAME OF A VEHICLE] can enter areas like [district] and [district]. However, we are sending the long base due to security issues. The road might be good, but you have to think of choosing between {NAME OF A VEHICLE] and Long base to run fast and escape at the time of security problem. Van can also go after confirming as there is no security problem. If your project the fund, first, provides training to everything related to supply . Second vehicle. The training will overcome problems with data quality and communication. The vehicle can improve our capacity to deliver the products. Another important thing is working on route optimization. Kaizen institution is also trying to do route optimization. This is also the one done by . So, these things can be merged and choosing the best one after comparing it. Or it can be integrated. To complete this integration, route optimization can be done in a better way. We do not use out sourcing. We never used out sourcing. I remember we used it once when it was out of management. It is difficult to out-source at branch level. You have to make a bid … it takes time. what we use as out-source is, for instance if tracker come to distribute bed nets, we make that car to distribute those products to the facilities on the road side when it returns back. Outsourcing is the mandate of head office. We cannot talk about the transportation problem if we have that mandate. There is no place that merchant's {NAME OF A VEHICLE] cannot reach. This is our van, there might be a balance problem. The other thing is, although it is not related to [ORGANIZATION], it is better if there is a means of reducing attrition rate for warehouse management. It is better if the skill transfer is done well for warehouse management. There are recording materials that should be available. Hospitals and health centers have to get softcopy of RRF and HPMRR. There should be professional integration. The technical working group must be strengthened. If you are working on that as your main activity, it should report to you. They can communicate with you through a telegram group or email and discuss the gaps, then the support can be provided on those gaps. Nothing can be achieved at once. It may start with the provision of training. Then continue with other activities. The order of activity is determined by the project owner. For instance, if you give the softcopy of RRF and IFRR, I can distribute it. Through this our communication becomes strong. When you visit me the region, zone, woreda or facility, it is

better if you ask them about the warehouse, what challenge they face, whether they register or not, whether they are reporting or not. When we stopped using hardcopy and started using the softcopy, we distributed the hardcopies to all the facilities in route after making them sign on model 19. We make them sign on the model 19 to make them feel they are responsible for it. [district], which is next to [district], is 82 kilometers from [zone}, but only a long base can reach there. What can you do in such a situation? There are also a places which is 10 kilometers away from woreda but not possible to reach. When you go to {district}, it is a place called {district], it is 2 kilometers far from a road but manpower is used to deliver the products from road side. A solution should be found for such a situation. You cannot change the topography. The health center was built there considering population density and other things. There is a health center which is called [district] in [district], we travelled on foot for an hour to reach there recently. People are living there. There is a school next to the health center. It is very difficult to deliver to this facility directly.

I: is it in the direct delivery?

R: No, the woreda delivery it.

I: what do they use to transport it.

R: they use horse or man power to carry it. It needs to go to the place to solve such problem.

I: let me ask you numbers? What is the lead time? after how long do you deliver after they request it?

R: RRF reach from 1 to 10. It means at the beginning of the month. We do the analysis of presence of the report, completeness of the report from 10 to 15, (*phone ringing from background*), products will be read from 16 to 20. There might be a situation in which we may request as emergency in a situation like back-log you have said earlier. Here is what we call a pick list, it is done from 15 to 20. From 20 to 30, it should be delivered to reach both direct and indirect facilities. It should reach the facilities that are direct and woreda for those which are indirect. There is also tolerance time. It is time until the next RRF arrives. But it is not recommended most of the time. but you can

tolerate up to 10. There is also the time when we can extend an analysis time for distribution until 25 following the reporting period if the product is not reaching us. This is tolerable until the next RRF is submitted. It is tolerable but not recommended. If you need for vaccine, it has its own directions which we call even and odd. For odds, it reaches here from 25 to 30 in the reporting period and should reach the facility from 1 to 5 following the reporting period. For the Even, the VRF reaches here from 1 to 5. We are responsible for delivering it from 6 to 11. Since the zone is the nearest, we agreed on it arriving here from 6 to 11. VRF arrives here from 6 to 11. We need to deliver it from 10 to 15. For this, we have an agreement.

I: It is every month for vaccines?

R: yes, it is every month for vaccines. It is every other month for others.

I: Is there any situation where you have checked the forecasting accuracy?

R: forecasting accuracy can be around 60% or 70%. As I told you earlier, they are sending it only based on consumption without considering prevalence or morbidity.

I: What are the main implementation challenges of IPLS in health facilities?

R: First, there is staff turnover. Secondly, there is transfer from facility to facility as a promotion. When the person who has been working well leaves the facility, the activities decrease. As there is no supportive supervision on its implementation, there is some sort of forgetting it and negligence. No one is checking the status of the store following the training. No one checks how the RRF was being filled. There might be no skill transfer activity. In order to keep the implementation of IPLS actively continue, needs to provide supportive supervision and feedback, making the skill transfer when the responsible person leaves the facility. If there are health professionals who are not trained on IPLS, the training has to be facilitated..

I: What about the implementation of eLMIS?

R: So far, there are some hospitals which are reporting using [eLMIS]. it is being reported using a hard copy. Other than this, telegram and email can be used. For instance, as the vaccine is reported monthly, they fill the format we gave them and send it to us using telegram. We will print it out and use it. Hospitals send using email when the [eLMIS] is not functioning. RRF formats are preprinted. To improve the implementation of RRF, it needs to give supportive supervision on the appropriate handling of the documents. We need to check if the IFRR is filled properly. It needs to check the status of warehouse management following the training.
I: what are the challenges of implementing electronic LMIS? You told us some hospitals are using [eLMIS]. I also have information about the one that is implemented by .

R: It is [Name of eLMIS 2).

I: Yes, I heard about, is there any situation in which such things can be implemented in these zones.

R: What we use here is [eLMIS 2]. We asked them about the importance of this (eLMIS 2). I don't know more about the . They told me it helps to on how to manage the store. They told me as it is filled on computer and mobile. There is no report coming to us from it. But they are planning to implement it just like [eLMIS 1]. I don't think it is possible to share it via email. But it was possible via the [eLMIS at (RESUPPLYING HUB)]. It might be difficult as this one is filled using codes.

I: Do you know the number of facilities that are using [eLMIS]?

R: Minimum of 10 hospitals. Big hospitals like [x, y, z] have the access.

I: what about the softcopy?

R: There are about 10. Many of them use telegram.

I: you mean the fill the excel and send it using telegram?

R: they fill on the excel and send then we can download and use it.

I: how many of them are using it

R: The majority of them use it. We can say all of them use it for vaccines. There are 8 items of vaccine and they can fill it on their phone. [eLMIS] is being recommended to change it to electronics.

I: How effective is the inventory management system in woredas, health facilities and health posts?

R: this can be difficult to say anything. I cannot even say anything about the health center next to my house. We can generate our inventory management data. That is why we were talking about the role of technical working groups during supportive supervision. They have to ask about the stockout, for how long they are being overstocked and then report to the project or responsible body. The committee should ask about the status of the stock during supportive supervision. They must ask for the data about the stock. I told you about this at the beginning of our discussion. They said MVA is not available while it is available. You can get more responses for the hospitals and health centers.

I: I understand, in case you observed overstock or expired products. As it also shows us their inventory management. Is there wastage rate?

R: This is another person's mandate. I don’t have the right to ask about that during my visit.

I: only from what you observe.

R: there is a problem in its management. You may find vaccines out of the fridge. It is difficult for me to say I saw overstock. For [ORGANIZATION], we haven’t done supportive supervision this year. I visited the facilities with partners on how to improve family planning utilization.

I: How is supply n data generated, shared, and utilized for decision-making within health

administrative units and facilities? How is performance of DTC and PMT? The role of pharmacist in the team.

R: there are health centers which are functioning without pharmacists. It will be difficult to ask them this in this place. Shortage of manpower mostly existing. Problem we had been stating earlier; emergency order and stockout are due manpower. We will work on this in the future. Until then we will communicate with the facilities. Our forecasting group call to facility and discuss on the data they have sent. We communicate with hospitals more when there is regimen change. For instance, previously the bottle held 30, now it is 90. We ask them how they calculate now. This takes us to others mandate and difficult to give answers.

I: In case there is a platform where you meet with zones, woredas and facilities including analyzing the data. You also told us you do RRF analysis and provide feedback. What achievements are there and what should be improved?

R: the question should be with whom our communication should be. The [ORGANIZATION] communicate directly with hospitals and health centers. It also has communication with woredas and zones. The woredas know the population. They get this data from health centers and health posts. Our communication with woreda is; about available products, non-available products, informing them to collect new products. For instance, there are three hospitals which provided MDR TB service. We will call and inform them to collect the products. Your communication with others is mostly through RRF. However, the data is provided by the woreda. The woredas send it zones, and zones send it to us. During the recent outbreak, the woredas requested zones, and zones requested us. Regions can also communicate with us even though their communication is with the main branch. They sent us the number of tests done and requested for the number of RDTs they need. We are not providing it unless in they bring the DHIS2 data. We gave good feedback on this to (region). They allowed us to see it. We cannot edit it. They gave it as access to it. I can open it and see it. I cannot edit it. But read it like a hard copy. This is a very interesting thing. We also thank them for trusting us. If electricity is not available and the miss to send it, we believe what they tell us.

I: we can take this as best practice.

R: using DHIS2?

I: is there any best practices in the supply n that could be scaled or replicated?

R: It is difficult to control everything at once. For example, I have a google sheet. I am the owner of that google sheet. The dispatcher fills it when he dispatches the product. He fills out the SPV number, including the products, who took it, the date it out and everything. Only the people I gave the mandate to edit it. Manager, WIM and others are just viewing it. The manager can see the number of if the facilities that are serviced today before me. Warehouse manager can also ask the STV is 50, but only 40 is loaded, what about the remaining 10 STV? It will help us to follow that number. We have for dispatchers. The person who provided the RDF tells me the number. It will show me the number of facilities that received the vaccine. It shows me the number of health program that is loaded and the number of printed STVs. It gives us the information for on the accountability. It is better if the google sheet is developed and used. Or any other software can be used. If your project uses such a thing during your supportive supervision or any activity and fill it on that software. I can see the status. Whether the product reaches the community or not is the responsibility of the regulatory body working there. I used the google sheet to register the model number of a STV. If he does not bring the model, it will be empty. Someone who delivered that product has the right to request the receipt. We will ask that person for that receipt. We don’t care whether products are provided as donations or purchased. There is software that can be used to allow someone to only view and others can edit or comment on it. using such thing can in increase the scope of the communication. Some individuals may not check that google sheet. Alarms should be used in such situations. By the way, everybody can check telegram when there is a new message. I can check what every message you can send to me. As it is time to use electronics, using it can improve our communication. For route optimization, you can go to the place and judge it. we have to be selective when we observe it. Traveling from here to [region] is a good road. It should not be like that. You have to randomly select one facility which is off the road and check it. this is important as evidence which helps for route optimization and vehicle utilization.

I: our main goal of our project and your system is ensuring and making the right product reach the right person. In addition to what you have stated earlier, is there anything you can add in terms of reducing stockout and minimizing wastage?

R: We have discussed IPLS repeatedly. It has six rights. Isn't it? it includes the right time, place and others. The seventh one is the right customer. If we strengthen what we have been discussing so far and prepare some kind of SOPs is a good thing. For instance, IPLS has no SOP. Depending on the IPLS, the project can develop and distribute separate SOPs for health center, hospitals, woreda and zone and then monitor them, which will be improved. Many things are being printed and distributed by the ministry of health. Your project can also do this. You can publish best practice as magazine and distribute them. we can do better by strengthening the existing practices. For instance, we had been teaching about IPLS at health center starting with what they have to do first. IPLS is a big book. If the operating procedure is prepared in a way that shows what they have to do when the product arrives and how the RRF is filled. It obviously requires a budget. If we publish a magazine that show how they reach one health post with challenging road infrastructure, the other facility will see it and say we can also cross the area which is difficult during the rainy season. You can make it colorful as long as you are investing in it. this will make the project popular and shows how the project is working on the challenges. I am happy if this is done. I think we can do better if these things are considered. [ORGANIZATION] is always participating in everything. If we work together and strengthen our communication, we can reach 100%, reduce stockout, reduce wastage, reduce over stock. Nowadays, we are holding information in our pocket. It only you who knows you are using the GPS or not. But your phone may be providing your location

I: Thank you for the information. End of interview

Transcript 5

**Name of Project:**	[project]
**ID Code**	KII_OA-05
**Type of interview**	KII
**Type of informant**	[sex]
**Region**	[region]
**Role of the Interviewee**	[health Facility staff]
**Years of experience**	13

**Name of Transcriber:**	[lecturer]
**Date of Transcription**	August 2024
**Length of interview**	Start time: **09: 03 AM** End time: **10: 34 AM**
**Names of Reviewers:**	

Start of interview

I: Can you describe your role and responsibilities in [health facility]?

I: I am facility staff. My role is to give direction, evaluate and monitor activities in the health center. Discussing with management body, we provide support to units that need it. we also mentor individuals who are in need. I am a leader of the departments or teams in the health facility. I help them have meetings based on the preset schedule. I will work with the management to overcome the problems identified during the meeting. I also monitor the health facility and health posts performance monthly. Three health posts are under our health center and we monitor their performance and provide support.

I: Are three of them basic?

R: eee, yes, they are basic.

I: Tell us about your work experience?

R: I have worked for about 13 years

I: How do you perceive the overall effectiveness of the supply n management system?

R: I see this in two ways. Many organizations ask us about the problem related to supply . No solution is coming. Supply is very crucial to a health center. We provide health services and medication to the community. We got the drugs from [RESUPPLYING HUB]. I can mention some points about [RESUPPLYING HUB] indicating what was previously happened and the what is happening now. We fill and send RRF every two months to woreda, the woreda will be sent it to

them. Through this system, our commodities will be refilled. There are two types here. Program drugs are refilled. The other is, the one which is called RDF and we buy it. these days, there is big problem with availing RDF drugs. We are not getting it easily. There is problem from the system itself. This means we are not reading each other. There are programs that are being implemented by other organizations. But the problem is still not solved. We are facing big challenge in terms of getting the commodities. Supply was a very big problem in my previous facility. You may ask for 80% but you get only 20% or 10% of it. you can get the products from external suppliers. However, the health centers cannot afford that price.

I: How do you see readiness in reporting and data quality?

R: Reporting system is using RRF. It is filled every two months. He will select and fill in the items he needs. There are two types of form. The blank and the one with the list of the items. We use the blank one when the drug we need is not on the list. For internal purposes, we can use IFRR. That means we can use it here. Each department fills it and requests the supply the need. I don't think there is quality problem as he has received training many times. Not only him, others are also trained. There is no problem with data quality.

I: What about adaption during emergency situations?

R: Supply is a must during an emergency situation. As long as we are in the malarious area, we are in an epidemic situation now. If you ask me about the supply. It is not good enough. We have communication problems as the health center, woreda and the organization which provide the supplies.

I: What are the major challenges for continuous availability of health commodities at health facilities and health posts? (*Phone ringing*). It might be related to storage or transportation.

R: We buy the products through woreda. The cars are availed by the woreda. Although it is not enough, we have the storage. The size may not be enough, but we have standard store. Due to this it needs expansion. Since the vehicle is provided by woreda, you may or may not get it. such

problem happens regarding transportation. There is a context in which you may not get car when it is out of the area. In my evaluation, the warehouses are not enough. They are small. You can check it later. Although it is built based on standard, it is not enough. The transportation is also not good all the time.

I: Can we say man power is a challenge?

R: yes, only one pharmacist is working in this health center. This means, he is working in two places. He works both in the dispensary and store. Work load exists. The other thing is we assign other health professions when he is off like today.

I: I am happy if you could tell us more about the human resource capacity. Please tell us the staff commitment, training and the number? What do you use as means of staff monitoring and means of motivation?

R: two things bring staff commitment. Human beings can work with commitment when their needs are fulfilled. The situation of the supply is making our staff disabled. We may give recognition every three or six months through performance monitoring. These things are not applied yet. We are planning to undertake it. Yesterday we had a meeting and planned to provide recognition for best performer staff every six months. However, there are many problems associated with this. It is difficult to say enough training is being provided. All staff here came from different facilities. The problem is we don't have national or woreda level database. For instance, we don't have we the data of who gets trained what, when he gets trained. There should be a database that links these together. For instance, when (his Name) comes from [another facility], there should be data indicating the training he has attended and activities that he is able to do should be available. Otherwise, I will ask for the training that I have attended. Now, we have started filling a database which overcomes this problem. We started identifying who gets training on specific areas. Things that initiate the health worker are also needed. there might be ours or woreda's problem. Motivating health workers woreda involvement. If you feel what health workers need, they work better. For instance, the health worker here is not motivated as compared to the facility that I had been working in previously. They raise reasonable points for this. Although there are some

individuals who is not committed, there are health workers who work with commitment. They are not even happy with the job. To overcome this, we are discussing with the staff on what to do and developed an action plan. Health posts are not getting program drugs most of the time when [RESUPPLYING HUB] is not providing them. when there is no drug, the patient will be referred from lower level to here. Shortage of supply has an effect on staff commitment and the community.

I: Do you provide orientation on use of IFRR and HPMRR when new staff are recruited or transferred? It can be for pharmacists or units. Is there an induction or skill transfer activity?

R: Yes, there is such practice. For example, when any staff member return after attending a training, that staff provides orientation for other staff in the facility. We also provide orientation on how to fill IFRR and Bincard for staff in every unit. They need to know the amount of the product they have at hand while requesting it. they have to know their stock. The focal persons are DTC members and there is meeting every month. He also monitors everything in the units every month. Previously the IFRR was not filled in this facility. Every product is distributed without control. I have printed out IFRR and distributed it to everyone. I then informed them that no product can be provided unless the IFRR is filled and he and I sign it. Previously, the doors were left open, they were not locked. There was big problem. To overcome those problems, what we did first was, for instance, we may be on duty during the night and handover the materials in the morning. No one asks you if you take the material in your bag and leave the facility. Many things were being stolen and we buy them again and again. What we did now is to handover the materials and the patients every morning. They have to say to the next staff “I provided this fluid to this patient; you will provide this next”. We have started handover the material. We have developed the format here and are using it. you can check it out after our interview. Each unit cannot use the product without filling the bincard. Now, every department is using IFRR to request drugs. We are making this system work.

I: **How about adhering to the schedule? Are they reporting or requesting according to the schedule?**

R: We have posted in pharmacy department that the units will receive the products from 1 - 5. It indicates starting from this date to this date OPD will receive it. From this date to this date, emergency department will receive; from this date to this date MCH will receive, from this date to this date health post will receive. We want all the units to be requested every 2 weeks. However, it is not possible sometimes. We also evaluate the DTC activity. We will check if they had done the quantification and listed the drugs. PHCU should have its own drug list. We'll check on that one. Eee… and will also. … check whether it identified essential, non-essential and vital drugs for the next season using VEN analysis. As we evaluate this, they can also check which unit they are requesting every 2 weeks. When they request it every 2 weeks, I approve it. I will sign it. So, 1 copy remains there and the other copy is with the person who reported it. We have a system to identify who is requesting every 2 weeks and none. Now we are in an emergency situation and cannot do it. we will also check stockout. There is problem with identifying the stockout. Some may consider having 25% as safe. You need to know at item level. If you need 6 items, when should you ask for them? There are staff who do not know this. Some units that request when it had 2 items that were used for today. If you have 2 items, you should ask for it when you have 1 extra item. We had been discussing on how to improve it. we will see its implementation in the future.

I: What is the reason for gap in adherence? From your understanding, what is the reason for not using IFRR?

R: A lot of things are remaining on data use. There is registration problem. I don’t know why we are afraid of it. there are health workers who do not register what they have done on the sheet that is ready to be used. There is problem with data management. It can be due to workload. It depends how people think about it. For example, if I believe I have to write down what I have done, I can do it. Working on changing what people believe is difficult. They may resist you while knowing how to do it. Some may not do it due to different stressful situations. The trend is also another problem. Getting out of the existing trend will be difficult. There was no trend of filling out the form and requesting it. the trend was shouting at the pharmacist to provide them when they are out of stock as if they are not responsible for what will happen if he is not providing them. To come out of that trend it takes time. This is how I see it.

I: **Can you describe the current quantification and procurement process for RMNCH commodities for RDF schemes?**

R: DTC decides which drugs has to be purchased. DTC committee members are department heads. Each department requests what is needed for their department. Laboratories, MCH, ODP and IPD come with list of the products they need. The DTC committee will check each unit's plan and give priority to what has to be purchased. That prioritization is done considering the drugs which are essential, non-essential and vital. It will be approved for purchasing essential and non-essential drugs. There are also drugs which are purchased and not used. To prevent this, the committee will do an analysis and list down what to purchase and bring it here. Then we approve it. after approval we will send it to responsible body with its price. We do the procurement in this way. When there is stockout, they will collect the "Performa" and bring it here. Management opens that Performa and check each item. We then select based on the items and sign it. the products will be purchased the next day.

I: Is there any challenge that you can tell us? In terms of forecasting and data quality?

R: challenges are always there during procurement. There are repetitions. When I say repetition, IPD may request for the drug needed by the OPD. That is why DTC is important to identify drugs that are only important for specific unit. There are units that do not bring a list of drugs when they are requested. Other than listing, they should post and use the drug they have at hand. They have to show the drug that is available. There is also problem of discussing with store manager. They have to talk to the store manager and check available products in the store before requesting it. Unavailability of all DTC members are also another problem. You cannot collect full information when all of them are not available. There were such problems that we will work on it to overcome it.

I: How do you for procurement?

R: they will request for it based on top 10 diseases. They also use EPSG (*to mean EHRIG*). There

is national guideline called EPSG (*to mean EHRIG*). List of items that a health center is supposed to have to provide the service is available. They bring that list and we purchase based on that.

I: From where do you get the quantity?

R: The quantity is identified by the store manager. He does it from bincard. He identifies the consumption and fills it.

I: what gap have you observed?

R: The gap I observed at store level is, eee… sometimes items available in the store may be forgotten and the procurement is done. In fact, I associate this with workload. When you are overloaded, you may forget something. There was a situation when the product that he was considering as available was purchased.

I: Do you follow the bin card?

R: bin card? I follow it. by the way we have to system. I can see the filled bin card sitting here in addition to the one filled on paper. We use "[another elMIS]". I can see what he has filled on the [another eLMIS} I will check the products that were purchased and reached the store. But it has not started at unit level yet. I can show you on my laptop.

I: Can you check the consumption of 250-gram amoxicillin on the softcopy bincard? Do you know the bin card?

R: I know it. it is available you can see it. If he makes mistake while filling it, we cannot edit without my approval. For example, if mistakenly fill 100 while he is supposed to fill 150, he cannot change it once it is filled. He has to come and tell me, and I will correct it.

I: So, this can solve the problem we were talking about?

R: Yes, it is very important.

I**: How long since you have started using it?**

R: It has been about 8 months or a year since Last mile started it.

I: Good. What about the budget? How much is budgeted for drugs?

R: Around 1.3 million birr is budgeted for purchasing a drug.

I: Do you also use internal revenue for it?

R: internal revenue is collected from the insurance. We use that to purchase the drug.

I: what about the 300, 000 that the region provides?

R: It is for program drug. It is provided twice a year. 380, 000 will be provided every six months. It comes in item. it can be ceftra, amoxa or NS.

I: So, you order it from [RESUPPLYING HUB]?

R: we don’t order. We out will do the quantification. I can show you a copy. We filled out the quantification for 2017. The one we filled out will be sent to them. The woreda sends it. their contact is with the woreda. Based on that, [RESUPPLYING HUB] will distribute it twice a year. They provide the woreda and we go and take it from the woreda. This is the one provided by [region].

I: So, you collect all program drugs from the woreda? Doesn't [RESUPPLYING HUB] directly provide it to you?

R: [RESUPPLYING HUB] delivers it directly. But it takes 5 or 6 months since they provided it.

it is discontinued now. We are getting it from woreda now.

I: So, you fill the RRF and send it to woreda. Woreda will send it to them. Then what car do you use?

R: We use woreda's car.

I: good. Has the store manager been trained on quantification?

R: yes, [RESUPPLYING HUB] gave him on-the-job training. I got the training when I was working in [health facility].

I: Did you get the quantification training?

R: yes, people come from [RESUPPLYING HUB] and have shown us how to do the quantification. It is kind of database. It will show you the price when you select it.

I: how do you see his capacity in terms of quantification?

R: Frankly speaking, he is active among the pharmacists I know. I am happy if he is here and you can see how he filled out the bincard and handled the files. They are clear. It needs self-motivation by the way. If you consider it as work, he can say it is not my responsibility. We are making him work in two places. I have worked as head for about eight years. I have seen different pharmacists. There were many problems. There was problem of taking the ownership of the job. You can easily access the data you need. His data handling is good.

I: How does the transportation and distribution RMNCH commodities, including vaccines, to health posts?

R: there are many challenges regarding transportation. It is difficult to go to [district] during the rainy season. We use motorbikes. But, the health center has no motor. The health extension worker

will come and collect it. only one kebele is nearest. Others are far away. [District] is about an hour away from here, while the other one is about 40 minutes away on foot. It will take around 20 minutes if motor is used.

I: but it is good road.

R: Only during the winter. It is not possible during the rainy season. The health center has no car to distribute it. we use motor. We also don't have a motor. So, they come and collect it.

I: you are getting the products from woreda. [RESUPPLYING HUB] is not directly delivering it?
R: in past five months we have been getting it from woreda. Previously, we sent it to the woreda, woreda will request it, and [RESUPPLYING HUB] will deliver it directly to us.

I: What solution do you suggest to deliver the products to the health post?

R: You can use different mechanisms to make the drugs reach there. Relatively, it is better here. If you go to [district], there are health posts that are 13 to 15 kilometers away from the [health facility]. Four health posts, only one of them is nearest to the road. It is very challenging to get there. They are women and you cannot make them carry the drugs. There are conditions in which we beg the community to carry it for them. Once I discussed with the woreda to modify a motorbike into three wheels and use it as a means of transporting the drugs. They refused it due to budget shortage. Have you seen the motorbike that was used during [a known event]? It has a box on one side. We can modify it and can make it carry up to 10 KG of drugs. I wrote and gave them the proposal in 2006. It was not accepted. We assumed it for two purposes. There were high maternal deaths in the area due to lack of transportation. It was proposed to carry products and people. Its copy is available with me. Think about how to facilitate things. But there is no opportunity to help you run it. the products reach there in man power. She came to take some of the drugs. She came the next day and took some of the drugs. Our infrastructure is known *(laughing*). During my stay here, I visited the health posts only once as we don't have a mother. We use transport motor. Those who ride motor are negligent and you fear the risk. If you use your motor you can take the safe way of riding, especially during the rainy season.

I: how about the cost of the transport?

R: 60 birr to go there.

I: have you considered those motors to deliver the product?

R: we think about it. but, this a drug, it is issued using a model. Who can take the responsibility if it is lost? We need someone who can take the responsibility. Unless the health extension worker is with them, it is difficult to think about sending it.

I: I saw this in Kenya. they deliver ART drugs to community pharmacies in the city. This is one of the model services. But it is not implemented in our country yet. They have license, and already know.

R: this is very good thing if there is responsible body. It is possible.

I: how often [RESUPPLYING HUB] had been providing you with the products?

R: it reaches here every two months.

I: within how many days. For instance, can you send up to 10 days RRF?

R: it reaches here at least within 3 to 5 days. After we send the RRF, they will bring it to use 3 to 5 day of the next month. We will send for June and July reports on July 30. However, it may take 10 to15 days to reach while it is compiled at woreda level. Then it will reach here between 2 to 5th day of the next month when they do direct supply. There is a situation in which it cannot arrive here on time. our problem is always with [RESUPPLYING HUB]. I always comment on [RESUPPLYING HUB] whenever the issue is about the supply. I don't know why the government is finding a solution on [RESUPPLYING HUB]'s issue. Everything needs competition. [RESUPPLYING HUB] would have performed better if there was a competing organization. This

is a situation where you only get 10 items and are out of stock for 30 drugs. You send staff paying transportation and perdiems and it is difficult for facility that depends on the insurance money. You will buy from private company with 2 to 3-fold money, while you are supposed to buy it in fair money from [RESUPPLYING HUB]. What will happen to the community? I have been working for 13 years and the only organization that is not showing improvement is [RESUPPLYING HUB]. I also told [name of official] when she visited [health facility] to celebrate national NTD day in our health center. I told her we have infrastructure and supply problems. For me, [RESUPPLYING HUB] is an organization that cannot be changed. We are in the situation of not solving problem drug availability. We don't have any commodities related to [RESUPPLYING HUB] up to health post level.

I: You don't have it this time?

R: We used everything in our stock. We listed the emergency drugs and they refused to provide it saying we haven't started providing services. They said they are expanding their system. We are not providing family planning services now. The provision was stopped in health posts too.

I: Have you worked in any woreda where [RESUPPLYING HUB] provide it indirectly.

R: No. Direct delivery started on 2015. Program drugs were provided through woreda. You can go and collect it if you have the capacity. However, you prefer not to go there, due to the system. They say bring the car when you go to collect 50 depo and 100 Implanon. You to fill fuel and travel from [health facility] to pick on plastic bag depo.

I: what was challenge when it was provided through woreda?

R: You cannot get it on time. You may not find the store man after it reaches woreda. There were many challenges.

I: in general, what should be done differently to improve transportation and distribution starting from [RESUPPLYING HUB] to woreda, from woreda to Health Center and from

Health center to Health post?

R: [RESUPPLYING HUB] should improve its communication with the facilities, as much as possible. It can be difficult for them to communicate directly. For example, there are only 2 health centers on the road side in Seka woreda. Road access to the other 7 health center is very difficult. It can be difficult to say they have to have direct contact in such situation. But, it is better if there is a way that you can go and have contacted them without bureaucracy. They know the capacity of the woreda. Sometimes we use ambulance that is supposed to be used for mothers. We don't have cars that are designated for this purpose. If you use Ambulance for programs and RDF drugs, the problem that can happen here will be the worst. It is better if any vehicle is allowed to be used. In fact, there might be theft and other problems in the process and they are requesting a car to prevent such problems. However, it is better if any vehicle is used after setting responsible body. This can overcome the challenges. You directly go there, pick it, and return. Transportation is an important thing in supply . Having direct communication can solve the problems.

I: What about storage capacity of other facilities?

R: [health facility] has good store. It has the capacity to hold any type of shelf. This one is smaller. We cannot hold extra products. I don't think the rooms were built based on plan. The laboratory is small. Infrastructure and buildings are challenging as a country. It needs expansion.

I: Can you please tell us about the implementation of IPLS?

R: I cannot give details about IPLS. He got the training very recently. We haven't started the implementation yet.

I: IPLS includes the utilization of formats like bincard, HPMRR and IFRR. Do you have enough formats to include model 22? It starts with using those formats.

R: I can tell you about this. except model, we make all formats printed including the bincard.

I**: Is there any shortage?**

R: its shortage exists. If there is shortage of IFRR, we will print it here. The HPMRR is printed by the printing company and given to the health posts.

I: What about RRF

R: We have enough RRF

I: How frequently do you request emergency orders?

R: We request it most of the time. we request it for family planning methods. When I was in [health facility], I needed to have 1500 including for the four catchment areas. But they had only been giving us only 500. *(pause*) so you frequently fill emergency orders. The amount they provide us with and our needs are not similar. There was a situation when we cannot reach all the health posts. The population was very dense. It is around 48000. Then you request an emergency order. Then they give you 300 or 200. That's enough. Again, feel it and go. It is difficult.

I: how is the IPLS not implemented so far?

R: Training was provided recently. Additionally, there is shortage of manpower. IPLS needs manpower and budget. Eee, we need to organize the room. However, IPLS is functional only in Seka health center. They are working on it. it needs rooms including counselling area.

I: that one is APTS, IPLS is being implemented here.

R: ya ya, you are right.

I: What about data quality when the HPMRR is filled by health extension workers?

R: there is big skill gap. They do not know how to fill it. We made him go there and fill it. Although

we show them how to fill it, incorrect data will be provided next month. He will go there and supervise them or show them here how it is filled.

I: how about wastage and what is the cause for wastage? Especially for program products.

R: there is no drug wastage nowadays. shortage is common than wastage since the insurance is started. The procurement system has also changed. The product that is supposed to be available in hospital was available here too. There is a system in which we exchange it. If it is extra here, we can exchange with hospital or other health centers. There is no wastage at this time.

I: what about over stock? Do you do stock status analysis?

R: we were not doing it previously. But now we are forced to do it through [another eLMIS}. We can see that the health center is overstocked. We also take from them. Woreda will also see which facilities are under stock. You can follow it without any difficulty. [another elMIS} can solve many problems.

I: What challenges do you face with its implementation?

R: Network is the main challenge. Other than that, it is good. It is something you can easily do. Both of us – me and the pharmacist use it. It is easy to use. We will travel to a place with good network to sync the filled bincard.

I: Is the network in this area poor?

R: it works but it is not to level of the network the database needs. It is very slow.

I: what about data accuracy and support?

R: The only support is provision of training. Other costs are covered by the health center. They provide support in terms of man power. There is review meeting every six months.

I: how do you utilize the data?

R: we can easily check which drugs are going to expire. If there is drug which is going to expire, we will make that drug transferred to another facility. It shows these drugs are near expire. It shows 45 days and 60 days. It shows stock on hand.

I: does it require internet all the time?

R: It works both online and offline. But to check what is filled it should be online. When I need to check it, I use hotspot of my phone or use wifi when I return home.

I: I think this is providing you with good information. What should be improved?

R: if there is any option it can be used offline. Or make it work on a lower-level network. For example, using 2G network. 3G and 4G networks are not working here.

I: How do you manage expired drugs?

R: expired drugs will be listed and we will remove them from the shelf. There is a room where it can be stored. It will be listed and reported to the woreda We will manage it this way. The problem we have now is burning it. it has been stored for years. But this time there was no drug that got expired. The insurance reduced the wastage rate. Previously only few people came to [health facility]. Drugs may not be used for six to seven months. The chance of it becoming expired is high.

I: Is computer available in the store?

R: no. the organization provided Samsung tab. He uses it to fill it out.

I: what do you recommend to improve inventory management system? When we say

inventory management system it includes, store management, first in first out. This included strengthening linkage between health posts through the use of HPMRR. Working capacity building.

R: all these things can be functional by humans. We need to work on workforce. You can only provide capacity building if you have the manpower. I would only say increasing the manpower. To work actively, one person should be in one place. when you assign one person here and there, one thing will be missed. Additional manpower is needed to run these things.

I: How is supply n data generated, shared, and utilized for decision-making?

R: The data will be presented to DTC. When we plan to purchase drugs and request an emergency order we start with the available data. We analyze when it is going to be finished and for how long are we going to use it and then decide it. However, we did not do this thing regularly.

I: What is the main reason for not having it regularly?

R: It is work load. The member may not understand you if you bring it to the meeting agenda.

I: Is there PMT, other than DTC?

R: yes.

I: is it functional?

R: yes, it is functional

I: Are they meeting every month?

R: Even though we have started department PMT. We started this month. They will discuss and evaluate their performance at department level and bring the findings to the main PMT.

I: What is the role of Pharmacy in PMT?

R: Pharmacy role in PMT is to report pharmaceutical supply and we also discuss the existing unit level problems. We will evaluate monthly performance of his department.

I: What is the agenda for PMT? You use DHIS2 or overall facility performance?

R: it is DHIS2

I: Are supply n indicators reviewed?

R: When we do LQAS, if the selected indicator is pharmacy data, we go and check it. For example, if he fills 1 for amoxa while it is not available, you can check it. if it is available, you will say yes. If it is not available but he filled 1, you will say no and discuss on the found gap.

I: that means you check data quality.

R: yes.

I: What about reviewing indicators performance?

R: Yes, they are being reviewed.

I: So, you do problem prioritization and root cause analysis?

R: yes, we do it.

I: I mean for supply .

R: no, it is not done for supply . But we do it for other indicators.

I: what is the reason for that? Is it not giving emphasis to it?

R: it is just not giving attention to it. No other problem.

I: What should be done differently to improve this?

R: you know what, it is not based on your choice, it is done based on random selection. Most of the time we do not reach that indicator when you take the first 85 or 100. I cannot say this is the challenge.

I: How are you addressing the stockout and shortage? Is there anything that can be taken as best practice? Consider the health posts.

R: there is a system in which they request for it. We refill them based on their request. There is RRF, I mean HPMRR form to request it. They will fill it out and bring it here. If it is available here, we will give them. If it is not available here we ask from woreda or borrow it from other woreda. For example, if the health extension worker asks for Amoxicillin and it is not available here, we identify health center which has it, then we take it from them by providing other drugs to them. We are resupplying them in such way.

I: Any other best practices that you can state about supply n that can be scaled in the future?

R: I am not sure if it can be best practice or not. The way we borrow products without any bureaucracy is interesting. We share it from a health post which has more products to other health posts.

I: You have told us about stockout, problems associated with resupply, and capacity. The ultimate goal of our project is to identify how to reach the community with RMNCH products? In this regard, from your perspective, what should be done by the facility, health post and woreda to reduce stockout, wastage and have sustainable supply n management?

R: it is better if there is scheduled communication between health posts and health centers. It is better if we use these formats appropriately. It can be HPMRR, IFRR or RRF. We have to use it properly at health and health center level. We need to strengthen DTC. We need to know what is expected to be discussed during the meeting and what the agenda of the DTC. It be conducted based on the plan. It is also better if training is given on supply n management. When we say training, it should not be going anywhere. It can be on job training. I think this gap can be solved if work is considered as part of our job starting from lower level. Additionally, it is better if we need more relationship with the supplier. We can solve the problem we had been discussing if the woreda and the supplier give us immediate response.

I: Thank you so much for the information.

End of interview

Transcript (6)

**Name of Project:**	[project]
**ID Code**	KII-OA -14
**Type of interview**	KII
**Type of informant**	[sex]
**Region**	
**Role of the Interviewee**	Supply n management expert
**Years of experience**	14
**Name of Transcriber:**	[Lecturer}
**Date of Transcription**	August 2024
**Length of interview**	Start time: **9: 05 AM** End time: **11: 05 AM**
**Names of Reviewers:**	

Start of interview

I: Can you describe your role and responsibilities within the health supply n system?

R: I have worked for more than 14 years with work experience including pharmacy supply n and pharmacy services starting from the facility to here in [Region] health bureau. Now I am working as a supply n management expert in [Region] health bureau.

I: How do you perceive the overall effectiveness of the supply n management system?

R: To talk about supply n management status in [Region] region, there is one supplier as a country which is called [SUPPLYING HUB]. It works to program drug procurement, distribution, LMIS activities in government structures starting from health posts. Our facilities request for drugs, especially program drugs, then the suppliers will resupply the requested drugs according to the set schedule. We have designed inventory control system as a country and [Region] region is also governed by that system. The RDF or budget drugs are not governed by this. That means the inventory control system is not like the program during that time is supplied based on the preset schedule. So, there is no strong supply n management system for budget drugs. More or less, we have a system called IPLS that we use to manage our supply n system in the region.

I: what about timely delivery of commodities, facility readiness in reporting and data quality?

R: As I have said earlier, the inventory control system is scheduled and they have to provide the report according to that schedule. However, we know we have pharmacy workforce capacity, competence and skill problems both at region and country level. Therefore, facility level data quality, data generation and use has not reached maturity level. Due to these reasons, there are interruptions both for program drugs and RDF or budget drugs. There is inadequate supply at the facility level. Although there are supplies from supplier, drugs are not reaching the facility due to data quality including not reporting timely. This is only applicable for health facilities that are accessible. Our health facilities that are not accessible have the worst problems. So, the problem is inaccessibility and those which are accessible have data quality problems. Those inaccessible health facilities face more health commodities shortages. In addition to that there are security

problems. Due to these reasons, it will be difficult to say the commodities are reaching health facility timely.

I: Can you tell us more about the challenges for continuous availability of health commodities at health facilities and health posts?

R: According to inventory control system, it is assumed the facility has the drugs that can be used for four months. To have this, the facility needs to have enough storage. This is not adequate for all facilities in the country. So, it is one of the problems. At the health post level, if I start with its design, there is no storage. The health facility faces problem to store to the commodities according to the inventory control system. The other is the transportation problem. This means as our suppliers face problem to reach all health facilities, our facilities are forced to collect it themselves. At this time, they face problem as there are not enough vehicles. Our facilities do not have specific vehicles that can be used for transportation of commodities. Due to this we also face shortage and interruptions.

I: what about workforce? what challenges do we have?

R: I have mentioned it earlier. There is shortage of pharmacy professionals. We don't have adequate number of pharmacy professionals in our health facilities. In [Region], it is less than 50% according the criteria set by the civil service. It says three pharmacies professional if the facility is B type and five, if the facility is A type. However, it is not fulfilled for many facilities. Secondly, there is a competence problem from the pharmacy professional side. This can be related to capacity building. Most of the time they work based on what they learned at university. They are not getting enough on-job training. Supportive supervision and mentorship are also not enough. There is a gap in this area as compared to other health professionals. Many things remain in terms of improving supply n competence and skill. We need to work on providing training and supportive supervision.

I: Can you tell us about performance monitoring and recognition mechanisms?

R: I have been working in this region, there has been no performance monitoring and recognition mechanism for the past 5 or 6 years. Everything is the same for those who are working better or not. This can also be a barrier for performance improvement. A person who works the improvement of supply n must get recognition. There is no such experience so far. We have planned to do it in the future. The region will work on it at least for those who are at zonal, woreda and facility level. It can be education sponsorship or making transfers from facility to woreda office or from woreda to zone, or from zone to region based on performance. I cannot say I have done anything in this area so far.

I: Can we say staff commitment can be challenging for the availability of commodities? for example, timely request, proper recording, data use for decision making?

R: regarding the staff commitment, we can say it is in good status for some areas. But in some areas, there is low staff commitment which leads to poor data quality, so they face interruption of supplies. But in some areas like [zones], the pharmacy workforce is so committed that, they have better data quality, then they have better supply n management and better communication with the supplier. Since the supply n and data quality have connections, they have better supplies. If the data quality is good, [SUPPLYING HUB] will not face challenges. For example, if we take [zones], they properly do refill analysis and send it to [SUPPLYING HUB]. [SUPPLYING HUB] will deliver the drugs it has at hand on time without a problem. On the other hand, in areas where there is a security problem, they may not be committed as they get less motivated. So, there are zones, woredas and facilities that are doing better, while there are zones, woredas and facilities that we have to work on.

I: what about management support?

R: I can confidently say there is good management support in [Region] region. Even in 2016 E.C. we got good budget and have been working on capacity building as our gap is in that area. As I have said earlier, there is a capacity gap among the pharmacy workforce. To improve supply n management performance and activity, we have provided training for facility, woreda and zone level workforce. This is because the regional management has provided us with enough budget.

We have been providing supportive supervision in collaboration with partners. Integrative supportive supervision was given to program drugs like HIV and malaria. However, there are problems in zone and woreda level management. They have no budget allocated for this purpose specifically for supply n and pharmacy services. Regional management cascaded integrated supportive supervision and review meetings for supply n and pharmacy services. However, it is not implemented practically. So, supply n management support is better at region level but not good enough at zones woredas, towns and facility level. Pharmacy workforce was not included in carrying out supportive supervision. They tell us they face budget shortage to visit health facilities, even once a year. In the [Region] region, we have allocated a budget and undertook supportive supervision for each zone and town. Commitment at region level is better. There is a management support gap at the zone, woreda, and facility level. [SUPPLYING HUB] provides the program drugs, there is a sense of considering it as the responsibility of others. There is a challenge with accepting and approving the RRF data that is generated. Failing to review the generated data by the performance monitoring team for its quality is a management problem. So, one of the activities we are going to do this year is to make the management support the supply n management and use the data for decision.

I: can you say something about induction and skill transfer?

R: I remember we conducted it a year ago. The document was developed by the ministry and we adopted it and transferred to zones and woredas. However, their practice is not good enough. A lot of things are still remaining. For instance, some activities become new for them when there is turnover. But in some areas, especially those I have mentioned earlier, they are inducing and some of them are also doing skill transfer. Some of them will be assigned to activities without getting orientation and this will create interruptions and affect the data quality. I want to say it was conducted at region level and only a few of them have practiced it. The implementation remains at the lower level.

I: what is the main reason for this?

R: It is commitment issue. As a region we called the responsible person, showed and discussed it with them. We discussed in detail with zones and town administrations. Then they are responsible to discuss with woredas and facilities, work on staff induction and share it with staff. The gap was in cascading what is done in the region to the lower level. As we cannot reach all 1400 health centers and 122 hospitals. The practice is not as expected. So, when a new professional is recruited, we need to provide orientation and training.

I: What do you recommend to be done in that area to improve supply n management: What should be done to fill those gaps?

R: Good. We have manuals and formats like LMIS formats. First, it is better if we work on capacity building activity especially at woreda and facility level. There is a huge gap at woreda level. Secondly, making the workforce there familiar with our formats. It can be softcopy or hardcopy. We have learned that we develop SOPs and manuals, and then share them zones and towns, but they are not reaching woredas and facilities. We are distributing the updated LMIS formats to zones and towns, but our facilities are using the older LMIS formats. This is due to a communication gap and we have to work on the communication area. I hope it will have better outcome if we work on supportive supervision and communication activities by using the existing system.

I: Can you describe the current quantification and procurement process for RMNCH commodities (through program and RDF schemes)?

R: quantification of the program drug is done at the national level. At facility level, as I have said earlier, they do inventory control and report it every two months, then collect program drugs. So, they do not quantify. For the future it's better to consider it. We have also given a comment on it. It is better if the quantification for both the program and budgeted drug starts from a lower level. We are not doing quantification for program drugs at region and facility level. this is being done at the national level based on HMIS data. Our role is to collect RRF data report. Quantification of budgeted drugs have now started. Training have been provided at the national level. In our region, the training on the quantification of budgeted drugs is provided to all hospitals. There are facilities

that has done quantification exercises and reported them. However, the challenge is the what they forecasted and the budget has big difference. Hospitals have experiences of doing quantification. But there is budget shortage to do procurement according to the supply plan. Overall, there is budget drugs quantification practice at the hospital level. When we talk about health centers, previously [SUPPLYING HUB] go and help them do the quantification which is not functional at this time. They didn't receive quantification exercise training. I hope in the future they will do quantification just like a hospital and get the budget. The current practice at the health center is, the woreda do the quantification for each health center once a year, then send it to us. Then based on that, the region will sign an agreement of 300,000 Birr for each health center with [SUPPLYING HUB]. Based on that [SUPPLYING HUB] will buy and distribute it. This is what is being practiced. It is questionable how 300,000 birr is enough for one health center. So, the procurement and quantification practices are different for hospitals and health centers. Hospitals do their own quantification and purchase it themselves.

I: What are the main challenges encountered? Including RMNCH commodities forecasting accuracy?

R: One of the challenges for RMNCH and other program commodities is … quantification is not done at a lower level. It is done at the national level and facilities request it to collect it. It is better if the facilities do the quantification of RMNCH products. Then, the facility informs the supply they need. Because of that, there is a capacity gap to quantify correctly. It is better if the privilege of quantification is given to hospitals and health centers. Training should be provided to improve their capacity of quantification exercise. Secondly, we also face challenges to get data that we need for the quantification. It is better if support is given at the facility level on these issues. Supportive supervision on recording reporting of the data that we need for quantification. There is also a big problem with data quality. There is no privilege to quantify it. You will only receive the products on request in this country. You cannot do quantification and ask for the supply of RMNCH according the current practice. So, it is better if to the quantification is done at lower level, but the capacity building is provided to them to do the quantification.

I: You have told as some point about the transportation. How is the transportation and distribution system for RMNCH commodities?

R: ehhh … it is just like other program drugs since the RMNCH drugs are also transported with others in integrations. The storage is also in integration. So, any distribution and transportation problems that affect other dur also effect it. Earlier we had said there was transportation problem. This is due to vehicle shortage at health facility, due to infrastructure problem, due to presence of man power problem. The other is budget shortages as there is no management support. If the supplier is not delivering the supplies on time, the facility needs a vehicle go and collect the commodities from suppliers. Eee it also needs fuel, it needs manpower, so there is a gap in these points. This means it affects RMNCH product delivery. Data quality is one factor for the distribution. If there is no quality data, some unnecessary products will be distributed to somewhere. So, distribution faces some problems. The factors that I have mentioned earlier can affect the distribution and transportation of RMNCH products.

I: What is the capacity of [SUPPLYING HUB] to deliver these products?

R: Okay. I have raised the problem from our facilities, woredas and zones side. The problem for the supplier side (*people talking from the background*), eee … there are problems, … there are barriers that they raise to delivering it on time. That is, the first unavailability of enough vehicles. Eee … secondly, it is ours. It our facilities are not sending the report timely. They raise the problem of data quality. On this area what we have learned is; supply n needs immediate response. There is a problem of delivering products from the supplier side, while there is vehicles, drugs and reports that have quality. It might be due to system problem but, they do not give an immediate response. So, it creates interruption of RMNCH products. Secondly, eee … [SUPPLYING HUB] must respond to emergency delivery, according to IPLS. The one I have raised earlier is about the routine one. If the report is submitted every two months, [SUPPLYING HUB] must deliver the drugs it has at hand within two weeks. However, it may take up to a month in some areas in the current practice. This will disturb their min-max. do you understand? The other thing is when there is an emergency, [SUPPLYING HUB] is supposed to deliver it once the facility submits the report. This is because, as we have said earlier, they have no capacity. The supplier is budgeting to deliver the products and there is no additional budget provided to the facilities for transport to collect the pharmaceuticals from the supplier. So, the big problem is the facility itself go, and collect it at the time of emergency. This happens frequently. The other is a security problem. The agreement at

national and regional level is that [SUPPLYING HUB] delivers directly to accessible facilities, including for RDF drugs. [SUPPLYING HUB] delivers both programs and budget drugs to our accessible facilities. But, when we see the practical thing, the [SUPPLYING HUB] only reaches the woredas, or even zones. (Long pause) they are not even reaching an accessible health facility due to fear of security. They leave it at zone, especially in [specific zones). It is a big challenge. Here in the [zone]. They put it at zone level and zone deliver it using ambulance after facilities experience stockout. Due to the current country level problem associated with security,, [SUPPLYING HUB] only delivery to facilities within a radius of 50 KM from its hubs. So, zone communicate with woreda and woreda with facilities, they collect the products and use the existing transportation modality. So, this are challenges from the supplier side.

I: Can you tell us about route optimization?

R: Route optimization, …. We have also raised about it previously. For some facilities, they use the route that was designed a long time ago. For instance, if there is hospital or health center around here and the facility not included in the map, the deliverer refuses to provide it to that facility and goes to other the woreda. Then that woreda will bring the products back to that facility. In my understanding it is better if the route optimization is revised and mapping is done again. This is the problem we are facing in [zones] They are reaching health facilities by passing the one which is not in the route. This should be changed to save transportation cost and deliver the products on time. To reach the facility directly and to save time, it is better if the route is revised. Other than other problems like security issues, almost all health facilities are accessible. Now, due to this factor most of our facilities are being considered as inaccessible and the drugs are being delivered to woreda. So, it needs route optimization. Another issue, … what we have faced is, you know, Bako (small town in west [Region])?

I: Yes

R: You Gedo too?

I: yes

R: you see, (hospital) and (health post)are found in the same town. One of them is on this side of the road and the other one is on the other side of the road toward Addis Ababa. The [health facility]

which is on the side closer to Addis Ababa is provided by this hub, while the other one is provided by the [SUPPLYING HUB]. This is a big loss as a country. So, route optimization should be revised to this extent.

I: What about the challenges delivering from health center to health posts?

R: I may say data quality is the main challenge. The storage capacity of the facilities can also be a challenge. (*People talking from the background*). Health post accessibility and their storage capacity is challenging. We have raised this earlier. There is no building or premises that is considered as pharmacy store at health post. So, there is a small shelf where they can store it after collecting it from the health center. Previously they had been taking products used for a month, in case their capacity of holding products is increased according to last mile delivery, they are not able to store more products. If the schedule of the last mile delivery is similar to that of IPLS, hospitals may face pharmacy store problems as it is not enough and not in a status to keep the standard storage condition. The other thing is, the data quality problem which needs to work in it. This is it if I understand your question.

I: it was mainly related with the transportation, especially challenges related with delivering at lower level. What alternative transports are there to delivery commodities? It from health center to health posts.

R: I might have raised this earlier. Since the [SUPPLYING HUB] or supplier has no enough vehicle, our facilities and woredas are forced to collect it, especially during the emergency time. Still, this is a challenge that we can face during the last mile delivery. So, our option is at least we have ambulance at woreda and hospitals level and it can be used. But big problem is … eee, pharmaceutical supply transportation has no budget. Budget is allocated only for procurement. Maybe they can communicate with [SUPPLYING HUB] to cover the fuel and other costs to be covered by them. It can be solved in such a way as this kind of practice was previously in place for some areas. Last mile delivery can be effective by using vehicles from the hospital, woreda and health centers, while [SUPPLYING HUB] covers the fuel and other costs. Based on the previous practice, I don't think [SUPPLYING HUB] can reach hospital, health center and health post. It might work way I have mentioned now. If they work together using woreda vehicle or then

they cover the cost of fuel and perdiem. (People are talking from the background). As the ambulances are also available. It is possible to delivery products to health posts by using motorbikes that are available at the health center level. (People laughing in the background). But there are health centers that have no motorbikes. It has been a while since they were provided with motorbikes. It can be challenging if it is not functional. Providing them with it may be a solution in case there is a support mechanism. It is better if motorbikes are provided to health centers for delivery pharmacy products to health posts. Other than this, if the capacity issue is related to LMIS implementation, it is possible to deliver it as we also have experience. For instance, if we take the [zones] areas where you might have also information, health post resupply activity is in good status. If the problems I have stated is addressed, (phone ringing from the background) I hope it is possible to deliver. The big problem is transportation and vehicle shortage. It needs to work continuously with woreda. Woreda and the supplier do not know each other. [SUPPLYING HUB] directly communicate with the facilities. They may discuss with the zone if they face problem. The is no communication between woreda and [SUPPLYING HUB] or supplier. But communication is back bone for supply . So, it is better if the supplier work with zone and woreda management body both for distribution and transportation. There is technical working group at zone level and its better if they discuss about it on this platform. I hope it is in good status at region level. As our director is member of the team, they discuss on it when there is distribution and transportation problem. Then they bring up solution and it will be communicated to the lower level. [SUPPLYING HUB] is not working with zone and woreda but facilities. The facility has no capacity, the woreda is not giving supporting on the issue, so there will be interruption. So, it needs working with woredas … to solve transportation and distribution problem. Working on facility level infrastructure problem will also help in the implementation of last mile delivery.

I: What about deployment of third-party logistics?

R: We also has recommended such things especially for security affected woredas and security affect facilities. What we said was, outsourcing transportation service. We discussed on it at region level a year. [SUPPLYING HUB] has tried to implement it in some area but, they are not successful. The problem is, the relationship between our supplier and the transportation sector is not as such strong. They just deal with individual owner and the level of sharing the challenge was

not clear. They just have a deal informal deal with individual owner and it was not successful. When such problem happens, especially in security affected area, we recommended [SUPPLYING HUB] provide their vehicles to private transportation sector. They were not okay with is, I do not know whether it is their internal policy. This good option that we also had been thinking about. Government vehicle is Code 4 (Plate?), and we had been using code 3 private vehicles in [zones] We have recommended it. But they said their financial rule is not flexible and [implementing partner] for such mechanism. If they outsource the transportation service, especially in security affected area, it will support last mile delivery.

I: May be (participant name), what I want you to explain is on from woreda to health center and from health center to health post. Last week I was at [zone]. As you have said, there are transportation problem, infrastructure problem and data quality. You have provided some recommendation about fuel support, motorbikes. What I saw in [zone] is there are place where it is not possible for motors too. What do you recommend to facilities where the woreda doesn't have vehicle and the [SUPPLYING HUB] cannot access the facility?

R: the trend we use so far is to use traditional transportation. We had been using traditional transportation where their problem of infrastructure and security issue. This is the last option we have. Our other recommendation was (Lough), the one which is used by many African countries. We use a drone for inaccessible facilities ya? It is used in Africa country like Kenya. Sometime it is better to think that way. Why don't we use to delivery for vital drugs and vaccines? I know it is costly, to save life, ee … it is good to plan such option as a strategy. What other option do we have? You may use manpower by covering transportation cost. Second using traditional transportation as we already have the experience, especially in [region]. First we need to design how we are going to use that traditional transportation and who cover the cost. It needs developing a system. Previously it was informal. It was only used in hurry when there is problem just to deliver the products. So, if resilient supply n is need, we have to design it well. It should be decided on who should pay for the transportation whether it is manpower or traditional one. Drone can be used, but it is costly for us. But to save life it is possible. It is better if we think of modern transportation. we don't have it even as a country. I think Kenya use it to transport vaccine. This my recommendation for last mile delivery.

I: What are the main implementation challenges of the IPLS in health facility?

R: eee … IPLS is the system we have for supply . I hope it needs to be updated. As a system we have been using it for more than ten years. In some place (phone ringing form background) health facilities report monthly while it s every two months in other places. So, I think this has to be similar. Or we may update the IPLS and make it based on accessibility. By the way we are facing challenge to evaluate it. Drug max-min is two months in some place, while it four months in other places. For example, the store we need for the four month is different from that of two months. The inventory management mechanism will affect the storage, it will affect the distribution. It also affects the LMIS too. So, let us update it, the IPLS. Other than that, we have mentioned about the capacity building earlier. The training is given once they then work for five or four years. But the supply n needs update. Even it needs to update how to request program drugs. It needs to provide training g immediately after the updated does not on LMIS formats. The programs are updated, the products enter the country, but the facility use the old formats, so you will not find the product in the facilities will product is available in the country. So, when the product is available, the supplier should communicate it and update the LMISs. When the products are updated, pharmacy professionals should get that update. How can he ask for the new product if he is updated about it? The system is there but when the is updated on program drugs for TB, Malaria, HIV the LMIS should be updated and the workforce should get training and supportive supervision.

I: Any challenge related to utilization of LMIS formats and emergency order frequency?

R: yes, I mentioned this earlier. Emergency orders have been implemented according IPLS. We have a design, we have a system for emergency orders, okay. However, there is a problem with its implementation. The facility is not filling the format and sending it on time whether it is on [eLMIS] or hard copy. Although there are facilities that fill and send it timely, [SUPPLYING HUB] is not delivering it. Now, we can say that IPLS is totally not functional for the emergency supply . You have to order emergency based on the set platform, then the supplier will deliver based on that. The emergency resupply must be within 72 hours, but a week is normal now. So, the problem is facilities are not using the format of emergency request appropriately. Some facilities use telegram. They also face problem to use [eLMIS] for emergency orders. As an expert,

I learned that it need investment to use [eLMIS] for an emergency orders. It requires training, monitoring and mentorship, especially by discussing with IT professionals. It needs more mentorship. On the [SUPPLYING HUB] side, they should delivery for the facilities that properly requested emergency order. Their biggest problem is responding to the emergency requests. They are responding after a week or two of occurrence of the emergency. It will be difficult to say we have a system in such a situation.

I: what about wastage rate?

R: is it wastage rate as a region?

I: yes.

R: to reduce wastage rate as a region, I can say we come long way. What we did was, we developed a guideline on product transfer in the region three years ago. We promoted it during review meetings and now our facilities are using it. The other thing is APTS is being implemented and its main function is to reduce the wastage. More or less our hospitals have implemented APTS. Some health posts also implemented it. So, there is nationally set wastage threshold, which is less than 2 %. For example, ours is 2. We are working to make it less than that. But the problem is related to some program drugs that were distributed using push mechanism, especially for COVID 19 which was distributed 3 or 4 years ago. This will increase the program commodities wastage rate. The cumulative percent region is around 2%, it is 2.1 or 2.2. But at the facility level (phone ringing in the background) we go up to 3 as there are products that are provided using push mechanisms. As a region we are working to reduce wastage by making them implement APTS, presence of stock transfer practices and not accepting support for products that has less than one month period. The stock transfer practice helped us a lot with this regard.

I: what challenges are there to implement [eLMIS] it terms of Infrastructure availability, functionality, data accuracy, support mechanisms, and data utilization?

R: the coverage has reached around 339 in the region. Out of 1427 health centers and 111 hospitals we have, we have 339 [eLMIS] sites. That means the computers were purchased and it was installed. For this, we have identified 139 of them functional during assessment. From this there are on-off facilities. This is what the status of [eLMIS] looks like. The problem is, the system is

not friendly. We know our professionals are not good enough even in using excel as there are someone who directly joined the work immediately after graduating from school. The training was given to IT and pharmacy professional, then IT expert from center install it and provided orientation on how to use it. But, since the troubleshooting training was not given, they stopped using it when there was a problem. Close mentorship and providing remote support were needed, but this not as strong as there is shortage of manpower. Since there is no support, the service is interrupted. IT experts at zone and town level have received training but they are negligent and have no commitment. There is a problem of giving attention just like they give attention to DHIS 2. They gave more support to DHIS 2 while ignoring this one. Since the pharmacy professional does not know how to do the troubleshooting when there is a technical problem, it is getting interrupted. We have evaluated it and expect the IT professionals will provide support. To increase the coverage, there is shortage of computers. There are around 1500 health facilities including the hospitals and only 50% them have access to electricity and internet. So, 50 % is around 750 health facilities. We need to have more computers and printers to reach all these facilities. These are the challenges we have with the implementation of [eLMIS]. In collaboration with the Ministry, we plan to deploy and make the existing functional in 2017. We have discussed with people from to communicate with IT professional at zone and woreda to support on stock on hand synchronization and RRF sink. I haven't heard it status at the national level. It is a big challenge by the way. To digitalize IPLS, besides the partners support, it needs government acceptance of its role in reducing wastage and increasing stock availability. Otherwise, it is difficult. (Long pause) how long is it since [eLMIS] is implemented? It is not mature yet. I personally have no health facility that I can say it has implemented very well. This because government didn't take the ownership. Sadly, there is internet service in DHIS 2 room but, not available in pharmacy store which is not more than 10 meters away. You will face problem to install it in such areas as there is no management ownership. For the presence of digitalization services there should be management ownership. We have discussed on this and hope it will be improved.

I: What are the challenges of inventory management systems in woredas, health facilities and health posts? Is the stock status analysis being carried out?

R: Eee, yes, it is done. Especially in hospitals and health centers which are doing APTS. They also receive interventions. When they do stock status analysis they are forced to receive important intervention. But it is not as satisfactory in terms of health facilities we have. Eee as I mentioned

earlier, inventory management is four months maximum and two months minimum. Taking intervention based on the analysis of the stock is very challenging. We can say this practice exists in hospitals and health centers which implemented APTS. The first problem is know-how. They get the know-how of stock analysis during IPLS training. If they get training five years ago and they don't have know-how, and there is no system which supports it, doing stock status analysis will be forgotten. So, you need to develop and inform the formats used to do it, availing it, communicating what intervention the take after doing it. Nothing is done in that area. Training was given and the way they are using it should be closely monitored through mentorship.

I: How do you evaluate Understanding of HEWs on the stock out triggering points?

R: Previously, the relationship between health post and health center was very strong. Nowadays, their activity is not good in as a region and country due to different reasons. When the pharmacy professional or pharmacy store goes there to provide supportive supervision, there are many gaps. They will be resupplied every month. They are supposed to collect if there is shortage, by monitoring it using bincard. Orientation and supportive supervision have been given. The health posts have now been upgraded to a comprehensive level. The comprehensive health post has a pharmacy and laboratory and we hope it will be improved. It will trigger them if they are using bincard but, they are not using it. Now a days many communicable diseases are rising and this shows they are not working. But there are places where it is implemented well. Especially in [zone], there are health posts that use the bin card properly, check their stock and report using HPMRR. There are about 7000 health posts in [Region]. In this regard, we cannot say what is being done in [zone] represent all these health posts. LMIS utilization is poor at the health post level. So, it needs capacity building, it needs supportive supervision, it needs availing LMIS formats, eee… it needs to update them, it needs to tell them what triggers the stockout.

I: What are the internal reporting and resupply system's challenges with the facility?

R: Okay, eee … internal reporting and resupply of the facility depends on the health facility commitment. It requires the involvement of many departments. If there is no management ownership, the departments may not stick to the schedule. The pharmacy store and management must enforce reporting on time. It is the role of CEO and PHCU director but there is negligence. They say to provide the drug if it is available. If the pharmacy store manager refuses to provide it for those who are not bringing the report, the PHCU director or the CEO force him to provide it.

This is the big challenge they were raising. Eee when the management body is supportive, the pharmacy store manager should support and supervise them to fill the IFRR correctly. He has to inform them to come and collect the products based on their schedule. There is a problem with this regard. But there are areas which are using it well, where the drugs are not distributed without IFRR. Gaps are there in many places due to these reasons. Most of the time they visit the store when they are out of stock without a schedule. At this time, it will make the store busy and the management body enforce him to prevent interruption of the services. In such situations, we will not get actual consumption and it will affect the overall data quality of that facility. In this area, it needs to be discussed with directors and CEOs during the review meeting and supportive supervision. This is a big problem associated with [eLMIS]. We hear that requests are not based on the schedule. Our partners are not discussing it with the management body during their supportive supervision. They go to store manager. Zone, Woreda and facility management have no information about it. We have commented as the n should not be in such a way. There should be management ownership to support [eLMIS], and we hope it will be corrected.

I: How is supply data generated, shared, and utilized for decision-making within health facilities?

R: eee (long pause) utilization of LMIS data for decision making is in infant stage even at region level. There are many gaps at the facility level in terms of generating, analyzing and using data for decision making. Most of the time they send the report and there is no practice of triangulating the data for decision making. They don't have the know-how. Since they don't have the know-how of triangulating the service delivery and supply n data, they are not using it. As our data use culture is poor, they are not practicing it. With initiative, we accepted its importance as a region, we are providing support availing LMIS formats, then checking the tallies are done from top to lower level especially for program drugs and tracer drugs. This will help utilize the generated data used at the facility level. After utilizing it they will communicate with the stakeholders. At the region level there is pharmaceutical and medical supply performance monitoring team and, we have shared role and responsibility for each member. As a good opportunity, we have provided monitoring and evaluation training and it has also cascaded it up to woreda level. [inaudible 1:42:51] we have provided medical supply monitoring and evaluation training to all hospitals, woredas, towns and zones in 2016 E.C. We hope …. our region level pharmaceutical and medical

supply performance monitoring team will evaluate the data for monitoring and evaluation of the data that was brought from the lower level in 2017. We hope there will be better data quality as it will monitor the data that come from lower level, give feedback. Previously the RRF were being filled, but its accuracy, completeness and timeliness was not checked. Other programs like TB, Malaria, and HIV are being reported after checking the data quality, but the pharmacy service was not like that. They fill and submit some format to get the drugs. There is big gab with regard to recognizing it as facility data and utilizing it.

I: what is the main reason? Is it related to behavior or, lack of motivation?

R: we can say knowledge gap, … competence, and lack of know-how. Secondly, as a health professional our data use culture is very poor. Data usage culture is not practical when it comes to supply n data. Additionally, no one follows it. If there is no accountability and no one asks you why you are not using the data you generated, you will not be concerned. I think these are the reasons. Therefore, supply n data is not used due to skill gaps and lack of accountability. No one asks you if you generate poor and false data. Supply n data can cause over-stock or under-stock, and lead to service interruptions. This is our data utilization culture as a country.

I: Are performance monitoring teams at lower levels using supply n data? What is the role of pharmacy in the team?

R: The performance monitoring team exists at the facility level. I mean starting from region to lower level. But the big problem that we recently learned is that, a performance team is not checking and, monitoring pharmacy indicators, both supply n, pharmacy services and medical device indicators. So, at region level we have established supply n and pharmacy service PMT and we hope it will be established at lower level namely at zone, woreda, town and facility level this year. We are specifically working on it in [zone] as a in collaboration with . We have started a pilot to help with the use of supply n and pharmacy service data at facility woreda and zone level. There is a big gap but we hope we can move forward. PMT sees only other services in the facility and pharmacy indicators are not checked or reported. Despite it being the backbone of the facility, the pharmacy indicators are not reviewed in the hospitals. … the data quality is not checked and this is a big gap.

I: you have been saying a lot but can you give us recommendations on each area regarding the supply n? Including workforce, inventory and overall supply n improvement. Anything that can be used as best practice.

R: we were talking about the recommendation for each component. What I recommend for improvement is. The LMIS format should be consistent. I mean, it should be the same throughout the country. At the same time, follow up should be there to check if it has reached a lower level. There are different formats at different places. This will be a barrier to data utilization. If the hard copy is used, … for instance, HMIS has the owner—Ministry of Health print and distribute it. LMIS formats interrupts and the facilities will be forced to use old formats. This will be a barrier for data quality and utilization. The LMIS format should have the owner. Region can take ownership if the Ministry of Health cannot print or avail it. the region can print and distribute just like other formats. Mentorship is needed for supply n and supply n data use. From my understanding, supportive supervision hasn't shown change. If there is mentorship and the health professional working at that facility, coached quality data will be generated. There are many gaps in the area of capacity building. We have discussed ART, IPLS, monitoring and evaluation with DTC members during training. There is a high turnover and training gap nationally. So it is better to work more on training. Another issue is related to partners. it is better if any partner working in the supply n area communicates with zones and woredas to bring ownership and sustainability. This is a big gap. We have support from [partners]. To have sustainability, zone and woreda should be engaged and communicated. If they leave then area the it will be interrupted and you will never get supply n data after that. Orientation should be given Pre refill analysis must be strengthened using the preset format. Only a few partners are working on the quality of RRF data, but zones and woredas are unaware of that. About two zones have got the orientation. Strengthening supply n capacity building and, availing consistent format and, mentorship. It is also better if training is given to the health center on the quantification. So far it is only provided to the hospitals. The tools should be friendly. Hospital tools are a little bit complicated. It can be found in the [eLMIS] Orientation or training can be organized for the health facility to help them use it. we hope quality supply n data will be generated and used after that. Generally, as a nation we need to have similar digitalization. As I have said earlier, there is no ownership, it is on and off, so management at the

facility level do not trust it. So, it is better if we have similar digitalization system that can be used by all just like other countries. Although we haven't checked it, we heard about South Africa and Kenya. Their supply n digitalization is better. I don't know what is preventing us from being like them. If we look at the software in detail, it is not as strong. It needs a big support and investment. We need management support. In terms of workforce the is shortage and those on job are overburdened due to shortage.

I: Thank you very much. You gave us a lot of information. Thank you for your time and contribution.

End of interview

Transcript (7)

**Name of Project:**	[project]
**ID Code**	KII -OA -16
**Type of interview**	KII
**Type of informant**	[sex]
**Region**	[region]
**Role of the Interviewee**	Logistic officer
**Years of experience**	
**Name of Transcriber:**	[lecturer]
**Date of Transcription**	August 2024
**Length of interview**	Start time: **9: 02 AM** End time: **10: 11 AM**
**Names of Reviewers:**	

Start of interview

I: Can you describe your role and responsibilities within the health supply n system?

R: My name is (participant name). I am a logistic officer at [zone] zone health department. Supply n in the zone is led by us. We coordinate it starting from zone to lower-level facilities. Information and reports from lower-level pass to higher level through us.

I: How do you evaluate the effectiveness of the overall supply n management system?

R: Supply n means, it should be linked from top to bottom without interruption. We can say the supply n is meeting its goal if there is no interruption. I can say the supply n is good in our zone.

I: What about timely delivery of commodity and health facility readiness to report?

R: we can see this in two ways. RDF which is purchased using internal revenue and program drugs that are is provided by the government. Health centers buy the drugs from their internal revenue every quarter. For that they will do quantification and show their interest. For instance, they do quantification for a year 2017. Based on the quantification, they check what they have at hand, what money they have and what to buy. They buy it directly from [SUPPLYING HUBS]. For program drugs, there is a reporting system which is called RRF. They will fill that RRF with what they have at hand and what they have used to report it, and then request the drugs they need. So they fill and send that RRF to [SUPPLYING HUBS]. They show I have used this amount for the past 2 months and I need this amount for the next month. They will deliver the products after they check if what the facilities filled is correct and based on their consumption. Regarding the readiness, we will confirm this whether they are sending the RRF or not. The schedule is, it will arrive at [SUPPLYING HUBS] on the 10th of the third month through us. If they are sending it on time, we can say their readiness is good. We can say they are delayed if they are not sent according to the schedule. it is measured in this way.

I: how was the status when it was measured?

R: there is no problem with sending it. There is a project called **last mile project** which is led by [PARTNERS]. We are in transit from hardcopy to softcopy. They gave me this mobile phone. I sit here and follow what each health center has filled and requested. There are 120 health centers and

from this 100 of them were provided with tablets. Logistic officers who are working at a woreda level have a tablet and we follow what is done at the lower level. They are filling the RRF using the softcopy. We have observed a gap in data quality. Ending of balance of last month is beginning of balance of this month, there is a gap in this area. There is also a network problem. We are not progressing to the level we want due to this network problem. For this, we proposed to use both methods for reporting. Until our system becomes 100% effective, they will send both the hardcopy and softcopy. Another problem is associated with timeliness. Some health centers in remote areas are sending it on the 11th or 12th day. However, RRF is 100% reported in our zone.

I: What is the main cause of data quality and timeliness problems? What is the main reason from a personal and system perspective?

R: for example, there is a Woreda called [district] which is very far away. We can say it is a transportation problem. It cannot be simple for them to arrive on time as they do not have their own means of transportation. Their only problem is distance.
I: what about data quality,
R: It is a knowledge gap. For that we have provided training. But, it is not possible to solve it overnight.
I: are they 120 health facility under your control?
R: they are 120 health center and 8 hospitals.

I: how many of them get direct supplies from [SUPPLYING HUBS]?

R: facilities that get it directly are … more than 90%. More than 90% of them get direct delivery. there is Woreda which is called [dis. It is a difficult area to reach during the rainy season. When it is difficult form direct delivery, we use woreda path through. They will receive it based on their quota.

I: How about the capacity of [SUPPLYING HUBS] and Facilities storage capacity?

R: we cannot say they are working 100% according to what is proposed. Sometimes the car can breakdown. We will do an assessment. We will confirm when they come for RDF. I kept a copy of the form they filled out. I register the number of drugs they have requested. For example, this one (showing a copy of the letter) requested for 58 items of drugs. I will call and ask him how many drugs we can get from the 58 items. If he gets 58 of them, it is 100%. If he gets 20 of them, I will calculate them. I will also check how much of this they get from private suppliers. There is an availability problem nationwide. We may not get 100% as it is on and off.

I: There are malaria cases and emergencies happen. Strong supply management is measured in how they coped with the challenges. What is the response to the emergency case from the facility and [SUPPLYING HUBS] side?

R: We can take the malaria case. It is not only in [zone]; it is a problem as a nation. We can say it is everywhere in our region. They are motivated to make emergency requests using RRF. If they come with an emergency request, they will be provided by [SUPPLYING HUBS]. There are drugs that are not available in the country. For example, if we take chloroquine and primaquine, there is a time when shortages happen. If available, it will be provided, otherwise nothing can be done. Coartem is available, you can get it if you correctly show your consumption. They gave us this as an emergency. They report their cases and it will be provided based on their cases. They can provide it as an emergency not only for malaria, but also for other activities. They can make an emergency request at any time.

I: We were also at the facility and the main complaint was related to infrastructure, storage capacity and transportation. Can you share these problems?

R: They may raise this as a problem. They give us the same response when we ask them why they are bringing it on time. It is difficult to say if this is really the issue.

I: How do you see the challenges to deliver from woreda to health and from health center to health posts?

R: We signed an agreement with [SUPPLYING HUBS] to deliver the supplies directly to health centers. They are responsible for delivering it. We also help them when it is difficult for them to reach some health facility. When their car is not able to reach the facility, woreda will send its car and deliver the products. Health center will deliver to health posts using a motor or other means of transportation. This is not [SUPPLYING HUBS]'s mandate. It is mandate of the facility.

I: what I want to say is supply n can be affected by transportation system, storage capacity and other infrastructure. Their min-max will be affected if they do not have enough storage space. How can we improve such challenges?

R: My question is can you bring a solution? For example, we know that the health centers are facing problems. Only a few of them have motorbikes. If you are saying we can bring change working in this area is the best. The health center motorbike will become out of service once it reaches and returns. It is better if they can be supported if there is any means to do this.

I**: What about the staff commitment in terms of timely reporting and filling out the forms?**

R: I have told you this earlier. There might be a problem with data quality. They are filling out the formats. There is no problem with filling the formats that are used in a store. Last mile project providing monthly supportive supervision. They are making the activity improved. So, there is no problem with recording and reporting. The drugs in the units have Bincard. There is no problem to know the amount they received, used and available. The problem is associated with quality.

I: what about management support? Including supportive supervision.

R: there is no problem. If you ask our staff here in the zone, they will respond to you the same way I am responding. When we go to the lower level, this part is included in the checklist. Moreover, there is no complaining about budget shortage for providing supportive supervision. We have committed leaders who can support us.

I: can you tell us the supply n staffing and capacity starting from zone, woreda and health facilities?

R: there is no problem. The activities are connected.

I: what about the number of the staff? How many of you are working here?

R: We are four, it is that enough?

I: Is it also enough at the facility level?

R: no.

I: Is there a regular performance review and recognition?

R: We will meet quarterly to discuss on the performance of the facilities.

I: For your experience, what gaps are there and should be improved in terms of filling out the formats correctly?

R: Pharmacy professionals who get the training may leave the job. It is better if you focus on providing training.

I: Is there any induction or skill transfer activity when new staff is hired?

R: At lower level?

I: Yes

R: Yes, check it. They are doing it. After getting the training. They will provide information about the training they have received to the staff and a unit that is connected to the issue.

I: Is this practiced in all the facilities?

R: we are working to get it practiced in all the facilities.

I: Let's talk about quantification and procurement. What is the process for RMNCH commodities?

R: They requested it using RRF. Quantification is done for procurement. Each health center has done quantification and the procurement will be done based on that.

I: What is your role in quantification?

R: We will inform them to do the quantification. Then send what they requested to [SUPPLYING HUBS]. When then check whether they are getting what they have requested.

I: what is the level of forecast accuracy?

R: You will do forecasting based on the service you provide and your budget. It can be 100%. However, this can be responded by [SUPPLYING HUBS]. Their forecasting may be 100% but they cannot get what they have requested.

I: Is there a need for capacity building on quantification?

R: We don't have a budget for this. E and [region] Health Bureau can provide the training. Our role is facilitation.

I: I have heard as you are providing them a good support. That is why I am asking you what kind of mentorship you provide on the quantification. What gaps are there? Including data quality.

R: regarding the M& E, [region] health bureau can also be a witness. We report the activities of all 120 health centers and 8 hospitals. Gaps may exist. If you ask me about those gaps, when those who get training leave the facility, new professionals who do not know it will be recruited. When such a gap happens, we will call them here and show them how it is done. We will show them how it is filled. This is how they are filling the gaps.

I: What about for quantification?

R: it is the same. We will call them here and show them how the quantification is done. [partner} has supported us on this issue. We developed a proposal including who should take part in the training. They confirmed it and we provided training for two days. If the gap still exists, we make logistic officer at woreda level go to the facility and provide support.

I: What are the challenges associated with procurement?

R: We see the procurement in two ways. The one which is called health care financing and RDF which is provided by [region] health bureau. [region] provides 300,000 birr for each health center for procurement of drugs. These 300,000 birrs will be provided to [SUPPLYING HUBS]. [SUPPLYING HUBS] will distribute drugs that costs 300, 000 birr for each health center based on their quantification. They distribute it every 6 months. Health care financing is the one that uses these 300,000 birrs as revolving. They purchase the drug which is important in the health center every quarter. They purchase it 4 times a year based on their income. [SUPPLYING HUBS] will delivery twice \ year based on their quantification. There is a budget problem. All 120 health centers are provided with 300,000 birr. I think this is only available in [region].

I: Do you think this amount of money is enough?

R: no, it is not enough. Previously it was 180,000. It increased from 180,000 to 300,000 following the drug price increase. It is not enough. even if you make it 500,000.

I: Do they have any other sources?

R: No, they don’t. They use it in addition to health care financing.

I: What should be improved to make quantification and procurement better?

R: It will be good if what they requested is provided to them. They are not getting what they requested. They get 50% - 58% from what they requested to purchase. It is better to work on availing the products they requested not only for [zone], but also nationally. Regarding the quantification, they don’t have to do it to balance the money they are going to receive. It is better if they request drugs which are used in that facility. If they request it based on their consumption, it will not be over or under stock.

I: We were talking about transportation. Is [SUPPLYING HUBS] directly delivering to those over a hundred health facilities?

R: It may or may not reach all of them. If we take [zone] branch, they are also serving the southern region. from [region], they serve [zone], [zone] and [zone]. I think there is also a special zone, next to [zone]. They serve all these zones. Their cars are old. They cannot do 100% of the facilities. They can also tell you this if you ask them.

I: What alternative means of transportation do we have to transport from health center to health post?

R: I have told you this. Unless the woreda gives them the car to transport it, it is difficult. If you ask, they health center what transportation they use to deliver to the health posts, they say “using a motor”. This is practiced everywhere, not only in [zone]. Unless they have special support, it is similar for all the facilities. If you have any alternative means of transportation that you are considering, we can try it.

I: Our focus is on how to improve last mile delivery When we say last mile delivery, it starts with woreda to health post. We are interested to identify bottlenecks and areas that need to

be addressed. Our focus is to strengthen last mile delivery based on the findings of the study. Now we are trying to understand the landscape.

R: The problem related to transportation is from health center to health post. It is better if that is addressed.

I: You mentioned woreda passthrough. Are there any challenges associated with it?

R: There may be a delay in delivering the product. IPLS which does not allow the woreda and zone to keep the drugs. That is why it is called woreda passthrough. Their role is to accept the products and transfer them to the facility. There is no problem other than failing to deliver it timely.

I: Why did that happen?

R: It is due to transportation. There is a maximum of 1 car per woreda. They will deliver the drugs after they complete their preplanned activities.

I: What are the challenges of the implementation of IPLS?

R: IPLS includes recording and reporting. It uses RRF, IFRR, bin card, and stock record card for information flow from lower to higher level. There is no problem with this its implementation and use. The problem happens when people leave the facility.

I: What about the utilization of these formats like IFRR and HPMRR?

R: Initially we were using hardcopy of RRF, IFRR and HPMRR. If you ask me for that hardcopy, it is not available now. We will share the softcopy with woreda and health centers. They will print it and use it out. The health center will print and provide 24 copies to the health posts to be used for the year. Then they fill it, submit 1 copy to health and keep 1 copy for themselves. There is no hardcopy of IFRR in health centers. The health centers have a budget for papers and printing

materials. If you ask me whether it is enough or not, it is not enough. It is better if this issue is addressed.

I: Can you tell us about an emergency order?

R: Emergency orders are not recommended. Unless there is epidemics or the consumption increases, we do not recommend an emergency order. Malaria has created repeated emergency orders this year. Most of the time they are asking for the same drugs again and again as they are not getting on their first request. The problem is due to availability.

I: What about wastage rate?

R: 2% is standard wastage rate. Ours is less than 1.2%. The drug we have at this time is not prone to wastage. (*laugh*), we are facing a shortage. We don’t have wastage now.

I: What are the challenges of implementing the electronic LMIS?

R: The only challenge is the network.

I: how many facilities are using [eLMIS]?

R: it is 90.

I: what about [supplying hubs]?

R: it is 6. [supplying hubs] is not functioning here at this time. Last time we had a review meeting and we were discussing it. They are not giving support. We can say it is not functional at all. They are providing technical support to [district], [district] and [district] health centers following my comments. What we need is to make them work side by side or focus on one of them. We don’t know what ministry decide but, we informed them of the problem.

I**: Whether it is [eLMIS] or [eLMIS], the facilities need infrastructure to implement it. What about electricity and people's behavior? What are the gaps?**

R: So far, we have reported twice using eLMIS. They may face problem to send the report from the facility due to network problems. Now, they are coming to woreda and sending it. To prevent any gaps, we are making them submit both the hardcopy and softcopy. We are also discussing with people from the last mile project on how to proceed. We are discussing on how they can send it offline from where they are.

I: Do the facilities do stack status analysis? What can be done to improve it?

R: Nowadays, they are doing their stock status analysis. As the system calculate and show them, they are knowing their wastage rate. We can say they are in good status.

I: What can be improved?

R: it is better if the offline method is implemented. They can synch it from their health center. They should not come to woreda.

I: How many comprehensive health posts are there?

R: They are around 14.

I: Are they still getting supplies from the health center?

R: Yes.

I: How do you see it from a supply n perspective?

R: they have to be budgeted just like a health center. What they are doing is difficult to differentiate from what is done in health centers. It is better if a separate budget is planned from them like a

health center. They are getting it from the nearest health center and it can affect the resources of that health center. It is better if they have their own budget.

I: What are the challenges related to internal resupply in the facility? What is the interaction between the units and the store?

R: there is schedule at the facility level. For example, they are supposed to have supplies that can be used for a maximum of a month and a minimum of 2 weeks. Max-min of dispensary unit IFRR indicates they have to request every 2 weeks that can be used for a month. You can develop the schedule. there are about 4 dispensary unit in health center. The store man cannot serve four of them on the same day. Requesting based on the schedule is not similar in all facilities.

I: Why are they not asking according to their schedule?

R: for example, if we take the family health unit. They may be attending child birth. It may not be convenient for them to receive the according to the schedule. if you ask at a lower level, they will also give you the same response. They say work overload.

I: What opportunities or obstacles are there to strengthen health post resupply?

R: The obstacles are related to unavailability of the drug they request. The drugs delivered from [SUPPLYING HUBS] might not be enough. For instance, they may request 100 Depo, and not get it. One health center will provide supplies to 5 health posts. But if the health center is not getting enough numbers from here, it will be difficult to provide to the health posts.

I: Are the health centers doing supportive supervision?

R: Health centers have their own program of giving support to health posts. A health professional is assigned to the health center to provide support. They use a checklist during their visit to give feedback on their strength and weakness. But it is not 100% similar for all the facilities.

I: Are supply n data used for decision making?

R: We are using it for decision making. We are not just reporting it. For instance, I will CC all pharmaceutical related data to all responsible bodies. We then discussed on identifying gaps and how to improve them.

I: What platforms do you use?

R: We have a platform lower level for [region] health bureau.

I: How do you prevent stockout and wastage?

R: There is a direction from [region] health bureau that was circulated in 2009. It indicated exchange of drugs that are overstock in a facility. We are preventing stockout this way. If the drug is overstock in one health center and that drug is required for hospital, they will make a formal transfer using a model. We are using this method to reduce our stockout. This will prevent wastage rate

I: Any best practices that can be scaled to other areas?

R: This can also be considered as best practice. If you ask [region] Health Bureau, they will tell you how they managed expired drugs in 2013. Other than this, the communication we have with our leaders and the way they treat us is very good. Other zones complain about the budget. There is no such thing in our zone. This is one important thing. Our staff's communication is very good. other staff can tell you more than 75 % of what I am telling you now.

I: Thank you very much. Thank you for your time and contribution.

End of interview

KII Transcript (8)

**Name of Project:**	[project]
**ID Code**	KII-P-20
**Type of interview**	KII
**Type of informant**	[sex}
**Region**	
**Role of the Interviewee**	Technical advisor
**Years of experience**	
**Name of Transcriber:**	[lecturer]
**Date of Transcription**	14th August 2024
**Length of interview**	Start time: 09:06 End time: 11:02 AM
**Names of Reviewers:**	

Start of interview

I: How do you perceive the overall effectiveness of the supply n management system?

R: You mean specific to RMNCH?

I: let us first discuss the overall supply n management and come to RMNCH.

R: This is an interesting question which is very wide. I can tell you from my experience when I was working in sub-national [RESUPPLYING HUB] hub. Once supplies are purchased, they will be delivered to the central [SUPPLYING HUB] hub. Then products will be distributed to last mile through19 hubs in the country. The commodities will reach the health facilities through the IPLS. The commodities reach the health facility in two ways. The first one is direct delivery by [SUPPLYING HUB] to a health facility and this has reached around 65%. Woreda passthrough, where the [SUPPLYING HUB] deliver it to woreda and woreda will deliver it to the facilities is also in place. Those with low volume will get it through the established system at a lower level. The health centers will take it from woredas and the health posts will get it from the health centers. As we see overall supply n management, it is progressing over time in terms of increasing direct delivery, system strengthening and capacity building through partner support. When it comes to national level, previously all issue of supply n have been implemented by [SUPPLYING HUB]. However, the ministry is now working more on supply n management and working on improving the availability of the products. The linkage with health facilities is also being monitored through

the ministry. The ministry is also working closely on filling the gap in the availability of the products by engaging the regions. Health commodity program management protocol is developed at the national level. Based on that, there is a platform that the ministry leads every two months. That means there is a meeting which is conducted every 2 months by engaging sectors. There is also a meeting twice a year by engaging the regional leaders. That means the ministry that control overall system can see the existing problem and take action. If we see the financial perspective, the government has reached an agreement with different donors and now the problem with commodities of maternal and child are in better status. Overall supply n management up to last mile delivery is in good status. I am not saying there are no challenges. We may discuss on the challenges later.

I: What about facility readiness in reporting, data quality and timely delivery of commodities?

R: As I have said, there are productive things with budgeting and communication with [SUPPLYING HUB] at the national level. Still gaps are being observed at the health facility level. regular facility level supportive supervision every quarter. We reach 359 health facility. We observed their inventory management and implementation of IPLS and how they are using the data. We will check their reporting data. From my previous experience I have seen big problems with data quality. Data quality is very important to deliver a product from a center to a health facility. They should be supplied based on what they need and the quantity of what they need. IPLS has been implemented to facilitate this process. They use RRF to request products. For that they need to record the data on bincard, recording the transactions correctly and data of products used within the facility. I believe there is a gap on this issue. If they are not recording the data continuously, there will be poor data quality. The RRF that is sent to [SUPPLYING HUB] is of poor quality most of the time. It shows gap in data quality. It might be due to negligence and lack of commitment. To get supply management to the level we expect, we need to automate it. there are activities that the Ministry and partners called are doing. I recently get information as they implemented [eLMIS] to 5200 health facilities. However, there are still issues on strengthening it and the health facilities commitment gap in implementing it. Of course, more than 80 % of health facilities are reporting if we check in at [SUPPLYING HUB] hubs. They are not getting

commodities if they are not reporting, this have created some improvements. Reporting from the facility to [SUPPLYING HUB] is in good status. But is that the data is right one is the basic question. We are happy if the commodities reach the community. RRFs that will be printed out of automated facilities are also not a correct one as they are not registering the transaction correctly. This exposes them to stockouts and false stockouts. Facility that really does not need the commodity pools it from the center. If you have national visibility, you will see the product that is stocked out in some facilities to be overstocked in other facility. Data quality plays a key role in artificial stockout. A lot of things have to be done on reporting at the health post level. the link between the health center and the health post are very week. When we come to hospital level, the data recording and reporting is better.

I: What challenges have you encountered in ensuring timely and efficient delivery of health products to the last mile?

R: When we say last mile, it is the level at which the community gets the service. We can see in different thematic areas. The first one is related to logistics. As I have said earlier, we have around 4000 health facilities (including hospitals and health centers) and more than 17000 health posts. If we take commodities of family planning, maternal and child and malaria, the nearest place for this health post. If we accept these services that can be provided at the health post level, these commodities should be available to the health posts. Logistics is the main reason these commodities are not reaching there to be used by the community. [SUPPLYING HUB] is delivering directly to only 65% of all these health facilities. That means 35% of them are getting as woreda pass through. These is huge number and they have lack of logistics to move the products from place to place. although the products reach woreda according to schedule after sending RRF, there is a situation when the facilities are becoming stock out due to lack of transportation to reach the facilities. Supply capacity at the national level should not be only 65%. We have 19 [SUPPLYING HUB] hubs and their infrastructure to carry products is good. We also think there is shortage logistics at the national level. So, efficient logistic management is very crucial. It is not enough to give the responsibility to the health facility to receive it from woreda as they have logistic problems. Not only logistic problem they also have financial problems to manage it. Financial capacity of the facilities is one of the challenges. The facilities are also not coming and

taking due to lack of commitment. For instance, the health posts are under the health centers that are nearest to them and they come to the health center for different reasons. They can use motors and other means of transportation, but due to lack of commitment they are not using it. unless there is accountability and responsibility, the warehouse manager at woreda and health center are not delivering the products that is available. The staff should use any means to deliver those products to the facility. HR is one of the challenges for last mile delivery. It might be due to shortage of manpower, commitment, or lack of continuous capacity building activity. Last mile delivery will only be achieved if supply n is strengthened as it is all about logistics. If we take RMNCH services are continuously supervised at all levels then we should bring this to the supply . There is a practice of monitoring, evaluating and addressing the challenges of the supply . The other important thing is the data quality that we have discussed earlier. Last mile delivery will be affected even though the logistic issue is resolved if the data quality is poor.

I: **Can you describe the current quantification and procurement process for RMNCH commodities?**

R: RMNCH quantification is done at national level every year. The ministry has established a quantification period. We have a longer lead time as a country. To request based on the lead time, the quantification should be done appropriately. The data for quantification is coming from a lower level. Different stakeholders who support the procurement also engage in quantification process. The quantification process starts by establishing task forces and big partners working on the RMNCH will be included. For example, , , , and were stakeholders. These partners are working by supporting people from lower to top level. The taskforce discusses on what is the demand of RMNCH, checking data and assumptions and financial capacity. Then data will be collected, analyzed and a decision will be made. [SUPPLYING HUB] is also another important stakeholder. I think region level stakeholders have been taking part previously. But, currently Ministry, partners at the national level and [SUPPLYING HUB] are engaged in quantification. Once quantification is done, it is expected to be disseminated. Quantification is done for done for three years and we forecast it for three years. The one we did this year will be used until 2019. But it will be checked if there is any change on target. So, quantification of three years that will be revised every year will be done. Budget plan of the first year of quantification will be done during the process.

I: Is quantification done at a lower level?

R: Quantification is done at the national level. previously region level supply n managers and RMNCH officers involve to provide region level service data and consumption. For quantification service data and consumption data are provided from a lower level and its aggregate is available at a national level. the quantification started to be done at national level through time, following availability DHIS 2 data and [SUPPLYING HUB] data. But at region level they identify their target and plan the number they need. Since the service data is already available at national level, the demand request is not coming from lower level. there is also a situation when the referral hospitals engage in maternal and child health quantification.

I: What about forecasting accuracy?

R: I haven't seen data as it is done by [SUPPLYING HUB]. But they were in the range of better forecasting, which is around 75% after the budget issues are getting solved. The performance of health facilities is better nowadays. If we take family planning commodities, their forecasting accuracy is more 80%.

I: Can you tell us about quantification of RDF commodities? What challenges are there?

R: [SUPPLYING HUB] leads the quantification of RDF commodities. The demand is requested by the health facilities based on their budget capacity. The demand comes from the facilities to reduce wastage. There is a standard tool on which the facility does their quantification. RDF also has its own quantification period. It is from September to November. The facilities inform their needs to their hubs by filling out that form. The [SUPPLYING HUB] hub will reconcile it with the one they have been selling to those facilities. It is not possible to do quantification or at all health facilities as it is time taking and need budget for capacity building. While it is believed the high-volume data can indicate the national need, sampling has been in practice for years. However, a ministry is engaging in RDF quantification with 30 hospitals, including federal hospitals and university hospitals, by providing capacity building training. We expect data quality for

quantification will be improved overtime. [SUPPLYING HUB] is complaining about high forecasting errors due to taking sample data from facilities. The [SUPPLYING HUB] done the quantification for the year 2017 based on data available at the national level. So far the practice is at hub and center level. now it is about to be brought to centralization from facility level quantification. Of course, 60% RDF commodity is for tertiary and general hospitals. It is becoming centralized considering them as the main consumer and using assumptions for the remaining facilities. I hope it will be better if they use different forecasting models. [SUPPLYING HUB] is also mentioning it as they are going to centralize it. The big challenge for forecasting is the data. If we take the family planning services provided and the consumptions, there is a big difference in data. Demography data is also outdated as the census is done long time ago. There is data accuracy problem. If we take the malaria data, the one on DHIS2 and the one reported through PHEM are different. This was a very big challenge for forecasting. Once we have national data visibility, we have an opportunity to see who used what and wastage rates. We will not depend only on the data from the hub. Professionals' commitment on data quality is a very important contributing factor. I also think that quantification tool should be developed. There is a tool called CAT that is available from . TB, HIV and Malaria commodities are being done using this tool. There is also a tool called QualTB. However, these tools are still managed by partners. The challenge is not only being managed by partner. Are we in agreement to use this tool as a country? Anyone at ministry level should be able to use these tools for quantification. There is no trend of using the data from such tool so far. You have to monitor the pipeline supply. We monitor pipeline supply and stock status using excel. We are not using the tool to see the status by entering the data. We have too much data, but we don't have a tool that can show us even supply n risk. We may use it for quantification but we need a tool that can show as the risk of stocking out of certain products. Once we do the quantification, we will have to monitor the pipeline supply. I think this is one of the challenges for the supply . It will be very difficult if we are still waiting for RDF quantification from facilities since there is data issue. Secondly, unless commodities are in full supply, you are increasing your quantification error if you are doing it based on their consumption. Failing to use different forecasting models that are used in other countries for RDF quantification is another problem. We may not assess the market status as the market is both public and private sectors. Not including private sectors for RDF commodity is one of the reasons for some drug expiration. So, marketing issues can also be a challenge for quantification.

I: What is the capacity of transportation and distribution of commodities to lower level?

R: As a country the big organization that delivers pharmaceutical supply is [SUPPLYING HUB]. I don't think we face any challenge to import the commodities. As long as you pay for logistics anyone can bring to your door. However, I can say there is transportation capacity problem to delivery at lower level after it arrives in country supply n, not only RMNCH, the health facility can only receive RDF drugs from [SUPPLYING HUB] if they have capacity. Transportation is the biggest cost and the government should manage to deliver it. that is why [SUPPLYING HUB] is taking the responsibility to deliver. Still there are facilities that it is not reaching. This problem boldly seen to deliver it from health center to health post. There is a transportation problem for those who get it through woreda pass through. A woreda may have one car that is supposed to be used by the woreda administration. There might be more motors and they are not enough. They use them for different outreach activities in addition to delivering the commodities. Only a few facilities are using motor to delivery it. most of the time health extensions themselves come and take. This will be very difficult when the products are in bulk. Road accessibility is another challenge for transportation. The challenge is higher as we go lower level. We need to think about how to reach the community level after the health center should be considered as a system. Since the implementation of last mile delivery, [SUPPLYING HUB] hub has started reaching health centers every month as a pilot. Storage capacity is another issue for these facilities. Distance is also another problem. I think we have to use different approaches for different facilities. It may not be equal to deliver to tertiary or general hospital or health center every 2 months. Not considering the facilities based on their volume is affecting us from reaching the last mile. If we make it every month it will shorten the 65% direct delivery at the national level. transportation is the main challenge to delivery from health center to the health post. You raise data issues when you start considering quality.

I: what can be specific challenges related to woreda pass through?

R: logistic issue is the main problem when the commodity is delivered as woreda passthrough. There is also a situation when the facilities are not capacitated on the data recording and reporting.

Woredas are filling the RRF of the health facilities and they are not giving ownership to the health facilities. I think this will affect them indirectly. Logistic issue which hinders them getting the product are the main challenges. This can be due to woreda transportation and financial capacity. This is preventing the health facility get the commodities every 2 months, even if it is prone wastage. There are products that are important at health facility but being expired in woreda stores as a result of failing to transport them. The facilities that are included in woreda pass through are those found at remote area and low volume. They are not getting capacity building and supportive supervision.

I: What alternative means of transportation can be used to deliver it to lower level?

R: I think they are using human power and motor to deliver to health posts. We may not say assigning a car as it cannot be efficient. It is better if they store manager can use the motor and deliver it. Additionally, as the volume of the commodity is one factor that prevent to use manpower, the resupply time can be reduced if the health post is not very far away. As the health post is considered as dispensing unit of the health center, responsibility is given to the health center. I think there will be better delivery if the woredas also help the health centers by creating a network to deliver the products.

I: What do you mean by creating a network?

R: there are health posts that are closer to woreda than the health center. So, these health posts can get the commodity directly from woreda after linking them to the woreda. Delivering is all about transport and transportation cost. In addition to that woreda can take the commodities from health center and deliver it the health posts if the health has no capacity to do so. There are an average of 5 health posts under 1 health center and they may not have the resources to reach these facilities. But woredas have better man power compared to health centers.

I: How do you see the capacity of [SUPPLYING HUB]? You told us the capacity of the facilities. What about [SUPPLYING HUB] in terms of route optimization and third-party logistics?

R: Assessment was once done to check the capacity of [SUPPLYING HUB] and to provide support on the identified issue and how to strengthen in country supply transportation. Delivery is all about transportation and availing cars can address the problem of transportation. If it is air transport [SUPPLYING HUB] uses its own cars to transport from the airport to the warehouse. Otherwise, it outsources the transportation from parts as it is container based. These hubs which are in a challenge due to shortage of vehicles and they are not able to increase their direct delivery. There are many vehicles at the center as they are supposed to reach 18 hubs every month. We have suggested to outsource the transportation from center to hubs out sourced just like the one which is brought from a port. There are many van cars that are transporting form custom to the warehouse at the center. This transportation can be out sourced and those cars will go to the hubs to transport to lower level. through this last mile delivery can be improved. So, far we have seen the transportation capacity of the government, I think it is possible to try third party logistics after subnational transportation. It was once tried to use it, after taking the experience of Coca-Cola. I have seen [supplying hub] has 3 or 4 cars. As you know Region] is scattered and a car can travel 1000 km and return. This shows as we have to think of other means for optimization of the logistics. I think it is better to outsource some of the facilities considering the geographic location and volume. We cannot make it fully outsourced, but [SUPPLYING HUB] can consider 2 ways to reach the health center level. Regarding route optimization, it is questionable to use available vehicles efficiently. There is practice of using even and odd. But, this can be optimized by using technology. Automation of the route can increase route optimization. If health facility improves their capacity to use health care financing, they may strengthen woreda passthrough delivery. It is huge capital to buy and provide a car. But it is possible to work by outsourcing this service with enough amount of money.

I: Is there anything that you think are remaining to be addressed?

R: RMNCH Once the products are out of store pharmacy role is over. The role goes to service delivery. Failing to engage supply n officer in the status of service delivery has created less commitment for supply n management. Once the commodities reached the store, the remaining role is to issue those commodities. It needs to check how the commodities are being managed at

health post level. If you ask a supply n officer how are family planning commodities are used and which commodities are preferred by the community, he do not know it. I think not engaging the supply professionals in the utilization of family planning commodity is one of the challenges. I think it is good if we are able to make the supply n professionals engage in service provision area. It may need to check practice of other country. It may need some desk review. Other important thing is, although woreda networking is for commodity delivery, the link with health post is in dangerous situation. Health posts are not recording the commodities and not requesting on time. the store manager is giving them without HPMRR is being filled. Linkage between the health center and health post is very weak. The linkage between service unit and pharmacy should not be only issuing supply, they should also check service delivery. The logistics is separated from service. If we take malaria, consumption is filled on RRF and service delivery data is available. They are not showing the same figure. The logistic management information system is detached from service delivery. We need to work on this issue. Triangulation of logistic data and service data is important.

I: what are the main challenges of implementing IPLS?

R: as I have said earlier, it is on the logistic management information system and inventory management of warehouse. Capacity building of HR and monitoring and evaluation. M&E is very crucial thing. Now KPI of Pharmaceutical services and supply are included in the M&E at national level. unless those KPI are monitored at zonal, regional and national level, providing training alone cannot bring change. As long as the professional is providing service, he is responsible to fill out the bin card, fill out the RRF and request products on time. At the end, to say IPLS is fully functional it should increase the visibility of the data. We cannot bring this data visibility by doing on paper. There is automated system, but it should be monitored for visibility. Although there is integration of online systems, there is no data visibility. Even [SUPPLYING HUB] see the performance of the hubs only on dashboard. Now there is ERP and you can see the activities from the system. They are also using [eLMIS]. The facilities that are using [eLMIS] are at least observing their stock. It will be better if the [SUPPLYING HUB] can receive order from the health facilities through this system. Lack of interaction between the system is also another problem. The status of this systems should be monitored at health facility level. I feel this activity is reduced.

There should continuously support at health facility level. The supply n at ministry level focus more on procurement. There is no strong connection with regions. If you ask how the regions are requesting for commodities and buying it. I think strengthening the link with the regions and regions with zones will improve the implementation of IPLS and LMIS.

I: What ais you experience with utilization of formats and tools, emergency order request? What are the challenges with poor utilization of LMIS tool? What is the reason if they are frequently requesting emergency order?

R: RRF, IFRR and HPMRR are the tools we use in LMIS. We also use bincard for data recording. RRF is utilized very well as the facility are cannot get the commodity if they are not submitting it. IFRR and HPMRR are not in good status unless those which are regularly supported by partners and regions. We have seen while they are using white paper. Even there are facilities which provide it without any form. Most of the time for resupply is not the same with in the facility. All the unit may not request routinely every 15 days just like dispensary unit. As the form is a little bit longer, people may become bored. There may be lack of commitment. As it has repetition of things people become bored. When we come to availability of the formats, previously preprint formats had been provided through support from partners. Recent version is printed in small number. They are expected to print and use it as they have the soft copy. Since it has many resources issue, the practice of printing IFRR format is not common. Now a days gaps are observed on utilization of the formats. They are not printing and providing HPMRR to the health post. They are using white paper to request it as there is resource limitation. Availability of HPMRR is one of the challenges. Availability of Bin card is good. The problem is not updating it regularly. The root cause might be knowledge and skill gap of using this format. Nonexistence of commitment and continuous follow up is another main problem. If someone is not accountable for not updating bin card, request using quality RRF, why should they fill it? It may also due to work load, especially store managers are becoming busy in high volume hospitals. I think this are the root cause. Although automation can make things easy, every transaction should be recorded. They are not recorded due to work load. There high transaction in high volume hospitals like tertiary hospitals. They also think the recording is time taking. None existence of enforcement from facility management is also another problem. The health center management should enforce if dispensing unit is not using IFRR. Weak

management and lack of enforcement at facility level is a problem. Store man says they are busy but this happens as they fail to provide it based on the scheduling of each unit. As there is no schedule, there is frequent resupply within the store. Or as they are not using the format correctly, they do not know their demand. Due to frequent request, the store man become busy and they spent most of their time with routine activities and not able to monitor other activity. The reason for poor RRF and recording data is frequent stockout. This shortage also happens as result of poor data quality and unavailability of recording tool. This led to frequent request from [SUPPLYING HUB] and resupply of emergency request. In addition to that, stock shortage is another reason. If they are not getting what they need more, they will be forced to emergency order. The main reason for all these thing is data quality and lack of regular reporting and resupply request.

I: **You have raised issues like interoperability problems of electronic LMIS. What are main challenges of its implementation with regard to infrastructure, functionality and data accuracy. Is there support mechanism?**

R: Functionality issue may need its own assessment. Electronics issue needs daily engagement as they are working on other thing. It needs to be experienced on that issue. I don't know if it works offline or not, the main problem at facility level is network issue. Facility may not take responsibility of installing a network. Failing to pay for the network routinely and not fixing when there is a problem makes the system interruption. At that time recording of the transaction will be missed and affect its functionality. This will increase poor data generated from the system. The system may also fail and they should be fixed immediately. Support to fix the system should be available at nearest. These are challenges that some facilities raise on the system. Presence different system that are not integrated is also a challenge. For example, there is [eLMIS] for warehouse monitoring and inventory, [name of mobile app] is used for vaccine, and some selected KPI are also available from DHIS2. There should be national level logistic tracking system from lower level to top. At lower level there is a system that health posts register logistic data. Using different system has created challenges. Interoperability will be difficult when there many systems. Visibility should be improved at each level. the data that is filled at health center level should be accessed by the woreda. To say the RRF is correct or not you have to see it. We may see it the dashboard at national level. management body is responsible to review the RRF and Bid card filled

at facility and they have to access it. this things can be challenges to our electronic LMIS improvement.

I: what is the status of inventory management system at woreda and facility level? do they do stock status analysis?

R: High level hospitals and [RESUPPLYING HUB] are doing better in doing inventory management. There is practice of checking their stocks and consumption. There is practice of transfer and exchange of over stock and near expiry drugs. There is transfer guideline. Using this guideline to transfer over stock and near expire is better at high volume hospitals. Not only to prevent wastage but facility with stock out can maintain the stock by transferring from nearby facility. Still there is challenge at lower tier of facility to do stock status analysis and make decision. If you are not updating your bin card or using automated system that show you your status, you will face problem to monitor your status. You need automated this system to do stock status analysis. So, inventory management system at lower level is very poor and need to work on it. That is why emergency order is high. If we do stock status analysis, we will know or risk of stock out and risk of expire. Inventory management stars from warehouse management and weak activity there also affects your inventory management. Skill and knowledge needed to do stock analysis. I don’t know how we can do it but, I think we need to have a tool which can be used to do stock status analysis in addition to RRF and Bincard. [eLMIS] has stock status page. It shows you minimum and maximum. Within minimum and maximum it shows this this are at risk of expire. We do have such system. Does the facility have AMC, average monthly consumption? You do inventory if you have a plan on your consumption. Of course, RRF has the AMC and it can support you to calculate your monthly consumption. When we come to RDF, each facility should have AMC for each product and monitor it continuously. Each facility needs to have a system that can be used to enter in their computer in addition to RRF. As long as we increase implementation of automated system, our inventory management system will be improved. Paper based activity will be challenging for inventory management. You know the commodity after they get expired.

I: What is the understanding of health extension workers to identify stock out triggering factors?

R: I think IPLS training has been given to health extension workers. It is not available now. The only training the health extension workers can receive on logistics is through ICCM. I am not sure whether these people really know IPLS. I don't think they are well informed on how to do inventory of their supplies. I don't think there is capacity to build existing among the health extension workers, other than ICCM training. I don't think they have knowledge of assessing the risk of stocking out or what triggers for stocking out. That is what I have been saying earlier. To what extent is the link between the health center and health post? Is the health center pharmacy professional even visiting the health post? The status of commodity that is near expire should be monitored. If we are not going to the level of monitoring of health post status, with the current health extension workers supply n knowledge, the chance of getting expired commodities is high. It depends on the capacity of the pharmacy profession at the health facility.

I: What are my opportunities to improve the health post resupply?

R: Previously two health extension workers were assigned to health post. When one of them provides a service, the other one goes for outreach. Now the health posts HR is being capacitated due to different reforms. Accountable people can be assigned to the supply n when they have enough staff. I think new system is assigning midwife and nurse and someone who should take responsibility of logistics should be considered. If it is not possible to assign a supply n professional, one health extension worker should be assigned and take responsibility of the supply . It is possible to establish accountable person who get logistic training. Another opportunity is that there is a mobile application that they use during outreach activities. The commodity they are supposed to hold is limited and can be integrated into those applications. Linking the health centers that have electronic logistic management information system with the health post, it will also improve logistic management at health posts. Coordination problems are seen within the facility most of the time. Postpartum IUCD is a new project that is going to be implemented in the facilities. Trained manpower is only available in a few facilities. Trained maternal health professionals and the commodity is provided to the facility but no one is bringing this to action. The store man considered IUCD and PP IUCD similar. Most of the time logistic personnel are not invited for training of new initiative. Of course, IPLS is a system used for logistic management. The logistics training is not focused on linking people from the program side and supply . Since you are not connected during this time, there is information gaps. Training on commodity

management of programs is needed to be implemented. Designing such platforms is important to fill program specific information and knowledge gaps.

I: Can you tell us more about generating and using data for decision making at a lower level?

R: I think such a trend is available at big hospitals and facilities that are automated. The budget is limited now a days. Wastage management is also coming as a KPI indicator. Facilities are expected to report wastage rates. Due to this improvement is observed in terms of doing analysis in tertiary and university hospitals. Other than during inventory time or emergency request, it not common to do what they have at hand. Quantification is one area that increases efficient data use. A facility needs the data to show its need. As we go to a lower level, using data for decision making is very poor. It needs data analysis skills and knowledge. You may need a simple excel tool. It needs computer. How many of the facilities have functional computer in pharmacy unit. It is difficult to ask them to work on paper at this time. unavailability of infrastructure and skill gap are main challenge to use data at a lower level. I feel tertiary and secondary hospitals are using data for decision making. I don’t think it is enough. We don’t need to do things centrally if the data is used at a lower level. Quantification is being done centrally considering the human resource, tools and other capacities.

I: What should be done to overcome this problem?

R: It can be capacity building. We may not support every facility directly. In-service training can be provided as majority of things can be skill gab. Now CPD is a good opportunity. It is making mandatory for everyone to have continuous professional development. If the professional development is mandatory, people start to get knowledge and new skills. We can include logistic issue in this professional development through in-service training after identifying areas that need to be improved. We may use an online or on-site approach to help professionals improve their skills. This will also increase their commitment. It is also better if the tools are simplified to help people exercise it easily. Recognition is also important. We can give recognition to health facility that has better performance compared to others.

I: What about functionality of PMT at the facility level? what is the role of pharmacy in the team?

R: I am not sure about it. It will be a big opportunity. I think performance management team is to evaluate KPI of the facility. Its role is to give feedback and present the gaps to the management. They have to be on this team. I believe they are member of the team at the hospital level. the point is how committed that person to show the problem they are facing to the team. It is also one mechanism of creating accountability.

I: What do supply n staffing, recruitment and deployment look like? are they enough?

R: deployment at a higher level is better in terms of knowledge and skill. People say pharmacists don't go to rural areas. As we go far from the center, the chance to get competent pharmacist decreases. I feel we have a gap in induction training as a country. Once, there was an interesting initiative that [SUPPLYING HUB] started on competency framework. Newly recruited professional will be assessed for competence before induction training. When we go to the lower level, there is shortage pharmacy professional. If you go to health center there might be one pharmacy professional working at dispensary unit and another health professional managing the store in addition to their other roles or shifting them to work in a store. This can be a reason for poor data quality, commitment issue that we were talking about earlier. If other professional assigned to it specially in facility that implemented [eLMIS], they system will automatically stop and they will start to use manual recording as they were not exposed before. We don't have HR framework to identify who needs what, and competency training is not in place. there is no trend of identifying the knowledge and skill gap of professionals and providing capacity building training as a country. Newly recruited professionals will learn how to work through trial and error. No one is telling you this is your job and you are supposed to do this. In the future, HR can make other to do skill transfer before they get clearance from a facility. It is better if there is a system which allows providing short induction for newly recruited. It took me 3 months to become experienced on contract as no one can give you training. How many documents should you read? Of course, there are too many activities.

I: Can you provide Us some additional recommendation that can be used to improve supply n management in the last mile?

R: There are things that we have been discussing to improve the capacity of HR. We also raised about standardizing the tools. This includes making these tools interoperable and making

consistent tool throughout the levels. Regarding what we have said on inventory management, we will not bring automation overnight. We have less than 50% of automation. We can start using excel based inventory management activity in facility that are using paper based. We can also support them to have AMC help them see their wastage rate. It is good if we are able to add such an easy tool. When we talk about tools, it is all about data visibility at national level. it should make the individual at the level closer to data. We can help people at a lower level on how they can at least use tracer drug data from DHIS2. It brings issue data and consumption data like wastage rate. Continued monitoring, supportive supervision and management enforcement are key for inventory management and LMIS. The thing about LMIS is the data and it should be done with responsibility and accountability. This requires management enforcement. We can solve the data quality issues through monitoring and evaluation. Strengthening linkages between health posts and health centers is important. There should be a platform where the pharmacy professional and service providers can discuss. Regional supply n forum has been established in 14 regions. The regional health bureau head and his vice this forum. They also engage with high volume hospitals, [SUPPLYING HUB] and partners. On this platform, the partners present the challenges and best practices they observed at the health facility level. This platform brings together all responsible bodies to sit and discuss. Such platforms can be organized at the health facility level by the management body. Of course, there is DTC, but it sees more of the pharmaceutical issues. In addition to strengthening this DTC, the management body can work on creating such a platform where the logistics officers present the challenges they are experiencing and what they need to improve it. I think such things can bring better communication and coordination.

I**: Thank you so much for your time.**

End of interview

Transcript (9)

**Name of Project:**	[project]
**ID Code**	KII_P-21
**Type of interview**	KII
**Type of informant**	[sex]
**Region**	[region]

**Role of the Interviewee**	Technical advisor
**Years of experience**	16
**Name of Transcriber:**	[university Lecturer]
**Date of Transcription**	August 2024
**Length of interview**	Start time: 02:10 End time: 04:01
**Names of Reviewers:**	

Start of interview

I: Could you describe your role and responsibilities within the health supply chain system?

R: I have more than 16 years of work experience in supply chain management. I started working at [Hospital]. I Worked for 7 years as distribution coordinator at [RESUPPLYING HUB]. I also worked as supply chain advisor for [RESUPPLYING HUB]. I joined [implementing partner] as supply chain coordinator. Additionally, [implementing partner] was a last mile project. There are pilot projects in [supplying hub] and around [supplying hub]. We are working on integrating direct delivery of vaccine, Family planning, RMNCH to health centers and hospitals. We support 2 hubs, 4 zones and 323 facilities. I am engaging in all the activities of this project. I coordinate the deliveries at the national level. I also coordinate COVID 19 supply chain activities.

I: How do you evaluate the supply chain management in the country?

R: the structure and skeleton it is organized on a national level is very good. We important 80% and above of the supply. The structure and the policy are good. However, the supply chain function should be improved. Due to inbound where more than 80% is imported, the supply chain leadership should be strengthened. Suppliers should sign a long-term contractual agreement with the government. Information flow from facilities, [RESUPPLYING HUB] and Ministry is very weak. Its visibilities are also low. The facilities data quality its utilization, capacity building status are unclear and soft activities remain. Service integration and product integration is pro[implementing partner]able activity. Availing products that are used in specific facilities by integrating them is very pro[implementing partner]able. We have to think of the cost and delivery. I believe any things are remaining on capacity building, monitoring and evaluation area and technical support.

I: How about the data quality and facilities readiness to report it?

R: there are many bottle necks from the facility side. For 323 facilities we are supporting, we have 100% successful facilities in [supplying hub] area. We are facing challenges in [supplying hub]. There are many challenges from both the facilities and [RESUPPLYING HUB] side. Challenges from the facility side include report quality. There is a problem from the facility side in terms of sending the correct data of what they need when they are going to use it. there is problem of matching consumption data with the provided service. From the infrastructure side there are shortage of fridge…. When we go for supportive supervision in pilot sites, there is a facility where fridge is only available at EPI units, including hospitals. It will not be possible to ask them to have products for two months keeping the cold chain. Strengthening facilities cold chain system is important. There is shortage and gap in continuously maintaining it. The is also about capacity building and spare parts issues. Supports are need around cold chain management. [RESUPPLYING HUB] has also infrastructure issue to deliver products according to plan. We need to have small pickups that can be delivered hard to reach the area. Private sector linkages are also important. They may not have enough vehicles that reach all facilities. By engaging private sectors, we can reach facilities that are difficult to deliver. These kinds of platforms are infant or not available at all. Security issues are also another problem. Transportation is another issue. For instance, transporting for more tan72 hour drug that needs a fridge is a problem. It needs to avail convenient transportation vehicles. Inventory management is also another issue. Cold chain management, manpower capacity building and strategy to motivate manpower is important. Demand management is the problem that exist starting form top level. For instance, data on the number of Oxytocin needs should come from a lower level. Since the data from the lower level are not used properly, demand at different levels will not be addressed. We are not working on the community engagement. You need to engage communities who get service at health center and hospital. If community engagement is in place they will protect it when there is a security issue. I believe we can provide better service if we work on these things.

I: What about the workforce? Is that enough?

R: There was an initiative to implement IPLS. It started in 2010. It provides training to many health professionals. It will get stuck if those who get the training stop working in the facilities. There might be a change in activity and turnover in the facilities. Workforce capacity building must be provided continuously. We must work in a workforce capacity just like we do for products. If we work on capacity building of work force at facility, woreda and zone level, all the activities done in these areas will be effective. For example, you may give orientation to the leaders or refreshment training to the professionals. It can be better if contextual specific training is provided. There might be no enough manpower at each department. However, capacity building can be done by providing short term or on-site training. We cannot say workforce capacity is full with the current situation.

I: From your experience, is there performance monitoring and recognition based on performance?

R: recognition mechanism is fluctuating. Supporting partners may sometimes do the recognition activity. There were partners who were giving recognition to the facilities and individuals who were performing better. I don't know about its current status. It should be in place. Regarding performance evaluation, they are becoming motivated when we provide training to the area where we implement our project. It is better if such initiatives are implemented. Recognition can be given to the facility which performed better. Individuals from that facility can be selected and given an opportunity to upgrade their education or their career can be promoted. I feel such a system should be implemented.

I: Can you say something on induction, skill transfer and mentorship activities?

R: There are induction and skill transfer guidelines. The problem is with its implementation. For instance, a certain facility, woreda or zone can make someone who is leaving that organization show each activity before leaving. Unless that leader is committed, they may not do that. There is no strong system for this. We were in [zone] and [zone] 2 weeks ago. Skill transfer is not practiced in all site we have visited in [zone]. As the logistic officer in [zone] is strong, he capacitated all the woredas and facilities in such a way. So, no one can get clearance unless they show each activity and handover things properly. Such a thing should be strengthened. It should be part of the annual report for facilities, districts, zones and regions. There should be a report showing this number of individuals left and this number of them hired and this number of inductions and skill transfers provided. There is a gap in implementation is and follow up. Regarding mentorship, there

a document developed at the national level. We are providing training on mentorship to woredas that are in our pilot project. The logistic officer in woreda should then do it continuously. It should not be limited to the pilot stage. Best practice in this pilot area should be scaled to other places by the government.

I: Can you describe the current quantification and procurement process for RMNCH commodities. It can be a program and RDF commodity.

R: RMNCH products should be fully available. Quantification and supply of program commodities are done at national level. This has both advantage and limitation. However, there is a problem with the data quality that the facility sends and their commitment. Doing the quantification at the national level is good. However, objective input data should come from a lower level.

I: What about the RDF?

R: They are trying it. there are few hospitals that take the country's consumption. There is a commitment to do quantification these hospitals and avail for them. However, there is a fear from [RESUPPLYING HUB] and the facility side when you enter into its implementation. The facilities demand to have the drug by any means once they depose their money to [RESUPPLYING HUB]. They are right. [RESUPPLYING HUB] imports 80% of the commodities, and they have no ground to ask for additional money during this fluctuating market. Regarding forecasting of RDF, a national quantification exercise is in place. There are data that come from hubs, regions and zones. Then the quantification will be done. [RESUPPLYING HUB] uses its own data. They have their own consumption trends. They check the last time consumption trend and add some contingency before purchasing. It has no clear process. The facilities are just putting big number. They don't have that budget if [RESUPPLYING HUB] buys it for them. With the current situation, it is not possible to make the health facility do the quantification. Although the have a need, the facilities do not have a budget. Additionally, the quantification they are bringing is not satisfactory. For RDF, whether it is at national or regional level, quantification can be done and distributed to the facilities. Currently regions like [region] and Amhara are allocating budgets for RDF. It started with 180,000. Now it is 300,000. They request drug items with this many for each health center from [RESUPPLYING HUB]. Such an initiative is very interesting. It can be taken as best practice.

The quantification done at the facility level is not good. They increase wastage. They do not quantify unknown drugs.

I: What are main challenges for both of the commodities? Including data quality and forecast accuracy? We know RMNCH supplies are not in full.

R: IF we make the facility do quantification of RMNCH commodities, … Facility plan ambitious numbers most of the time. in my opinion, committees in the facility are putting in this ambitious number. It's better if quality data is analysis done for quantification. There are gaps in recording and reporting quality data. I think most RMNCH products are provided by donors. When you ask them for quantification, they always bring an exaggerated number. They will increase the number considering they will get additional funds for RDF. This will lead us to conclude that there is no objective data. I also believe gap in expertise skills to do the quantification. There is a skill gap in doing quantification and forecasting based assumptions. Fourth, quantification is not considered as the main task of that facility. There is feeling of not accountable for the shortages and stock out problem happened due to that quantification. If these problems are solved, the chance that the facility can get the products continuously will be increased. It will be better if these problems are solved and support is provided. To do this, the facilities need to be capacitated through close follow up for at least a year.

I: Can you tell us about the distribution and transportation?

R: [RESUPPLYING HUB] transportation is classified into two. It is from the center to the hubs and from hub to the facilities. There is no problem with transporting from center to the hubs as they are going to big towns. They are delivering it without any problem. However, transportation to reach the facilities from hub is very difficult. There is transportation limitation in terms of deliveries to hard-to-reach areas. Vehicles which fridge is installed can only function on "asphalt". For those who pick up which carry cold will be used. There is shortage of these cars. [RESUPPLYING HUB] faces challenges with its current capacity. The is also a problem with the utilization of cars. The cars are not efficiently maintained. The cars need to be maintained after delivering 1 or 2 rounds. There is no system that follows this efficiently. [RESUPPLYING HUB]

has manpower shortage to deliver products to each facility based on their request. They cannot deliver to the last mile according to the schedule in such a situation. [RESUPPLYING HUB] has also system problem. They are shifting from {eLMIS] to another system. It's been around three months and it’s challenging them. The other is order management. If the facility request is not coming on time, it will have an effect on transporting based on the schedule. For instance, if there are 20 facilities in one zone and 10 of them do not send a report based on the schedule, it may disturb the transportation process. Another important thing is how to deliver to seasonal facilities. Such thing needs detail analysis and finding a solution. I don’t think [RESUPPLYING HUB] should reach around 4000. There are sites that it cannot reach even whey are using pick up. Such a thing needs other options. It can be locally available for transport like animal and motor. This limitation can be solved by engaging private transportation in the area or the woreda can arrange it. I don’t know if your pilot area has such sites but, it is better to think that way.

I: What about route optimization and third-party logistics?

R: Third party logistics is private sector. third party logistics are available for transportation from the center to the hubs. Private sectors are involved. They use third party logistics when they transport it from Djibouti to the center. There are pilot projects on this. I don’t know if you are aware of [IMPLEMENTING PARTNER]. They started working with 2 cars. So, this indication of engaging third party logistics to reach many places. Third party logistics is very feasible for sites that [RESUPPLYING HUB] are not able to reach. Regarding root optimization, it is one of the objectives of our project. There are 300 sites that [RESUPPLYING HUB] is delivering. We do route planning by identifying facilities that are not included in [RESUPPLYING HUB]’s route. On the other hand, it is being updated on the system. You need to have facilities data to update the routes. All of the hubs have a route plan that is categorized as odd and even. They organize the vehicles based on the size of the facilities.

I: what about the challenges associated with woreda passthrough practice?

R: [implementing partner] has done one activity in [[zone]], [zone] and [zone}. Woreda passthrough is better than other initiatives. If there are 10 health facilities in the woreda, the individual will send the RRF and VRF (Vaccine request form). Then it will be packed individually. It will be delivered to the woreda for x and Y facility with their name labelled. It is better to do it

this way than packing in a packaging for the entire facility. Although it takes a long time, the facilities will get what they have requested. The limitation of woreda passthrough is the woredas has low transportation capacity. There is a high chance that it will be kept there without delivery. The facilities chance of stockout will be higher. The woreda may sometimes change or reduce and give it to another facility.

I: What do [RESUPPLYING HUB] capacity look like? How can the distribution and transport of last mile delivery be improved in an innovative way?

R: most of the challenges we faced during the implementation of our project are from [RESUPPLYING HUB]. As the number of site order increase it affect operation of [RESUPPLYING HUB] hubs. Order management around the warehouse is difficult for them. We have discussed with hub management and central [RESUPPLYING HUB] to overcome this problem. Additional manpower will be recruited to the hubs. They need manpower for warehouse management. They also need capacity building for warehouse operations to improve last mile delivery. Secondly, starting from national level, all leaders should buy the idea. There should be a launch workshop where all stakeholders can engage and discuss in detail on the issue and buy it. this will help the hubs get a solution faster as they discuss with leaders at the center. Thirdly, the establishment of internal TWG is very important. It is important to discuss internally on the challenge they have, how they can solve that challenge and which challenge is difficult to solve. They need to organize a platform where they can evaluate their capacity to overcome challenges. Three different platform is needed. We plan to have a central TWG in which [RESUPPLYING HUB] directors, regions and other stakeholders are involved. The second one is zonal TWG where the [RESUPPLYING HUB] chair and zonal distribution offer is secretary. This is very important as [RESUPPLYING HUB] will also explain the challenges they face. They discussed the data quality. Such a platform is very important. Otherwise, it will be very challenging to implement your project. We have successfully implemented this in a year. Regarding the facility readiness, most of the facility has storage problems. Most of the warehouses are substandard. Additionally, I think some RMNCH products need fridge. Fridge capacity should be checked in detail. It should be checked if they we have the capacity to store for two or three months. Whether we have the capacity to store enough products during the campaign. For example, last time we visited a health facility called Shamba. Oxytocin is stored in the laboratory unit fridge. There is one fridge in EPI

unit. This is not enough to hold a vaccine for a campaign. Products that need fridge is very critical. It also needs to check family recording and reporting practices as the data that comes from the facility is very important for last mile delivery. People at [RESUPPLYING HUB] are motivated. We need to plan together and continuously follow up. Arranging performance monitoring activities is also crucial. Back-order management is not practical. It is only documented (inaudible: 50:15 – 50:21). [RESUPPLYING HUB] provides it every two months. They are not delivering to a single facility.

I: Last time we were in[zone]. We can say there is no drug in the health post. Everything is stopped due to transportation and capacity. In your experience, what can be an alternative means of transportation to the last mile? What do you recommend?

R: Most African countries use segmentation, especially for vaccines. There are health facilities which are which are infant. They may not have cold chain services or electricity. This facility will be linked with those facilities which are in good status. Their reporting system will be linked to that health facility. The health center will request products from those health facilities from [RESUPPLYING HUB]. This might not be feasible for all health facilities. Such strategies can be used contextually. This it is very difficult to reach health facilities that are very remote, I suggest to have contractual agreement with local private transportation service. It can be motorbike. They will be accountable for delivering the products and making random supervision on its implementation. Partners may support them for initial implementation. Product management system at the health post level is infant. Only HPMRR is implemented. They stopped using Bincard. I think capacity building activity should be taken to a lower level.

I: What are the challenges of IPLS implementation?

R: People say IPLS is a system that is bringing nothing. But I don’t see it in such a way. Wherever you go the facilities are using Bincard. In fact, the bincard is already there before IPLS. They are using RRF for reporting. Every facility that receives products from [RESUPPLYING HUB] uses RRF. This is the result of IPLS. What every it is, IPLS is being used for logistics services in the country. We need amendments as additional requirements are coming frequently. Some fabricated stockouts are coming, I think we have to do more on triangulating the service data with the consumption. I don’t think it has brought 100% of what it has planned for. It was implemented to

have quality data. But this is associated with accountability and motivation. Since they are not getting what they requested, they may carelessly fill out a random number. Although its implementation is not effective, it has brought changes on country's logistic system. LMIS formats are still supported by donors. However, I have seen sites that are budgeted for printing IFRR. Since RRF is on a pad, it needs to have a budget at region, zone and woreda level. so far, there is no such consideration of printing RRFs on themselves. Amendment was done on RRF and we need to arrange refreshment training. Just like other formats used in the facilities, the regions should print RRF. It needs to aware them to print that has been when they print their annual reporting format. Regarding data quality, it is still challenging in may facility. It can be improved by the system. You can add service delivery data and they will fill out the correct data. There is a practice of triangulating it with service data. We have seen it while it is used in some facilities. Triangulating with service delivery data will require commitment and it is time consuming and its performance is lower.

I: What about emergency order frequency and wastage rate?

R: I recommend facilities send quality data. Emergency orders will be zero if they send quality data. The weakness of the I facility is measured using its emergency order frequency. If a facility has many emergencies order, it has poor data quality management system. It is not reporting what it needs or what it has used. Emergency orders should be the last option. Hospitals may use emergency order if [RESUPPLYING HUB] is not issuing the product they requested. I have not experienced a report which indicate the percent of facilities that requested an emergency order.

I: What about wastage rate?

R: Wastage cannot be inevitable. I cannot avoid wastage. But we can reduce it. for example, for RMNCH, I have seen magnesium sulfate injection expire due to lack of communication between store managers and clinicians. Wastage always exists but products that are not supposed to be wasted are being wasted. You may get the actual figures from [RESUPPLYING HUB]. There are facilities that have 5 to 10%. However, it is reduced from the previous time as the push system is reduced. The facilities are refusing to accept the products that [RESUPPLYING HUB] is sending without their request. I hope you will take baseline data from the facility and your project will work on it. For instance, if we take baseline wastage for LMD project, we will see to the changes

we have brought at the end. Wastage is very common at woreda level as they are storing products of many facilities. They might have no commitment to distribute it to the facility and the products are becoming expired. To prevent woreda level wastage, the facilities should not use them to store the products. Last mile delivery is a strategy to reduce wastage.

I: What are the challenges of implementing electronic LMIS?

R: Electronic LMIS is the one we call [eLMIS], which was a different version. In our LMD project, we had been discussion with [RESUPPLYING HUB] and zone representatives. What they raise as a problem is the implementation of [eLMIS]. They say “we have capacity problem; we need to have training and supportive supervision”. I feel there are a lot of things to be done. Facilities that are expected to send electronic RRF are not sending it accordingly. This happens due to failing to make a transaction using the system. [eLMIS] must be a system that they use in a simple and entertaining way. [eLMIS] must simplify the activity of the facility. My recommendation on [eLMIS] as logistician is, it should be simple to be used at the facility level. It should be simple and easy. Plus, it should be selective. For example, all the program and RDF is being entered. It will be boring to the store manager to fill all those products issued to all the dispensaries. It should include only the data that is needed for decision making. It should be visible up to the top level. systems implemented at the facility level should be simple and easy as there is a capacity and manpower issue at that level. One of the big challenges of implementing [eLMIS] is manpower. The system by itself should be evaluated. Whether it is bringing what it is expected to bring.

I: What is the effectiveness of inventory management system?

R: The first thing in inventory management is data recording. Most of the facility knows how to filling and updating the bincard. But I feel there is some commitment issue in some facilities. Updating bincard is better on sites where interventions are carried out. I have no understanding of the effects of overstocking or understocking. There are activities that are started to make them do stock status analysis and fishbone analysis to identify the problem and take intervention. Such things should be on all sites. You may find only 3 from 20 sites. It is infant at facility level.

I: What about its supportive supervision?

R: It is mixed. The supportive supervision provided to facilities may differ based on the projects. When I was in JSI, there was staff who conducted supportive supervision. It has a mechanism for supportive supervision. There is a mechanism for having minutes of feedback provided during supportive supervision. Feedback provided during supportive supervision should be clearly presented in all facilities. Such practice is available in some facilities.

I: Understanding of HEWs on the stock out triggering points?

R: I don't know if their curriculum is updated. Have been visiting hospitals, health centers and health posts while I was working in the field. You can start talking informing them about the logistics issues. We will tell them what model 19 is, stock status and bincard. You have to make them understand everything about logistics. They are expected to feel the essential data on HPMMR and the professional at health center will calculate it and send them the amount required for the health post. They are not filling the bincard. They are not filling the HPMMR correctly. They fill 00 for begging balance. They only fill the ending balance correctly. Although they have minimal engagement in supply chain management, there is big problem with doing it with quality. As mothers and children will have the first contact with their support, it is very important at this level. you cannot get them in the health post most of the time. You will see it when you go for supportive supervision. They are not around most of the time. You will find a closed health post when you go for supportive supervision. We can say it is not functional at the health post level.

I: What are the internal reporting and resupply system's challenges with the facility?

R: Dispensing units are not doing internal reporting on many sites. As it affects the warehouse activity, he goes around the unit and fills it out for them and asks them to sign on it. Due to this, they don't have motivation and a feeling of responsibility. I feel this is related to facility management weakness. Unless the warehouse asks for the report no one can deliver it. They wait until they finish what they have at hand to go to the store and take it. there is no trend of asking ahead based on the schedule. I am not saying this is common in all facilities. I am saying it is not to the expected level.

I: What is the main reason for this?

R: it should be considered as one part of the responsibility and they should be evaluated for this. There is a gap in making those who are not filling IFRR accountable. The warehouse manager is not reporting to the facility manager to make them accountable. This is due to loss of accountability and responsibility. There might dispensing units that have a skill gap on how to fill out the formats. The warehouse manager or facility pharmacy head has a responsibility to identify and capacitate those with skill gaps. It might be format issue. Currently, IFRR formats are not printed. IF the IFRR they have is the old one, it can be one of limiting factor. As you know papers are expensive, there are facilities that doesn't have printer, so the format may not be available.

I: What should be done differently to improve the system?

R: Whether the facility is providing quality data or not, that facility is not getting the right product [RESUPPLYING HUB]. At least the facilities should get fully supplied products. RRF should be updated routinely as drugs are added and removed from the list. There are many facilities that are not using the revised RRF as it is not provided to them. Huge number of resources are invested but the output of IPLS implementation is not at the expected level. Facilities that do not have resources and skill gaps should be accountable. There should be a system which makes them responsible for the created gap from their side. [implementing partner] health facilities should be accountable for the gap they create. It can be by writing an official letter or discussing with the leader of the facility about it, or signing memorandum of understanding. What I think should be done differently is, security and transportation problems, it may require resources to send RRF every two months. Simple excel system that records all important data should be implemented.

I: What are the barriers and opportunities to strengthen the health post-resupply system?

R: I feel the products should not be sent to them. You will see the actual situation during your baseline assessment. Very expensive drugs are exposed to wastage. Have you seen the facilities? The buildings are built well. However, emergency drugs should be available there. We may need to discuss with kebele administrator. They have a storage problem. They don't have shelfs. There are rats in the building and they are destroying the products. The products that can be prone to theft and wastage should not be taken there. Health centers are responsible for making close follow

up and provide supervision. they use motorbike or travel on foot to carry the product they collected from health centers. They should be paid for the transportation they use. You have to check whether they can keep the products or not. There are health posts that do not have fridge. I am not sure if the HPMMR format is available in all health posts. They need to identify which products they can have in the health post. They need to be included in the training and performance evaluation strategy. They are ignored most of the time.

I: What about their interaction with the pharmacy department? What should be improved?

R: Actually, they are only expected to count the available drugs. Unless the pharmacy warehouse manager registers the expired data of the product he issued last time, they will count all available drugs. The pharmacy head and warehouse manager were not going to health post to provide supportive supervision. Facility heads might go and check their activity. They will only meet with the store manager when they come to take coartem or family planning product. Their interaction is low and on-the-job training is not given to them. They are strongly linked with EPI unit.

I: What should be done?

R: I am going to repeat what I said earlier. There is a package that health extension workers should perform. We have to check if the environment allows us to do this. If we expect them to have the drugs, do you they have that infrastructure? We have to fill at least one place where they can store the drugs. They need an orientation on how to fill out the data. We need to check if they have HPMMR and bincard formats. The facilities should pay for the transportation when they take the drugs to the health post. Most of them are not motivated due to this. she may say it is available while she is out of stock. It is better if a system is designed to overcome this. it is also better if a system is established to engage warehouse managers during supportive supervision.

I: **How are supply chain data generated, shared, and utilized for decision-making within the health administrative units and facilities**?

R: It is infant. Some health facilities where we implement our project are generating and using the data for decision making. It can be stock exchange or to reduce stockout rate. They may use to

follow whether their consumption is higher or lower. They use it for refill supply by checking the amount they requested and the amount they used. Such things are practiced in some of the facilities. Most of them will shout when they are out of stock. There is also misuse. If we take Dipo, it is mostly misused. The main problem is related to accountability and responsibility.

I: What additional recommendation or any best practice that can be scaled?

R: I have raised most of them during our discussion. As a general, to improve last mile delivery, there should be better infrastructure. Transportation network that I have mentioned earlier. For example, finding alternative option to engage private sectors in inaccessible areas. Secondly, enhancing storage capacity of the facilities. If you are planning to reach health post, you need to work on improve storage capacity before delivering the products. Inventory management system should be strengthened. The manual inventory system may not be productive so, electronics inventory system is crucial. The other thing is to capacitate facilities to predict demands. It needs to create a facility which say my RMNCH demands in this amount. This will prevent over and under stock. [RESUPPLYING HUB] should provide the products that facilities are requesting. If the facility is requesting with 100 % accurate data and [RESUPPLYING HUB] is providing only 50% of what they have requested, it will be very difficult. This will lead to stock out and facility may not fill out accurate data next time. I hope the program also considers products that need cold chain and it needs to consider improving cold chain system infrastructure. It can be buying or maintaining fridges. If you are planning to work on vaccine, cold chain management is important. The country has to work more on cold chain system, especially at health center level. it needs to provide regular maintaining training. Retention mechanism is also important. Retention mechanism must not be monetary. If you give recognition for their performance or give training, you will increase it. Coordination and communication platforms are also very important. TWG and coordination platform are need at national, regional and zonal levels. It may also need to revise policies. When we say policy it can be on availability of RMNCH products and family planning products. It can also include funding sources. Advocating on how to use new products is very important. We have been talking about demand forecasting. It needs data driven demand forecasting. It also needs to have adjustable procurement plan. Strengthening data management system is also needed. Community engagement is also needed. If we are planning to provide new products that are newly introduced, the community may not accept it. it needs to make the

community become aware and use it. the other important thing is technology integration. Service data should be integrated with logistic data. The data should be available at national level for decision. Emergency preparedness and response plan is also important. It will be interesting if these things are addressed.

I: Thank you so much for your time.

End of interview

KII interview (10)

Name of Project:	SSD
ID Code	KII-SP -01
Type of interview	KII
Type of informant	[sex]
Region	[region]
Role of the Interviewee	[MCH expert]
Years of experience	10+
Name of Transcriber:	[university Lecturer 2}
Date of Transcription	August 2024
Names of Reviewers:	[university Lecturer 2]
Length of time	1:19 minute

I: Can you describe your role and responsibilities within the health supply chain system?

R: In the [region], including [town], there are three hubs. We are assigned specific zones for support; for example, I am currently supporting [district]. We use a WhatsApp platform that includes members from the Woreda Health Office Heads, the Regional Health Bureau, and [RESUPPLYING HUB]. Health facilities submit their RRF (Request for Requisition Forms) via WhatsApp, allowing both the Regional Bureau and [RESUPPLYING HUB] to review them. We

provide comments and feedback on the completeness of the RRF and address any additional issues. Our role is mainly to offer feedback regarding the RRF only and completing and submitting RRF is role of the woreda.

I: Probing: How do you perceive the effectiveness of the supply chain management system?

R: I believe that it is not effective because it is not started with capacity building at a lower at lower level. The pharmacy professionals, Nurses and other health care providers don't take any training in regard to supply chain management systems system. In some facilities, there may be complaints about insufficient commodities. For instance, a health center might receive only 100 syphilis test kits, which could be inadequate for their needs, leading to dissatisfaction. This issue often arises because the RRF (Requisition and Reporting Form) may not be filled out completely or submitted on time. There is no responsible body in every woreda and there are always is always errors. I believe that there is not enough no enough supply in every woreda. I did not see facilities with enough supply. Even in some woredas the clients are enforced to pay fees for the program services even though the services are free. There is no uniformity among woredas on the service fee.

I: Probing: What are the problems on RRF?

R: Filling out the RRF is not seen as a major issue among health facilities. In most of the cases, somebody may complete RRF without the woreda and [RESUPPLYING HUB] ensuring competency at the [RESUPPLYING HUB] and Woreda levels, person who completes RRF healthcare providers often lack consultation with relevant departments and may not understand how to complete the RRF. For example, he may not request test kits while there is shortage. There is also a communication gap in between them. Consequently, they do not regard the RRF as crucial for estimating drug requisition and demand. Some may not understand that resupply is depends on the quality of ordering and requesting. Additionally, even when facilities submit the RRF correctly and on time, [RESUPPLYING HUB] sometimes fails to supply the requested commodities.

I: Probing: What is the problem behind if the facilities are correctly filled the RRF?

R: We don't know the reason behind, may be shortage of commodities whether it is due to RRF

completeness or shortages of medicines. [RESUPPLYING HUB] also receives commodities from[central resupplying hub] after compiling the RRF. The [RESUPPLYING HUB] doesn't give the number of commodities that the health facilities requested. For instance, if they have 5000 of a certain items and one facility may request 2000 kits, [RESUPPLYING HUB] doesn't give the requested amount and [RESUPPLYING HUB] may take some considerations. , [RESUPPLYING HUB] allows them at least 1000 or some amount.

I: What are the major barriers that deter continuous availability of health commodities at health facilities and health posts?

R: The [resupplying Hub] covers around 80 woredas. It is so hard to cover all these areas. May be after the [resupplying hub] hub opens, the burden will be minimal. In Some woredas the health facilities receive the commodities directly from [RESUPPLYING HUB] and other facilities from some woredas would receive the commodities indirectly which means that the health facilities receive commodities from woreda health office and zonal health departments after [RESUPPLYING HUB] stocked them. Program drugs are not stored in the RHB warehouse, specifically emergency drugs and drugs from donors are stored at RHB warehouse and mostly these are planned for targeted woredas and distributed based on the regional plan. . As I told you before, what I assume is that the problem is nothing is done at a lower level about how to manage it if once it is stocked at woreda level. Sometimes, family planning supplies are available at the woreda level but are in short supply at the health post level. There is no established system at the Primary Health Care (PHC) level for replenishing these supplies at the health posts. Instead, health centers address these shortages by restocking health posts during their supportive supervision visits.

I: Probing: What types of support do you give for health posts and health centers?

R: Commonly during supportive supervision we review the RRF and we asked MCH head as to whether RMNCH commodities are requested and how to communicate each other within the department like MCH head and store manager before fill out and request commodities. To solve such problems system should be established to link out the program and logistic personals. Capacitate and increase their commitment are necessary for logistic personals. There is also negligence among logistic personnel, they believe that we always don't receive what we requested.

If supplies are not resupplied based on requested quantity, which may affect the value of completing RRF.

I: Probing: What about the budget issues?

R: Regional bureau doesn’t allocate budget for the facilities rather the woreda cabinet /finance/ allocate budget for the woreda health office then then the woreda health office cascade the budget to the health facilities for non-program drug procurement. For program like maternal, no need of money allocation. If once they requested the regional bureau, they can receive what the amount they need. I'm facing some uncertainties with several issues. For instance, certain facilities lack essential medical equipment, and it's unclear where to request these items. We’ve reached out to the [higher level] but haven't received a satisfactory response or solution. There’s still no resolution for these problems. While some medical supplies, like magnesium sulfate and oxytocin, are provided to clients for free, the availability of other drugs is determined by the woreda health office head, leading to inconsistency from one woreda to another. Overall, there is no streamlined system to address these issues effectively. In some woredas mothers pay fee for those drugs which should be provided freely.

I: How effective is the current supply chain workforce staffing and capacity in managing the supply chain at various health care system levels?

R: I believe there is a shortage of pharmacy professionals, leading to many tasks being handled by clinicians such as nurses and other healthcare providers. Additionally, we lack a structured induction and skill transfer program for new hires and for professionals transferring to one place to others.

I: Can you describe the current quantification and procurement process for RMNCH commodities (through program and RDF schemes)?

R: I don’t have any involvement during drug procurement. May be the woredas themselves purchase drugs through their system. Before four years the ministry of health allocated a budget for hospitals to purchase drugs as MCH a reimbursement, but currently that is not the case as hospitals are supposed to manage it by themselves. Before, there was like allocation of 400,000 ETB for reimbursement for a hospital. I was working with pharmacy once ; we made first lists

of drugs and check service data to forecast the amount with conversion factor. But currently I don't know what it looks like. Maybe hospitals who have strong logisticians can buy using that system.

I: How did you see the procurement and quantification process during supportive supervision?

R: We cannot inquire about the purchase status at this time. If the districts and hospitals have enough budget, it will be quantified and forecasted, and then purchases will be made according to that budget. As I mentioned, hospitals purchase what they need after making accurate forecasts and quantification. However, I doubt at the forecasting exercise at district and facility level. The RRF itself includes quantification and forecasting. We can notice a lot of zero reports, incomplete and inconsistence in the RRF, it's better to fill it based on the current inventory to avoid waste. It's important to pay close attention when filling out the RRF form, as it provides clues for forecasting the next commodity amount based on the previous two months' usage. This process requires serious attention and awareness. Additionally, there is currently a lack of communication among program personnel and logistics team.

I: As you said there is better forecasting and procurement process in hospitals, what are the solutions in woredas and Health Centers for better work?

R: Following this, these individuals should receive training or capacity-building support. Next, effective coordination and strong linkage between the woreda program and logistics personnel are essential. Both logistics and program staff must pay close attention to their roles and ensure accountability is firmly established. Additionally, systems should be developed and strengthened at the PHCU (Primary Health Care Unit) level.

We have observed varying situations across different woredas and health facilities. For example, some facilities have an adequate supply of injectable contraceptives, while others lack them entirely. This disparity highlights the issue of inconsistent availability of commodities in both woredas and health facilities.

I: How does the transportation and distribution system for RMNCH commodities, including vaccines, function across different settings?

R: The transportation system performs well, particularly during campaigns. There is relatively better attention for EPI. However, there are issues with resource scarcity and other challenges that lead to commodities, including vaccines, being delivered to facilities on the same route within a single day. The RHB provides deliveries to these facilities every two months, which means there may be periods of commodity shortages in between. Although [RESUPPLYING HUB] follows its own delivery schedule, it often cannot meet this schedule due to a shortage of vehicles and road problems. Some health facilities have reported that [RESUPPLYING HUB] does not deliver commodities on time. [RESUPPLYING HUB] also tends to deliver to multiple facilities on the same route rather than servicing individual facilities according to the schedule. There is no good infrastructure like road access. We have an information from health facilities that [RESUPPLYING HUB] couldn't deliver commodities on time.

I: Probing: Did you have any discussion with [RESUPPLYING HUB] about the problem?

R: There is new maternal health kit that has not yet been distributed. I tried calling the logistics officer for assistance, but he did not respond so far. Additionally, there seems to be a communication barrier between the RHB and [RESUPPLYING HUB] officers, and issues with updating information about incoming commodities. This problem could be due to factors on both sides.

We have three hubs: [resupplying hub 1], [resupplying hub2],and [resupplying hub 3]. We've received complaints from health facilities on the delivery of commodities, particularly from the [resupplying hub]. Our director has visited the area to address these issues.

To improve the situation, I suggest that woreda health offices communicate directly with the hubs to resolve problems more effectively. Another solution is to ensure that health facilities complete and submit the RRF accurately, completely, and on time.

There other solution is smooth communication with [RESUPPLYING HUB] officer is mandatory. We should exchange data especially about the delivery status. About for which health facilities does [RESUPPLYING HUB] provided commodities and which facilities are left monthly or every two months or quarterly. These improves commodities availability for facilities. We're facing issues with commodity distribution, particularly with program-related items. Data indicates that

drugs have been issued to facilities, but facility heads report that they haven't received anything. We're still uncertain about where the problem lies, and this remains a significant challenge for us.

We must have a system to get any information from [RESUPPLYING HUB] about which facilities get medicines and which facilities do not. Even it is good to know the number of commodities that are purchased from [RESUPPLYING HUB] through woredas.

I: Probing: what would be the solution?

R: Continuous review meeting and accountability and data sharing among [RESUPPLYING HUB] and woreda or Health facilities. Commodities stoked at the at woreda health office before delivered to facilities. Some woredas may have enough stock whereas the others may not have commodities. Transporting commodities from the woreda to health facilities is challenging due to a lack of vehicles and the fact that some facilities request supplies verbally rather than through the RRF. Health posts are tasked with receiving drugs from the PHCU. During supervision, woreda health office experts issue commodities using the model 19 form and distribute them to health facilities in the same manner, allowing health posts to receive drugs similarly.

I: What are the main implementation challenges of the Integrated Pharmaceutical Logistics System (IPLS) in health facility?

R: Currently, there's no cooperation from [RESUPPLYING HUB], particularly regarding emergency orders. Recently, the [RESUPPLYING HUB] head issued a directive for emergency orders, instructing health facilities to wait for their routine scheduled to receive commodities. As HIV testing services are free, only 60 out of 90 woredas are currently providing them. The remaining 30 woredas have reported a lack of test kits and have ceased offering the service. Health facilities requested HIV test kits through the RRF, but the [RESUPPLYING HUB] has indicated that RRF alone is insufficient; a service report is also required but woredas/facilities may not know that. This report should include the number of kits previously received and details on the number of positive and negative results. Without this information, [RESUPPLYING HUB] will not provide additional kits. Unfortunately, this requirement was not communicated to the woredas in advance. The (region) only learned of it after [RESUPPLYING HUB] imposed restrictions on kit distribution. Some facilities that have submitted service reports are now receiving HIV kits, but

many others have not, as they find the new reporting requirements burdensome compared to previous procedures.

I: probing: What measures have you taken?

R: We have communicated with [RESUPPLYING HUB] and the woreda heads. As moderators, we cannot enforce compliance but can only encourage them to cooperate voluntarily.

I: What are the implementation challenges of the electronic logistics management information systems (eLMIS) in health facilities?

R: I have some information but I don’t know what exactly mean.

I: How effective is the inventory management system in woredas, health facilities and health posts?

R: I have worked on logistics monitoring for other non-governmental organization. We have reviewed lots of problems like no shelf, no good drug arrangement and also there is no inventory using BIN card. Most of the health facilities don’t use BIN card and there is no BIN card in the facilities too. Hospitals, they use well. During supportive supervision we also evaluate and review the availability of commodities rather that the utilization of Bin card.

I: Probing: How did you give feedback about the supervision?

R: After supervision we give feedback orally on site for the facilities. For zonal departments, there is only one person assigned in zones, thus, we give feedback for that single individual. Here in Region] the zonal departments are not well organized, even as I mentioned they have only one person in each zone. Just our feedback looks like fill the RRF correctly and timely, the schedule. We give feeds backs when there is integrative supportive supervision like BIN card, stock analysis, how to manage expired drugs and others. No system or what we made in regarding to stock exchange or transfer in woreda level or at facility level. Commonly departments conduct supportive supervision independently. May be once in a year integrative supportive supervision is conducted.

I: Probing: How do you conduct stock analysis and prevention of over stock?

R: Typically, overstocking is not a common issue, although it does occasionally occur. Some facilities may have excess supplies while others may be lacking, which is a frequent scenario in our setup. Notably, there is an overstock of IUCDs in some facilities. This is due to the fact that mothers predominantly use injectable and pills, and more recently, implants. IUCDs are not widely used in region] for family planning; they are rarely chosen except by some mothers following a cesarean section. Therefore, while stock levels vary between facilities, there is generally no significant overstock issue overall.

I: Probing: Understanding of HEWs on the stock out triggering points such as campaign, case surge, product expiry/damage?

R: Health posts are responsible for collecting commodities from health centers, typically making requests verbally. Some health posts focus solely on delivering immunization services. In and around [resupplying hub], these health posts are relatively well-organized due to support from various NGOs and there is strong community awareness too. However, in more remote areas away from [resupplying hub], they pastoralist communities' nomadic lifestyle makes the health services delivery more complicated. Only they serve for very few individuals. Orally by phone call they request drugs from the health center.

I: What are the internal reporting and resupply system's challenges with the facility?

R: I can't say for certain, but I believe they have an internal reporting system in place. As mentioned earlier, internal communication between department heads and the store manager is weak. Therefore, there is a need for improvements in awareness, commitment, and staff accountability at the lower level. All facilities should have a clear understanding of their roles and responsibilities and ensure that everyone is informed about available free items. Currently, in some facilities, clients are charged for free drugs like iron for pregnant women, while others provide them at no cost. This inconsistency arises from the lack of straightforward systems in the region.

I: Probing: Behavioral and technical challenges on the regular reporting?

R: I mentioned before, no more to say about it. They use as usual if once they received from the store man.

I: What would be the solution?

R: First, issues at the woreda level must be resolved. Effective coordination and linkage should be established between the woreda health office and health centers, and between health centers and health posts. The quality of services at the health posts should reflect the support and commodity provision from the health centers. The health center is responsible to provide supportive supervision for health posts, but what we know is there is a gap in regards to frequency of supervision and feedback system since health facilities should visit the health posts monthly and quarterly. Overall health post and health center linkage is weak so far.

I: How are supply chain data generated, shared, and utilized for decision-making within the health administrative units and facilities?

R: There is a major issue with data utilization among the facilities. We are working to make quality data a central part of our decision-making process. Additionally, there are challenges with data quality and effectively using action plans to achieve facility-level services and outreach goals. We have a plan to strengthen the PMT at health facility level. In hospitals the PMT works well. There is no even data discrepancy. Sometimes the PHCU doesn't incorporate the health posts' services in monthly report. Logistics officers are part of the PMT, but they are not fully functional in their roles. Although all department heads are PMT members, they often do not review data or reports thoroughly. We have observed instances where reports include data from health posts that were not actually done at health post level. While PMT members' names are posted in every office, the primary issue remains their lack of effective functionality.

I: probing: What are the major challenges that makes the PMT not well functioning?

R: The primary issue is a lack of commitment and attention to the importance of the PMT. Additionally, there is insufficient regular monitoring and supportive supervision of health facilities at the woreda and RHB levels. We often correct data discrepancies by calling woreda health offices and health facilities this is because of the health facility's' PMT are not organized well and don't evaluate their report. Although we conduct supportive supervision, it is limited to specific areas due to budget constraints and transportation problems. We cannot supervise all facilities or

woredas consistently; our efforts are program-based. We have also provided sporadic support, such as RRF filling guidance and training sessions.

I: How do you address stock outs or shortages at the last-mile level?

R: We request support from donors and the Ministry of Health while applying positive pressure on [RESUPPLYING HUB] to ensure timely delivery of commodities to health facilities. Currently, there is no commodity procurement conducted at the regional health bureau level.

I: What are the most successful examples of best practices in the supply chain that could be scaled or replicated?

R: We don't have any exemplary practices to share. However, may be if it is, in one time we asked donors to buy hospital beds and other medical equipment.

I: Recommendations for seamless flow of commodities in the subnational supply chain

R: Improvements are needed at both lower and RHB levels for better management of the logistics system. This includes assigning responsible individuals, constructing and renovating stores at all levels, and raising awareness at the regional health level about the importance of LMS. Induction of higher officials is also crucial, as their involvement is essential for strengthening any program. Additionally, woreda health offices and health center directors must be educated about the system to enhance its effectiveness.

I: we have finished, thank you for the time

R: thanks!

Transcript 11

**Place/region**	[region]	
**Role and respo.**	KII -SP -03	
**Educational status**	First degree	
**Interviewer**	[university Lecturer]	
**Transcriber**	[university Lecturer]	
**Year of experience**	Above 6 years	
**Sex**	[sex]	
**Total Time length**	1:25:42	

I: First I want to thank you for your willingness, and then let me raise the first question. Can you tell us about your role and responsibility that you have in this institution?

R: I am a Distribution and Fleet management Team leader at Resupplying hub. I am leading the team with the distribution of the vaccine and the medicine.

I: How do you evaluate the effectiveness of supply chain management? As you were here and now you are the leader of the team, how do you explain the general accomplishment of supply chain management administration?

R: When we compare things that were six years back with the current situation of commodity distribution, the main target of the distribution is to address the necessary equipment/supplies to the person who needs that means the last mile distribution. When we look at the direct distribution that was performed six years ago, it is nearly 28% of facilities received directly, but the rest 72% get the distribution through the zone or woreda. Currently, we are directly able to address around 75% of the necessary commodities for the health program. Yearly we plan and the number is increasing from time to time. Regarding distribution, our main goal is direct delivery or last-mile delivery; the customer can easily use it if the delivery reaches the service point. When we use the distribution through woreda/zone the resources might not arrive at the service point, and it is difficult to request who else do that. Concerning vaccines now we are around 47% but before six years we were 0% in all Woredas; we are forecasting to reach 80% to 90%. Within the coming

two years, because of the arrival of supportive organizations, this is the one. The other thing is that when we deliver these resources data/reports are required about the facility readiness, especially about the vaccine. Now, we communicate with the health bureau about the holding capacity of the health facilities and we request a study report concerning the holding capacity of their facilities since we have a capacity regarding the vehicle and the resources. Still, we are waiting for their response to expand. The other thing is the RRF, including the gaps that we have with the RRF now we are nearly 75%, but we monthly provide feedback about how they send the form with quality and trimness, how many RRF are we received. Beyond this we have a what app platform only to exchange information about RRF, using this platform we are providing feedback on the spot and this helps us to make corrections immediately because of this there is improvement we are about 89 to 90 % it is displayed in the report. Generally, according to [resupplying hubs] we have 110 facilities under this situation means that there are 110 facilities that we communicate through RRF. For VRF vaccine usage we have around 107 facilities.

I: probing: Why the difference did occur?

R: There are regional laboratories that are not using the vaccine, that is why the number is decreasing. Overall, we are working to make our direct delivery 100% as the last mile delivery.

I: Thank you very much, what are the problems as you mentioned earlier the performance is 47 % and 28% as you have said there is a little improvement and you are working to increase these performances. As you mentioned, it is better to deliver medicines directly to address the customers who use them.

I: Probing: what kind of challenges are there?

R: Every time there are improvements, it is better to look by dividing them into the problems that are solved, problems that will be solved, and problems that cannot be solved.

I: Well, let us start with the solved problems.

R: The first solved problem is, the facility readiness, and our capacity mean that because of the lack of facility readiness, there were resources that we could not address. On the other hand, we were ready, but the lack of road problem was a great challenge for us. Because of this road-problem, we were completely unable to reach two woredas.

I: probing: What are these two woredas?

R: **[district 1]** and **[district 2]**. The regional bureau also has the information. It is a place were surrounded by the flood and the road is cut off. This is one problem. Though we did not reach these woredas, some woredas have very bad road types, and if a vehicle goes once and when it comes back it is directly going to the garage. Our main issue is road problem. This is a problem that we are unable to solve. I think this case might be solved by the region or by the country. But as I have informed you 75% is our great achievement though it is a bit slow. Regarding vaccines, we have the capacity and we can reach 80%, but the problem we blocked is that we are waiting for a report from the health bureau about the facility readiness unless we have confirmation as it is known vaccine is something which is expire easily so we did not take and throw it away. There should be trained personnel because of this we hold it and we are communicating. On the side of [RESUPPLYING HUB], we can reach. Except for this, there is a problem, now we have caught four zones for three years, but we have seven zones before the [resupplying hub 2] is opened and the branch holds three of the zones. When the seven zones were with us, we were forced to drive 1000 kilometres for one-way distribution starting here in [resupplying hub]. It was difficult to hold vaccines for more days and it was very difficult. Now, the other [resupplying] is opened and our radius is narrowed. Our current radius is four hundred kilometres, and these were the problems that we were facing. The zones that we have are named as [zone 1], [zone 2], [zone 3], and [zone 4].

I: [implementing partner] currently expanding sites. There is one [district] and there is another called [district].

R: There is no woreda called [district], it is zone. Under (zone] there is [district 1], [district 2], [district 3], [district 4], [district 5], [district 6] and [district 7] these are found there.

Interviewer: Is [district]under your catchment area?

Respondent: Yes, [district is under our radius.

I: What are the challenges that hinder you from delivering the commodities till the health post? Can you tell us something related to this issue?

R: Okay, our last point is the health centre, and the health bureau is the one that reaches the health post. How it arrives/or delivers there, I think they might have a strategy but I do not know their strategy. The great issue for [RESUPPLYING HUB] nowadays is direct delivery which is the last delivery. Now [RESUPPLYING HUB] is in a good capacity, and it does not have a vehicle problem like the previous time. We were facing a great challenge at [RESUPPLYING HUB] as we were traveling a long distance, because of the opening of one hub, on our side ([RESUPPLYING HUB]) I do not think we have a problem. As I have mentioned earlier road problem is one of the problems and as [name of a person] has said the filling of RRF is the other challenge. I do not know how much it takes but I believe it will be improved. To solve this [RESUPPLYING HUB] and regional health bureau and other concerned bodies should work cooperatively to solve the problems. On our side, we are struggling and solving problems at each stage. As I have informed you, we have a WhatsApp platform at the zonal level, by the way Somali region did not have a zonal structure like other regions like [region] and [region]. There is only one professional who is working as health works at the zone, so we have a close relationship, and, in the WhatsApp group we exchange feedback there is an improvement but not as it is expected. Concerning the vaccine, the main thing is facility readiness; facility readiness is not our mandate, it is beyond our mandate and the region should take this responsibility. We had requested by letter to the regional health bureau, that we are planning to deliver resources and the holding capacity and the facility readiness should be confirmed before the delivery so that we are waiting for their response. Except that we are ready for the delivery.

I: What are the obstacles that hinder the direct delivery beyond the road problem? Do you have something to say about the inventory and controlling mechanism and the facility's internal system?

R: It is related to vaccines. For example, today a new health centre might be opened in a place where road problems might not be a problem. [RESUPPLYING HUB] can simply deliver medicine to this health centre the formality should be kept and RRF must be submitted. By requesting health facility professionals how to fill the RRF and asking them about the inventory management of the commodity. They should have a hint and need to inform us what has to be done. As I have informed you this thing has to be performed by the regional health bureau. When a new health centre is opened, we provide RRF and they directly came and asked us we provide a

short training here how to fill RRF, that we inform the focal the opening of the new health facilities and they need to check whether they send RRF or not, monthly they are providing feedback in the WhatsApp group about the status of RRF mean that whether they send or not the RRF. We tried to manage the quality and other issues based on the feedback. The road problem is one and if the facility needs and if it is not beyond our plan, we do not have a problem to deliver if the facility is ready.

I: How do you evaluate supporting each other [RESUPPLYING HUB] with woreda, zone (though there is a single individual), have you seen shortages concerning working cooperatively and supportively with each other?

R: Honestly speaking, we closely working with the region and they responded to our questions as I have mentioned earlier there is a zonal focal professional, we also work with closely because there is a model issue during the distribution. One of the problems is that completely one model has disappeared and we solve this problem by communicating with them. There is model 19th that you can find only in the woreda in the Somali region. The issue model is a great thing for [RESUPPLYING HUB] to bring that model because the donors are looking for that model, as commodities are issued by the donors and they need to know the distribution so it is obligatory to bring that document. The health bureau did not have the model, because of this the medicine was submitted to the finance bureau. Regarding the model, we have solved the challenge and now at this time, all health centres have the issue model. Beyond that they support us in every aspect, and we have a close relationship and the region is here also; we solve other problems too. As I have informed you about the facility readiness-related issue, they cannot respond quickly because it is mandatory for them to go and evaluate the facilities. I think because of this; they are unable to respond on time. Other things we communicate and solve issues on the spot.

I: Forms that have been filled are a resource to you, how do you evaluate the correct filling out of the formats?

R: Yes, that is what I have informed you earlier.

I: Though 100% of the formats are filled, how completeness and timeliness look like?

R: Yes, we have the form and we provide feedback. We told them that the report we expected seemed this but what we have received is. It has this much quality and timing. We provide feedback monthly and based on that feedback there is an improvement though it is not as it was expected.

I: How do you evaluate the effectiveness of the current human power number, capacity, and skills with tasking the supply chain management starting from here till your roots?

R: On which side?

I: You are going to tell us both sides as you are working together as you must meet, and it is a barrier you will tell us about [RESUPPLYING HUB] and the human power at the facility centre. Even though you have enough amount, so how do you evaluate the amount and quality of human power, capacity, and skill on both sides of the supply chain management system?

R: [RESUPPLYING HUB] has a sufficient amount of human power in quality and quantity. Especially the professionals are capacitated well and they do not have a gap. When we were looking at the facility, the problem of filling RRF i.e., requesting and reporting because there was one professional who was filling that form when s/he released the job s/he did not share the experience of how to fill the form. When the form arrived with us the report showed us the woreda that we did not know before there was such kind of gap. I am not familiar with the current structure of the facility I know the previous structure and the amount of human power. I believe as the facilities are found in a remote area the human power shortage might be the problem, this is not the problem of the structure it is the absence of the professionals who want to work in a remote area. All health centre structure is formulated by the Ministry of Health structures that belong to the regions. I know what had been there since I worked in the {region} region, and there is a similar story in relation to this. In most places, you cannot find the pharmacist running this activity, and other professionals are filling in based on what we have shown them. Concerning VRF we do not have a problem since the pharmacists are not participating so the nurses are filling that. Most of the facilities use nurses instead of pharmacists. The inventory management of the health facilities is very poor.

I: What is the main problem related to this?

R: The new facilities premises that are built every were did not have a store.

I: Is it because of changing the role of the room or what?

R: No, if you look at the plan no store is included there. As you know every part of the building is assigned in the plan before it is placed to the ground, but in the plan, you cannot find the store in most facilities. In the areas where huge transaction NGOs are building health facilities, not health facilities that are found in the remote area facilities that are found in the town did not have a store. High-volume health centres did not have stores.

I: So, what do they do to keep the medicines?

R: By making one of the rooms that provide another service vacant they keep the medicines there. One of the issues that make inventory management poor is this. Hospitals have good stores. When we come to health centres, they do not have stores this case creates a problem with inventory management. Even though they are using another room to the store they do not have a full shelf to store the medicine. When you look at where the medicine is kept you are scared. If you inform the professional to use a bin card how he can use it as he places the medicine on the floor? There should be work on this issue.

I: what are other gaps that you Identify in addition to infrastructure?

R: There is professional turnover; there is a shortage of professionals on the market, especially pharmacy professionals.

I: When you post the vacant position no applicant?

R: Yes. There is no.

I: Does the quantification and purchasing process go under your notification?

R: Yes.

I: If so, how do you evaluate RMNCH quantification and purchasing, how do health facilities quantify and send to you? I think it is RDF.

R: We don't have quantification, and it is prepared centrally. There is a yearly RDF quantification program. We quantify, and two samples are taken. From each type of centre i.e., high volume, medium volume, and low volume, all hospitals are considered in quantification.

I: How do you level the facilities?

R: We get this from the health bureau and it is related to patient flow. Among these I think it is two/ four have been taken and quantification is prepared, taking this as a baseline generalization will be formulated. All hospitals are considered in quantification.

I: Who sends the data are they sent by themselves or do you go there and collect the data?

R: We send the formats and support them going to the facility centres. Before they start the work, we load the data and show them how to work on it when they finish, we collect it.

I: Are they sending through the woreda or directly to [RESUPPLYING HUB]?

R: The health facility directly sends to the [RESUPPLYING HUB] centre and they copy to the woreda.

I: Through that format, you can get the forecasting quantification. So, you support and help them. From the format that comes from the health centre, how do you evaluate the quality and completeness of the format filled by them?

R: I am not quite sure about this, as the task is performed by other departments and they are the ones who perform the details and send them to the concerned bodies. We can get the information there.

I: Did you get any problems through this way, in relation to quantification, commodity forecasting exercise, and data quality?

R: In this case, there is a well-known challenge that we have got. If you ask the health centre to forecast, they might forecast a 20 million birr but the budget for the health centre may be one million. They send all the types of medicine and you need to correct and send that based on their budget. If [RESUPPLYING HUB] brings the medicine based on their forecasting, no one is going to buy it. Referral hospitals have 40 to 50 million budgets, but they forecast a 100 million birr

which is a very high amount, and cannot be afford by them, though they have huge data we return and inform them to correct based on their budget. Therefore, this is one of the main challenges.

I: What is the problem in forecasting huge amounts of birr and what is their reason for that?

R: The first is negligence, from the format we recommended and inform them to take three months of consumption as a base, and they are expected to forecast the medicines that are necessary to the health facility.

I: Did not they forecast correctly using the three months' consumption as a baseline?

R: Sometimes there are some medicines that are not used or they are kept for certain purposes or the medicine might not be with us, or they did not get from the market, at this time they simply requested without following the steps. Lastly, the birr might be six/seven million.

I: Is there a medicine that they need but are unable to purchase?

R: Yes, there are.

I: What is the problem?

R: The budget problem is across the country. I have worked in the {region} region and the budget allotted to one health centre is 180 thousand this is the amount for a long time. Once we might buy two or three car commodities but when the medicine price is increasing the budget is still there, so there has to be revision considering the situation. Regarding the Somali region, the budget is at the central level. In the {region} region, all bene[implementing partners] from that and they have a revolving fund. Concerning the purchasing issue, the region is the one that pouches and delivers to the woreda.

I: Is it because of the absence of health care financing?

R: One there is no health care finance and the other the health centre themselves should come and facilitate. There few woredas in the [zone] that tried to facilitate by themselves. The woreda came here and facilitated. The woreda might have five health centres and Pharmacist from the woreda came and facilitated all based on their interest. This kind of structure should change like other regions to be effective.

I: What is their reason for not coming and processing though there are pharmacists and health centre heads?

R: There is no budget and they are not managing it well.

I: Did not the woreda allocate a budget if it has five health centres?

R: They might allocate the budget but how do they know the medicine that the health centres are looking for? If the health centre has the right to go to [RESUPPLYING HUB] to process by themselves it is one option but it is the woreda health professional (pharmacist) who decides for the health centers and provides what he thinks is necessary rather than the interest of the health centers. It is the woreda who decides. This is practiced in the [zone]. By the way in the other zones, it is decided and processed by the regional pharmacist.

I: Based on what circumstance?

Respondent: The region said that the health centers send their interest only.

I: Do they bring any evidence in this case?

R: They brought lists but we are not sure whether this is their own or the health centre's request. We do not want to get into detail about that issue. The main thing is that the structure needs to be changed and the Ministry should work on this. There is no decentralization, the whole things are occupied by the region.

I: The health facilities did not come to you to buy commodities.

R: Yes, the woreda or the region performs that.

I: Is the region that took and stored the medicine to the woreda?

Respondent: The region is processing the medicine as we process by the name of the woreda.

I: Does commodities taken and kept in the region or in the woreda store? Do they directly take the medicine from the woreda or from the region?

R: Most of the time the medicine is kept in the woreda.

I: So, it is less chance to address the medicine to the facilities, and there might be a problem related to this.

R: Yes, it has less chance and there is a great problem.

I: What about the RMNCH products our main focus is related to RMNCH and there are commodities, and supplies that are purchased, whoever purchased them these things should be delivered to the facilities. Is there RMNCH commodity they purchase from you like antibiotics, ceftriaxone, gentamicin, and others, do they buy these from you?

R: Is it for the RMNCH program or what?

R: First, we receive the breakdown except that these resources are prepared for purchasing purposes and if they are filled in the RRF we provide them based on their consumption. They can buy additional amounts if they need whether they use for that purpose or others as it is antibiotics. Regarding Amoxicillin or dispersible tablets always we are not stocked out and we have an adequate amount. Only we need is the filled RRF. For instance, buying Amoxicillin suspension is difficult to use for that program but that might be used for the outpatient.

I: From the points that you have raised such as quantification, forecasting, and budget issues, what do you recommend to be done? What are the solutions to the problems you have mentioned? You have raised different problems including the budget at various levels like the woreda, zone, and institutions that affect the system so what would be your suggestion to improve this process?

R: There might be technical issues related to the budget, what I have recommended is that it is advisable to increase the budget. The questions of how, why, and under what conditions the budget needs to be increased have to be answered by the concerned bodies. The other thing is that the points that I have mentioned are very significant points that should get attention and get into practice. These are using revolving funds, and health finance care, like other regions, and if this region takes the experience from other regions with the concern of the Ministry health and makes this process avail in the facilities by pushing forward; it is the first step to improve the process and makes facilities self-dependent. When we say the facilities become independent, they have the right to meet directly with the concerned bodies, and you can access accurate data and they are near to us. What we are doing now is that we request them to do the forecasting and the medicine

is delivered through the woreda, this creates discomfort. Health facilities should be capacitated and making them independent is the better solution, this is my recommendation. Particularly, they must use a revolving fund means that the health care finance is something they need to sell what they have and buy the medicine by the amount of the money they have. For instance, one facility has a budget of about 180 thousand birrs, it cannot face a challenge related to medicine as it can be sold that amount of medicine and buy that amount of medicine using that money. But the current practice shows that the budget is allotted by the woreda and they sold the medicine and refunded it back to the woreda. So, it does not have any effect, unless [RESUPPLYING HUB] has delivered a medicine they simply sit idle, they cannot buy a single medicine. In the {region} region this kind of situation is not there if we need any medicine for an emergency case we take the money from the health care finance and buy the medicine. Even they use it for internal service improvement, and they improved many services using that money. This is one of the major recommendations for the region.

I: Well, How is the ability to use the third parties institutions, when you need additional fleets though you told us your fleet capacity is good? Do you revise the back order? Are you delivering based on back-order management or are you looking emergency order from the facility?

R: Okay, let me start from the last question; yes, we manage the back orders; we have a list for the back order we can see. Sometimes there might be a medicine that might not come on time and when the RRF timetable is reached we are forced to deliver, after we have completed that, by collecting every facility back order we work on it. Regarding emergency cases we are always ready; in order not to face emergency stock-out problems, the first thing we inform them is that, they need to have emergency stock for a maximum of four months and a minimum of two months based on the IPLS strategy. If the report is performed exactly, honestly speaking you cannot face a shortage unless that medicine is stockout. If they face that challenge, we do not prohibit them. What we are requesting is that they should have an issued letter from the concerned body and model 19. They need to take it by their own vehicle if they have an emergency case.

I: Is it because of the emergency?

R: Yes, it is, if it is back order, we deliver that. However, this happened because of their own problem, they simply sent their request without checking their stock. It is obviously recommended

to have a maximum of four months' stock. If the problem is ours, we deliver the medicine to the woreda. If they took the medicine this month and if they request that after a week it is not our problem it is the report, they send that is why we tell them to come and collect the medicine that they are looking for. For emergency orders, what we have decided as a management, they must bring an emergency order letter and model 19th. Concerning Fleet and root optimization, we are performing root optimization yearly. We are doing this because now the roads are under construction to be asphalt in some places, and we are considering these things. Now, we have new arrival vehicles and there are vehicles that are unable to drive out of asphalt. We shift our vehicles based on the road type. We divide the roads into two groups the own which is structured by the machine and the other road which is made by the hands of the community. Here in Somali, you can go in any direction unless you have someone to show you by dividing all of them and considering the season, we optimize the root. There are vehicles that we use only for the rainy season, but we stop in the other seasons.

I: Do you have adequate vehicles?

R: Yes, we have. Now, we have a new arrival, and [RESUPPLYING HUB] is in a good stand. The question that I did not respond to is that the delivery from the woreda to the facility, honestly speaking it is not [RESUPPLYING HUB]'s mandate, and we did not check. If we check we do not have any mandate I do not have any information about this.

I: What is your understanding of the distribution system from the health centre to the health post?

R: Instead of arguing always the last mile delivery is the best solution. If we bring the last mile delivery to 100% , you directly meet the health facility after that there is a customer.

I: Therefore, road infrastructure and facility readiness are the two main hindrances that blocked the last mile delivery from becoming 100%.

R: Yes, if these two things are solved, we reach 100%. Recently, an organization that is called [IMPLEMENTING PARTNERS] selected this branch, and in collaboration with them and their support surely, we will reach both 100%. We had a communication, and they started the process, and we signed the MOU.

I: What alternative transportation system do you recommend to transfer the medicine from the woreda to the health facility? [RESUPPLYING HUB] has a plan to address the medicine to health facilities, till that day, what kind of transportation choices are there, as this environment is a pastoralist?

R: Now a day, every health centre might have an Ambulance, though it is not advisable to use an ambulance for this purpose. From my previous experience at the health centre, I was working with them and in the current situation practically the health centers are using ambulances to collect from the woreda and to distribute from the health centre to the health post. Though the ambulance's purpose is not that, I do not know, maybe it is not 100% restricted to use ambulances in other situations. As we have observed, they came and collected by ambulance; this is a temporary solution. Sometimes when the emergency has occurred, this thing might create obstacles. Therefore, it is not an easy thing that I simply recommend, but it requires a strategy. Under this condition there is a question of fuel and other related things, so things need to be going on in the existing way.

I: As you have mentioned earlier you are supporting the health centers in filling out the format, is there any problem related to the IPLS that occurred in the health centers? Is there a means that you go to the woreda and health bureau to support?

R: We have a supportive supervision.

I: Do you have, when are you doing supportive supervision?

R: I think it is two times a year. Most of the time supportive supervision is conducted with the forecasting team, but it is integrated with the health bureau and we go with them too.

I: What is the challenge to strengthen IPLS in the health centers?

R: After the withdrawal of it is degrading. When I was in the facility and it was the first time I was working at [district], it was very hot, they had close contact and supervision, what I have observed now is that the quality, quantity, and professional understanding are degrading. You cannot get a facility that uses IFRR except RRF. Currently, it is totally zero and no one requests them. In order to get the medicine, they are forced to fill RRF. As to me, it is better to be handled by the project itself or by the Ministry of Health. It is mandatory to provide continuous refreshment

training and supervision because there is a huge gap. The first is that the trained human power that who know IPLS well are withdrawn, now it is very rare that provides training on IPLS. I have heard that, IPLS is given in the school, but I am not sure whether they have graduated. Beyond that, actually, it does not require much training, but it requires follow-up. For example, [RESUPPLYING HUB] is supporting to make them fill the form appropriately and serve them properly. There is no anything that is said to [RESUPPLYING HUB] to support IPLS. But if it has belonged to [RESUPPLYING HUB], I am sure [RESUPPLYING HUB] shows an improvement in it.

I: How does this is related to the continuous commodity availability and wastage product management?

R: There are many wastage rates and if an individual correctly fills and gets into an emergency they bring a car and take the medicine. There is great wastage in budget. The first thing we are forced to do and the other is that they are at emergency they come and say there is a budget mistreatment for this case since this happened because of the wrongly filled form. The second is that if they did not look at their stock and ask after four months the medicine would expire, as he/she might say it is out of stock (no), IPLS is the real solution for this case. Especially every two months they need to count and know the available medicines in their warehouse. If they properly fill the form, it is possible to manage easily.

I: Did you see expired or over-dump medicines during your supervision?

R: Yes, there is. I do not remember the name of the woreda to that we gave the medicine and two months after the first delivery they came back by emergency means, but when we look our list the woreda had taken many doses of the medicine. By communicating with the focal to conduct inventory the store lastly, they have found many medicines in the store. We have this case in case of an emergency. Though we are not going to the supervision I believe there will be findings. Unless we implement IPLS, we are at risk.

I: Okay, what are the hindrances to implementing the electronic information logistic management system that exists in the institutions?

R: We properly use sites that are found in [ELMIS], the other was Mbrana, and it was in Woreda and not functional. **[elMIS]** is at the woreda level. The system is designed in that manner. We have

reported in the meeting. As we are saying direct delivery, we cannot do that, if we functionalize the health centre under the woreda the woreda becomes disfunction. We do not need to send a report as we have directly met the facility by the VRF. So, it is better to turn to the facility.

I: Is the [ELMIS] functional?

R: Yes, it is functional. Even it is developing to integrate with ERP. [ELMIS] is directly synchronized and ERP presents their request and simply you can process it here.

I: Do you directly use the RRF that has been generated by [ELMIS], or do you use it manually?

R: We have received and observed and found some items which are exaggerated. In this case, we manually tried to manage. This output occurred because of the wrong entry of the data. Sometimes it brings a maximum medicine which we did not provide, at this time we manually correct that. If you simply give based on what has been asked like 10,000, what are you going to give to the other facilities?

I: Is there anything that they are raising for inability to use [ELMIS], like lack of infrastructure, the absence of a generator?

R: There are very few in number that use [ELMIS], it is around eleven facilities that use [ELMIS]. These are health facilities that are located in RT sight.

I: Facilities that used [ELMIS] are in the RT sight, what is the reason others not using [ELMIS]?

R: I think during the selection they used this system.

I: Among these eleven facilities that use [ELMIS] is there any interruption they have got?

R: They do not have such a case, and they are properly utilizing that.

I: Okay, as you mentioned earlier some institutions unable to conduct stock analysis because of this they came by the emergency case and they have also a medicine that is expired. Do you have the experience of transferring and sharing the medicines that are dumped in certain woreda?

R: We simply provide our recommendation. For instance, a national stockout medicine that is very sensitive like TB, or RH is dumped in a certain facility in this case by communicating with the regional bureau we request to be redistributed.

I: Is there this experience before?

R: Yes, there is.

I: As you informed us in collaboration with the health bureau you go out for supervision. How do you support and conduct a follow-up, and what is the feedback mechanism?

R: the feedback is given through the regional health bureau. The supervision team went out separately from the vaccination team and if it is a program the forecasting team is going for the supervision. After the supervision, there is a review meeting, during which the supervision analysis is displayed and each concerned body is requested to forward comments. If there is a comment to forward, we forward it, and if there is the suggestion that [RESUPPLYING HUB] needs to take we take that comment and work on it for future improvement.

I: How do you provide the feedback, is it aurally, or in a written form?

R: There is a checklist that helps to provide feedback immediately. In addition, that finding will be well discussed in the review meeting. Based on the discussion we developed an action point and put our signs.

I: Is there the experience of providing a letter that shows their strengths and weaknesses?

R: The checklist itself is enough; I do not think it requires an additional letter. For the other we use letters. Monthly, we use letters to provide feedback to RRF.

I: Is there any improvement that has been made after providing feedback related to RRF? Timeliness, accuracy, etc.

R: Yes, there is. Especially presenting the RRF on time. Because of the ARP transaction i.e., the transaction is from HMIS to ARP; we have a month gap and we did not distribute, and also the facilities are not requesting as we earlier distributed four months resources. So, the timeliness is showing very good progress.

I: Is there any bottle-necked factor to strengthen the feedback mechanism? Is there anything that you recommend to be improved in the future, particularly decreasing interface and data sharing cases?

R: To me, it is better to continue in the current situation, as I have informed you, we are using the WhatsApp platform to provide timely feedback and make corrections immediately this process is very easy and helps us to solve the problem on the spot.

I: who is the member of the WhatsApp group?

R: All the regional concerned bodies, all [RESUPPLYING HUB] personnel, we are there, all zonal focal persons, coordinators including the deliverer, and stockman. If there is a product error in the packaging, it will be reached to them. Even if the second-stage vaccine is added to the first vaccine package, it will be identified by them. So, it is visible to all who are in the group, and if we work on it. It is a very good means.

I: I think we are the way to finish our questions. How are the data from the woreda, the zone, and the region strengthening, and how are they using data for decision-making? As you know our decision is based on the database that we have. How is the experience of using data for a decision-making purpose?

R: Regarding [RESUPPLYING HUB] we do not have any problem. You can produce your data according to your own report, so have the right to do that. The problem might be the utilization issue the system is advanced, and it is helpful for decision-making, and we do not have a problem with that. The gap that I need to raise is that, if it is possible, we/[RESUPPLYING HUB] need to get permission to access the DHIS 2 reporting system. Why I said this is that things are not only managed by RRF, perhaps there might be mischief on these resources and we have got a direction from the Ministry; for instance, we are requesting malaria patient mobility data, and they are giving the mobility data in parallel with the report. If they did not give us that, we did not give malaria medicine. Thus, if we have permission to access DHI 2 we can easily get the data there.

I: What is the gap in that situation, as you access the service data you are trying to see the data based on scope, strategy, and others.

R: These need to be shaped.

I: What is the obstacle that you face to meet the health facility?

R: We do not have the access. The permission is provided at the regional level. I think the system should be integrated with our system; this is also my inquiry. If you asked to bring the data that

have been fabricated and shaped, it is better to access the data that the Ministry of Health follows up. We need to know the data and we need to provide by doing our consumption ratio.

I: In my understanding, if the data is sent it has to be triangulated with the service delivery and related cases. What is the current practice of health facilities, are they simply sending two of the reports or have they tried to triangulate?

R: Some send simply by filling, but others are doing this by reviewing their archives/documents to identify the exact number of malaria patients; TB is obviously known, unless there is a new entrance, we did not request them. Based on this we are managing the situation.

I: According to your view, there is a discrepancy between the service data and others if there is a problem does it create discomfort in your distribution activity?

R: Yes, it has an effect. By the way not only mine, but if the 19 branch has a similar case it creates another challenge. If it is the only problem of our branch, it does not have that many difficulties, but if this case existed at the country level it brings a problem to the country.

I: **If you have heard, there are various teams or platforms that are formulated under the health administration structure in order to solve these cases these teams are performance management teams, quality improvement teams if these teams are found in health centers, health facilities, and hospitals what is the interface that you have with them?**

R: Concerning the health facilities, it is known there is a gap. If you establish PMT., they should evaluate the RRF. Every report that is released from that facility should be repeated. The question is, is it practical? No, an individual might write the report and send that so it is the individual's interest that is reported. To protect such cases the system should be strengthened.

I: Though teams such as PMT, and QIT are found in all institutions, what is the challenges not to being effective?

R: I think it is because of weak leadership. If the leadership manages in a good manner, it can be practical. So, if the challenge that revolves around leadership is solved, I believe it can be functional.

I: Well, we have tried to raise many points, when we tried to sum up this there were intermittent stockouts, last man delivery at the health post. In general, if you are asked to mention three obstacles what are they and to solve these obstacles what are the mechanisms?

R: You can find from what we have discussed so far. The first one is the leadership, the commitment from all parties and at all levels; There might be a part that we need to take; we ([RESUPPLYING HUB]) might have a problem, and we are not saying that we do not have a problem.

I:probing: By what case?

R: There might be a commitment problem on the side of the workers related to the bene[implementing partners]. Though commitment is not supported by a bene[implementing partners], everyone needs to have a personal commitment. The second is the leadership in the country or at every step properly manage leadership no problem cannot be solved. I have remembered only two; I think that would be enough.

I: Do you know a best practice that can solve the supply chain-related issues in the regional health/ health facilities? If you know currently, share it with us if not you can suggest. For the future. For instance, I have recorded one best practice which is the WhatsApp platform that you are using for feedback and support.

R: We have very close communication with the regional health bureau. They are always ready for any support we need. All the leaders are positive for personal communication or else. It is communication that brings a solution to every challenge. Sometimes when we have a problem related to our vehicles on the road or if the vehicle is stacked with mud immediately communicate and adjust ambulances from the woreda so the medicine is shifted and delivered to the facilities on time. Because of the smoothness of the communication, we solve the challenges that we face in supply chain management every day. I am not sure, how it is scaled up to a higher level. As a rule, [RESUPPLYING HUB] should have a good relationship with the regional and zonal health bureaus. It is supportive of the supply chain system.

I: Thank you, we have finished our question, if you have something you want to say lastly you can.

Respondent: I have said a lot, but I wish you to change our discussion to practice.

Interviewer: Thank you for your time.

Transcript 12

**Place of interview**	[region]
**Role and response.**	KII-A-06
**Sex**	[sex]
**Educational status**	Master’s degree
**Experience**	15 years and above
**Type of interview**	KII
**Interviewer**	[university Lecturer]
**Transcriber**	[university lecturer]
**Time**	01:10

I: Thank you. Can you tell me about your responsibilities and role in this organization?

R: I am the Director of the mothers, Children, Youngsters, and Nutrition System [region] Health Bureau.

I: This interview mainly focuses on supply chain management, and I might ask you a few questions before that. I request you to be calm and respond to those questions. Having said this, how do you explain the effectiveness of the supply chain management system? Perhaps it is effective or not starting from the health bureau to the health post. Can you give me a brief explanation about this issue? How do you look at this?

R: Thank you. Resources are vital to providing proper service in the region and at the country level. Supply management system is taking place starting from the health centers. The health centre requests it every two months using RRF format. These formats come from the health centre to the health bureau, and we present this request to the [RESUPPLYING HUB]. According to the [region] region, we tried to address the necessary resources directly to the service point, i.e., health centers and hospitals. This is the way that the commodities tried to be addressed. At the beginning of the budget year, we tried to quantify and forecast. The forecast and quantification are performed based on the service and workload they have, and the health bureau approves this; after that, we present it to [RESUPPLYING HUB]. When we look at the system's effectiveness, we do not face a shortage challenge in accessing the necessary resources if we look at the vaccines this year. Similarly, we tried to send the necessary resources to the health centre as there was a shortage of one or two antigens in 2014 and 2015.

I: What are the antigens that you had a shortage of?

R: In 2015, there was a big shortage of BCG vaccines, but this year (2016), we did not have a shortage of resources, and we can say it was in good condition this year.

I: When we discuss the effectiveness of the system, we evaluate it by addressing the commodity to the health centers and the concerned bodies' ability to correctly fill out RRF timely and address that form timely to your bureau or to [RESUPPLYING HUB]. What do you say about this issue?

R: In addressing the form, the time and the completion have their problem. The vaccine is filled with VRF, and filling completion in time is problematic. Moreover, in the other parameters, such

as accuracy and legibility, gap is observed. Periodically, feedback is given, and sometimes correction measures are taken. Various capacity-building training sessions have been given for improvement. Till now, we did not believe that completeness and timely finishing of the activity is not in a good position, but there is no shortage of resources about vaccination. Even though there are certain problems in managing tasks in time and completeness, we tried to communicate through a phone call to correct things, and we have good communication with [RESUPPLYING HUB]. However, the request had a problem: we did not face a challenge to get what we required from them, and individuals tried their best. Particularly the great achievement this year about vaccination is we increase our eligibility up to five years. From the current issue, which is the Zero dose and Measles, the case which occurs here and there, we enhance our eligibility for up to five years. For this, we need commodities, and we have adequate resources. Only the completeness and the time issues are the cases that we need to mention. There is a maternal-related commodities shortage. We have a serious Family planning commodity shortage. For instance, we have only 45% of the family planning commodity we need for eight months. Even though we did not evaluate the yearly plan in general, and I do not have full information, we have a critical problem, particularly the huge shortage of implants. This creates a good governance problem, and till now, we have been unable to resolve it, and it is continuing as a problem. Thus, in a family planning commodity, there is a serious gap. The maternal service delivered is free of payment in the region and the country. This means the government is filling and replacing the necessary resources periodically, but the reimbursement system guideline is not prepared yet, which burdens health institutions. Considering three years of data on the Hawassa referral hospital maternal supply expenditure, it is about 16 million. This amount has to be reimbursed by the region and the country. The delay creates a great burden on the health institutions and the mother's health condition. This is a great challenge, and the gap continues in this way. Similarly, maternal service is given freely by health centers, primary hospitals, and general hospitals, but the cost or the fee has not been replaced. Because of this, the leaders face a significant challenge. The other thing is that although you have a budget, there are commodities you cannot buy from the market. When the woredas are coming to purchase commodities from [RESUPPLYING HUB], the commodities they need are usually in stockout condition. The stockout average is nearly 60% up to 70%. Because of this, they are forced to purchase from private suppliers. If it were from the government that the purchasing takes place, they could get much number of items but would be forced to buy a small amount from

private suppliers. They are also enforced to release operational and transport costs. This becomes a very significant challenge for the woreda health offices. They did not get an adequate budget from the government, and their movement supported only by internal income, with most of the internal income budgeted for free services. The government is budgeting very low amounts or nearly zero or no reimbursement. Therefore, the issue that highly affects our health institution is supply chain management and support.

I: **Thank you very much. You have tried to touch many points, such as the budget, supply, and lack of access to buy commodities in government stock rather than private organizations, perhaps to provide the required facilities to all health organizations sustainably. How do you evaluate the infrastructure issue, which means they have adequate storage and transportation? For instance, are health posts taking medicine from the health centre? Do they get it in a timely manner, and how do you see the opportunity for the health centre to access the necessary commodities periodically?**

R: Our facilities did not have a storage capacity problem**.** They have a distributing unit and a store. Even though they have a store, the medicine has stocked out. Because of the lack of storage, there is no commodities that they refuse to accept. Concerning the cold chain, this year, we distributed more than 291 refrigerators to health centers, particularly those that can work solar. They have a storage that can hold adequate vaccines and medicine and have a good storage capacity. About the health centers, some medicines went there: family planning commodities, child health intervention, and antibiotics. Our health centers required maintenance.

I: What kind of maintenance do they (health posts) need?

R: The health posts, there are about 511 health posts in the [region]. Most of the health posts, nearly 96 to 97%, had been built by the local carpenters and the available resources around the year 1996/97, and some of them at the beginning of 1998 and had been built in the campaign, so they required renovation. According to the health extension road map, the health centers are divided into three sections: merged, basic, and comprehensive. If we look at the comprehensive health posts, only two are kept clean. It is around Aleta. One is under construction, and it requires a budget; one comprehensive health post requires around 16 to 17 million birrs from the experience of last year's bidding. According to the [region], we must have around 67 comprehensive health

posts, and we cannot afford that, so it is better to maintain by mobilizing the community. Because of this, we have maintained many health posts through this program this year. The types of maintenance: there are health posts, their walls are washed out, and there are others that pour rain, so we started maintaining them, but they are not finished yet. In the health posts we had maintained, we guess that they are delivering good services, and we have seen that. Overall, it is not the storage problem that the health centers face. It is the lack of the required medicine.

Concerning the transport, we are following direct delivery to the health centre. Whether it is emergency medicine or to purchase a program. The normal medicines are delivered directly to the health centre. In this case, they did not face a challenge; however, there is a transport shortage, and some might come by ambulance and collect emergency medicines. Few woredas have a car and use it for purchasing purposes, from [RESUPPLYING HUB] to the health center. The great challenge with transporting medication from the health center to the health gate is Almost all our health gates did not have motorcycles. When they took the medicine, they went on foot and carried it by hand. If a kebele officer is with them, they go with them by holding the medicine to the health gates. This is a critical issue, and it has been repeatedly reported at the regional and federal levels, but still, no solution has been found. There is a problem with going from the health center to the woreda and from the health center to the health gate to support it is a very challenging issue. When there was a campaign, the health officers went to the nearest health centers on foot, but for the health gates found at a very distance, they rented a motor bicycle. Sometimes, they might borrow from other sectors, so the transport case is a critical challenge we faced.

Concerning human power, when we look at the [region] in general, we do not have a human power shortage, but there is a specialization in which we have a shortage of workers. There is a pharmacy, laboratory, and midwifery health officer's problem; without conducting a critical study, the pharmacy and laboratory training was held for five or six years because of the adequacy of pharmacists and Laboratory officers. Because of this, we have a shortage of these officers. You can get a clinical officer in the dispensary instead of the pharmacist. Sometimes, a person who is a storeman might be clinical or other professional. The shortage of these professionals exists in health centers and woreda health institutions. The training has started, but as it was held for some years, the burden is still there. Similarly, there is a shortage of laboratory health officers. Concerning Midwifery, when you go to the highland area, there is a shortage of midwiferies, but

when you come to this place, there is excess midwiferies. This is because of the distribution; we tried to make corrections in 2016, and we tried to assign and make a fair distribution. We have done well with some professionals but still have a problem with others. In general, except for these professionals, there is also a shortage of bioengineers. There are two bioengineers, and there was only one for a long time in the region. We recently had two in rotation, about three or four months. When we go down to health centers, there is a shortage of bioengineers, as there is a lot of medical equipment that needs maintenance, and our refrigerators, because of the absence of professionals, are not appropriately maintained. This case is highly connected with the shortage of spare parts and professionals. In addition, we have adequate doctors and public health officers in the central towns of each woreda. Clinical health professionals and health extension professionals. Concerning these professionals, the region did not have a shortage. We do not have a problem submitting the RRF format promptly, but there might be an accuracy problem when filling out the forms. Some officers copied last month's form; some tried to fill it without checking the amount of equipment they had and sent the form. Because of this, we observed that the number of vaccinated persons is higher than the vaccination they received. Fearing this last year in the budget year, we requested them to give us the vaccine at their hand, and how much vaccine is delivered, and when we checked that the report of some of the health centers shows that the vaccination service they provide is more than the vaccine they received this might be information problem. The other is that they did not check their vaccine from the beginning. This kind of problem might happen because of one or two of these issues, and this year, such a problem might not happen again.

I: What is the experience of using data for decision-making**? There** are various data and different teams in their institutions, such as health centers or health posts. How do you see their ability to use data for decision-making since the database information should support the decision? In addition, is there an adequate professional who has received enough training in supply management systems, or is there access to training?

R: Concerning using data for decision-making, it is mainly workable where the data is generated. It is vital for the person who collected the data to use it in decision-making. When we look at the performance management team (PMT) starting the health gate to the region bureau, we see a huge gap. For instance, if we look at the region, according to the **PMT** standard, they perform a monthly

evaluation; no, they did not do that. The zonal administration is not as strong, so let’s look at the woreda. The zone was recently formulated, and they provide a coordination activity, but they did not have a full structure. Unlike the previous south zones, which have power, a council, and the right to make decisions, I said, let’s pass. When we look at the woreda, we see that they must periodically evaluate not only PMT but also RDQM, and they need to triangulate the data, but there is still a gap here. This problem has occurred in the lower health service providers, too. If the evaluation does not perform well, it is impossible to use data for decision-making.

Still, we did not start the hepatitis birth dose it is by four or five sources that the service is provided according to [region] as a pilot and it is not stated yet, but we received a report concerning this. There are health centers that directly send a report about hepatitis this happens because of the inability of the PMT to evaluate the activities. There are a number of death reports which is not really occurred. In general, the training of using data for decision-making by PMT should be developed and this thing should be led in a proper way we as a bureau make things ready for the coming year 2017 and we have prepared the TOR and will cascade for the concerned health. So, we have a strong stand on this issue, as we observed there is a gap in this condition. Concerning capacity building we have tried our best and Marie Stopes provided us with a blanket, and we provided training for all woreda logistic officers, but there is a turnover because of that there is a need to be refreshed. As I have informed you instead of a pharmacist there is a clinical in this case there has to be refreshing training. These issues were raised every time and there was no training that was given by our bureau and other partners many complaints are coming about capacity building training. Professionals who are working in this office not only the woreda, health centers, and hospitals in order to manage the logistics system by itself and support the health centers, health gats, and hospitals to make them effective in their logistic management, such as IRF and the health posts to man and clinical cost form let the health gates use forms properly, and using the income and expenditure model in relation to this it is required a capacity building training.

I: **Thank you, you raised good points. How do you evaluate the impact of the pharmacy in the PMT and what kind of data do pharmacists bring, for instance when you have a meeting what kind of data do the pharmacists present? And what is their role? Primarily let’s discuss about supply chain management system's impact on the organization and the information that the pharmacists bring when they come to the meeting and then we will try to discuss the**

bottlenecked factors that affect PMT not to use the data in a supply chain management this will help us to develop an intervention.

R: In this part, my partner will say a lot. What is the role of PMT and what do they bring when they come to the meeting, the first thing that we do at the director level is evaluate the monthly, quarterly, and yearly report during the evaluation the pharmacists participate in that meeting, in the absence of the logistic director we did not make any evaluation at the department level. During this meeting, the supplying case was thoroughly raised and discussed. We tried to evaluate the challenges and the hindrances of supply chain management in relation to vaccines, commodities, family planning, and maternal issues and we discussed how to avoid these challenges. Concerning the delay of submitting the format, sometimes the delay of the request and sometimes the request is sent through the region like the nutrition commodity Plump nuts, and child health antibiotics the accomplishment of these is present, and which supply and which commodity face a critical challenge; in this case, they actively participate in the discussion and forwarding a solution. When we pull the woreda health professional to conduct an evaluation the woreda logistic coordinators are also part of the discussion. During the discussion time, the woredas presented what they have on their side and we also presented what we have in our side we had a discussion, so detailed LMIS, GRF, inventory, maintenance the forecast and other issues they reported in detail and in-depth then we discuss and design action plans and we depart. There is a jointly supportive follow-up. This supportive follow-up is performed with this department, the Diseases Prevention Bureau, concerning malaria commodities.

I: Is there a skill exchange between the professionals who have released the job and the ones who are joining the job? Did you have induction training for newly hired professionals at the region or the woreda level? What can you say about this issue?

R: The employment is at the woreda, and the region has no right to hire. The region and the partners give the capacity-building training. If the professional did not take any training based on the criteria, the professional might get capacity-building training. Starting in 2016, every institution had a morning session where professionals learned from each other by presenting a case. We have begun in some health centers, but I do not think it is going on now; in the new budget year, it is considered one of the platforms. It isn't easy to train and equip everyone by preparing training and

mentorship. We were thinking of continuing the morning session by adjusting the necessary things. For the position that required managerial skills, we prepared a four-day training for Woreda main and vice leaders and hospital leaders here in Hawassa, and we provided them with leadership training. One of the training contents was commodities, and they were trained on how to manage the commodity by the way the [RESUPPLYING HUB] main CEO gave the training about commodities. The capacity building training is not only for newly hired professionals; the professionals with the experience were also part of the training. We focus on the leaders because the professionals are looking for refreshment training, or there is on-the-job training. There is also mentorship in collaboration with the Ministry of Health. Using this, we are working to empower the professionals through various ways.

I: As you have touched, how do you evaluate the connection between the [RESUPPLYING HUB] and the institutions based on providing the requested commodities timely? What are you looking for in this context?

R: There is a positive relationship, as I mentioned earlier, but because of the absence of program medicine here, they did not obtain it based on the requested amount. If you remember what I informed you earlier, we have gotten 45% of the required medicine for eight months. Usually, when they come to buy medicine, about 60% to 70% of the purchasing is done by private institutions. Nothing creates discomfort between [RESUPPLYING HUB] and Health facilities, and the lack of supply is the only problem, but [RESUPPLYING HUB] has a good relationship with the facilities.

I: When you work in collaboration and when you evaluate reports together, for instance, family planning. In your evaluation, what is the root cause for this issue, i.e., the shortage of the provision and supply? What will bring your collective evaluation?

R: They did not have a problem in requesting, but the problem is the inability to access the supply that they are looking for. After passing the bureau, we directly contacted the [RESUPPLYING HUB] focal person, and we had a discussion in our office in the presence of the bureau Head. Many cases were raised, but I do not want to raise them here. The problem/ shortages occur because of the causes that are well-known by the government, and the shortage happens because of the well-identified cases, so we have already moved to that extent.

I: How do you see the facility's capacity with forecasting? Sometimes, we observe unnecessary requests or requests that have been asked in small amounts, which is not adequate for the quarter or annually; how is their skill in filling out the formats in a proper manner?

R: This issue is better explained by [expert], a logistics director. Here, they review the request, looking at the amount and unnecessary requests. If equipment is missing, they thoroughly revise it and pass it on to the concerned bodies.

I: RMNCH resources are directly delivered from the [RESUPPLYING HUB] to the facilities without the connection of the region and the woreda. How do the health posts get these commodities from the facilities? In which form or condition do they take the resources?

R: There is a model 19 and model 22 that they received and returned. No one gives them without the model.

I: Most of the main questions are raised. There are a few questions that we are going to ask. You have said a lot about transportation, and it has been noted that most health facilities are direct. It is good, and keep it up. Since [RESUPPLYING HUB] directly delivers the resources to the facilities, do you get any feedback as a regional bureau? Do they give feedback and how is the facilities' readiness? [RESUPPLYING HUB] is looking at many things, such as formats, meaning that the RRF should be completed on time, storage should be prepared ahead of time, and there should be trained personnel at the health facilities. Based on the [RESUPPLYING HUB] direct delivery perspective and health facilities' readiness, how do you see these conditions? [RESUPPLYING HUB] is expected to inform the health facilities about the direct delivery ahead, and it is obligatory to deliver the resources on time. If there is feedback that comes from the health facilities, say something in general about this case.

R: About the facility readiness, there are [RESUPPLYING HUB] cars, and which care goes in which direction is already known. We communicate the time and the date that cars reach the place where they need to deliver that resource. The region bureau informs the woreda, and the woreda also informs their health center and hospitals; messages are delivered in this way. Sometimes, [RESUPPLYING HUB] faces a challenge in providing the resources because of the absence of the officer, and sometimes, Saturdays and holidays are the factors for the challenge. This is a complaint from the [RESUPPLYING HUB] side. The feedback from the professionals was that I did not get

the commodity I needed. One Woreda might say they only gave me one implant for one Woreda, so there is a lack of resources based on the facility's needs. In some woredas in the highlands, malaria medicine is delivered. This is not the case. This is done out of their request. Sometimes, the [RESUPPLYING HUB] delivery system enforces that if you do not take this, you cannot get that resource. These are the complaints that are forwarded from the [RESUPPLYING HUB] side and the professional. There is a platform where we meet with [RESUPPLYING HUB], and [RESUPPLYING HUB] is a member of the logistic technical group. The medical service director leads this, and the partners are also members. When such a complaint is read, it is tried to manage the issue/case. If the resources are delivered improperly, they are redistributed to the concerned facilities.

I: Does any alternative transportation system considered at the lower health center mean that health post? Regarding the transport perspective, particularly the linkage between the health center and the health post, what should be done, and what do you think?

R: Regarding transport, we have reported so many times this is one of the main issues that is repeatedly reported to the Ministry; The Ministry of Health is reporting that they are trying to solve this problem, and they told us about 500 motorcycles arrived in the port; how many of them were given to the[region] region? No one knows. That is, the motorcycle is directly given to the health centers. Some health centers buy motorcycles on the inside income; if you go to [district], the woreda buys a motorcycle for the health center. We suggest woredas, which can buy the bike and make it available for transportation. The current situation in the region is unthinkable, and most of the woredas are now unable to pay salaries. It is very difficult. There are woredas who cannot buy medicine and cannot decide on a budget. We request the Ministry of Health and the partners. There has been a repeated request, but the response has not been positive. WFP has bought three motorcycles to three woredas, which come through CBHI and others by WASH. By collaborating with the woredas, we suggest they use motorcycles together; this is what we think and recommend. Concerning the supply, [RESUPPLYING HUB] is not feasible for the manual regarding the relationship between the health center and the health gate. It is stated that the health center supports the health gate by the resources. In this case, the health extension should not come as they have a burden task to accomplish. Now, the CVH burden is on us, our package is increased, and they bring the initiatives. Moreover, they also take part in political activities, so they feel overburdened

and bored. The primary health extensions are old. They feel bored, and there is no accountability. When they get him/her under their capacity, the officer who goes to support the health extension neglects that officer. The health extension professionals are not expected to come to the health center. Rather, the health center needs to add resources to the health extensions. Is it performed in this way? No, why did this happen? The health center director we assigned cannot manage the organization collaboratively. There are skills, commitment, transport, and accountability problems. I think this is the compilation of all these issues. We want it to be better for the health center to address the resources for the health extension instead of the health extension coming to the health center and closing the health gate. There is a coordinator at the kebele level, and that individual coordinates actively to address the resource timely. I think the health extension officers can work in stable conditions. The main challenge is transport; ordering health extensions to carry medicine is also difficult. The directors did not have the moral to order the professionals to carry the resources. There are many factors, and we must select the one that facilitates the resources. Even though there are small resources that require critical thinking and guidance as a government,

I: Thank you. This might be the last question. What is your view about inventory management? For instance, at what time is medicine counting taking place? How are medicines identified that are expired, medicines that have long and short age by identifying this at the dispensary room? How is the performance of providing medicine that has short rather than long age? And how is the capacity to address medicine to the customers?

R: Inventory is taking place every six months. The health centers are expected to perform inventory every six months. Is it taking place? There are some who are doing it and others who are not. In order to have a full inventory, including the medicine, the medical structure, and the lower health centers, this kind of inventory is not stated yet. Because of this, the vaccine carriers that come in this season vary from the other season, as there is no counting system.

I: Probing: what is the reason for this?

R: There are several reasons that they mentioned; the first is that they use those who are called volunteer health messengers, most of the vaccine is transferred by them. It was suggested that volunteer health messengers be replaced with women volunteers. They feel discomfort, and every one of them takes the container to their home, and no one is returned to the concerned body. When we plan to make the service available by the volunteer messengers, they bring the vaccine carrier.

There are lots of vaccine carriers at the hands of volunteers messengers. When they took the vaccine carrier, they did not sign it was distributed. When we made counting /inventory, there was a problem with including the resources in the health center. And there is a lack of knowledge of the bene[implementing partners]s of the equipment. The other issue is that there are expired drugs in every facility, and it is difficult to avoid these. In the previous time, it was avoided by burning, but now it is prohibited to do that. There was a place to collect these near the airport, and someone lifted the construction. Avoiding drugs that are expired is a great challenge, according to the F[region] region. You might get the expired drug in the middle of the non-expired one. This is because of improper usage of bin cards and stock cards and the inability to perform inventory properly. In addition,

I: We are finishing our questions. How do you see the internal service? For instance, there is MCH, family planning, and a pharmacist; how do they work collaboratively? As you can go down to the facilities to support and oversee, is there anything you have observed? Sometimes, even if there are some resources in the store, there might not be resources for family planning. This might happen rarely in this case if there is no mutual understanding and good relationship, and if there is no strong inventory system, such kind of thing occurs. What is there in that condition?

R: We got resources from the store that are out of stock in the service delivery rooms, but they are in the pharmacy. She said I do not have it here, but we can find the supply in the store. This is the existing problem. This is because of a lack of information exchange among the professionals (each other). If she finished, she said it ended, but she did not check others; this happens in many places. Some work together, but many do not work cooperatively. The request is not even good; the professional who works at material health care needs to bring the RRF, and they are unable to request the commodity that is available at [RESUPPLYING HUB]. The resource is here, but the request should be completed at the lower level, which is the major gap we faced.

I: We have finished our questions on our side, but on your side, enhancing the supply chain management system shows improvement if it stands in this way if this institution works on this case, and if we are working on this issue. The supply chain management structure can be improved at all levels. So, what do you suggest or recommend about this?

R: In the middle of our discussion, I tried to make some recommendations. We observe that these challenges and shortages will not be solved by the government's capacity alone. It requires a multi-

sectorial engagement and partner involvement. The government has a major duty to fill the human power; such a case is the one I mentioned earlier: the shortage of pharmacists and laboratory professionals. Capacity building requires collaboration, and certain partners need support. The government alone cannot fulfil the provision of necessary resources. [RESUPPLYING HUB] is the only governmental resources supplier, so it is better to expand such branches. Suppose the resources are not available in the [RESUPPLYING HUB]. In that case, the purchasing is accomplished in the private organization, but if [RESUPPLYING HUB] has many branches, the availability of resources also has a good opportunity to be gained. It is necessary to enhance the accessibility of governmental suppliers. There is a misunderstanding between the regional [RESUPPLYING HUB] and the central [RESUPPLYING HUB]; for instance, if you ask the planning commodity, the central [RESUPPLYING HUB] says I do not have a problem, but in the regional [RESUPPLYING HUB], you cannot get it. There has to be a well-developed system that governs this institution from the top to the bottom in a good structure. There should be a resources visibility system among the regional [RESUPPLYING HUB] and the central [RESUPPLYING HUB] to check the available resources in the store. We did not have a system that allowed us to check the available resources, and sometimes, we squeezed in the middle of the woreda and central [RESUPPLYING HUB]. The controlling system, auditing, and fulfilling human power should be the government's responsibility. Excess drugs are released from [RESUPPLYING HUB], which a strong regulatory system should govern. This is to strengthen what has been said before.

I: We thank you very much

KII(13)

**Name of Project:**	[project]
**ID Code**	KII- A- 07
**Type of interview**	KII
**Type of informant**	[sex]

**Region**	[region]
**Role of the Interviewee**	Health Extension Worker
**Years of experience**	15 years
**Name of Transcriber:**	[university Lecturer]
**Date of Transcription**	August 2024
**Names of Interviewers:**	[university Lecturer]
**Length of time**	48 minutes

I: How is effective the overall supply chain management system?

R: We receive commodities from [Health Center], but the supply has been decreasing over time. Previously, we had a sufficient amount of maternal and child-related supplies. Currently, we only receive commodities from the health center once a month, and the quantity has been steadily decreasing. Despite our inquiries, the health center heads and woreda health offices have informed us that the commodities are supplied by [resupplying hubs].

For the past five months, we have not received any commodities from either the health center or the woreda, including essential items such as ORS, Coartem, family planning methods, pills, and RDTs. This is particularly concerning given that our area is highly prone to malaria, and we currently lack the necessary drugs for treatment. We also do not have any maternal health drugs, such as injectable and pills, though we do have some long-term family planning methods like

Implanon and Janelle. Following a recent malaria outbreak in our kebele, we received a limited supply of RDTs and Coartem, but now we are completely out of stock.

Our health post serves many clients, and they are increasingly dissatisfied due to the lack of commodities available at our facility.

I: probing: where do you get commodities?

R: We receive commodities from [Health Center], and when supplies are available, we typically use private motorbikes for transportation. During campaigns, such as immunization campaign, the health facility provides commodities using their own transport system. Recently, we acquired a solar electric system, and our refrigerator is now functional, allowing us to store vaccines at our health post. Previously, we relied on health development armies and private motor owners for transportation, and we paid for these services from our pocket.

I: probing: when do you receive commodities from the health center?

R: We have an immunization program that takes place twice a month, so we receive immunization commodities on a bi-monthly basis. For other types of commodities, such as drugs, we receive once in a month.

For the OTP (Outpatient Therapeutic Program), we prepare a list of children and order the necessary commodities weekly, specifically on Thursdays. Commodities for mothers and other supplies are also received on a monthly basis.

We understand that [resupplying hubs] provides drugs to health facilities. Once the health center receives these commodities from [RESUPPLYING HUBS], they inform us to submit a request. We submit our request in written application every month. After approval from the health center head, the store manager provides us with the required items.

Although there is a Requisition Request Form (RRF), we have not received it yet; we are currently informed about this processes orally.

I: How do you compile and report data?

R: The report covers HMIs (Health Management Information Systems), maternal and child treatment, infant health, family planning, and disease statistics. During the reporting period, the health center head provides us with the HMIs form, which we use to compile our report for the 18th to the 21st day of the month.

I: How do you report drug balance like family planning commodities?

R: No, we don’t report in that manner anymore. Previously, we used a BIN card that was updated monthly to show the balance at the end of the month. However, we no longer use the BIN card and do not fill it out now. For family planning commodities, the HMIs require us to report the number of mothers using pills, depo, and implants.

I: why do you stopped to fill out or update BIN card?

R: I think there is shortage of BIN card that is why we stopped to fill out or update it.

I: Have you stopped immediately after BIN card stocked out or trying to use the copies?

R: we use bin card and now updating it, the only issue is we don’t report it for the health center. We do our commodity balance using BIN card

I: What are the challenges that hinder the continuous availability of commodities?

R: We ask the health center head about the situation, and they in turn contact the woreda health office. The response we receive is that there is a shortage of commodities. According to them, the commodities are supplied by [RESUPPLYING HUBS] and are currently undergoing an audit. We have to wait until the audit is complete. The delay in receiving commodities can range from three to five months, which is why we are currently experiencing a shortage. This issue is affecting facilities nationwide.

I: How transport challenges look like?

R: Regarding transportation issues, we face significant challenges. Transporting commodities for the OTP program is particularly difficult. The road conditions are problematic, and during the dry season, we rely on motorcycles, while in other seasons, we may need to transport goods on foot. This situation sometimes results in children not receiving their food. Even when commodities are available at the woreda level, we often can't access them due to transportation problems and road inaccessibility. Additionally, the woreda is unable to stock commodities for the health center during the rainy season. When the road conditions improve and are suitable for motorcycles, we retrieve the commodities from the health center.

I: Do you have enough store and is it appropriate for storage?

R: Since we are a basic health post, we only have two shelves provided by NGOs, which are insufficient for our needs. Despite this, the woreda has determined that this setup is adequate. Ideally, we need 4 to 5 rooms to accommodate various activities, including ANC (Antenatal Care), storage, and immunization.

I: when do you request commodities?

R: After submitting the monthly HMIs report, we inquire with the health center head about the availability of drugs or commodities. For the OTP program, which operates on a weekly schedule, we contact the health center every Wednesday to check on Thursday's schedule. We typically make these inquiries by phone. If the health center confirms the availability of commodities, we then submit a written application letter to formally request them.

I: How about supportive supervision from woreda health office and the health center?

R: Yes, we receive supportive supervision from both the woreda health office and the health center. Together, we visit model households, encourage the community to adopt model household practices, and provide health education to pregnant women during mothers' conferences.

I: probing: any support in regarding to supply chain management?

R: In regarding to supply chain management, that is what I told you previously. In case of drug or commodity management they don't give any support. Rather in case of reporting problem they give us feedback monthly. Like it includes what is the gap, any inconsistency in the report. In case of commodity, nothing is done. Because there is shortage of supplies, so it is not important to give support in without adequate supply of commodities.

I: Probing: do you have now an expired commodities or drugs?

R: Last year, we had a system in place, but it was handed over to the woreda health office. This year, we no longer have it. We had listed all drug items and their quantities, but then they took over the management. Currently, our health post is staffed with four health extension workers, which is adequate for our catchment population and the required manpower. All of us are experienced professionals.

We primarily receive supportive supervision from the health center. The health center head and the health extension worker linkage focal person frequently visit us to identify and address any gaps. During these visits, we discuss the issues and propose possible solutions together.

I: How is the lead time for resupply commodities for the health post?

R: As I told you we receive commodities once in a month. Once, if they tell us about the availability of the drugs, we prepared lists of drugs with application letter then the health center head approves, we immediately get commodities from the store.

I: What training you need to give service in a better way?

R: Yes, we require training in several areas. For instance, if additional rooms are added, we need training on delivery procedures and skills for providing comprehensive ANC visits. Additionally, all necessary formats should be made available to the health post. Although I have heard about the RRF (Requisition Request Form) informally, I have never seen it. Similarly, we sometimes lack the HMIS (Health Management Information System) report formats. Currently, duplicating these formats is costly. In such cases, after submitting our monthly reports using the HMIS format, we request that the health center return the original report to be stored at our health post.

I: do you conduct bi annual or yearly inventory?

R: No, but we update the BIN card.

I: when do you update the BIN card?

R: We issue drugs daily, but over the past six months, we have not received any. When we had sufficient commodities previously, we updated the BIN card regularly and used the first-in, first-out method for drug management. However, we are currently unable to do so due to the lack of available drugs.

I: How do you control the inventory system like minimize wastages since you are four workers working here?

R: Ya…as I told you previously, we update the BIN card while issues drugs or commodities, we all know about the updating of BIN cards. For instance, if I issue four injectable family planning method today, I will register on the number of injectable issued on that date. But, currently we are not using it because of shortage of commodities.

I: probing: why did you stop to update the BIN card for the available commodities?

R: Sometimes because of work overload and negligence. But we know that it should be updated on time.

I: How are supply chain data generated, shared, and utilized for decision-making within the health facilities and health post?

R: We compile and evaluate our report before sending it to the health center. This process involves reviewing our goals and achievements, identifying areas for improvement, and addressing any gaps. We conduct this evaluation monthly.

Regarding the supply chain system, we often face challenges with insufficient quantities. For example, if we receive 20 injectable family planning methods, it may not meet the needs of our catchment area. We provide services on a first-come, first-served basis. While we regularly evaluate our service achievements in monthly meetings, we do not consistently assess the supply chain system itself.

I: What is the reason behind that makes you not evaluate the supply system?

R: we don't have previous experience about it.

I: Are the existing platforms such as PMT available at HP?

R: We don't have a formal system in place. Instead, we receive feedback on our reports when the health center head identifies potential errors or discrepancies in the data. Generally, we address these issues during quarterly review meetings at the health center.

I: How do you evaluate or review data discrepancy?

R: At our health post, we evaluate data for consistency across tally sheets, registration books, and report formats. However, we do not apply the same evaluation to our supply chain management system. Previously, we faced issues with discrepancies and conflicts among health center staff due to the lack of standardized models. We would simply record the drugs received from the health center in notebooks and then provide services.

Currently, we use issue models such as Model 19 and Model 22. After receiving drugs from the health center, one health extension worker uses Model 19 for issuing, while another uses Model 22 for service distribution.

I: What are the major challenges related to supply chain data generation and use in the health post?

R: The major challenge we face is receiving commodities from the health center, which ideally should be delivered directly from the woreda or [RESUPPLYING HUBS]. We need a streamlined

process for receiving commodities or drugs directly from any source. Ensuring the availability and accessibility of all necessary legal formats and models is essential. Additionally, improving the transport system is crucial. As an alternative, providing training for volunteers could be a viable solution, as they are committed to working with us.

I: thank you very much!

KII (14)

		Remark
**Place/region**	**[region]**	
**Organization Name**	**KII-A-09**	
**Responsibility**	**Health extension workers**	
**Sex**	**[sex]**	
**Experiences**	**8 years**	
**I**	**[University lecturer]**	
**Transcriber**	**[University lecturer]**	
**Total Time**	**1:23:38**	

I: Can you tell us in brief about your role and responsibility in [health facility}?

R: I was a clinical nurse for seven years and for one year I am a storeman.

I: Did you work here for all years?

R: Yes, all year I worked here in Moto.

I: Where do you get supplies?

R: Most of the time we directly receive supplies from [RESUPPLYING HUBS]. There is an interruption in delivery. Sometimes unnecessary medicines were delivered. Family planning users need Depo and Implanon, but the medicines that we have are IUCD, Condom, and others.

I: Thank you, so you directly get the medicines from [RESUPPLYING HUBS]. Does [RESUPPLYING HUBS] deliver the medicines on time and the amount you requested?

R: Most of the time [RESUPPLYING HUBS] delivers our medicine to[**health post}** as the road is very difficult to reach our health centre. We got the medicines after five months it arrived in the woreda.

I: Why? Does not [RESUPPLYING HUBS] deliver the supplies?

R: Yes, we are collecting our medicines from the Woreda health offices.

I: What is the problem? Did not you ask the reasons?

R: The woreda are requesting, but no one informed us about this.

I: What kind of medicines do you get from the woreda?

R: Medicines like Pills, Coartem, RDT, and small amounts of TB medicine.

I: No any other?

R: Yes.

I: Where did the woreda get the medicines, and delivered to you, are they purchasing or what?

R: I think [RESUPPLYING HUBS] turns back the delivery system to the woreda, and they get it from [RESUPPLYING HUBS].

I: Currently, [RESUPPLYING HUBS] is implementing a new system which is called ERP. You are expected to implement so in the middle of that they are working on it, did you hear about this?

R: Yes, I have heard they are in a system change.

I: Generally, in your health center you have a store and there are health posts under the health center? What are the reasons for the shortage of medicines to deliver to the health center and the health posts?

R: Our customers (patients/users) are many in number. And the population is many in number. There are six health posts under the Moto Health Center. When we share the medicine for all of these health facilities we face a medicine shortage.

I: Do the health posts get the medicine from you?

R: Yes, they take from us.

I Do you give them the adequate medicines that they requested?

R: When we obtain, we give them.

I: Is there a medicine they requested, but you are unable to give them? You mentioned [RESUPPLYING HUBS], as they are in system implementation; currently, what kind of medicines do you face a shortage? The medicines that clients are in need but they cannot access? For example, family planning, what kind of shortage is there in family planning?

R: Here people need Depo, for around five months no Depo here. We have finished Pills, and Implanon but we have a very small amount of IUCD, however, the clients refuse it.

I: So, these medicines are not available. What kind of medicines do the health posts have not? What are the medicines that you cannot deliver to the health posts in the previous time they were taken but because of lack of the supply that you cannot give?

R: Last week, they took Pills, Implanon, and others it is in this week the store is stockout. It is about four months that we do not have Depo. We planned to go to the woreda to request the supply.

I: Did people come here for assessment from [implementing partner]?

R: No

I: What is the reason for the absence of family planning medicines, though you have few amounts of certain medicines?

R: I believe that, if they adjusted the system they will bring. I think it is a system problem.

I: Do you have a transportation problem when you travel and transport medicines?

R: Yes, there is a great challenge.

I: What is that, can you tell us?

R: When I went to the woreda to bring Plumply nut I was waiting the whole day for ambulance without having my lunch. I waste the whole day to bring plump nut. There is a transportation problem. When I came back the ambulance dropped me on the way as the road was damaged, and I had to be transported by a cart.

I: Are there adequate pharmacy professionals?

R: No, there was one he is on study leave. No midwifery and laboratory. There are clinical nurses.

I: So, there is a lack of midwifery, laboratory, and pharmacy professionals. How are you managing when you want to purchase some medicines? For instance, you are going to Woreda to bring medicine; are you requesting before medicines are stockout or what?

R: Yes, when I go to the woreda, I prepare the list of the supplies and I request the headmaster to give me the letter that is directed to the woreda; then I go with that letter.

I: When did you request the letter for approval of your request? Is it when it is finished or before it is finished?

R: The woreda delivers medicines once or twice a month.

I: While you request, how much of the supply do you have in stock? Is it when you have fifteen days' supply that you request or what?

R: Before the medicines are stockout I request the woreda to resupply.

I: Do you have a record of the amount of medicines that you have?

R: Bin card yes but there is also a shortage.

I: Do you have a record that shows the balance of the medicine which is delivered and in the stock?

R: Is it a bin card?

I: Yes, are you currently filling bin cards?

R: Yes, we fill.

I: Is it updated? Can it show the balance?

R: Yes.

I: Do you get any support from the woreda about the medicine handling, skills, and other related issues to the pharmacy?

R: Periodically, we meet with the woreda.

I: Do they come here?

R: Most of the time they did not come. If they can two or three times in a year.

I: What did they support you?

R: It's a year I have been here, they told me to do things in this way.

I: For example, what are the activities they informed you?

R. Bin card, they informed me that when people are taking out medicines they need to have the issued letter.

I: Are they providing you with verbal feedback or written feedback?

R: It is verbal and they are annoyed.

I: Is there a bin card shortage?

R: Yes, there is. I have a copy format.

I: Who copied the form to you?

R: I did that.

I: Is it by the cost of the health center?

R: Yes.

I: Did they provide you with the supply that you asked for on time?

R: Yes, no problem with providing me the requested supplies.

I: As you told us there is no pharmacist and you are a clinical who represents and works as a storeman, did you get any orientation with how to manage the store, how to deliver medicines, and related issues?

R: No.

I: How did you start?

R: They forced me to get in, and since then till now I have been working.

I: Why you did not want to get in?

R: I was not interested in quitting my profession as I am clinical I want to work on that rather than being a pharmacist.

I: What are the challenges that you faced after you became a storeman?

R: The problem is only the absence of medicines rather than I can work. If the clients face a problem I feel disappointed in case of the absence of medicine.

I: Do not you have a skill gap in relation to managing the medicines in giving and receiving, placing in a proper place, and if I can get this, I can perform better, do you have such kind of question? What hinders you not to working better?

R: If they support me by providing necessary supplies like bin cards for all items I can do better.

I: What are other supports you are looking for?

R: Most of the medicines are in their final stage if they resupply on time, it is very good. If I have got training it is good, now I am working without training. If I get training, I can serve the community in a better manner.

I: Did you get any skill transfer, which means that the professional who is going to leave needs to share his experience, skill, or empower the capacity of the newly assigned professional concerning managing the store did not you get the chance to meet and do these issues before he left for study?

R: No, the night before they informed me that the storeman is going out for study leave so you are the one who is going to hand over, so that tomorrow when the auditing is going on you have to be there. After that he left, and no orientation that I had concerning the store.

I: Did not you get small training with filling bin cards, placing medicine, and how to deliver and receiving medicines?

R: {name of expert] from the woreda came and oriented me, but the pharmacist who left for study did not tell me anything.

I: How do you quantify medicines that are going to be purchased especially RMNCH medicines, how is the purchasing process?

R: The DTC committee discussed the necessity of the medicine to the mother, children, and others, and then they decided on the medicines from the list to be purchased.

I: How do you forecast the next purchase? What did you do? What are the bases to forecast to purchase such a list of medicines, for instance, if you request **NS fluid** to be purchased for the next time, what is the reason for buying **NS fluid**?

R: The case flow is one. There are cases with us, for instance, Typhoid, Typhus, and others like this.

I: Are you purchasing or quantifying yearly? Do you have documents related to quantification here?

I: What is the reason to purchase Fluid for this year which you need for children and mothers?

R: We prepared a plan. If we purchase four times a year, in each quarter we try to purchase the supplies based on our plan.

I: How often did you plan to purchase this year?

R: Every quarter.

I: Therefore, based on the case, service delivered you perform the purchasing. What is the budget challenge that you faced during purchasing?

R: The budget is not sufficient, there is a shortage. The budget and the amount of the medicine are not balanced.

I: Are the budgets allocated by the government and released are equal?

R: It is only from our internal income we purchase the medicine.

I: Is there a budget allocated from the woreda?

R: No, we got it from health insurance. In 2016 E.C. the woreda gave us once but no other budget. They gave us about 5,000, but the cost is very different.

I: You are functioning the health care finance and it will be reimbursed from the health insurance, having this money you purchase medicines though the shortage is there. It is challenging to purchase the requested medicines, as the budget is not sufficient. Did you buy the medicine with the quantity and kind?

R: Yes, the supply that we purchase in a quarter finished in a month. So, it is difficult to buy the requested number of supplies in quantity and item difference. Currently, there is a case so the supplies end early.

I: The first reason that the medicine finishes are because of the cash flow. What is the second reason?

R: As we have a shortage of budget, we purchased a small amount so it is stockout in a short time.

I: What should be done in relation to the purchasing process to provide you with adequate medicine?

R: It is better to improve the budget allocation. The main reason is the budget.

I: Where do you get data for quantification? Consumption, case data where do you get these? Earlier you told us that you are quantifying the medicines, to plan first you are looking at the case, for instance, if the case was malaria you are looking at the **Coartem** consumption, and then you plan for the next. One is by looking at the case what is the other?

R: I do not understand the question.

I: Did not you report medicines consumption?

R: Yes, we use it.

I: Earlier, you mentioned the transportation problem of transporting the supplies from the woreda to the health center and from the health center to the health post, [RESUPPLYING HUBS] brings the medicine up to **[health post],** how do you deliver the supply from the place where [RESUPPLYING HUBS] drops to the health post?

R: If [RESUPPLYING HUBS] brings laborers we use them, but sometimes we use motorcycles. Sometimes they bring in a labor worker and take them by covering the cost.

I: Who paid the cost?

R: The health extension herself.

I: Was there a problem that occurred because of this? What is that problem? Are they always coming and taking by paying, so are they taking medicines before they finish?

R: Yes, they are. For instance, if the Plumpy nut is delivered on Thursday they come on Wednesday and take it, and they spend the night adjusting things for tomorrow.

I: How about the family planning medicines and how do they take them?

R: They take themselves.

I: Are they careering by themselves or by a labourer?

R: If it is heavy to carry, they let a labourer carry for them. If not, they can handle it by their bag or by the carton.

I: Do not you think there is a gap because of this, delay?

R: This is a problem of the road. There is no road that can take you in various directions. In summer most of the time they career themselves but, in the Winter, there are many people and they can use a motorcycle. The health center sometimes gives a ride. Sometimes they cover the motorcycle cost by themselves.

I: Are they paying for the person who drives the motorcycle?

R: Yes, they are.

I: Is there a private motorcycle that gives service?

R: Yes, there are people who rent motorcycles but it might be difficult in the summer season.

I: What should be done to solve the transportation problem?

R: If the roads are constructed, that is good.

I: Which Road?

R: The one you came through is one of the roads if it is constructed many problems will be solved.

I: What should be done? Is there any transportation choice beyond this? Is there a motorcyclist who rents the motor? Is it from [town] or from this?

R: They bring the motorcycle around the health post they are working with them. There is in [health post], and in the other places.

I: Sometimes the health center gives a ride to them, or they rent a motorcycle to take their medicines. Does the health center deliver the supply to the health post?

R: When the health posts come to the health center to take their medicines and if there is a motorcycle in the health center they give a ride. Now, the motor is out of service

I: Did they deliver the supply at the previous time?

R: Yes, they are.

I: Who was doing this, is it the interrelationship focal person who supports the health post?

R: There are six but it is not easy to reach all the six health posts. One or two of them might get that chance.

I: What is the reason to stop?

R: It is damaged. The head has his own motor.

I: Every health center has its own motorcycle, did you count all the supplies, when do you do it?

R: We count the supplies once a year.

I: Did you count this year?

R: Yes, we did. We already identified the medicine that we need to purchase but the program medicines did not count.

I: Why?

R: As the dispensary is in giving birth, we are unable to count but the rest is counted. Rest. The family planning is uncounted.

I: Do you have a form that you filled in the counting data?

R: we don’t have a form we have an archive,

I: Is there the expiry date and other information?

R: Yes, there is.

I: Are there medicines that are expired?

R: It is not much but we have some.

I: Is it only this or is there any other?

R: No, these are all.

I: Did they separately place or mix?

R: They are separately placed.

I: What is the reason for the expiry of these medicines?

R: In the previous time, the number of cases was very small in number. the 2010 compilation of medicines is case by case. Since 2012 the number of cases has been increasing.

I: What types of medicines are many in number?

R: Cloxacillin, antibiotics,

I: Is there family planning, children?

R: ORS, this is ORS.

I: Is there any other medicine?

R: Family planning I do not have.

I: a children's Zinc amoxicillin, is here?

R: No, we do not have children's medicines that expire.

I: What are the principles that you follow when you withdraw medicines from the store? For instance, there is a law that we need to follow when we withdraw medicines.

R: For the dispensary, we do not have formats such as RRF, and VRF we have finished the format.

I: What are the formats? RRF, VRF, and IFRR. How do professionals take medicine from you? Family planning,

R: They wrote a letter and presented it to the head and he approved that and then I gave it to them.

I: They wrote in a paper the head approved that and then what?

R: I let them sign model 22, and then I gave them the requested medicine.

I: How often did they withdraw the medicine? Did they take the time they wanted?

R: They are taking every fifteenth day of the month.

I: What about family planning?

R: Four times a month. Every eighth day of the month.

I: Do not they have a permanent schedule? What do you think is the reason, if they have a schedule do, they use it properly? If you schedule, it every week is she able to come and take it?

R: Yes, they follow the schedule.

I: What did they bring, when they came for withdrawal?

R: The list of supplies they finished.

I: They simply say we have finished, but they did not do the beginning balance and did not count stock at hand. When the medicines they need are finished they directly come and take them. Therefore, the weakness of the internal system is because of a lack of formats, and because of the lack of preparation schedule.

R: Yes, you can.

I: How do you see the punctuality of the professionals in requesting by fulfilling the required formats? For example, if Monday is the day, the professionals come and take every Monday. Is there any gap? The gap means, when they request, their request correctness; the exact

quantification of the medicine not too many and not too very small, commitment, lack of interest to their career and only they come to the schedule, how do you see these things?

R: There is a lack of shelf in the health post which is why I withdraw small amounts and they come and take every week. This is to protect against the wasting of medicines.

I: Did not they report how much they use?

R: They are not reporting to me. They give the report to the head of the health center.

I: Do you know how much they did use? What you know is the request or the amount that they came to withdraw but you do not know their consumption. If they need ten, they come to take ten.

I: Did not they inform you of the balance between the medicines used and the rest in their stock?

R: They withdrew the medicines which they finished.

I: The internal system means the relationship between the pharmacist and the health units (dispensary). Except this, the health center have additional tasks that cooperation between the health center and the health posts or the health extension officers so, how do you see this in relation to the supply? The health extensions request by correctly filling HPMRR, monthly reporting, and taking the supplies, how do you see these issues?

R: They have a good relationship with the interrelationship focal officer. They are doing things based on their discussion. They are asking about the availability and the shortage as they are going every week. There is also the command post.

I: Are they taking every week?

R: No, what I mean is that there is a command post team that provides support every week to the health post.

I: Who are they?

R: They are a command post.

I: They called the health post-supportive team. Is the health post-supportive team going every week?

R: Yes, they are going.

I: Yes, they are going; every weekend they evaluate, the command post is the name evaluation team.

I: Are they evaluating every Friday?

R: Yes, they are.

I: Do they evaluate the resources supply?

R: Yes, they evaluate.

I: What are the major challenges that hinder the presence of medicine in the health post? Can you tell me one, by one? Reasons that hinder the occurrence of medicines in the health post?

R: If the medicine is available here the health post also has the medicines. If we do not have medicine health also faces a challenge. There are many family planning users, which is why they frequently withdraw the medicines.

I: What is the problem with the absence of medicine which is available here but they are not avail there?

R: As I have informed you earlier transportation problem is one of the challenges.

I: How about the request are they reporting their consumption, or simply requesting what they want to take? Are they requesting correctly by filling in the exact amount they need? How often do they request?

R: They did not request the medicine which is not available in the health center.

I: How do they know that?

R: Before they wrote the letter, they came and asked me about the available medicines.

I: They check by phone, is there a form or are they use paper?

R: They also use plain paper.

I: What kind of paper and what it contains?

R: White plain paper (Dets paper).

I: They list the medicine that they need with the quantity they want, but they do not report what they have consumed.

R: They did not report.

I: To whom the paper is presented?

R: The paper is presented to the head.

I: Then he sends it to you and you will give it to them.

R: Yes, they bring the model and the approved letter.

I: What should be done in order to improve reporting consumption, and filling forms?

R: If they get the form, they can use it.

I: Are they requesting supplies before they finished or after they finished?

R: They requested before they finished.

I: How much did it leave, is it a one-day, or two-day?

R: If they plan to come on Monday they requesting on Thursday. They stay the whole Friday on their duty and come on Monday and withdraw the requested medicine.

I: Is it because of their program that they are requesting additional resources they have in stock? For instance, if a vaccine is needed for tomorrow, by checking the right amount in the stock.

R: If it is Plumpy nut, I asked the amount in their stock and I gave based on the data. If she has five children, I gave counting the adequate amount. When it was a vaccine, I gave a one-day vaccine, as they did not have a refrigerator. They are taken when the medicine is three days amount.

I: Is there a written rule/guideline that governs the date to request the medicines?

R: No

I: Have you heard about PMT, is it available here, do they meet?

R: Yes

I: Is there anyone who participates in representing the pharmacy in that team? Are you attending the PMT MEETING?

R: Yes

I: When do they meet? Is it once a month or a week?

R: Monthly

I: So, you are the member What do you do in the meeting, and what do you take to the meeting, as major health activities are evaluated in that meeting, and you are not going there without having something, therefore what do you take to the meeting? Do you take a report?

R: Yes, I took a report. In the report I talk about the program's medicines delivery schedule, and what is needed to bring adequate dispensary. I discussed how to improve my career.

I: If the pharmacy team is not properly participating in the PMT and if this is the gap, to avoid such things what should be done? When you meet the report of under-five, family planning is present, and you try to evaluate the strong and weak parts, what needs to be improved in under-five, family planning you wind up the meeting by preparing an action plan. The supply case did not stop only here it goes to the health post and others. In general, to make the supply chain management system fruitful what should the concerned bodies such as the region, the woreda, the health center, and the health post do to improve the challenges that you faced, and what should donors do in this way?

R: Our population is huge in number considering this the woreda needs to allocate an adequate budget for medicine purchasing. You the NGOs, as I have informed you earlier there is a

transportation problem because of the road so if it is possible support us by informing the region to repair the road. There is a great shortage of necessary medicines for family planning and others so these medicines need to be delivered on time and adequate number.

KII (15]

code	KII -A -10	Remark
**Place/region**	**[region]**	
**Organization Name**	**[health center]**	
**Responsibility**	**Health center head**	
**Education**	**First degree (health officer)**	
**Interviewee**	**KII**	
**Total Time**	**49:31**	**Starting time**

I: You are the head of the health center, what is the main challenge on supply chain management in general, medicines always should be available in the health center, in health posts, what did you

see about the supply chain management from the woreda, [RESUPPLYING HUBS], health center, and the health post? Focus on problems.

R: There is a great difference in the number of the supply we request and the delivery we get. For example, we request 1000 supplies but the delivery is 200. Most of the time demand and supply were not balanced, particularly MCH supplies we did not get based on our request. This is one of the main problems. The other is that…

I: What is the reason that you are not getting based on your request?

R: We do not know the reason. We are requesting in two ways. One we send our request online (with phone), and we also send the hard copy. Though we requested in two ways, we did not get as per our request. They sent a small number of supplies by saying there was also a shortage of resources. In the previous time **(2016)** it was better though there was an interruption, after they turned the delivery to the woreda the supply that we are receiving is very small.

I: Did you get the program medicines directly from the woreda?

R: Yes, we get from the woreda.

I: What did you fill in the form?

R: Monthly we fill and send the RRF form. When the supply came, they informed us to come with model 19th to withdraw the supplies.

I: How often did you request?

R: We request monthly, if the medicines in necessary we might request them as an emergency medicine.

I: One of the challenges is you did not get what you requested; you mentioned the reason. How is the quality of the report produced from the health facility as the quality of the request/report determines the resupply, how do you see the capacity and data quality to fill especially RRF?

R: The storeman is the one who is filling RRF. There are some problems, in filling the supplies that are completed, and in the ability to balance the completed and the supply at hand. When we asked her about the errors, she said I am not a pharmacist it is not my profession and I do not have the concept to fill out the form. It is not only the problem of them, there is a problem here with us. The form they sent shows the beginning balance and if she did not reply that she has received the beginning balance and she requested again they did not send it as she did not respond to the request. We discussed with the Woreda health office storeman this case as a gap.

I: The capacity and professional are not the right people so there might not be motivation. So, we can consider these issues. Is there another issue, these are the cases between the woreda and the health center, we have discussed about lack of adequate medicines in the woreda, if the medicine is available here is it possible to transport them on time to the health center?

R: There is a great transportation problem. It is possible to say no transportation access. We requested them last Thursday but since then we have not gotten it. It is difficult to get as soon as we request. In the previous time, when [RESUPPLYING HUBS] was delivering, we were waiting for the day they appointed with our model. On the due date, we come close and receive on the specified day. Now, when we are assigned to the woreda there is a transportation problem. Today, I asked Plumpy nut for children but they said no vehicle because of that I did not bring it. I left without it. We have such kinds of problems. Though there was an interruption, we did not miss all.

I: What is the problem related to health extension professionals concerning delivering the supply on time, how do they request and get supplies?

R: The system they followed was free and relaxed. Currently, we bought and prepare models 19th and 22. They fill the request and they need to submit the model 22 and when they request, they need to show the model 22 that they use to submit the supply at their stock. The chain which is between the health extension and us has transparency.

I: Do they have models 19th and 22?

R: Yes, there is.

I: Who receive and who withdraws?

R: If one of them has intake the other withdrew. After that, she will bring the model to us to show her intake.

I: She was taken here and withdrawn by another officer and put to usage, does she need to bring the model which she had taken the supply?

R: Yes

I: How often do they request?

R: Every week.

I: Did they have a schedule?

R: Yes, on Thursday.

I: How much stock did they need to have to request, is there a standard about this?

R: The standard says when they have 25%, they can request.

I: Is it monthly?

R: Yes, it is monthly, but here they might come every week and take. Currently, there is a shortage of supplies and they do not have much. There is a shortage because of that there is a problem, so, they can come and take when they have 25%, currently we can say that health extension professional did not have adequate supply.

I: Are they requesting when they completed the supply?

R: They requested even though they have some at their hand as they did not have an adequate amount. When [RESUPPLYING HUBS] was delivering we had many supplies and we were sharing them many supplies, but now we have a very small amount of supply so, we are delivering in pieces. We cannot say we are delivering. For instance, today I have brought four RDTs, how can I divide them as there are six health posts? In the previous time we were providing one or two RDT and **Coartem they were sharing.** Currently, I have brought 60 strips, and I am sharing a strip for them. Thus, there is a supply shortage. They are not getting the amount they request.

I: How is the human power in the health center?

R: In the health center there is a shortage of human power. There is no pharmacist totally and there is also other professionals' shortage. There is a gap in human power.

I: What is the reason?

R: We repeatedly requested to fill the position which has a budget, but I do not know why they neglect the issue.

I: Where did they go professionals who were working here?

R: Half of them were on study leave last year and this year. The other is that there are professionals who shift their working place (rotation) still that gap does not fill though they leave the budget. Some professionals leave because of medical problems (mental cases). In total, nine professionals are left from this health center. Others went to the [industrial park **]**, but these all-vacant positions were not filled. We have a great number of patients flow it is nearly about **52,890** catchments.

I: On average how many clients did you serve?

R: About 50 and beyond that.

I: So, there is client flow?

R: Yes, there is.

I: You are delegating active professionals to the positions that the professional left. Did the professionals who lift for study leave hand over the necessary documents, share their experiences on how to perform the intake and withdrawal process, and provide orientation about the overall of the position, before they leave to the newly assigned professional?

R: We did not simply assign someone without giving orientation. In the orientation, we tried to inform them how to do the activities, and how they perform the intake and withdrawal. So, we are replacing it by performing orientation.

I: Is the woman in the pharmacy had the orientation, and working in that place?

R: Yes, by the way, she was oriented by the pharmacist, before he left for study leave, he delegated, and she was also working as a dispensary professional at the previous time though she is a clinical professional. She has experience. She creates a complaint and wants to leave, but no other problem.

I: There is a budget for the professionals who leave their position, but not the practice of assigning other professionals, what is the challenge?

R: We were trying our best not to let the professionals leave their workplace, but to respect the rights of the professionals we permitted them to leave. We lost a professional who has the capacity to cover two or three positions; there was one professional from MCH not to let her leave was very difficult so we permitted her to leave her work. The woreda has to focus on the human power. I have discussed with the human resources officer about the number of professionals this health center requires five midwives and two or three pharmacists, why do not you employees considering this or why do not you bring professionals by rotation/shift? I have reported these issues to the concerned bodies. Still, now they did not respond. I asked the human resource why you did not request on time. For instance, yesterday, around the 11th hour when were to leave from the health center and most of the professionals were left, three mothers came to give birth while we were waiting for the duty professionals, so I took care of one of the mothers to give birth and left. For the other one, we look at her situation after a while we refer her. In a day there will be three or four mothers coming here for giving birth. There should be a professional who copes with the challenge and the risk. If the professionals are not ready to take responsibility it is challenging, for instance, a clinical is assigned to the position of a pharmacist, and in the middle of that if something happens, they might accuse us so and they refuse to accept the risk as the professionals are creating a compliant. Therefore, the woreda should give attention to this issue.

I: How do you quantify and forecast RMNCH supplies, how is the purchasing process of RDF? What are the bases for forecasting? What are the challenges related to this?

R: Most of the time we forecast discussing with the DTC committee. Most of the time when the medicine is 25% it shows a sign. they discuss the issue and we discuss to purchase the supply and then we request the woreda. This is the way we are performing the purchasing.

I: In order to forecast the next year, are you forecasting once a year?

R: We forecast once a year, but we have got the budget every quarter. Now around this, we are working only with health insurance (CBHI).

I: What are the sources of money for purchasing, is it by the health finance care budget which is an internal income, or by the budget that you get from the woreda?

R: The woreda release is small, the purchasing is performed by the internal income. We report the consumed, and based on that they released, and after that we purchase. We are purchasing when we have a 25% supply.

I: Let's see this in two ways, there is annual quantification in which the DTC is involved, do you quantify annually? For instance, are you requesting Cipro medicine this much, Paracetamol medicine this much, or when you want to purchase you report to the woreda and then you purchase? Does [RESUPPLYING HUBS] come to you for the quantification, or are you a member of some selected health centers that [RESUPPLYING HUBS] uses for quantification? Did not they come to you?

R: In order to forecast the next year's supply first we count the supplies in the budget year. After we counted, we tried to identify medicines that are frequently taken by the clients and medicines that are less to be taken. Considering this we request plenty amount for the one that is needed by the clients and we hold the other item in a small amount.

I: Do you have a proposal that you quantify the next year's supply?

R: Yes, we have.

I: How do you manage the quarterly plan, is it from the yearly plan that you take or what?

R: We take from the yearly plan, how much Ciproxin, Amoxicillin, Doxycycline, and Ceftriaxone are needed in the quarter. These are also the medicines that most of the time clients need. There are ten top diseases and five top diseases considering this we plan in two places for adults and those under five. We perform based on this.

I: What kind of data does DTC use for quantification? What is the base for quantification?

R: The case flow and number of patients based on this they quantify.

I: Is there any other reason for quantification except the case flow and number of patients? Is there any indicator for quantification?

R: No.

I: What about consumption? For instance, last time I used 100, and the next I am going to use this much, or there might be such a number of cases that I need this much supply, are they quantifying in these ways, or what?

R: Even though we quantify in this case [RESUPPLYING HUBS] did not provide us the amount we requested for the purchase. We were requesting to purchase 200 but we purchased 100.

I: So, [Health Centre] is not an [RESUPPLYING HUBS] quantification site, so you did not send based on that.

R: We sent it to [RESUPPLYING HUBS].

I: This year, did [RESUPPLYING HUBS] provide and coach you on how to fill out the form?

R: No, last time they were requested by phone. They told us that they are adjusting the system. They are digitalizing the system.

I: There is annual forecasting that [RESUPPLYING HUBS] rounds to a selected health center and supports them to do quantifications, so did you do such kind of thing?

R: No, they asked me in a phone-like data collection.

I: How do you perform the purchasing?

R: The woreda informed us to purchase **686,388**-birr medicines.

I: Who permits this?

R: The Woreda administration. We purchased beyond 400 thousand-birr medicines. The subsidy from the region was 105 thousand and 89 thousand. Generally, this year we have bought around 90% of our yearly plan.

I: Why did not you buy the supply by the rest birr as you know there is a shortage of medicines, but you returned a certain amount of birr, are you going to use for the future?

R: We do not have an extra budget, but we properly managed our budget. To avail the medicines that are vital for the community. We were protecting against unwanted waste.

I: Is there a budget shortage?

R: Yes, there is.

I: For purchasing medicines?

R: For medicine purchases no shortage of budget.

I: If there is no shortage of budget for purchasing medicine, there would be adequate medicines in the health center. But if there is a shortage of medicines availability what is the reason?

R: In this cluster, this year we did not have a problem with a shortage of medicines. They permit based on our quarterly plan, and then we purchase. They were asking us if we had bought medicines beyond our consumption. There might be a shortage in other clusters but here there was no medicine shortage problem.

I: Is 686 thousand birrs, is it for a quarter or annual?

R: It is an annual budget.

I: Do not you face a medicine shortage because of the budget shortage in this health center?

R: There might be, for instance, we are not getting the major kinds of items such as Ceftriaxone when we want to purchase Ceftriaxone the cost is expensive, so by dropping that we purchase others by balancing our budget.

I: You drop the medicines because of the budget problem. It costs many birrs as it is expensive. So, in general, there is a budget shortage.

R: Yes, there is. It is not adequate for instance if you divide 683 thousand by 52, 890 it is very small. We are purchasing paper from that budget, fee for the operational process is also subtracted from that budget, we can go to the fourth round and we tried to purchase soap and other things by taking a small amount.

I: You were talking of DTC and PMT, is there PMT, or is it functional?

R: Yes, there is. Concerning functionality, it becomes a weakness.

I: Why?

R: It is the second month that all the professionals are working in the locals (kebele). The professionals are working from Monday to Saturday in the kebele. It is because of this that PMT is weak. They are going to kebele and unable to come here. We did not have time to meet.

I: So, you do not regularly meet and evaluate.

R: Till we started the Champaign we were properly doing our activity but now there is a gap.

I: Who are the members of the PMT committee?

R: The PMT is formulated by different teams.

I: Are there pharmacy professionals or a person who represents pharmacy in the PMT?

R: Yes.

I: For instance, there might be a member from a pharmacy, under five, MCH, OPID, interrelationship focal, when the pharmacy professional comes to the meeting what kind of data did, he bring?

R: The data which is necessary to them.

I: What kind of data? Do you remember any data, a problem that you saw, and a solution that you forwarded concerning the pharmacy?

R: There are lots of things that need to be filled, need to be planned, and need to be accomplished. The pharmacy department is the major department of the institution. Unless the client gets the medicines, it does have a meaning for the client having various examinations. It insults the organization. To provide quality service to the client pharmacy department is the main department. To divide into two essential and non-essential and to overall activities pharmacy department is the core department in the health center. The professionals who are working in that position need to know many things for that we send them to get experience and training.

I: What is the role of the pharmacist who is delegated in the PMT?

R: Protecting the drug shortage, quality. Checking the process of requesting and resupply the items on time.

I: How is the PMT, is it functional?

R: Since March it has been weak. I already mentioned the reason, as we are in a continuous Champaign. All the professionals are out of their working place and they are in the kebele.

I: The pharmacy in the PMT or the supply chain tried to review the indicator, for instance, our woreda requested this amount but it did not get the amount it requested, the Depo we requested was 40 but we got 20, The number of patients with correctly filled prescription this much, and their knowledge is at this level and it should be corrected, our wastage level is like this, do the pharmacist bring such kind of data in the PMT meeting, and based on that, does the management give a solution or what?

R: He came having what he has, for instance, what is needed in the pharmacy he shared his idea, and discussing there might be questions in the discussion and I tried to reflect on how I can react to those questions which I can request the woreda to react. There were supplies that we were requested by receiving supplies that have been gained; we again planned those supplies which we did not get now. In the previous this institution was in a very weak performance, but since we started discussing and evaluating the various issues brings an improvement. We are trying our best.

I: In general, we are talking about supply chain management, earlier you mentioned lots of problems related to this, what should be done to improve the supply chain management? Supply periodically and on time would be in the institution. What do you recommend, the woreda, the health center, and NGOs, need to do what?

R: This is a nice question, the first is that hiring a professional is the primary task. If there is a pharmacy professional, he/she can manage the supply in a proper manner to address the client. The other is that we are not getting the supply as per our request and I cannot able to identify where the problem is. Our monthly consumption of MCH is 1006. Our 2016 plan was 1006 including all, but we are unable to get per our request there is a resource shortage. It was 1006 but they may give us 600 or 700. By dividing the total amount for each item, we request items Depo 200, Implanon 100, IUCD, and Pills total is around 1006. If we get the supply as per our request, we can solve the problems. The cause for our problem is the inability to fill out RRF and VRF and if we fill and send them on time the problem will be solved. The other on the woreda should give us according to our request, we precisely solve our hindrances. [RESUPPLYING HUBS] did not give us based on our request, if they deliver based on our plan we can work in a better performance.

I: Thank you very much.

KII (16)

**Name of Project:**	[project]
**ID Code**	KII-A-08
**Type of interview**	KII
**Type of informant**	[sex]

**Region**	[region]
**Role of the Interviewee**	Logistics officer
**Years of experience**	6 years and more
**Name of Transcriber:**	University lecturer
**Date of Transcription**	August 2024
**Names of Interviewers:**	University lecturer
**Length of time**	1:21:58

I: OK, thank you. Now, proceeding to the first question on the supply or supply chain management program or system: Would you please tell me your role and responsibilities in the supply chain management system?

R: OK, my responsibility in district hm….hm……hm… at the Health Office, I am a logistics officer and delegated store man. I am responsible for monitoring, following up, and supporting activities conducted in the lower system for any questioning. So, my work responsibilities include being an officer and a store man.

I: Thank you, that sounds very good. As supply and logistics officer, and store man and leader of the system, since you have worked as an officer and manager, how do you view the supply chain management system from the health post to the woreda? In this process, how would you explain the existing supply chain system effectiveness and the overall process?

R: The first thing that existed… eee… eee… it was requested directly from [resupplying hub] and provided to the woreda. But now, it has been three years it stopped since a project called "Last Mile Delivery" started, and [RESUPPLYING HUBS] itself began direct delivery. Now, all health centers receive supplies from [RESUPPLYING HUBS]. They request from [RESUPPLYING HUBS] and receive the supplies, but there was a compiling trend prior to three years where each health station filled out RRF requesting form. After the forms were compiled here, they came to the woreda only. However, after three years, it started direct delivery. So now, we do have a follow-up and supporting role.

I: So, regarding addressing the need on time and in the requested amount, what is the situation or the reality that exists here?

R: It is very challenging. Currently, [[RESUPPLYING HUBS]] does not provide supplies in the requested amounts; sometimes, it's very small, even below the necessary amount. There are an also stock out that has been accessed, in some cases it is none at all. So, the interaction between [RESUPPLYING HUBS] related to supplies is quite challenging… eee… On the other hand, the requesting system itself, coupled with the shortage of supply and lack of formats has been difficult. We were used asking tools by print hard copies using a computer here. However, due to the challenges faced, all reports were not compiled and sent. This was a significant challenge at the health centers.

I: health centers?

R: yes health centers.

I: What was the problem in reporting? Sometimes expert's readiness may create gap in input supply. Especially in relation to sending request on time….and sending report on time filling in the right way could be a challenge. So, what is a challenge existed there related to health centers preparation and readiness related to preparing reports and data qualities. What problem you observe?

R: This situation is very challenging.

I: what are these challenges?

R: The challenges include the unavailability or a lack of health workers. This is particularly challenging in our woreda, where the main issue is the shortage of health workers particularly pharmacy professionals. We don't have enough pharmacy professionals to compile and preparing reports. In some areas, we provide on-the-job training and guide other clinical health care providers on how to work through practical demonstrations. This is one way we support them, but it remains a significant challenge.

In addition, the formats used for reports, like the IFRR and those specific to health centers, have their own forms utilized in their dispensing units.

I: Do you mean internally?

R: Yes, exactly. The inability to use these forms is a challenge. There is also a lack of paper for printing in each category, along with budget issues, which are significant challenges. Furthermore, health post deliveries are not available. There is a sense of negligence, as some staff claim they

have not received training in health extension work. These factors, among others, contribute to the challenges we face. We have identified such reasons.

I: What major challenges have you identified? For example, could it be related to transport or storage? There are always interruptions, so what could be the reasons behind this?

R: Yes, having an uninterrupted supply is crucial. The major challenges we face include transportation issues, which can delay the delivery of essential supplies. Additionally, storage limitations at health stations can lead to shortages. There are also budget constraints that affect our ability to maintain a steady supply of inputs. Moreover, the problems are related to the requesting system and supplies shortages, such as paper and formatting issues. Sometimes, we even send requests using printing paper. This adds to the challenges we face in maintaining an efficient work flow.

I: What are the challenges in the health sector related to reporting and requesting inputs on time?

R: The challenges are primarily related to reporting formats, such as the IFRR, which are used by health centers in each dispatch unit or working units. Additionally, we face budget shortages that further complicate our ability to maintain efficient operations. These issues hinder our capacity to report and request supplies in a timely manner, ultimately affecting the quality of care we can provide.

I: It is essential to have a continuous and uninterrupted input supply system in health centers and health posts. What are the reasons for the interruptions supply? Could you list the major challenges? For example, it could be related to transport, storage, or stock outs. It might also include issues related to expertise. What are the major challenges to accessing inputs continuously and on time in health centers or health posts?

R: In addition to the challenges, I mentioned earlier, supply integration is another major problem we face. While the intention is to have a consistent supply of commodities in health centers, interruptions still occur. One significant reason for these interruptions is transportation problems. If the logistics are not well-coordinated, it can lead to delays in receiving necessary supplies.

Moreover, there are issues with the requesting system, which can complicate the process of obtaining supplies on time. When the integration of supply chains is not seamless, it creates gaps that can result in stock outs or delays.

Overall, these factors contribute to the ongoing challenges in ensuring a continuous and timely supply of inputs in health centers, ultimately affecting our ability to provide quality healthcare services.

I: How could it be a challenge? What do you mean by it?

R: The challenge of timely input supply is multifaceted. For instance, even when supplies are available at the woreda level, health centers may not be willing to collect them on time due to transportation issues. Many health centers require a motor vehicle to reach distant locations, and often, health posts and health centers share a single motor. This shared resource can lead to delays in distribution, as it may not be available when needed.

Additionally, there is a lack of knowledge among staff regarding how to effectively request and report their needs. Experts often struggle with the process of requesting what they need and accurately reporting what they have received. This knowledge gap can lead to inefficiencies and further delays. Moreover, the limited number of human resources is another significant challenge. In many cases, staff may refuse to complete tasks on time, citing reasons such as a lack of knowledge or other barriers. To address these issues, we have identified the need for targeted training programs to equip staff with the necessary skills and knowledge to manage requests and reporting effectively. This training is crucial for improving the overall efficiency of the supply chain in health centers and ensuring that inputs are distributed on time.

I: so, now how health posts access supplies from health centers?

R: health posts?

I: Yes, health posts.

R: health posts take supplies from health centers by asking/ requesting.

I: How do health posts request inputs from health centers?

R: Health posts typically request inputs from health centers using a couple of methods. In areas where it is available, some requests are made using the RRF format. However, in other locations, staff may resort to using any available paper to write a request letter. This process allows them to obtain the necessary items they need.

Initially, the IFRR pad was utilized across all kebeles for requesting supplies, but unfortunately, it is no longer in use or accessible in our woreda. This lack of availability has made it challenging for health posts to effectively communicate their needs.

They depend on informal methods, like handwritten requests, can lead to inconsistencies and delays in the supply chain. Therefore, improving the accessibility and functionality of standardized request systems would greatly enhance the efficiency of how health posts access supplies from health centers.

I: How do health extension workers transport supplies to health posts? What challenges do they face in this regard?

R: Health extension workers transport supplies to health posts by first requesting the necessary items and obtaining approval from the health center manager. Once they have the approval, they use available transportation, such as a motor vehicle, to carry the supplies to the health posts.

However, there are several challenges they encounter in this process. One significant issue is the availability of transportation. Since health posts and health centers often share a single motor vehicle, this can lead to delays in picking up and delivering supplies, especially if the vehicle is not available when needed.

Additionally, upon arrival at the health post, the extension workers must go through an approval process before they can provide the items to clients. This step can further slowdown the distribution of supplies, particularly if there are any discrepancies or issues that need to be resolved. Overall, these logistical challenges can hinder the timely delivery of essential commodities to health posts, impacting the quality of care provided to the community. Addressing these transportation and approval bottlenecks is crucial for improving the efficiency of the supply chain in our healthcare system

I: Who covers the transport cost for health extension workers when they use private motor cycle for transporting commodities to health posts? Do they pay out of their own pocket, or is there a system in place to cover these costs?

R: The transport costs for health extension workers are generally covered by the health centers. Each health center has designated leaders and coordination focal persons that assist in organizing travel to health posts. Additionally, there are motor vehicles available at all health centers that are used for transporting supplies.

This system helps ensure that health extension workers do not have to bear the transport costs personally. Instead, the health centers facilitate the transportation of inputs, which allows for a more efficient distribution process to health posts. By utilizing the available motors, health extension workers can effectively deliver the necessary supplies without incurring additional

expenses. However, in some cases the health extension worker pay fee for the private motor cycle owners for their transport

Interviewer: so, there is no problem in this regard?

R: While there is no a system in place for transporting inputs, there are significant challenges. One of the main issues is the ratio of health centers to health posts; there is typically one health center for every six health posts. This means that each health center only has one motor vehicle to serve multiple kebeles, which makes it challenging to deliver supplies on time. At times, health extension workers or experts may have to take it upon themselves to cover all the costs of transportation. This not only places a financial burden on them but can also lead to delays in delivering essential supplies to health posts. Therefore, while the system has its advantages, the logistical constraints and resource limitations present considerable challenges in ensuring timely access to commodities.

I: Do you mean that health extension workers cover the transport costs from their own pockets?

R: Yes, that's correct. Health extension workers often have to cover the costs for transportation themselves, particularly when using private motor vehicles. This situation can be very challenging for them, as it adds an additional financial burden.

I: So, are these the primary means of dispatching inputs from health centers to health posts?

R: Yes, that is indeed the primary way commodities are dispatched from health centers to health posts. Despite the challenges, this method is currently the most utilized for ensuring that health posts receive the necessary supplies

Interviewer: ok, very good. For health centers, you mentioned that supplies are directly delivered from [RESUPPLYING HUBS]. How is the provision of supplies for health centers looks like? Are there any challenges? How do [RESUPPLYING HUBS] and health centers interact?

R: in this regard the existing system is very difficult.

I: what makes it very difficult?

R: as I mentioned earlier, prior to three years ago, there was a delivery system directly from IPSA to woreda health office store. There were also direct connections between health centers and [RESUPPLYING HUBS] using mobile application systems provided by the organization. During that time, tasks such as requesting, receiving, and reporting were all managed through the application, which facilitated [RESUPPLYING HUBS].

However, this system had its problems because it often bypassed us, even though we are responsible for overseeing these processes. As a result, we are arranged a new system and started receiving hard copies of requests and reports instead. Many experts have expressed their dissatisfaction with this change, arguing that the mobile application was sufficient for managing these tasks. This shift has created additional challenges in communication and efficiency, making the distribution of supplies more complicated than it needs to be.

I: was it bypassed you?

R: Yes bypassed us not only this but also there was interrupted since we were not following up it.

I: What problems have you identified between [RESUPPLYING HUBS] and health centers in accessing supplies like drugs on time?

R: There is generally on-time access and distribution, especially after the direct delivery system started. However, all requested amounts and types still face challenges in access. For example, with anti-TB medications, there have been significant interruptions. Since April, there have been no supplies are available without interruptions, and currently, the supply has completely stopped. This situation highlights the ongoing issues in the supply chain, where even with a system in place for timely deliveries; specific medications are still not accessible when needed. These interruptions can severely impact patient care and treatment outcomes, making it crucial to address the underlying problems in the distribution system to ensure that health centers can reliably access the necessary drugs.

I: totally stopped?

R: yes, totally stopped.

I: what is the reason for this?

R: The interruption is primarily due to accessibility shortages and the implementation of new working systems. [RESUPPLYING HUBS] has informed us that they are in the process of updating their system, which has resulted problems to access supplies at all. They mentioned that supplies have been allocated to the regional level, but the amounts are far too small to meet our needs.

Currently, there is a high level of grievance among health centers as we find ourselves in very challenging conditions. Previously, when health centers were unable to request or access supplies, we could still make requests. However, now, we are unable to ask for anything because the systems are directly connected with health centers, and the ability to place requests has been closed off.

I: What about the staff commitment? I think there is a minimum standard of amount (stock) for requesting. It is not good waiting until the stock is zero. So how is the staff commitment in requesting supplies before it becomes zero, filling the required data, and using the data for decisions?

R: this is really a challenge we faced.

I: what challenges you faced? Would you tell me?

R: Unfortunately, staff commitment to this process has been lacking. Many experts do not see it as their responsibility, which has led to negligence despite our efforts to encourage their commitment through various means, including direct engagement. They often express that they are not pharmacists and therefore do not feel accountable for the tasks. This mindset has become a source of complaint among the staff.

The delegation of responsibilities created discomfort among non-pharmacy professionals. Since these workers are not trained as pharmacists, they feel justified in their reluctance to take ownership of the task of managing stock levels and making requests. This has resulted in a situation where they wait until stock levels are critically low, which is not ideal for patient care.

Moreover, the current system's limitations have exacerbated this issue. With the inability to make requests effectively, even those who are committed find it challenging to fulfill their roles. This lack of accountability and commitment to the task at hand is a significant barrier to ensuring that health centers have the necessary supplies to operate effectively.

I: do you delegate clinical workers?

R: Yes, we do delegate tasks to clinical workers, as there are no pharmacists available in our woreda. In fact, we have three to four health centers that do not have even one pharmacist on staff. This situation necessitates that clinical workers take on responsibilities typically handled by pharmacists. While these clinicians are capable of managing certain tasks, the lack of formal pharmacy training can lead to challenges in their commitment to these responsibilities. They often feel that they are not equipped to handle tasks related to supply management and supply requests, which can result in delays and inefficiencies in the system.

This delegation is essential for maintaining operations, but it also highlights the need for better training and support for these clinical workers. By equipping them with the necessary knowledge and skills, we can enhance their confidence and commitment to fulfilling these critical roles, ultimately improving patient care in our health centers.

I: do you have a health center with no pharmacist?

R: yes we have.

I: how many health centers?

R: four health centers

I: so, who worked the task of pharmacist? Who is delegated?

R: Yes, we delegate tasks to clinicians and nurses, especially since we lack pharmacists in our woreda. This delegation is necessary, but it also creates communication problems. We have made efforts to trace issues within the system, identifying where supplies are overstocked, under stocked and other gaps that exist.

The challenges we face are significantly related to lack of commitment and communication among the staff. When clinicians and nurses do not feel fully responsible for the tasks assigned to them, it leads to misunderstandings and inefficiencies. This lack of commitment can stem from their perception that these responsibilities are outside their expertise, which further complicates the situation.

Effective communication is crucial in healthcare settings, as it fosters collaboration and helps prevent errors. However, when there is communication gap, it can lead to delays in addressing stock issues. This is a significant challenge that we need to address to improve our operations and ensure that we meet the needs of our patients effectively.

I: How is the supporting system from the woreda for health centers and health posts? For example, in managing, budget allocation, format and pad distributions for reporting and requesting purposes, etc.

Interviewee: The woreda does provide support for health centers and health posts, but there are significant challenges, particularly regarding budget shortages. In our woreda, this issue is more noticeable. For instance, this year (2016 EC), the woreda was only able to allocate budgets for three health centers out of the total requested budget. This limited budget severely impacts our ability to operate effectively and meet the needs of the community.

Additionally, while there are established formats and procedures for reporting and requesting supplies, the distribution of these formats can sometimes be inconsistent. This inconsistency can lead to confusion among staff, making it difficult for them to complete reports accurately and on time.

Addressing the budget constraints and improving the clarity and consistency of reporting formats are essential steps toward enhancing the operational efficiency of our health facilities.

I: from how many total health centers it means?

R: from nine health stations.

I: for how many it allocated and distributed?

R: in our woreda, although the budget was allocated for nine health stations, only three received the necessary support. The remaining health stations were left without any resources, which presents a significant challenge for us. Transportation is another major issue; it requires many resources, and without adequate logistics, any program can collapse. Resources are essential for various inputs, such as paper and transportation, but unfortunately, nothing is being done in our woreda to address these needs.

Previously, resources like IRR, IRF, and HPMRR were provided with pads, on-the-job training, and on-the-spot support, but now all of that has stopped. This absence of support has highlighted the weaknesses in our system, particularly as there is no special assistance coming from the regional level.

I: How the transportation system is?

R: it is clear that transportation issues are at the forefront. This has led to significant food shortages for children at the Integrated Community Care (ICC) facilities. The transportation problem is indeed a serious concern that needs urgent attention to ensure that our health programs can operate effectively and meet the needs of the community.

I: you told me that you have conducted supervision, in what ranges you have conducted supervision and support?

I: Our supervision has been integrated with various programs due to the ongoing lack of transportation. We have conducted supervision across different health facilities at various times throughout the month and over a three-month period.

There are instances when we focus solely on the work processes, but we also utilize checklists to identify specific tasks that need attention. This integrated approach allows us to cover multiple aspects of health service delivery during our supervision visits.

By combining different programs, we can maximize our impact and ensure that we are addressing the most pressing needs of the health facilities. However, the transportation and budget challenges often limit our ability to conduct more frequent and comprehensive supervision.

I: How many times you go filed for supervision by the work process only per year?

R: two times a year.

I: what about in integrated program?

R: integrated it is done in a month.

I: In case there is a turnover of staff and new staffs are employed, do you have systems like inductions and experience skill transfer?

R: No, we do not have such systems.

I: so, what is done when new employ is joined as of your woreda?

R: as woreda, when a new employee joins our woreda, the roles and responsibilities of their position are primarily communicated by the work process owner. This provision aims to clarify what the new staff member is expected to do, along with the relevant statuses and directions. Unfortunately, this is the extent of our formal induction process; we do not have a comprehensive system of inductions or well-prepared training programs in place.

I: Do you have any rewarding systems for senior experts? This could be a mechanism to retain long-term workers and encourage them. What systems are available in this regard?

R: yes we encourage especially when we identify gaps. Overload and duty have been paid for experts. However currently, we do not have a structured rewarding system specifically for senior experts. While we recognize that having such a system could be beneficial for retaining experienced staff and motivating them, our resources are limited.

Implementing a structured rewarding system could help improve staff retention and morale, and it is something we hope to develop in the future as resources allow.

However, one of the problems in our woreda is unable to pay over load and duty for Pharmacists and laboratories.

I: Are they working like others? Why? If they have entered, why are they not paid?

R: Initially, payments were only made for weekends, and at that time, we only had laboratory experts. The budget is managed at the woreda level, but the details were not disclosed to us. When the woreda pharmacists began to voice their concerns and ask questions about this situation, the

efforts we made to address their inquiries were ultimately ineffective. This became another significant challenge we face.

I: Is this problem due to budget challenges, or are there other reasons involved?

R: The issue is primarily budget-related. Currently, the woreda lacks the necessary systems and guidelines to facilitate payments for pharmacists. While clinicians receive payment for their duties, including DOT (Directly Observed Treatment) and overload work, there is no allocated budget for pharmacists' overload payments. This absence of a structured approach and clear directions regarding compensation for pharmacists has created significant challenges within our organization.

I: do pharmacists work over load?

R: no. they don't work since they are not paid.

I: So, who dispenses drugs on weekends and holidays?

R: At night, necessary medications are provided to the active emergency workers who are responsible for dispensing. The balances of these medications are then checked during regular working hours on the following business day. This system ensures that emergency workers have access to the drugs they need while maintaining accountability for inventory management.

I: Could you explain the process of quantification and purchasing, particularly in RMNCH and RDF situations?

R: Drugs are procured through communication among the members of the Drug and Therapeutics Committee (DTC). This committee collaborates to make decisions regarding purchases. However, when it comes to quantification, there is currently no systematic approach at the woreda or regional level. Only one health center in our region is designated to handle quantification.

The drug purchasing process, they submit request with decisions from the DTC, including the budget and list of drug items. We then review their plans to determine if they are necessary. Unfortunately, even when purchasing is approved, the required items are often unavailable at the Ethiopian Pharmaceutical Supply Service ([RESUPPLYING HUBS]). Out of a hundred items, it is difficult to access even 15, despite encouraging health centers to procure from government organizations. Most of the time, they end up with stock-out drugs. While [RESUPPLYING HUBS] is supposed to serve government organizations, obtaining the needed drugs is often impossible. Moreover, there is a significant disparity between the budget planned and the actual purchasing costs. The allocated budget frequently cannot cover the intended drugs due to price inflation.

Therefore, we face two major challenges: first, the unavailability of required drugs at [RESUPPLYING HUBS], and second, the variation in budget or increased costs.

I: What additional challenges are encountered in the drug procurement process beyond the two issues previously mentioned?

R: I mention the budget. Sometimes, even if there is a budget, the health center and woreda managers collaborate but do not approve the allocated budget, which prevents them from making purchases. The health centers themselves must approve what they purchase in committee at the facility level, and then the DTC approves the budget for the health center. The health center board, which is responsible for the woreda health office, along with the administration committee members, must also approve the requested budget submitted through a letter. Each health center has its own board that is accountable to the woreda health office, and these board members are involved in the process.

Thus, the budget is approved by the board, which decides how many drugs should be purchased. The DTC only determines the items to be procured, while other quantifications are handled by the pharmacists. After passing through all these processes and obtaining the necessary approvals, pharmacists from the health center proceed with the [RESUPPLYING HUBS]. If [RESUPPLYING HUBS] says stock-out drugs or if certain items are unavailable, pharmacists distribute preforms, which are then submitted to the committee for consideration. The costs are compared, and preforms are also collected from private suppliers. Finally, the winning bid is selected, and the drugs are purchased and supplied.

I: how is the transportation process?

R: After the drugs are purchased, the health center contacts the woreda health office, which then sends a vehicle. There is a vehicle that has been donated by the minister health specifically for this purpose. In some cases, health center ambulances are also utilized to transport the supplies.

I: who is the owner of a car donated by health minister to support health transformation?

R: woreda health office

I: what if this car is not available? How it could be transported?

R: if it is not available, as our woreda, we use other cars like agriculture office, woreda administration office car covering the required costs like perdim and fuel.

I: so in the process of buying drugs all the problems you mention are faced, what has to be done to alleviate the quantification and challenges of drug supply? What solutions you suggest?

R: I believe that, first, it is essential to ensure that the quantified drugs are available at [RESUPPLYING HUB]. Second, health centers should receive adequate support for their budgets. If these two measures are implemented, I believe the challenges can be effectively addressed. Sometimes, when transportation issues arise, experts and health centers end up covering the costs themselves. Additionally, there are challenges related to road conditions, rain, and storage facilities. At the woreda level, it is crucial to have reliable transportation options that can protect the drugs from rain and sunlight. Therefore, it is important to resolve all these issues to facilitate a smoother procurement and distribution process.

I: as you mentioned earlier, most health centers are direct delivery from [RESUPPLYING HUB] in transport wise. The challenge is transporting from health centers to health posts. And as you said indifferent time different mechanisms used; sometimes motors used and in some cases experts cover costs and traveled it. However, who is responsible to access supplies from health center to health posts?

R: health center. It is the responsibility of the health centers. Something which I don't mention earlier, as our woreda, there is health centers faced challenges for the direct delivery. Especially Motto is not accessible for transportation if rain. There is a road problem. It doesn't think to enter and out car after raining. So what we done as a solution is health posts should take and received. It is difficult access direct deliver due to road. Even health extension workers received and take child foods by their own costs when it is scarce.

There are some health centers don't have motors. Some are stopped and don't give services. As our woreda, it is since five and six years that motors bought for health centers. Even the existed are very old with no significant maintenance. So I believed that it required and better to have better transportation access from health centers to health posts to solve input interruptions.

I: very good, what other solutions you suggest to have better transport access from health centers to health posts?

R: I propose that health center areas should ensure better road access in the regions and woredas. There have been instances where mothers have died during childbirth while trying to reach health centers using traditional ambulances (Kareza). Therefore, improving road access is crucial.

Additionally, there are situations where patients face the risk of drug shortages, even when medications are available at the woreda level, due to transportation issues. This often occurs during the rainy season. Thus, enhancing road infrastructure is essential to address these challenges effectively. This is what I believe should be done.

I: Does [RESUPPLYING HUBS] supply for all the nine health centers? Have no any problem?

R: Direct deliver?

I: yes direct deliver? Is there any reason not to supply for all the nine?

R: No. even in what I said earlier [RESUPPLYING HUBS] go until it is possible and then calls or message for the health center and make ready to receive and take it in the nearby areas.

I: How many health centers are difficult for transportation in raining time?

R: three health centers. Especially [3 health centers] are difficult in raining time. In the raining [RESUPPLYING HUB] is not entered there. Health center experts take on the road.

I: I am not clear that if the [RESUPPLYING HUB] car unable to enter, how they can receive and take it?

R: as a woreda there is Ambulance, so ambulances are used while the road becomes relatively dried. In case there is no Ambulance using our own woreda health office car is common.

I: you mean receiving from [RESUPPLYING HUB] car?

R: Prior to distribution, pharmacists receive the drugs and store them in health posts before later transporting them to health centers. For example, for Motto health centers, experts receive the drugs from [RESUPPLYING HUB] and store them in nearby health posts. These health posts have storage facilities and a key for access. When the weather is relatively dry, the drugs are then moved to the health center.

I: is the health post near than the health center?

R: yes

I: is there a possibility to stay for a night at health posts?

R: yes it stays until the road dried. They do have storage place and key. We have arranged it through communication and cooperation to solve the problem.

I: how is the inventory management in health centers? In how many time inventory is done? Are formats are recorded as expired or not? What is in inventory management system?

R: it is recorded. We have done Physical inventory two times specifying and focusing on our programs. In this time, we tried to identify the expired bills and update requirements. Clinical also do physical inventory two times a year. We make them cooperative and supportive in making inventory.

I: with regard to drug wastage prevention, do you have any finding of outdated drugs?

R: yes we found

I: is it placed in a separate place?

R: yes, it is placed separately. The problem is in avoiding it. It is simply accumulated without discarding.

I: Why not discarded?

R: It is a significant issue that the drugs are simply ordered by [RESUPPLYING HUBS] to be placed with a full record, leading to accumulation in every health sector without proper removal. This problem remains unresolved, even after we, as a woreda, raised our concerns. The disposal system prepared by [RESUPPLYING HUBS] has failed, and there are still no clear decisions on how to dispose of these accumulated drugs. This situation poses a serious challenge that needs immediate attention.

I: What do you think are the reasons behind the issue of expired drugs?

R: Some drugs are distributed through a push system without any prior need or request. For instance, Oral Rehydration Solutions (ORS) are sent to health facilities even when they may not be necessary at that time.

I: who bring it without need and request?

R: [RESUPPLYING HUBS]

I: while not asked?

R: yes while not asked.

I: How?

R: Sometimes, requests for drugs are made by individuals involved in the program, and they receive supplies from [RESUPPLYING HUBS] based on these requests. However, there are instances where drugs are requested in excess, leading to overstock. To address this issue in our woreda, we have developed forms to facilitate the redistribution of overstocked drugs from centers with excess supplies to those that are experiencing shortages.

I: what electronic systems do you have to capture data? Is it [ELMIS] system?

R: no, we don’t have

I: don’t you have medical recording system?

R: we don’t have

I: don’t you have [ELMIS] site?

R: no

I: all nine health centers are not used [ELMIS] site?

R: yes all are not used.

I: Based on the data analysis issues you mentioned earlier, as well as the transfer system for redistributing overstocked items to those in need, what observations have you made regarding the exchange, transfer, inventory, and waste management systems?

R: In the exchange system, transfers are primarily made for RDF drugs. For other items, we conduct stock analysis within the program and facilitate transfers to health centers where they are needed, using forms created by health center managers, medical posts, and health centers. This process ensures that inputs are redistributed to areas experiencing shortages, while also considering the suitability for auditing purposes. This approach is particularly important in program areas where audit gaps are frequently identified. The forms used for these transfers are prepared at the woreda level, ensuring a structured and accountable system for managing drug distribution.

I: Who conducts the stock status analysis— is it done by the woreda or by each health post or center?

R: the woreda

I: where you access each stock status

R: In our case, support is provided in coordination with MCH, and we conduct visits every month or two. During these visits, we identify the items available in the store, perform a physical inventory count, and compile the data. Other locations follow a similar process to ensure accurate tracking of stock levels

I: Do you have AMC?

R: eeeeeee………hmmm….. what have you said?

I: Do you have an Average Monthly Consumption (AMC) metric to assess your stock levels? For instance, if you have 100 units in stock but a monthly consumption of 200 units, it indicates that your stock is insufficient for a month’s needs. To make informed decisions, it's essential to conduct a stock analysis, which involves dividing the available stock by the monthly consumption.

Additionally, it's important to monitor expiration dates and take appropriate actions based on this data.

From this perspective, what actions are health facilities and the woreda taking to manage their stock effectively?

R: In our woreda, we monitor the Average Monthly Consumption (AMC) by visiting programs to assess activities, and we also receive monthly reports. As you mentioned, we check for expired items and overstock situations. It is permissible to maintain stock levels equivalent to one month's consumption. However, in our woreda, the maximum stock is set for two months since we receive inputs every month. If stock exceeds the two-month threshold, we decide to transfer excess items from one area to another.

For such decisions and communications, consumption data is recorded on the bill card if utilized, or through data reports if not. All decisions are made collaboratively through discussions and effective communication.

I: Regarding the LMIS, we have discussed various aspects. Do health facilities utilize applications to fill in data, which is then accessed by [RESUPPLYING HUBS]? Additionally, is the data quality filled by the woreda being checked? What actions are being taken in this regard?

R: In this regard, we have had numerous discussions with [RESUPPLYING HUBS] and the regional authorities. However, our woreda has faced challenges in using the applications, as we have been unable to access them; access is currently limited to health centers. Although there was a plan to enable access for follow-up and support of health organizations, this has not yet been achieved.

As a workaround, we ensure that data is collected using both the application and hard copies for follow-up purposes. Each month, we compile the data, review it, and then transfer it to the region. The region also verifies the data's accuracy before sending it to [RESUPPLYING HUBS]. Currently, communication through the application is only between health centers and [RESUPPLYING HUBS], and the woreda and region remain uninformed.

I: is the application is functional still? Are they used it?

R: it is available. But I don't know whether a project owner is used or not. I am not much informed.

I: As a logistics officer, do you receive a copy of the information? If so, do you file it? Following that, do you conduct a preliminary data analysis to monitor data quality and provide feedback to health facilities? This proactive approach helps improve the data quality of the health facilities.

What are your reflections on this process? How effective do you find it in enhancing data quality and supporting health facilities in their operations?

R: We receive data from the IRF and check its quality as it arrives. We provide feedback to health centers and the experts involved whenever improvements or corrections are necessary. However, we do not retain a copy of the data; they bring us a single copy, which we review for any missing or incorrect information to ensure that nothing is out of stock before passing it on to the region. This data typically stays with us for only one or two days, so we do not have a permanent record.

I: when you provide the feedback?

R: soon as it comes

I: Does the feedback help them to improve the data soon or for the next?

R: mainly it is for the next but it may use for the reported input data because we asked them through phone communication looking at the requests. There are cases we correct data by communicating with them.

R: how health posts know supplies are available at the health center and will take it? How they know? How they refill when cases are increased and stakeouts are increased which are factors accelerated stakeout. How they know and request inputs?

R: We have provided support for using bill cards to record usage at both health centers and health posts. There are also experts stationed at health centers who assist health posts, with one expert assigned to connect all health centers with their respective health posts, acting as a focal point for support. When we visit, we collaborate with this focal expert to provide assistance.

However, we often observe issues with form completion due to paper shortages among health post leaders. Additionally, some health extension workers express difficulties in filling out forms due to a lack of training and knowledge. While we have attempted to provide copies and encourage duplication at health centers, budget constraints make this impossible. Consequently, it is challenging to obtain accurate data, but the focal expert can communicate necessary information with the health extension workers.

I: In your evaluation, can health extension workers proactively request and order supplies before a product runs out, especially in situations like emergencies or high case scenarios?

R: Yes, they asked. They have got what in every 15 days and reports if urgency faced.

I: The standard is monthly. Why you make in 15 days? Is there something you faced?

R: yes it is monthly but when new thing is faced like I mentioned earlier for example plumynut, may come for a month but it may asked again within 15days.

I: It is known that health centers have various departments, such as outpatient departments (OPD). How do these departments communicate with the input supply department and with each other?

R: Health centers have a robust section known as the Emergency Technical Committee (ETC), which communicates and discusses input-related issues with the health center manager or delegated pharmacists. This collaboration helps solve any problems related to supplies. In our woreda, there is a challenge with the implementation of the Integrated Financial and Resource Reporting (IFRR) system by health centers. While we have identified this issue, it requires additional budget and paper to initiate. Despite our efforts to raise these concerns, the woreda has not allocated sufficient budget support, which has led to ongoing challenges in addressing the problem and subsequent stock outs.

I: Do they have a schedule in place for services such as family planning?

R: yes.

I: how many times?

R: every 15 days.

I: Is the schedule being executed effectively and is it convenient? Are store managers available on the scheduled days and times?

R: sometimes, the scheduled items may not be available on the designated date. In such cases, it is possible to obtain supplies without adhering strictly to the schedule. Since they are not using the IFRR system, they can request items from the manager using any paper, and the manager approves the request. This allows them to take supplies at any time outside of the scheduled hours if they have run out.

I: what they write on the requesting paper?

R: Letter and what they need. It is not written any other detail data

I: What about behavioral issues? How do store managers respond when asked to provide supplies outside of scheduled times? Are they cooperative? Why is there a schedule in place, and why are these schedules sometimes not followed? One reason could be that the IFRR system is not functional. What other factors contribute to this issue?

R: Another reason could be that the experts are not pharmacists, leading to off dates and off times, which presents significant challenges. In our woreda, fostering commitment among pharmacists,

especially when they are delegated, is a substantial hurdle. Although we have a Telegram group for communication with pharmacists, their participation is not voluntary. Additionally, managers or leaders may shift their responsibilities if they believe that certain tasks are not part of their job description. These factors contribute to the ongoing challenges we face.

I: Do clinical nurses have responsibilities in this area? Do they work in the store if assigned?

R: yes, they do take on these responsibilities. Why do they do it? Because they want to earn overtime pay.

I: Do clinical nurses work overtime, or do they primarily focus on daily routine tasks?

R: They primarily focus on their routine tasks, but they also take on overtime tasks as needed. All of these reasons contribute to the existing problems.

I: do you know the new health extension programs?

R: What kind?

I: A new program has been introduced that is an improvement over the previous one. For instance, it includes comprehensive health posts and emergency health posts, with a focus on integrating a pharmacy department. This integration is important because, in certain situations, preparation is necessary to ensure access to a suitable supply chain for the new program. How does this impact the current situation?

R: Just like pharmacies, it's important to be informed about various programs, especially as terms like "basic" and "comprehensive" have become more common. Each of these categories contains specific details, and understanding the available services under both basic and comprehensive programs is essential for us.

I: it was touched earlier in PMT, PMC and DTC are available in all nine health centers?

R: yes, it is available.

I: how is its functionality?

R: normal it is available but there is a functionality has gaps. We traced this problem and they have communicated and discussed for purchasing purpose only. They don’t have fixed range of communication intervals in month or two months etc. so there is no regular time of communication.

I: in each committee pharmacy experts are the members by default if they are available. What is their role in the committee? How is their role in improving key performance indicators, data reviewing, producing quality data at the woreda or the center level in the committee?

R: there is gap in case of our woreda in this regard; Health centers do not invite pharmacists to set key performance indicators. In this woreda I don't know, may be my boss was participated but we are not oriented. Only management member participated.

I: you are logistics officer, is there any facilitator?

R: Yes there is one facilitator

I: Is there woreda level PMT?

R: yes

I: Do both of you or either of you participated in setting performance indicators?

R: it could be at the woreda level. Yet it is not available in most health centers because of pharmacist shortage. So, others are delegated.

I: Are pharmacy issues not being addressed? Who represents the pharmacy department, and who raise these issues with the management? For instance, clinical issues in PMT may be presented by clinical delegates. What specific pharmacy issues are being raised by the team at both the woreda and health center levels?

R: yes, problems are presented. For example, budget shortage, human resource (expert) problem. To use bill card, IRRF.., to fill gaps, knowledge is required and lack of skilled man power is raised and discussed.

I: What steps should be taken to address the issues related to PMT, DTC, and other programs at the woreda and health center levels? Based on the problems you've identified, what solutions do you suggest?

R: To improve the issues related to PMT, DTC, and other programs at the woreda and health center levels, several actions should be taken. First, it is essential to allocate a sufficient budget and provide the necessary materials, including formats that facilitate the required requests. Second, addressing knowledge gaps through targeted training is crucial. This training should focus on enhancing the skills and competencies of the staff involved. Third, regular support should be provided to strengthen the working capacity of the teams. Finally, it is important to foster a political environment that encourages belief in and advocacy for these initiatives.

I: Please share any exemplary initiatives you have implemented in this regard. Is there anything else you would like to share with us?

R: In our woreda and [RESUPPLYING HUBS], one significant improvement has been the transition from bimonthly commodity supplies to a monthly supply system, which has enhanced

availability. If this trend continues and the direct delivery system improves, we can expect even better support. We have implemented strategies such as providing on-the-spot assistance and sending letters to boost performance, as well as utilizing the IRRF. Additionally, we have prepared formats to facilitate the transfer of supplies to prevent overstocking. We are also actively engaging in discussions to address challenges at the woreda level.

I: We have completed the interview. Thank you for your time!

R: Thank you as well!

KII (17)

KII	KII -A -11	Remark
**Place/region**	**[region]**	
**Organization Name**	**[supplying hub]**	
**Responsibility**	**Distribution expert**	
**Sex**	**[sex}**	
**Experiences**	**14 years**	
**Educational status**	**Bachelor of pharmacy**	
**Interviewer**	**[university lecturer]**	
**Transcriber**	**[university lecturer]**	
**Total Time**	**50 minute**	

I: Given your experience as a vaccine focal currently and your previous role as a distribution officer, how would you describe the effectiveness of the [resupplying hub]input supply system or supply chain management system? Specifically, when we refer to "supply chain," which extends down to the grassroots level, what factors contribute to the effectiveness of this supply system?

R: To discuss the effectiveness of the drug supply system, it is essential to ensure that drugs are accessible at the grassroots level in the required amounts, on time, and of the appropriate types. Over the years, the [RESUPPLYING HUBS] has made significant strides in enhancing accessibility, which is evident in various ways.

One of the key initiatives has been the focus on last-mile delivery. Initially, distribution only reached the woreda level, which meant that lower-level facilities often lacked essential drugs. However, with the launch of the Last Mile Delivery (LMD) and the Facility Inventory Distribution (FID) projects over the past two to three years, we have successfully extended deliveries to health centers.

We conduct assessments of the supply chain every six months, and these evaluations consistently demonstrate its effectiveness. A major factor contributing to this success is our ability to deliver inputs directly to grassroots and outreach levels using appropriate means of transportation. As a result, we have achieved above 98% availability rate of inputs at these lower levels, based on our evaluations of stock availability.

In summary, I can confidently say that our performance has been effective in enhancing drug supply accessibility.

R: We primarily utilize two types of reports: one for dry items (program products) and the RDF reports, which encompass vaccine components. These reports are submitted simultaneously for both categories, which is a significant advantage.

The effectiveness of our system largely hinges on the quality of the reports. If a report lacks validity, it loses its value. Once we receive the reports from the IRRF, we have a dedicated committee responsible for validation and verification. The reports are not directly used as received; instead, they undergo a thorough review process before being integrated into our working system. Our assessments also incorporate the reports. For instance, if a report indicates that stock demand is filled, we must ensure that this is accurately reflected. We have cross-checked the reported quantity with a physical evaluation of the stock in time of assessment. If discrepancies arise, we identify this as a reporting gap and investigate the underlying reasons, which could include knowledge gaps among staff. In such cases, we provide targeted training to address these issues and improve overall reporting accuracy.

I: Is there any problem regarding the quality of reports, particularly in terms of their completeness?

R: So far, we haven't faced any significant problems except for timely submissions. This issue arises due to staff turnover in remote areas and a lack of experience sharing with new employees. When these problems occur, we report them in advance since we maintain early communication via phone. We have already provided phones for this purpose. Additionally, we are working to hire new staff trained in IPLS.

I: So, have health centers been sending fully completed reports?

R: Yes, they have been sending.

I: In general, regarding the input supply chain system from the health system to health posts, have there been any challenges?

R: Thank you for the question. Some inputs may not be completely available, so it's not accurate to say that everything is fully completed. On a national level, we've encountered issues periodically. For example, during the COVID-19 pandemic or malaria outbreaks, there can be gaps in supplies when these emergencies occur. Additionally, our supply donors may not be able to access certain areas on time, which can especially impact family planning supplies.

When shortages happen, there might be regions with excess stock, so we communicate with various [RESUPPLYING HUBS]A branches to facilitate exchanges between areas that have supplies and those that do not. This way, we strive to manage supply gaps effectively. As a result, no one has been harmed due to supply shortages; primarily, the shortages are in storage rather than in the supply itself.

I: What about transportation problems? There may be inaccessible areas, a lack of storage, and issues with expertise during handovers when delivering supplies. What challenges do you face in this regard, particularly in coordinating with health facilities to complete the input supply?

R: We have effective communication and understanding with the store managers as well as the health facility managers. They are aware of the distribution schedule, including the available items, before delivery and distribution begins. Prior to starting the distribution, we ensure that the storage areas are thoroughly checked, especially since expired drugs can accumulate and cause mixed side effects.

Before we initiate the distribution, we communicate with them via message. The distribution is organized by integrating routes; for example, one woreda may have 15 facilities, and all supplies for these facilities are delivered in one vehicle. When transportation starts, we provide the car's identification number, the driver's name, and phone number, as well as an estimated arrival time via message. They follow up on this information to ensure they are prepared to receive the supplies, even on weekends. As a result, we have not encountered any problems in this regard, including with cold chain management and storage.

I: Are all facilities served through direct delivery?

R: Yes, health centers are served.

I: Are there any areas that are difficult for transportation?

R: Yes, there are a few places that pose challenges, but overall, accessibility is quite good. In [zone], there are one or two difficult locations, such as [zone]. However, we have adapted by using smaller vehicles that are suitable for those road conditions. All deliveries reach the health centers—no supplies are left at the woreda level. Overall, transportation has not been a significant challenge, as we utilize various vehicles based on the specific road types.

I: How committed are the staffs regarding sending reports and requests on time with the required amounts?

R: Initially, there were issues in some areas. Some organizations were not sending their reports on time due to a lack of accountability. However, this has since been resolved thanks to the established chain system.

I: What kind of system have you established?

R: We have implemented an integrated chain system led by a woreda focal point. For instance, in [district], if there are 10 health facilities in the woreda, the focal point supports and coordinates by visiting these locations, integrating the reports, and then sending them to us. If a report is not sent, the woreda is held accountable. We communicate directly with the zone, which then communicates with the woreda, making it clear that failing to send reports on schedule is now a matter of accountability. This system effectively addresses reporting issues. Additionally, we have a communication system using Telegram, which makes it easy to send information quickly.

I: Who is the woreda focal person in this system?

R: The woreda focal person is typically a pharmacy expert or any delegated individual who has worked as a pharmacist, often referred to as the logistics focal point.

I: Do you have a system like Telegram or WhatsApp for communication?

R: Yes, we have Telegram groups set up for facilities. Health facilities in regions such as [zones] use it to send reports. However, others found in near areas submit in person coming here.

I: Have you identified any gaps in filling out and sending reports? I'm asking because it's important to understand any issues so we can design appropriate interventions for a four-year project aimed at improving supply chain systems.

R: As mentioned, there can be delays. When delays occur, we communicate closely and urge them to resend the reports. Sometimes, items may be missing from the reports. For example, today I noticed that the Hadiya zone had not sent their report. Upon contacting them, they claimed it had been sent, but I found they had missed one item. This issue can also arise with vaccines. Missing items often occur at the woreda level, but the facilities do send reports to the woreda completely. As soon as I communicate about the missing item, it is sent. Therefore, there are no significant gaps; even cases of delays are resolved quickly through effective communication.

I: What problems have you observed regarding management support for the facilities, including budget allocation, supply chain management, and supervision?

R: There are noticeable gaps in management support, especially when new managers who lack experience in the health sector are assigned. Due to a lack of awareness or insufficient attention,

they often do not allocate adequate budgets. Sometimes, they mistakenly believe that drugs are readily available and inexpensive, while the reality is quite the opposite. Health inputs, such as chemistry machines and laboratory equipment, require substantial funding. One indicator of the low attention given to the sector is the inability of some facilities to pay for drugs supplied to them, citing budget constraints and other reasons. For instance, this week, several facilities expressed their inability to make payments based on agreements and are accused.

I: How many facilities are accused?

R: In [region], approximately 22 facilities are affected. There are also facilities in [zones] among others. In total, around 30 facilities are unable to make payments according to their agreements. This situation reflects inadequate budget allocation. They may have various reasons for this, including health insurance implications and the extensive nature of free drug delivery services. The health ministry does not provide sufficient refunds for these services, which has led to budget shortages.

I: do you find gaps in accessing inputs?

R: We have identified significant gaps, especially concerning the procurement of RDF drugs. Frequently, when facilities attempt to purchase medications, they find that their available funds—usually around one hundred to two hundred thousand—are inadequate to buy even a minimal quantity of drugs. This situation often leads to frustration and emotional responses, as the budget allocated for RDF drug purchases is insufficient, which ultimately affects the overall program budgets.

I: How is the supervision and support structured?

R: We conduct supervision regularly, integrating efforts across regions, zones, and woredas. We have dedicated supervision teams and maintain a supervision schedule to ensure consistent oversight and support.

I: What is the time frame for your supervision? Can you provide details?

R: The time frame varies based on the programs. For instance, for the [IMPLEMENTING PARTNERS] program or direct delivery, we conduct direct supervision every three months.

I: To what level do you supervise?

R: We supervise up to the facility level, with a random selection of facilities included.

I: What about other programs beyond [IMPLEMENTING PARTNERS]?

R: Supervision for other programs is also conducted based on their respective schedules.

I: Is there a known schedule for forecasting?

R: Yes we have and have communicated the schedule, and they conduct supervision accordingly.

I: Are there any gaps in the reporting formats, such as pads, and what are they used for?

R: There are some shortages. For example, we have issues with the bean card, RRF, VRF, IFRR, and other formats used in stores and posts. The bean card is crucial for every transaction at the store, and if it is unavailable, the RRF formats become ineffective. This is important as it indicates the end balance. We supply all these formats, and some, like the bean card, VRF, and RRF, are accessible online and can be printed on plain paper.

I: So, is a soft copy of these formats uploaded to the system?

R: Yes, they are available. We also have copy able pads that we can show you later.

I: So, there are no gaps in formatting?

R: Correct, there are no gaps. We distribute the materials evenly, and in cases of shortages, we address them during our monthly input distribution visits. We inquire about the availability of pads and respond according to their needs. They also monitor how they fill out the bean card and report to woreda-level managers. Therefore, there are no issues with report formatting or shortages.

I: Very good. From what you mentioned, do you refill health facilities every month regularly?

R: Yes, we refill every month.

I: What measures are taken if there is an input shortage in the middle of the month?

R: As I mentioned, everything is scheduled. For instance, if we find ourselves short on drugs in stock, we manage the amount we distribute accordingly. We are aware of our facility numbers and the quantities of drugs distributed. If we anticipate gaps, we report to the head office before distribution and aim to fill those gaps within two days. If there is a shortage at the head office, we may need to reduce the distribution amount, for example, from two to one. In case of emergencies, we can also shift supplies from one area to another. For example, if there is an outbreak of diarrhea in one area, we can allocate additional supplies thereby minimizing distribution from another area and request replenishment from the head office for the supplies that were subtracted.

I: How sufficient are the human resources in the health facilities and [supplying hub], and how are they trained?

R: On our side, has sufficient human resources and vehicles. However, there is a shortage of trained personnel in health facilities, particularly in dispensing roles. It is common to find nurses or laboratory technicians working in dispensing units, but pharmacists are rarely found in rural

areas as they are often not inclined to live there. There are no applicants for pharmacist vacancies in these regions. Additionally, top officials in the health sector have not allocated budgets to increase the number of human resources, claiming there is no funding available for hiring. When we manage to recruit a logistics officer, we attempt to build their capacity in coordination with the region. Overall, there is a critical shortage of health experts, especially pharmacists, with only one pharmacist often covering all tasks day and night, which is quite challenging.

I: How do experience sharing, induction training, and skill transfer occur during turnover and hiring of new staff? Do experienced experts provide on-the-spot sharing for new staff? How is the system organized in this regard?

R: To facilitate this, more time is needed at the woreda level. From my observation, such a system is often lacking, as I have noticed gaps in filling out pads when new staff are hired. Most experts are not allowed to leave, resulting in a disruptive turnover process. Only designated experts demonstrate the work processes. Sometimes, experts may hand over responsibilities to others in the name of working in the dispensary and then leave without returning. If a manager is informed, they may insist that the expert share their knowledge with the new staff. If the departing expert is nearby and knows the new staff, they may share their experiences.

I: Let's discuss quantification and procurement, PMT, performance measurement, etc. Who is responsible for these activities?

R: [name of expert] is responsible for these tasks according to performance indicators. After data is generated, feedback is provided to the organizations.

I: So, is RRF analysis performed?

R: Yes, it is done, but only for selected organizations.

I: What are the major obstacles that challenge last-mile service delivery without interruptions? Can you highlight three or four challenges from the [RESUPPLYING HUBS] perspective?

R: Timely delivery of products relies heavily on submitting reports on time and accurately based on correct consumption data. If reports reach us appropriately, we can deliver accurately. Conversely, if the reports lack accurate data, it leads to gaps and interruptions in input distribution. In some woredas, direct delivery is challenging due to security issues. For example, in [zone], security concerns and power interruptions can disrupt input supply, which affects the timely sending of data and requests. Even if we deliver to the woreda, they may face challenges in delivering to facilities on time.

It is difficult to isolate the challenges within the supply chain. Every level, from top management to grassroots, must fulfill their responsibilities. A single failure can impact the entire supply chain process. Therefore, every responsible party should adhere to their timelines. Strong follow-up and stock status analysis from the national level down to the facility level are essential. Overall, problems at both the facility and managerial levels can negatively impact the supply system if not managed properly. Ensuring product availability at the national level is crucial; otherwise, gaps at the branch level cannot be effectively addressed. Even when product availability is secured, interruptions in supply can still occur if accurate data and proper consumption requests are not provided. Thus, the issues are interconnected and should be viewed comprehensively.

I: Do you have a resupply request process via Telegram?

R: Yes, we do, but we also create hard copies through printouts.

I: Don’t you need stamps for that?

R: A scanned document must be stamped. If the facilities are nearby, they provide us with the original hard copy. However, for those that are farther away, they send the documents via Telegram. They first need to fill out the hard copy completely before scanning it.

I: How much does a power shortage affect refrigerators? Is it a common issue?

R: No, it is not common.

I: Okay, thank you.

R: thank you!

KII (18)

**Name of Project:**	[project]
**ID Code**	KII-A-22
**Type of interview**	KII
**Type of informant**	[sex]
**Region**	[region]
**Role of the Interviewee**	Supply chain coordinator
**Years of experience**	9 years
**Age**	33 years
**Educational Status**	Degree
**Name of Transcriber:**	University Lecturer
**Date of Transcription**	30/08/2024
**Names of Reviewers:**	University Lecturer

I: I have started recording the audio/sound. I have gone through the basic questions. What are your reflections on its effectiveness about supply chain management system? How would you explain it? Please describe the entire process, starting from the region to health centers. For instance, how do you interpret the supply chain and management process? This could include aspects such as accessing commodities on time for health centers and health posts, their readiness to receive those commodities, filling out data promptly, and sending it to the organization. How would you express all of these processes?

R: supply holds the largest share because it is impossible to cascade tasks without inputs. Starting from 2012 EC, the [supplying hub] began delivering directly to health centers with the support of the (LMD) project. Prior to 2012 EC, [RESUPPLYING HUB] delivered supplies to the woreda level, and the woredas were responsible for dispatching these supplies to health centers and health posts. This process was part of the program, and communication occurred primarily at the program level.

I: We can observe this at both the program and RDF levels. As you know, Reproductive, Maternal, Newborn, and Child Health (RMNCH) supplies are distributed through the RDF. What is the overall process of RMNCH and RDF, particularly focusing on quantification within the RDF? Additionally, how is it managed when these systems are not integrated?

R: Programs directly deliver supplies to health centers, covering all associated costs. However, four months ago, starting from April, the project entered its phase-out stage, which has presented significant challenges due to the conclusion of the LMD/FIT project. In this overall process, the organization was responsible for filling requests and sending them to the woreda. The woreda would then aggregate all requests and forward them to the region. Finally, the region would send a request to the RDF and BRF, including vaccines, through the [RESUPPLYING HUB]. Subsequently, the organization became responsible for refilling and receiving supplies at any time, including weekends, based on their consumption needs.

I: How is the data quality? This includes aspects such as formats and ensuring that it is filled out correctly.

R: There is a significant gap in this aspect. Although it has been raised many times, we have been unable to address it effectively. We have attempted to improve the situation through training, but progress has been limited relative to the investment made. The issues in this regard include requesting unused items, inaccurate stock registration, a lack of consumption-based data, and undisclosed stock. Overall, there are numerous gaps in the transaction process. Logistics management also faces many challenges, which can lead to vulnerabilities in emergency situations. This issue requires additional effort and attention to resolve.

I: How do you manage emergencies?

R: As I mentioned, due to inaccurate requests based on consumption, demands are not being accurately refilled. Additionally, there are limitations on the [RESUPPLYING HUB] side. When some organizations, such as hospitals, submit requests using manipulated data, [RESUPPLYING HUB] may not fulfill those requests. This can lead to emergencies. In such cases, organizations can refill and request from [RESUPPLYING HUB] only if stocks are available after the initial refill process. Therefore, organizations handle emergency situations in such away.

I: Does [RESUPPLYING HUB] deliver directly in case of emergencies?

R: No. organizations take it by their own efforts and costs

I: What is the status of the transportation system?

R: The responsibility for transportation lies with the health centers. [RESUPPLYING HUB] is tasked with delivering supplies once a month. However, there is close communication between the health centers and [RESUPPLYING HUB] to avoid wasting resources on unavailable services, especially during emergencies. They verify the availability of required items and needs before proceeding. The health centers will only move forward if [RESUPPLYING HUB] confirms the availability of requested emergency supplies. Overall, emergency transportation is managed by the health centers, while normal requests are fulfilled by [RESUPPLYING HUB] on a monthly basis.

I: What are the major bottlenecks in the process of consistently accessing products without interruption for health organizations?

R: This issue is closely tied to national economic challenges. As a nation, there is an imbalance between demand and supply; while demand continues to increase, supply often decreases and cannot be stabilized. This presents a significant challenge for the country.

Additionally, data inaccuracy, quantification issues, and budget shortages are fundamental obstacles. Many woredas are not allocated sufficient budgets for the RDF, which can be considered a total neglect of this area. Budgets have been consistently decreasing. Some organizations have figures included in their plans but lack corresponding budget allocations. These circumstances pose serious challenges for the future sustainability of these organizations.

I: What is the condition of the infrastructure, including organizational storage, readiness, human resources, and transportation to health centers and health posts? How is the overall situation?

R: Regarding human resources, there is a significant gap, particularly in the availability of pharmacists across health centers and woredas. Some health centers do not have a single pharmacist. For instance, in one woreda with five health centers, there is only one pharmacist available. In such cases, nurses attempt to manage pharmacy-related tasks, but they often lack the necessary commitment, resulting in careless data entry. Because they are forced to take on these responsibilities, they do not record data responsibly. Overall, the gaps related to human resources are quite serious. The situation is relatively better in hospitals, as they have the ability to hire their own staff.

Another significant issue is related to transportation. While there are no remote areas that complicate transportation, many woredas face challenges due to a lack of vehicles. Inputs are delivered directly to health centers by [RESUPPLYING HUB], and there are no issues in this regard. However, transporting inputs to health posts presents challenges. Health extension workers

are responsible for this task, but their ability to do so often depends on their strength and determination.

At times, medications may remain at health centers for a month, and health extension workers may incur transportation costs or even carry supplies on their heads. This makes transportation particularly challenging. Additionally, budget constraints and issues related to drug purchasing further complicate the situation. Fortunately, the road infrastructure is generally navigable for motor vehicles.

I: What solutions are available to address the challenges faced by health posts, particularly in relation to emergency drugs such as vaccines, first aid supplies, MNCH, and other essential items? Considering that health centers receive direct deliveries from [RESUPPLYING HUB], what options can be implemented to improve the situation for health posts?

R: Interviewer: In many cases, health centers play a crucial role in supporting health posts by facilitating access to necessary supplies. However, health extension workers often face significant challenges, as they frequently bear the costs associated with transporting these supplies and accessing services. This situation is particularly difficult given the current economic crisis and rising inflation, as they are compelled to travel long distances by motor vehicle, incurring additional expenses. Overall, transporting supplies from health centers to health posts presents a considerable challenge.

I: How is data being used for decision-making, especially data coming from health centers and health posts? What gaps do you observe in this regard?

R: There is a significant gap in data feasibility. Even within our region and woreda, we lack clarity on the data that [RESUPPLYING HUB] collects from health centers and health posts. [RESUPPLYING HUB] requests data directly and fills it in them; we are merely intermediaries. While we may occasionally see what they are requesting, we do not have information on how much they supply. Consequently, we face challenges in decision-making, as we struggle to determine how much was requested, how much was received, and how much was utilized. Even woredas can only access this information by going directly to the centers and posts.

I: How is the management support structured? Considering you conduct monitoring and supportive visits, what is the situation in this regard? This includes support from your directorate to the woreda, from the woreda to health centers, and from health centers to health posts,

particularly focusing on supply chain management extending from the region to health post supervision.

R: In our directorate, there are gaps related to facilities and inputs. However, we conduct supervision and provide support in coordination with others. As a directorate, we can conduct a maximum of biannual supervisions and visits. Frequent inputs are required to carry out these supervisions, which is challenging in our case. While we manage to conduct supervision once or twice a year, it is done intensively, covering all service items including storage, manpower, and data elements related to RDF. We prepare action plans and provide feedback based on our findings. However, due to the infrequency of supervision, it is difficult to offer ongoing supportive feedback as outlined in the action plan, although we do provide immediate comments and practical support during our visits. Overall, due to shortages in infrastructure, budget, and transportation, we are unable to provide continuous support. Sometimes, woredas may independently address issues with one or two organizations. In cases of integrated supervision involving one or more entities, we can take the opportunity to conduct thorough checkups.

I: What are the critical reasons for the shortage of pharmacists in this region? Why the shortage of pharmacists, midwives, and laboratory technicians is particularly acute compared to other professionals like clinicians?

R: I believe this issue stems from directions given by the health office in the past. At that time, the office instructed that no students should be enrolled in pharmacy programs, assuming there were enough pharmacists available. This decision has created a gap. However, as organizations have expanded and engaged in practical work, the shortage of experts has become critical. The number of organizations has increased from three to five and even more, which contributes to the issue. To be honest, there is currently no shortage of pharmacy experts in the market; the problem lies with the hiring practices of the woreda.

I: What specifically is the problem at the woreda level?

R: The issue is related to hiring practices. There is an excess of clinicians in the woreda, leading to the assumption that they can fulfill the roles of pharmacists to minimize redundancy in efforts. The major challenge here is a resource issue. This concern has even been raised at high levels, but it remains unaddressed due to the capacity and perceptions prevalent within the woreda.

I: So, can we say that the woreda perceives clinicians as capable of performing the tasks of pharmacists?

R: Yes, they are currently handling these responsibilities, even though they lack commitment to consider it their duty.

I: You mentioned earlier that there is a shortage of pharmacists, and they are often replaced by others. When there is turnover and new staff are hired, do the experienced staff share their knowledge and experiences with the newcomers? Do you provide induction training, orientations, and opportunities for experience sharing?

R: This occurs only in rare cases. While we discuss the importance of this practice, we do not implement it intensively. The positive aspect is that turnover mostly happens within the same woreda. However, there is little emphasis on providing training and induction for new employees.

I: What is the reason for this lack of training?

R: The primary issue is a lack of commitment.

I: Regarding integrated skill transfer, there are directives from higher authorities that include providing inductions, guidelines, and working documents. Has anything been initiated in this regard? When skilled staff leave the organization, their experience needs to be shared in a structured way. What activities are being undertaken in this area, including retention mechanisms? What is being done to sustain experts in the workplace?

R: In terms of induction, there may be a one-time formal session, but it lacks continuity and depth. As for retention mechanisms, there are currently no active initiatives in place. More efforts are needed in this area. As you mentioned, during times of resource shortages, losing a skilled individual creates a significant gap, yet the current strategies are not effectively addressing this issue.

I: How is the situation regarding quantification and purchasing? What is the current forecasting process, particularly in relation to RMNCH? How do experts quantify and manage RMNCH commodities and similar items?

R: Orientations have been provided to experts on how to conduct quantifications.

I: Can you explain how RDFs are quantified and processed? Who is responsible for the quantification? When do health facilities submit their data? What is the situation with the budget, and what measures are taken when gaps are identified? Additionally, after [RESUPPLYING HUB] has made purchases, how are the supplies distributed? What does the overall process look like?

R: The quantification process primarily revolves around data quality. In our region, we provide on-site support to selected facilities in collaboration with [RESUPPLYING HUB]. Quantification

was previously conducted twice a year, but it is now done quarterly. Following established directions and standards, quantification occurs in all hospitals, as well as in woreda logistics and one health center in each woreda. While quantifications are conducted in this manner, there are notable gaps in data quality. Furthermore, even when quantification is completed, there is often a budget shortfall related to those quantifications, which is disheartening given the significant investments of time and effort involved. The central office and woreda finance are often unwilling to accept these budget requests. There are issues with data and requests, even when purchases are made based on what has been requested. To address this problem, we have signed agreements with all organizations, based on their service loads, to facilitate annual purchases of up to 30 million, including MCH. This distribution is based on consumption rates, although it does not fully meet all needs. This initiative aims to provide some support by allocating a specific amount, and if they exhaust the assigned budget, they may purchase additional supplies from [RESUPPLYING HUB]. In serious cases, the region may provide subsidies; however, if the woreda hospital has the capacity, it may also support health centers and health posts by supplying drugs to serve the community.

I: Is the agreement part of the reimbursement process?

R: Yes, indirectly, it is part of it, but reimbursement does not cover all expenses.

I: Does the region handle the purchasing?

R: The region is committed to sign agreements with [RESUPPLYING HUB] for purchasing supplies. Therefore, [RESUPPLYING HUB] coordinates purchasing in collaboration with the regional government. Each year, purchasing can reach up to 30 million for all health centers and hospitals, but there can be variations in distribution from year to year. Some organizations may not have additional purchasing options throughout the year, relying solely on these allocated amounts.

I: Why don't the woreda purchases?

R: This is primarily due to the woredas' budget capacity. Additionally, delays in data release could also be a contributing factor.

I: Are you saying that the data aren't provided quickly?

R: It's not just a matter of speed; in some woredas, the data may not be provided at all. They often try to cover expenses using internal revenue.

R: Is the internal revenue sufficient when it is released?

R: No, it’s often inadequate. It might only cover 30% to 50% of what is needed.

I: do funds released timely? How often does it occur?

R In most cases, funds are released towards the end of the budget period. The situation is quite challenging here.

I: So, there are both delays in budget release and shortages in budget allocation?

R: Yes, it’s neither timely nor fully allocated. For example, it could be as low as 30,000 or 50,000 out of hundreds thousands needed.

I: So, the released budget is both limited and delayed?

R: Yes, in some cases, it may not be released at all.

I: So, you’re indicating that there are significant issues with budget allocation, both in terms of amount and timing?

R: Exactly.

I: Let’s discuss data quality. You mentioned that there is low data quality. What do you think contributes to this issue?

R: The issue starts from its source and the transaction process. If there is a gap in any part of the transaction, it can lead to inaccurate data being produced.

I: What do you believe should be done to address this?

R: It requires commitment and additional effort. In our case, I believe we should identify at least a few areas and develop them into models. Trying to perfect everything simultaneously can be challenging, time-consuming, and resource-intensive. Therefore, it’s better to select specific organizations and create model programs through intensive support and training. I believe this approach could serve as a solution.

I: So, you are suggesting that selecting organizations, providing training, and using them as models is a strategy. What other strategies can you suggest? For instance, how should health centers, [RESUPPLYING HUB], the woreda, and the region operate independently?

R: We are currently in discussions with [RESUPPLYING HUB] regarding data quality in the LMD projects. We need to sit down and evaluate the incoming data portals to identify the sources of problems, such as training deficiencies, commitment issues, and guidelines. This will help us determine the necessary interventions to report to the relevant authorities.

I: Are you saying there is a technical team in the region addressing this issue? Is it functional?

R: Yes, it is functional.

I: How often do you meet?

R: We meet monthly.

I: What topics do you primarily discuss during these meetings?

R: We focus on issues like data quality, stock outs, budget shortages, and other concerns involving all program personnel. There are action plans directed by a doctor or office manager. However, sometimes we also engage with emergency issues, which can disrupt our timelines.

I: What is the situation regarding transportation distribution, particularly for RMNCH commodities? Are there any observed issues with input supply options?

R: To be honest, we had been relying on direct deliveries for transportation, but over the last three months, it has become quite challenging. Not all woredas have equal capacity. [RESUPPLYING HUB] has been placing some program inputs at the regional level and making the woredas responsible for collecting and distributing them to health organizations. This has proven difficult due to the varying capacities and resource constraints across woredas. While we have tried to integrate programs, such as using pamphlets, this strategy has not been sufficient to address the challenges. We have also started delivering supplies to those woredas that are unable to transport them from [RESUPPLYING HUB]; using whatever resources we have available, although our stock levels are not adequate. The LMD project assumes that [RESUPPLYING HUB] will handle the distributions.

I: Is this project related to LMD or something else?

R: It also involves ART, but distribution has been particularly challenging. Some commodities are stored at the regional level and then distributed to health facilities.

I: Are these commodities stored in a regional warehouse?

R: Yes, they are stored in our facility. Health facilities can request supplies, and we coordinate with various programs to assess their needs based on service demands and caseloads, such as malaria and TB. We announce availability via telegram, and then they can collect the supplies. If a woreda is unable to manage this, we collaborate with the distribution team to ensure delivery.

I: So, you distribute supplies up to the woreda level?

R: Yes, we distribute only up to the woreda level. Previously, we handled direct deliveries.

I: What about the next step? How do health centers and health posts access their supplies from the woreda?

R: At every stage, the woreda is responsible for delivery. However, there is currently no stock at the woreda level, which makes it challenging. They strive to deliver to the designated facilities since they are accountable for any service interruptions, but it's not as easy as it used to be.

I: So, can we summarize that the main challenge is access to transportation?

R: Yes, exactly. There are no available vehicles. If we had a car, we could manage the deliveries.

I: Moving on to quantifications, when quantifications are submitted by the bottom facilities, who is responsible for aggregating that data? Are the supplies moved directly to the hubs? As you mentioned earlier, there is a committed team, and organizations request supplies based on their budgets and consumption. How does the purchasing process work, and how is distribution handled? Are they receiving supplies regularly?

R: Organizations are actively working on this. Referring back to the earlier data issues, when quantifications are done, we previously faced human resource challenges, but that has improved somewhat. Once facilities complete their quantifications, [RESUPPLYING HUB] is responsible for approving and aggregating them. However, in terms of distribution, they face challenges because the [RESUPPLYING HUB] store often experiences stock outs, making it difficult for them to access quarterly inputs. They typically receive a list of organizations that can access supplies based on their quantifications, but they may not always be able to obtain what they need from [RESUPPLYING HUB]. As a result, they might have to return monthly, especially if their procurement timelines with [RESUPPLYING HUB] and RDF differ. They may also have a frequent procurement plan for emergency purchases, but these are not officially classified as emergencies. Overall, facilities are responsible for purchasing based on their requests, but we and [RESUPPLYING HUB] also compile lists of facilities starting from the budget release I mentioned earlier (30 million). This process occurs when the region releases funds, and [RESUPPLYING HUB] requests that facilities be prepared based on their figures.

I: Who compiles the aggregated list?

R: We compile the list of facilities and then provide it to [RESUPPLYING HUB].

I: Does [RESUPPLYING HUB] have its own quantifications or lists?

R: It includes both quantifications and drug lists.

I: What happens if [RESUPPLYING HUB] refuses the requested item from the list?

R: The facility itself requests and collects preformatted data, then purchases the items by selecting the winning bid. The region also has injection facilities.

I: There's a budget of 30 million for regular RDF commodities. Based on this, they request facility data and communicate with [RESUPPLYING HUB] to secure their needs. Can we say this?

R: Yes.

I: You mentioned that data quality and budget issues are challenges you still face. Are there any additional challenges?

R: Yes, there are challenges related to transportation. These challenges vary by region, especially when it comes to collecting what has been injected. Some woredas are facing difficulties. Recently, they may request items and quantities that are not available in stock.

I: Can we say this situation is irregular?

R: Yes, it can be irregular. They may have their own schedules that relate to [RESUPPLYING HUB]'s stock, which are affected by stock release problems and budget resource shortages.

I: Let's discuss inventory management. How are organizations handling this aspect, such as using formats and placing orders?

R: Inventory trends have improved over time. In the past, it was not effective and there was insufficient support. However, now there is improved support, and we are required to conduct inventory checks. We have implemented measures to prevent inputs from being supplied unless inventory is completed. Overall, there have been some improvements.

I: What specific problems remain?

R: It all comes down to commitment. There's nothing more to it. This relates to professionalism, as I mentioned earlier. Inventory management is carried out by a team with special coordination from the store manager. However, there are issues concerning the store manager related to duty

I: What is the issue?

R: Unlike nurses, the store manager is not compensated for duty, even though they work at all hours. The primary issue stems from the fact that the store manager is not a trained pharmacist by profession.

I: Are there any other issues, particularly related to technical problems like wastage?

R: Yes, there are technical problems, such as discrepancies in reports from different departments. There are about seven indicators that need to be accurately checked and matched with pharmacists before being submitted to [RESUPPLYING HUB]. There's a significant gap in this area. Incorrect data can arise due to the lack of coordination among experts across departments, including

pharmacy and planning. Additionally, there have been internal communication issues between experts from different departments.

I: did you find expired drugs while you go for supervision? Or it could be wastage?

R: The problem of expired drugs is significant and cannot be addressed effectively at a regional level across all organizations. It is a national issue that requires attention too. We attempted to tackle this problem through the Essential Pharmaceutical Supply System ([RESUPPLYING HUB]), but despite a year of efforts under their leadership, we have not seen any meaningful results. I hope this matter will eventually fall outside their scope.

Additionally, when we raise concerns about storage, we encounter a major issue with expired products. Due to storage shortages, all items are often placed together, leading to confusion and mismanagement. Even organizations that successfully identify expired drugs struggle with adequate storage solutions. In our region, a program called "Side Selection" was initiated to organize a team to address this issue, but it has yet to receive approval or become functional. This highlights the broader national problem related to the disposal of expired drugs.

I: Are there any computerized systems in use, like the [eLMIS] system?

R: Yes, some organizations are using it.

I: How many organizations have made it functional? If so, how is it working?

R: Approximately 43 out of 162 facilities in the region are utilizing it. These facilities have started to implement the system after receiving relevant training. However, internet connectivity has become a significant issue, causing frequent interruptions, and some organizations lack access entirely. As I mentioned earlier, the challenges of skill transfer and induction provision are also present. Currently, we have identified around 25 facilities that effectively request and refill using the [eLMIS] system. The remaining 15 organizations are inconsistent due to staff turnover and the need for practical support from [RESUPPLYING HUB]. In this context, assignments have been given to officials to address these issues.

I: you said that you found expired drugs in stores. Was not possible to prevent it? How is a trend to prevent it using different mechanisms like exchange?

R: There is a trend of exchanging drugs between hospitals, and even between different woredas. This situation negatively impacts data quality, leading to the unnecessary accumulation of inputs and excessive refilling, which often results in expired drugs, despite regular communication. For instance, it is unusual to have drugs with six months left before expiration listed in the RDF. The

program has a push system that requires us to take drugs from the Essential Pharmaceutical Supply Agency ([RESUPPLYING HUB]) in combined invoices for both near-expiration and long-expiration drugs. They insist that we cannot receive the long-expiration drugs unless we also accept the near-expiration ones. This practice contributes to the accumulation of expired drugs.

I: How do pharmacists manage the use of drugs that are nearing their expiration date compared to those that are long expired?

R: Yes, they do use them, but these issues still exist and could potentially lead to further problems.

I: What are the other reasons for having expired drugs?

R: There are several factors contributing to the presence of expired drugs. For instance, the exchange of medications between hospitals and different woredas can lead to discrepancies in inventory management. Additionally, the lack of proper tracking and communication can result in the accumulation of drugs that are not used in time. Furthermore, the push system employed by the Essential Pharmaceutical Supply Agency ([RESUPPLYING HUB]) requires facilities to accept both near-expiration and long-expiration drugs together, which can exacerbate the issue of expired medications. There are also problems at the regional level that we should confess. There are drugs come to the region in campaign beyond the required services. There is such kind of pushing systems. There are also such problems related with [RESUPPLYING HUB].

I: What problems could be there in the organizations side?

R: it is what I have said early, that is unnecessary requests. Sometimes there may be blind requests in organizations side and refilling of [RESUPPLYING HUB].

I: How is the management system of expired drugs in general?

R: It is somewhat plan-based, but there are instances where it may not be. Hospitals tend to be proactive in this regard and generally perform well at all levels. However, health centers are not as proactive in prevention efforts. Prevention should be treated as a separate service and should be tailored to meet specific needs. For example, it is essential to identify which items are necessary and which are in excess so that excess supplies can be shared with areas experiencing shortages. This approach should be consistently applied across all institutions. Such initiatives are currently in place, and as a result, we are seeing a reduction in waste.

I: How is the feedback and supervision? Is it at inventory level or general?

R: it is general when it is a time. The same is true for inventory. We announce through call and letter by attaching formats with a fixed time period. By the way, there are two kind of inventory.

For DFSI side inventory is done in every three month. As a region we do have around 7 hospitals and 8 health centers in the DFSI side and conduct inventory every quarter, for none DFSI inventory is done every month in store at regional level.

I: We are currently talking issues with inventory and stock outs. How effective are health posts in identifying, avoiding, and managing stock outs?

R: In the supportive woredas, there is assistance in identifying necessary inputs and requesting needed supplies, which are then delivered using ambulances. Health extension workers may also transport these supplies themselves. To prevent stock outs, they ensure that requests are filled according to a set schedule. However, in emergencies, health centers coordinate with the Essential Pharmaceutical Supply System ([RESUPPLYING HUB]) considering the needs of health posts. Unfortunately, health posts do not engage as much in prevention efforts.

I: how are health extension workers in knowing about stock out before dated? For exampling it could be when various cases happened like malaria.

R: in this regard just I said earlier, whether health center or health post to tell the truth updating bean card and identifying each item expired date, predicting the possible shortage etc. is not that much active. It is not that much strong .so there is no immediate response for outbreak even by [RESUPPLYING HUB]A. Even if it is in outbreak, cases should be selected from each health post, community, and health center and health office to respond. Sometimes [RESUPPLYING HUB] stock may not be in the base of the outbreak. Therefore, this delay of responses for outbreaks led to stock outs frequently in health centers and health posts.

I: so, can we reach on consensus that stock outs happened in health center and health posts due to emergency order is not placed as it happened. For example, emergency orders should be ordered in health centers 0.5 MOS and at health posts 0.25MOS. In health posts mostly not expected more since the capacity is low. It is expected to have a weak stock. They communicate with a store manager and make triggers so that if a stock is below the standard, they order emergency. However, as you said earlier in most situations they order when faced stock outs. So, which one we take, prevention or ordering when stock out as it happen?

R: prevention is not that much. It is mostly ordering due to stock out. This is the reality that happened. Data management for pre prevention and service continuity is a weak trend.

I: what about internal system between pharmacy store and the different departments? I hope all access supplies from the same store, so how is the trend in reporting to the store? What challenges available in this regard?

R: one area of our support focused in this area. We support them to have a schedule to refill every two week in health centers. Delivery should be on the base of refill from the unity. There is also programed template though not implemented accurately. Which is creates a gap in requesting and I think there are some differences with IFRR format. So that requesting out of formats like through letter and head approval may be available. On the other hand, hospitals ask requests upon the schedule. In this case there is a tendency to be depending on systems. In line with this there is problem related with emergency and [RESUPPLYING HUB]. There are some improvements in IFRR by indicating and prioritize requesting items from the unities. However, the refilling issues have gaps in following procedures.

I: Are there any behavioral and technical issues you have observed, such as commitment and punctuality?

R: Yes, there are both behavioral and technical gaps, though they vary from organization to organization. In hospitals, experts, including store managers, tend to be relatively more committed. However, in remote areas, commitment levels decrease, and the adherence to schedules and working days is minimal. This indicates that there are both technical and behavioral problems present.

I: what are the behavioral problems?

R: Lack of commitment

I: some individuals may not report and conscious for each activity, how it could be?

R: this is a commitment issue. Simply, it is lack of commitment and in some situation there is misperception between service and product. They may perceive as no service without product in all cases.

I: How is the availability in the workplace and punctuality?

R: Currently, there are no significant issues in this regard, unlike in the past. We have implemented strong measures to address closed health posts during working hours. We have an initiative called "Work for Change," which aims to improve our working culture and promote loyalty among service providers. We closely monitor this, and health sector leaders diligently follow up with each facility. However, there are still some instances of inconsistency.

I: How is the situation in monitoring units to prevent wastage and misuse of inputs across each facility?

R: Yes, there can be wastage from health centers and health posts. We have made efforts to monitor and control this to minimize it, but it's difficult to claim that our measures are perfect or sufficient. We recognize that improvements are needed, and we don't perceive the current situation as ideal. Misuse of resources is also present in every organization, indicating that there is significant room for improvement.

I: Regarding the strengthening of linkages between health centers and health posts, what opportunities or challenges exist? If the linkage is strong, refilling and supply are assumed to improve. What opportunities are there in this regard, especially to enhance health posts' inputs?

R: There are gaps in supporting health posts with refills and addressing their needs. We tend to focus more on health centers when providing training and on-the-spot support for how to refill and make requests. I believe that more efforts are needed in this area to establish consumption-based requests and improve data quality.

I: How about supervision? Since health posts request supplies from health centers, how is the linkage and supervision managed?

R There are linkages between health posts and health centers in terms of services, but more activities are required for product-related support. Each health center has a focal person, and daily tasks are monitored on a program basis. The health center-health post focal person is responsible for providing continuous support and communication with health posts and for bringing their challenges to the center's attention.

I: To generalize, what are the major challenges in resupplying between health centers and health posts?

R: The primary challenges include the aforementioned technical problems, such as knowledge gaps related to data management and the requesting process, as well as shortages of necessary formats, like the IFRR form.

I: Do health extension workers use IFRR formats?

R: No, they are currently not available and are not being used. Instead, requests are made using white paper with the manager's approval. In the past, they were used at various percentages.

I: What about transportation gaps?

R: For transportation, health centers deliver supplies by ambulance upon the request of health extension workers, if available.

I: Who is responsible for delivering commodities to health posts?

R: Health posts and health extension workers are responsible. However, ambulances are utilized when available, and health extension workers primarily handle the deliveries.

I: What mode of transportation do they use?

R: They typically use motorcycles and may cover costs themselves or carry supplies by hand. Sometimes, there is support from the woreda and health centers for refilling and delivering, especially for pamphlets, which are stored at the woreda level.

I: Do health extension workers cover the costs themselves?

R: Yes, they do so in cases where ambulances are unavailable.

I: So, can we generalize that transportation is a challenge?

R: Yes, it is a significant challenge; a major bottleneck.

I: How are health posts in terms of security? Do they have storage facilities? Are these locked? What challenges do they face in this regard?

R: Most health posts are located in kebeles. Some are close to health centers, which minimize security issues. The storage facilities are locked at the kebele level, and there are security guards in place.

I: With the introduction of comprehensive health posts, how do you see this as an opportunity?

R: It represents an opportunity to provide additional health services. There could be more health extension workers and an expansion of services available to the community. We also expect improvements in product quality. Comprehensive health posts could function as institutions, and there could be a merger of health posts to enhance service delivery.

I: Does this require additional products?

R: Yes, the services provided at basic and comprehensive health posts may differ. As a result, there will be a need for additional products. This will increase product demand, and the supply must be adjusted accordingly.

I: What is the situation regarding data generation, management, and sharing for decision-making across each unit, especially at health facilities? For example, data may be collected using different formats to understand how, who, and what activities are being performed. How is this data

generated for decision-making? Are there PMT and QIT in place? How is data-based decision-making handled?

R: PMTs are available at the managerial level in health centers but exist primarily at hospital levels. QITs are also present. However, there are gaps in data generation and quality, particularly regarding timelines and uniformity. The generated data is not evaluated by PMTs in a detailed manner before sending out. We face challenges in correcting data once it is released online, which necessitates prior evaluation, but gaps persist in this area. There are issues with filling in random numbers across all directorates. On the other hand, parallel data exchange (e.g., between pharmacies and MCHs) is relatively efficient, although there are still timing issues. However, in systems like DHS2, data evaluation by PMTs is not common, which affects data quality.

I: How is the data from DHS2 and logistics?

R: It is parallel data.

I: What are the root causes of data discrepancies between DHS2 and logistics data? You mentioned that sometimes there are discrepancies, with data being scarce at times and excessive at others. Have you tried to trace the root causes?

R: The primary root cause is the lack of communication between individuals involved in the program and the systems. This is a major issue. Due to inadequate communication, there is often the filling of random numbers. We can also attribute commitment and technical gaps to this, and we have traced these issues at the directorate level.

I: When you conduct PMT meetings, do you have a regular schedule and charts?

R: Yes, we do have schedules in various formats, such as figures, despite their inactive nature. PMT performance is generally better at the hospital level. Currently, especially in relation to BMC, there are random supervision visits, and any challenges or interventions are reported within 24 hours at hospitals.

I: Is this documented in minutes?

R: Yes, minutes are taken, even if it happens in the middle of the night. However, this level of documentation and activity is not as strong at health centers.

I: I hope that during your supervision, you check the PMT for its gaps and strengths using secondary data and similar resources. Are pharmacy experts incorporated into the PMT? If so, what is their role?

R: The PMT has guided us to the DTC, which is available, but it primarily focuses on procurement, and this varies across different hospitals or health centers. In stronger organizations, the PMT includes members who play a role in addressing consumption, supplies, and shortages, and then the PMT assigns interventions to fill those gaps. Overall, there are systems in place to manage this.

I: You mentioned that PMTs are strong in hospitals. What are the reasons for the PMTs not being as effective in health centers?

R: A significant factor is the leadership gap. Leaders are not actively collaborating with experts to address operational challenges. Additionally, technical experts are not proactive in evaluating their activities.

I: Are there reasons for this issue beyond leadership and the experts' involvement?

R: Yes, it could also stem from a lack of commitment, which is influenced by perceptions and attitudes.

I: So, the issues you've mentioned are the reasons for the PMT's lack of functionality?

R: Yes, that’s correct.

I: What do you think should be done to resolve these issues?

R: We need to focus on improving commitment and communication, as well as giving proper attention to the products. Since products are distributed through various programs, it’s essential for these programs to prioritize product management.

I: To ensure a smooth supply chain, what systems would you suggest implementing?

R: for smooth supply service it is better to have more partners working on availability. It could be one best solution to strengthen supply chain. This could be one of the best solutions to strengthen the supply chain. Additionally, it’s crucial for higher officials to take a strong stance on budgeting, especially in these economic crisis conditions. If higher officials do not prioritize budget allocation for the health sector, it will pose significant challenges to health services. The House of People’s Representatives should also pay attention to this issue. Furthermore, at the national level, minimizing stock outs requires focused attention. It would also be advantageous to move away from push systems. Building the capacity of staff through training and enhancing their technical knowledge is essential. Additionally, addressing formatting gaps and improving disposal systems is crucial, especially given the shortage of storage, to avoid unnecessary clutter and mitigate risks. Tackling all these issues can contribute significantly to ensuring a smooth service and supply chain.

Addressing resource challenges and providing support for programs, including from the Ministry of Health, is crucial. Programs require adequate resources and intensive support, follow-up, and training. While the ministry occasionally provides training, it is often insufficient, necessitating greater effort. Additionally, human resource experts should be assigned to their respective professions. Overall, this effort demands strong political commitment and dedicated work.

I: You mentioned that prevention is one strategy for avoiding stock outs. How are you currently preventing stock outs, and what mechanisms are you following?

R: We try to reverse or exchange supplies between facilities as much as possible. This mechanism helps prevent service interruptions and input stock outs. However, it is nearly impossible to avoid stock outs of items that have been accumulated over a long period at the national level. For instance, in the case of TB, we are in a critical situation where we can't initiate treatment for patients who test positive. Not only are newly diagnosed patients affected, but those who started treatment are also dropping out due to shortages. Overall, we lack the capacity and resources to manage this effectively on our own, except for exchanging supplies, particularly within programs. I believe that upgrading local manufacturers to produce sufficient products could be a viable solution.

I: You mentioned local manufacturing capacity development as a solution, which is a great point. What other solutions do you recommend?

R: The government should consider options for purchasing from private firms in addition to [RESUPPLYING HUB]. It's essential to have private distributors with balanced and reasonably determined costs. This approach could enhance access to supplies and improve overall accessibility for everyone.

I: private firms sold inputs in free cost

R: yes, it said they are controlled yet not applicable.

I: As a final question, do you have any positive experiences or initiatives you'd like to share with us?

R: When raw materials are available, some hospitals have implemented compounding initiatives. This includes preparing NS on-site and providing topical treatments, such as producing hand sanitizer during the COVID-19 pandemic. However, shortages of raw materials have become a significant barrier, forcing some hospitals to halt these initiatives. For instance, [Hospital] started preparing flood treatments but faced shortages of raw materials. Similarly, [Hospital] initiated

projects as well. If they could receive support in accessing raw materials, it could lead to much better outcomes.

I: We’ve just about finished, but I have one more question regarding the LMD system. You mentioned that you follow a monthly refill system, with a maximum of one month or a minimum of two months. How does this system work? Has it been effective over the last four years? What improvements have you observed with the LMD, and what has been its contribution to the direct delivery system?

R: The system delivers supplies to health centers every month, bypassing the woredas. This has been very supportive for the delivery systems at both the woreda and regional levels, as it minimizes transportation costs. However, we faced significant difficulties in the last three months, which was a major issue. Transportation problems were resolved during the LMD implementation. We believe [RESUPPLYING HUB] is now taking over, but we will need to check on that in a week. Additionally, they have been refilling supplies even an on weekend, which has improved service delivery without having to wait for specific days like Saturday, Sunday, or Monday. This aspect deserves recognition and further enhancement.

I: What percentage of deliveries was made through the direct delivery system?

R: It was 100%.

I: Does this system continue to operate now?

R: Yes, there is such a narrative.

I: How was the stock out tracked each month?

R: Emergency orders accounted for 0.5%, and the health sector also had a 0.5% rate.

I: How is the current stock out situation? Has it decreased, increased, or remained the same?

R: Well, we can’t say that stock outs have completely decreased. Since emergency orders are not tied to stock reductions, we can't claim there has been a decrease. The [RESUPPLYING HUB] refill and the organizational refill need to be aligned for a reduction in stock outs to occur, but this often doesn’t happen. Sometimes, when an emergency arises after a refill, there are still stock outs, and we have systems in place for both refills and emergency requests.

I: We’ve reached the end of our discussion. Thank you very much!

R: Thank you as well!

KII (19)

KII	KII -SP -17	Remark
**Place/region**	**[region]**	
**Role and responsibility of the interviewee**	**Health Extension worker**	
**Sex**	**Female**	
**Educational status**	**Level four**	
**Experience**	**5 years**	
**Interviewer**	**University Lecturer**	
**Total Time**	**51:21 minute**	

I: tell me about your role and responsibility in this health post and how many health extension workers are there?

R: In this health post there are two health extension workers. For the time being I am the only person working here, because one of the health extension worker is in [town] for medical seeking purpose.

I: probing: for how long have you been working as health extension worker here?

R: For 5 years, here for three years and I worked 2 years from another kebele and I am 22 years old person.

I: What services are you giving for the community?

R: We receive commodities from the woreda store keeper, we treat children with ORS and Amoxicillin for those who have like diarrhea and cough. For women like family planning, immunization, health package and hand washing.

I: where do you get commodities to provide services?

R: for instance, if we get shortage we go to [health Center] or the nearest hospital which is 4 K.M far from our health post

I: probing: commonly from which health facility is it from the health Center or the hospital you get drugs? Is that hospital or health center?

R: Commonly we get drugs from the nearest health center called [health center], despite this health post is under [health center]. But the [health center] is appropriate and the nearest facility to get drugs for us. When we get an ambulance for transportation we use [health center] to refill our stock

I: Do you face any challenge in related to stock out at health facility level?

R: Yes. Sometimes we faced stock out challenge. If we face such challenge we go to the [health center] using private car by paying costs from my pocket. Commonly we face shortage of commodities since we are health extension workers. We may not get enough drugs like fluids; it is necessary that somebody may come at night due to snakebite.

I: which commodities are commonly stocked out?

R: Ibuprofen tablet, paracetamol tablet, suppository, Implanon are stocked out currently

I: what should be done to assure the continuous availability of commodities?

R: we call to the woreda if there is shortage here. Then they say why you finished, and it is not a quarter to refill. After we finished we call to woreda and tell the lists of commodities which are out of stock and also we give them report. If there are commodities in the sore, no need to wait the quarter rather we get within the month.

I: what are the main challenges which creates drug shortage in this health post?

R: the problem is not in our side, it is from the store, they said there is no enough stock in the sore. There is no transportation car to transport drugs. We call to the woreda and they respond to us that there is no car or ambulance. If there are serious cases we refer to [health center] for further treatment.

I: Do you conduct inventory at the end of the month?

R: we do drug balance and send report to [health center]**.**

I: How do you request drugs?

R: we report or talk about the available drugs. We use phone SMS or using white paper for request. We get support from the health center and woreda health offices. Sometimes within the month, after two month or quarterly. The woreda store keeper also visit quarterly, on immunization, identify gaps, health extension package like numbers of toilets prepared.

I: What the store manager from the woreda supports you?

R: The store keeper supports on how to manage drugs including not to expose to sun light, asks about the expired dates of the drugs, how to issue and utilize drugs .

I: do you use Model 19, 22?

R: We don’t have model, but have model from the other kebele what I have been before.

I: do the store manager check or conduct inventory?

R: the store man checks expired drugs, conduct inventory in together. The store manager visits us usually every three months.

I: Do you need any training to do better in the future? And what trainings did you take?

R: we received 16 package training, nutrition, EPI/immunization

I: probing: any training in regard to IPLS or stock management?

R: No, we didn.t receive such training, only we got from schooling.

I: any training you need?

R: I need IPLS or drug management, MCH and we need delivery coach, gauze, cut gut, sterilization materials. We use only boiling types of sterilization. We don’t have also HCG kit for check-up before giving family planning methods. We have much drugs like Iron tablet, and deliver health education on nutrition

I: whom are you submitting monthly report?

R: we usually send our report to health center on the day of 21th of the month.

I: When did you request commodities?

R: we request every quarter but there is shortage of supplies. We also request every month and two week. Every quarter we receive commodities. Such as nutrition commodities, family planning, EPI and others. We send our request on phone SMS before send to using SMS we write down on white paper.

I: do you send report every month for resupply?

R: we don’t report monthly.

I: what are the challenges to avail commodities in continuous way?

R: the problem is transportation issues, no car for transportation. We use private transport cars for restocking supplies, after we receive commodities from the store man we wait long time to get ambulance or private car. That is the usual problem.

I: does the store man delivers stocks at health post level or do you go to woreda to pick up stocks?

R: quarterly the store man provides us supplies, in between I go to the woreda store to collect supplies. Quarterly the store man come to for both supervision and refill of supplies. He come up with drugs using woreda car and before provide me the drugs first we count the available commodities in the health post.

I: what would be the solution to avert these problems?

R: the solution would be avail transport car at woreda level, but there is shortage of cars at woreda level.

I: How do send monthly report to the health center? What things you do before sending?

R: Before sending our report to the health facility, we first evaluate it for consistency, coverage, and gaps, identifying who is responsible for each gap. Once the report reaches the Woreda, they provide us with feedback regarding those gaps

I: what about the stocks?

R: When we identify large quantities of stock near their expiration dates, we consult with fellow Health Extension Workers (HEWs) before contacting the Woreda store manager to request redistribution to facilities with higher caseloads. We regularly assess whether our current stock is sufficient for the month while monitoring for short-dated items. Over the last three months, we have not requested any stock transfers, as no medications are currently approaching their expiration dates

I: tell me about the process about support from store manager while he come here for resupply and supervision

R: first we give report to him, then he come up with supplies using woreda car, then asked us the stock out, check the report, count the drugs and if there are stocks, he don't give us and if there is stock out he provide us immediately after verifying the stock.

I: do you use BIN card?

R: we heard that the BIN card will come, but still, we don't receive it. And we don't fill out it.

I: How do you know about the signs of stock out and when do you ask the store manager?

R: we request before we finished it. We evaluate the stock on the shelf and if there are stocks we don't request, if there is small amount we request to refill it. We advise women to take Iron for pregnant women and those who gave birth at home, and also we give education about family planning methods and choices, but the girls or women sometimes afraid to come to the facility for health care services.

I: what suggestions do you have?

R: my advice is to bring drugs like family planning and other drugs and also delivery couches

I: How do you know currently the number of drugs on the shelf?

R: I know all amounts we have. We have small amount that is why we know all the amount. We don't have any list that shows the amount. But we know simply by watching over it.

I: do you have stock of expired drugs currently?

R: When we have expired drugs, we take out from the shelf. And when the store keeper come to us for supervision, he collects it. But for the last three months we don't have expired drugs because we always receive small amount.

I: thank you for your time and we will visit you

R: thank you

KII(20)

	KII -SP -19	Remark
**Place/region**	**[region]**	
**Role and responsibility of the interviewee**	**Supply chain officer**	
**Sex**	**[sex]**	
**Educational status**	**First degree**	
**Interviewer**	**University lecturer**	
**Interviewee**	**Logistics officer**	
**Total Time**	**1:30:07**	

I: Can you tell us in brief your role and responsibility in the Somali Region health bureau?

R: I am working in the {region}region health bureau at the Department of Pharmacy as a Logistics officer. Activities directly related to the supply chain from the [RESUPPLYING HUB], Region, Woreda, and health facilities are undertaken by me. I am in the middle of them. If there is information that you are looking for related to this department I can share it.

I: How do you see the commodity supply chain starting from here the bureau till the health post and on the other side as you know [RESUPPLYING HUB] is there? Therefore, how do explain the administration and the effectiveness of the supply chain system?

R: As a country, the supply is delivered similarly. This means that [RESUPPLYING HUB] provides to the facilities. The role that the region has in this case is that coordination. With the concerned bodies, we are working to fill the gaps that are related to supply. The current supply chain has better performance than the previous one. The direct supply to the health facilities was very poor and the [RESUPPLYING HUB] branches are located in a very far distance. To show you how hard it was, the [region] was using other two resupplying hubs located in two endways. Through time. the resupplying hub was opened in the region and now there is another branch in [location]; both are working well. [RESUPPLYING HUB] is coming near to our facility in direct delivery and the number of health facilities is increasing now at this time. The regional bureau is taking its own intervention between the resupplying hubs and the facilities; based on this there is

an increment in reporting rate and commodity supply. Generally, the situation is getting better in most cases.

I: The main thing is that addressing the commodity on time to the user is the main task, you in collaboration with [RESUPPLYING HUB] tried to address commodities to the facilities directly, but there are facilities that cannot get directly, how are they get and who delivers to them? How is delivering commodities to health centers and health facilities timely, and how is the readiness of the institutions in delivering quality reports?

Respondent: As I have informed you, the delivery is taking place in two modalities such as direct and indirect delivery. In the direct delivery, [RESUPPLYING HUB] addresses the commodities directly to the health facilities these are health centers and hospitals. The other is indirect delivery, in this case, [RESUPPLYING HUB] delivers the commodity to the remote woredas and other places because of road infrastructure and other [RESUPPLYING HUB]'s own issues. After the commodity is reached in the woreda, it is the woreda's responsibility to deliver the commodity to health facilities. By the way, the number of indirect deliveries now at this time is minimizing as the number of direct deliveries is increasing. What we have recently working on is that the city zones which we reach on the direct delivery are small in number, taking a great initiative this month, we are trying to increase the number of facilities under direct delivery. For the practicality of this, the regional bureau developed a proposal because of this two new woredas are the only ones that will be under the indirect delivery modality, but the others will get the commodity directly.

I: What are these woredas that will be addressed in an indirect delivery mood?

R: **[district]** and[district] these are newly established woredas. We recommend these woredas to be in an indirect delivery because of the infrastructure they have. Previously, the reporting system was very poor; even health facilities that are under direct sight delivery were skipped in the commodity provision as they were not properly reporting the RRF. We tried to categorize as even and odd to deliver the supply in a root, in that root when [RESUPPLYING HUB] brings the supply to two or three facilities if they did not report on time or they report lately during the supplies are loaded in this case two or three facilities are skipped. To solve this problem, we created a platform that associates the health facilities and the health bureau are members. Using the platform [RESUPPLYING HUB] provides feedback, and the health bureau also provides feedback; the

reporting rate was 50… now it is around 90. What we are doing now is enhancing the quality, but the reporting problem is solved.

I: How do you manage delivery when an emergency case happens, like the outbreak of a case, though there is a schedule for the delivery?

R: When an emergency case happens, there are various stakeholders who act in time the Ministry of Health, regional health, [RESUPPLYING HUB] hub, are also taking part. Most of the time when an emergency happens central EPHI and [RESUPPLYING HUB] have a discussion and delivery is done by the quota. At that time, necessary commodities that come from [RESUPPLYING HUB] will be directly delivered based on the quota allotted to the place where the emergency is happening following the reporting chain. It will be delivered based on the primary calculation.

I: This is when an emergency has happened. What I want to know is that, if the medicine is depleting before their normal schedule, or the number of users might be increased unexpectedly, so is there a chance of coming and taking the necessary medicines in the middle?

R: Yes, there is.

I: Did not they face the challenge of waiting till the normal schedule?

R: Most of the time such kind of cases did not happen in health program products. If such kind of case happens, they can ask for the medicine they need, but no such kind of case happens here. As I have informed you earlier, if emergency cases happen they are managed in the emergency system. If the case happens they fill the RRF, and they can take it by ambulance since their ambulances]usually come to the region or [regional], or [towns[, they can take it from the place that is near to them, but we do not have the experience of emergency stockout problem.

I: As you told me, you are delivering the commodities in two modalities direct and indirect. During the delivery of the commodities directly or indirectly to address the facility or the use what are the challenges that you face on the side of the health facilities, [RESUPPLYING HUB], and health bureau? To address commodities to the facilities to the user what kind of challenges do you face?

R: There might be some challenges. If you see things on the side of the health bureau a professional who trained how to fill RRF and assigned at the health facility or the woreda might leave his/her

working place, and the new professional who took that responsibility unable to fill the RRF in the similar way because of this an interruption might be happened in that facility. Unless [RESUPPLYING HUB] has got the RRF, you cannot blame it. In this case, it is trying to support that health facility at a distance to fill the RRF; get the correct filled RRF and post it in the platform to stay in a line. Even it is possible to make a follow-up from a distance if you have communication, and good reports can be formulated in this condition.

I: Is there a problem with filling RRF or VRF at the health facilities according to your observation?

R: Such kinds of problems are there since there might be staff transfer, lack of commitment, and other things that make the data quality and reporting rate less than 100%. When you work hard things will be improved.

I: If there is a gap in relation to storage, transport, and others. Can you say something?

R: There is a transport problem particularly [zone] whose number is small. They have two cars one is Pickup. Their catchment area is very wide and they go passing the nearest part of [resupplying hub]. They had one van-car that I knew it got problem once and they enforced to deliver the facilities using another car. Seriously, they have a critical transportation problem. I am not sure if the [resupplying hub] has adequate transport but they have better transportation than the [resupplying hub].

I: Was there a challenge in delivering commodities because of the transport and the road?

R: After shipping the supply with their car, [RESUPPLYING HUB] did not complain about delivering to the health center because of the road, unless the facilities are located in remote areas and unable to deliver directly, and the road is found in the mountains, damaged and is not suitable for huge cars.

I: can we consider road infrastructure as a bottleneck issue?

R: Yes, that is why a woreda which found nearby but the delivery is indirect. For instance, here in [zone], [district 1], [district 2] woredas which are not far but the road geographical structure does not allow the big cars to get on that road because of that the delivery is indirect. Thus, the road is the primary issue, the other the cars, and the third is human power, in the facility there is staff turnover because of this there is a capacity gap.

I: Staff turnover and capacity are the problems, what about the number, is there adequate human power?

R: It is difficult, to find pharmacy professionals in all facilities. In most places, you can find other health professionals who are managing the store, in some you can find the health center head has the key.

I: Is it because of the absence of pharmacy professionals?

R: When the health center head fills out the form, he might miss some and he needs to check in his card. He was not able to fill in correctly, what he has and what he is looking for. There are also other gaps.

I: How do you evaluate the management in relation to assigning budget for the purchasing issue, sometime as you said there might be a capacity gap to fill the format and there might be a format shortage. What are the challenges to preparing an adequate format and providing support on the side of the management?

R: It is regarding RDF so it starts from the health professional that is the pharmacists, and goes to the management. It is difficult to get requests that show the exact quantification of items in kind, amount, and size, based on their previous consumption that the facility requires for the coming year.

I: Okay, I will come later about the quantification and forecasting. I will bring this up later now can you say something about the management team?

R: I am telling you our experience. There is a budget for a certain woreda from that woreda budget some amount is budgeted for health issues from that budget the task division parallel to the budget is adjusted. This is our usual training. This is a simple figure. There is no exact thing that you can access in case of the budget that has been allotted to each task.

I: Who do that?

R: It is regional MOFD to every woreda, and they divide the budget to each sector. So, the health scoter divides its budget into medicine purchasing and other activities. Therefore, the budget is cascaded from the top to the down. So, your main goal is to share that budget very carefully.

I: During your supportive supervision or evaluation how do you evaluate the budget allocation or do you think an adequate budget is allocated, how do you see this? Do you think the work that has been accomplished in the woreda in relation to preparing the format, print, and cascading to the health facility, do you think that would be adequate?

R: Which formats?

I: For instance, the formats that are used for RRF or VRF. The formats are filled in the health facility, does the woreda allocate an adequate budget for printing and producing the formats?

R: No, if we look at the model they are model 19th,22, and RRF. The RRF reporting format is owned by [RESUPPLYING HUB], even though [RESUPPLYING HUB] was by another partner which is withdrawn from this activity. The current training is that by duplicating they bring, if this is not happen they send the soft copy. It is better if they have the hard copy for the filing purpose. There is a great challenge in accessing the model 19th and model 22. RDF is accomplished very fast as it is purchased by the woreda since they hold money. The woreda finance did not want to give model 19th this case was reported to [RESUPPLYING HUB], and they gave it. This is one of the challenges. Model 19 is accessible as it is a model which we use to receive commodities.

I: During delivery, [RESUPPLYING HUB] requires model 19th on the delivery. If you do not have model 19th the donors did not consider the commodities are delivered. This case shows an improvement and we have information for that, how do you see these things currently? Can we consider it a bottleneck issue?

R: Though it can be considered as a bottlenecked case, there are great innervations that have been done. If [RESUPPLYING HUB] reaches the facility carrying the supply and if the fiancé officer who has the model 19th is not around, in collaboration with the disease prevention and division health promotion department model 19th is prepared, and it says only for that program. In the form, there should be the facility or the woreda stamp. This is considered as a proof of delivery.

I: This is not coming through MOFD.

R: Yes, it is. Recently there has one a proclamation that has been approved by the cabinets that all health facility centers should have model 19th and model 22. Concerning mass transactions, the papers that the electronic system produces should get recognition in model 19th and model 22

similar to the hard copy. Any health facility that provides service without using model 19th and model 22, the health facility is considered as the one who opposes the proclamation, because they will be accused.

I: The model that says the health program model is a temporary solution,

R: Yes, it is. A temporary solution.

I: Who is supposed to print this model?

R: The health bureau itself. When the proclamation is in practice, every health post should have model 19th as the rule says any health facility should have model 19th. So, a health post resupplied from the main store they use model 22. This is one improvement. Regarding the Somali region, if any partner comes, or [RESUPPLYING HUB] and we also have a complaint about this issue, this case has got a solution or will get that soon.

I: We were discussing in general, but now we want to have a discussion related to specific issues. The Human power starting from the health bureau to the health post has a standard. So, how do you evaluate the effectiveness of the human commodities capacity, skill, professional assortment, and number of human commodities in managing the supply chain system? Concerning the mentorship that goes you (the logistic team) to the health bureau, the health bureau to the woreda, and the woreda to the health center. What do you say about this?

R: Except for [another region} and [other region], nationally this department is administered by a directorate. Here in our condition, we are working in a case team having three groups such as biomedical, laboratory service, and logistics. When you see things in line with the human resource, the number of facilities, the size of the region, and the size of the woreda, the number of the task force is very small. This happened because of the narrowness of the structure. The structure was developed five years before so now it is a bottled neck issue. Unless there is an open structure, it is difficult to hire professionals; even if there is a need. During the mentorship most of the time we took from the hospital. We are working with them by providing training to cope with the situation. In addition, under the Somali region, there is no zonal health structure. We have six administrative towns and 17 hospitals. The number of health centers is more than two hundred. It is very difficult for three or four professionals to manage all these structures; especially when you go down it is very challenging, when you look at the to, it is blocked. If the zone structure exists

you get a summarized report of each woreda's report. If there was a zonal structure you could manage the logistics in one way to the zone and from the zone to the woreda, but the absence of a zonal structure creates a very complex situation on the supply chain, report, and other tasks.

I: Do you think the woreda has adequate human power?

R: Even though all might not have adequate human power there is at least one logistics officer. Woreda is better compared to zonal health departments and health centers.

I: What about the health post and health center human power?

R: There might be one or two health extension professionals under the health center.

I: In order to add regarding the facility, when we say last-mile delivery we mean that the primary unit care includes primary hospitals, health centers, and health posts. There might be a minimum of two health extension professionals in the health post. The health center has its own structure starting from three health professionals. When you look at this through that lens what do you say?

R: Some of the health centers have a minimum of one pharmacist.

I: Can we say it is adequate?

R: No, we don't say. Since all of the health centers do have not pharmacists. There is a place where more than two pharmacists are found.

I: In the facilities where at least one pharmacist professional does not exist, who is responsible for managing this? Till filling the human power.

R: As I have informed you earlier a professional who is not a pharmacist might manage the store and the dispensary too. When it is reported the non-pharmacist's professional who is in the store or the health center head will report that. We do not have figurative data related to the professional's existence in the health facility and health centers. Lack of the professional interest of graduates to go remote area might be a problem but there are many graduates of pharmacy with diploma. At the regional level from two or three colleges, there are graduates. These graduates are going down, but we have to specify and have a discussion with HR we can manage. In some facilities you can get two or three professionals when you go to long distance you cannot find one.

I: Do you notify the vacant position or what creates this problem?

R: Yes, there was employment, especially after the reform the region was employing and assigning nurses, druggists, and other professionals for different facilities but I do not know why it stops now. The facilities are required to send employment recommendations but the facilities are looking at their budget and based on that they send their recommendation to HR and the office employs the professional that is required by the facility this is the way. As you know it was prohibited to employ new professionals in 2016. At this time, we were employing professionals by the position that a certain professional leaves though the code is not similar the region shifts the budget and employs the professionals based on their supportive letter. If the salary is similar simply employ the pharmacists sent to the facility.

I: How do you evaluate the capacity of the professionals regarding the overall process of purchasing, quantifying, and forecasting children and maternal-related commodities?

R: We are talking about the program supply, most of the time the forecasting of the program supply is accomplished at the national level. The facility can request the supply to the nearest branch by filling the RRF and they cannot quantify. They quantify the RRF concerning the budget sources and amount, is it exactly quantified? Though these inquiries are there, the hospitals are preparing the RRF by themselves. [RESUPPLYING HUB] goes down to some sample woredas and health centers by helping them work on the formats this is the reality. Then the RRF filled by the health center is extrapolated at the regional level. For instance, if the resupplying hub requests a certain amount of **X** – product for the next year is determined based on the data that is collected from sample representative health centers. This RRF will be sent to the center after that things will be managed quarterly.

I: Generalization is done based on these sample representative health centers that fill the form with the guidance of [RESUPPLYING HUB]. Did not they involve the region?

R: Most of the time they invite the region, if we are not engaged in another task we will go. I have the experience of attending this program.

I: What are the main obstacles in relation to quantification, and purchasing, particularly at the lower health facilities interims of data quality, and forecasting accuracy for example there might be cetirizine commodities in the actual time and by looking at the consumption it is forecasted, how are these things in terms of the budget and other issues?

R: For instance, last year's data might be exactly an issue and they did not recode the exact stock out date by using the main card. There might be problems with using Excel professionals. If the data is screened/evaluated this highly exaggerated budget did not come from a single health center. You might get a budget that is forecasted by a single health center that holds the woreda budget. It is impossible to access that amount of budget. This happens because of the capacity and the data usage.

I: How do they perform the purchasing process, the health facilities come and buy themselves from [RESUPPLYING HUB] by formulating a purchasing committee or is it the woreda that buys, or who else performs the purchasing it is about the procurement mechanism?

R: The purchasing follows two modalities; the first one is the perches that are done by the health bureau on behalf of the health facilities, and the other is that the health center or the woreda will buy the commodities and they try to manage by themselves. Currently, most of the purchase for health facility is done by the regional health bureau. There are woredas we purchase the commodities such as [zone] and others. This is because of the absence of pharmacy professionals who can perform this activity accurately. They have a budget that is allocated to medicine. The budget allocation is notified by the letter based on that budget the woreda tried to make division to all sectors. The woreda replied to BOFD after this stage. The budget allocated to the medicine has a code that the budget code can work in this region BOFD. In this case, the woreda health office delegates the health bureau to buy health facilities for a year or quarter. Collecting that from the health offices shift that budget from BOFD to the health bureau. Till the end of the year by dividing the year into three or four seasons we tried to purchase the facilities. The need comes from the woreda itself. As I have informed you is it real and is it that much the consumption of the woreda is in question? We might not get the requested supply in the [RESUPPLYING HUB]. The woreda prepares its own STB document and that shows the specific need of that woreda. Based on that we tried to directly address the woreda.

I: Is the woreda preparing its own?

R: Yes, it is. As per the budget allocation the medicine delivered till the service charge is 3% for the labor and fuel left, every budget should be used for the designed activity as calculated by STB that is prepared by [RESUPPLYING HUB]. This is the way that the health bureau accomplishes activities, and addresses the facilities of the woredas.

I: Therefore, the health bureau purchases the medicine and delivers it to the woreda that delegates them. What kind of transportation mechanisms does the health bureau use?

R: The bureau might use its own cars or if the bureau cars are engaged in another task and the case is very urgent the bureau might rent cars. The advantage that you gain in using the bureau vehicle is cost minimization means that you are expected to cost fuel and allowance. There is a certain amount of budget around 3% that is left for fuel by collecting that and addressing the supply on time to the woreda by renting a vehicle you tried to address the supply in one root. On the other side, the woredas come and buy their supply by themselves for example zones like [zone 1] on the side of [resupplying hub], and [zone], {zone], and others which are selected, and those who have the capacity can buy their commodities. The health centers which are found near[regional capital] purchased by themselves as they have skilled manpower.

I: The main bottleneck is the lack of getting skilled professionals. Can we consider the inability to decentralize the procurement supplying commodities as a blockage?

R: Yes, it is. That is why our bureau has that burden.

I: Who performs the purchasing as you said is it the case team or what?

R: Mainly the logistics case team purchased the supply. They are assigned to the zones and by preparing the needs of each woreda, and by the sequence they adjusted, they deliver and receive model 19th for the supply they address They get model 19th from the woreda finance. Next, they might not receive from finance; they will get from the Woreda health office. Since the rule enforces you to do that.

I: For the next, they will get model 19 from the Woreda health office.

R: Yes, they will receive from the Woreda health office, but till now they are receiving from the Woreda finance.

I: So, the [RESUPPLYING HUB] case team will give up there in the woreda.

Respondent: Yes, they stay there, and we bring our one copy and model 19th.

I: Inability to decentralize procurements, lack of accessing issuing and receiving models in all facilities centers, and the three points we have mentioned earlier are the obstacles. Well, we are

going in a good manner in addressing our discussion questions. Having said this, there might be supplies that are not purchased as the forecast is not committed demand, this might happen because of the budget shortage. Did [RESUPPLYING HUB] purchase based on their committed demand or, for instance, a certain facility quantifies one million birrs, does the woreda or the health bureau purchase that or what?

R: Do you mean the woreda has a budget of one million?

I: Are they quantifying based on their budget?

R: No, sometimes it might be low or high. Almost 99% of them quantify over their budget., that is why [RESUPPLYING HUB] itself goes to the facilities and prepares the forecast; therefore, the purchasing is performed based on the [RESUPPLYING HUB] forecasting.

I: When you go for pipeline monitoring and supervision, do you have the experience of looking especially the revolving drug fund usage? In the revolving drug fund RMNCH commodities are there like antibiotics though the reimbursement is stopped the medicines are purchased and delivered to them like cetirizine, what kind of RMNCH commodities are mostly purchased by the RDF?

R: Already they are using the RDF and purchased Cefetrizole, Gentamicin, Gloves, and others. In the previous time, there was reimbursement but now there is reimbursement, So, they purchased and use or if the facility head believes the woman cannot afford they might cover that.

I: What about health care finance, do not they work this?

R: It is not well going here.

I: You told us many points related to transportation and distribution, but we are looking to tell general ideas related to especially [RESUPPLYING HUB] emergency order managing technique related to RMNCH commodities, for instance, if a health facility comes in the middle and requests an energy medicine does [RESUPPLYING HUB] itself deliver the medicine or not? Sometimes there might be a case surge like malaria, in this case, if [RESUPPLYING HUB] does not have a vehicle searching alternative vehicles as you informed there is mobility of private vehicles. As you informed us direct delivery is increasing in relation to the spread of direct delivery how do you manage back orders? This case might happen as the facilities order certain medicine and if it is not

in stock and after some time that medicine is available, is there a culture of delivering that medicine for the health facilities?

R: No, if they request again, their request might be considered here, but no experience in delivering medicines that were asked for the previous time and they were stockout. The emergency case is difficult usually they come by ambulance and take the medicine they are looking for. For health facilities that are found in a remote area if they are looking for one or two medicines and trying to address that medicine seems theoretical. Even if such a case happens in Addis Ababa I do not think they manage this. You might manage by certain system by the ambulance or the person who came for the report might take that.

I: How do you see the last-mile delivery flexibility, responsiveness efficiency? Nowadays, last-mile delivery is widening and your status is about 75%, it is good practice how is the practice of responding on time to the requests of the health facilities growing parallelly? Is the delivery performed based on the needed time/ schedule?

R: We did not hear a complaint about the delay of [RESUPPLYING HUB] delivery. The previous time, there was a complaint about the delivery I sent the report two times but still, I did not receive kind of the complaints were there. There might be some but if it happens, the delivery might not be reached at the exact time that they are waiting for one- or two-day delay might happen because of the road or some other cases. Now at this time, no report related to the absence of resupply cases.

I: What will happen if they do not report?

R: They will be skipped.

I: The professionals are the ones who did not report, and because of that [RESUPPLYING HUB] did not resupply. Is there any measure that [RESUPPLYING HUB] takes about this case as we are considering the end users? How many facilities are skipped because they are not reporting on time?

R: These practices are stopped now; a rare number of facilities are reporting in relation to delivery skipping because of their report delay. If it happens and occurs, you blame the facility itself. Feedback is given to the Woreda health office about the seriousness of the case, they are very good at listening to the health bureau. They are enhancing their commitment. In the previous time

commitment was a very challenging issue, but now they have respect and they try to respond to the inquiries though they cannot keep the quality and time. The quality and number of reports are increasing when your attitude is progressing, so it is going well.

I: Till now we have been discussing addressing the supply up to the woreda after this who is responsible for addressing the supply to the health center and from the health center to the health post is there any structure this activity is governed? Is it the health center to come and collect from the woreda; similarly, does the health center need to deliver to the health post, or should the health post come and collect the supply? What is the role of the region?

R: If the woreda is under the indirect supply modality when [RESUPPLYING HUB] delivers the supply to the woreda after that the woreda tries to deliver the supply to the health center by its ambulance in doing this no refund and compensation related to the fuel and allowance is performed by the woreda commitment and responsibility. After that, the health extension comes and collects from the health center.

I: IPLS has a gap in its SOP I do not know if it is revised or not, but it does not say about the way the delivery needs to be done after the woreda. This means it does not say the woreda should be delivered to the health center or that the health center come and collect the supply for an indirect sight. Can we conspire this as a hindrance?

R: It is for an indirect delivery and yes, it does not say anything it stopped there in the woreda. Even though it is not written in a document if the supply is reached at the woreda there is a belief that the woreda will be distributed by making the breakdown and this kind of assumption is already there.

I: Is there any discrepancy that the supply is in the woreda and it is stocked out in the health facilities?

R: No, surely more than 90% of the woredas have their own vehicle and the health centers have an ambulance. If there is a supply in the woreda and it is stockout from the health center or if [RESUPPLYING HUB] deposits the medicine there in the woreda they come and take it by their own vehicle.

I: What about this case if the supply is in the health center and it is stockout in the health post?

R: The health extension will collect that.

I: When you go out for supervision and support did you get the family planning commodity which is available in the health center but is stockout in the health post?

R: Yes, there might be commodities because of certain issues.

I: What transportation system are they using?

R: Motorcycle, ambulance.

I: Did you see any bottleneck issues around that between the health post and the health center?

R: When we speak frankly sometimes the health post directly comes to the woreda and collects/takes the medicine, but this is not a structure it is practical. The main target is addressing the supply. There are health centers that act as health posts since their site are found in a remote area where there are no health posts around them.

I: Is it because of the nearest woreda or because of some health centers irresponsiveness?

R: It might be because of both cases, or in one of the two cases the health post takes its medicine from the woreda. It means irresponsiveness of the health center and its distance to the health center means that the health center might be located a far distance from the health post.

I: Irresponsiveness and the distance, are the bottlenecks for this case.

R: Yes, it is, but when this case is managed based on the structure things will get solutions. In one woreda there are three or four health posts, on the other hand in one woreda there might be one health center but there might be nearly twenty health posts. The law has stated that one health center needs to compile five health posts but in the woreda, there is only one health center but twenty health posts how can this issue be managed? Therefore, this and other cases are pushing them to go to the woreda.

I: Irresponsiveness, if the health center is placed far from the health center, and when the health center is overloaded (the number of health posts is many in number) in this case the health posts go to the woreda to bring their medicine.

R: Yes, that is right. Do you know the drive initiative, it program? Some health centers try to provide a motorcycle to address high-to-reach areas health posts use that motorcycle. At an

entrepreneur's office, a motorcycle is given to a certain young man. In a month there are thirty days for ten days he helps the health centers by addressing vaccines and supplies, whereas the rest twenty days he uses the motorcycle for his own purpose.

I: S the health post uses ten days the motorcycle, who he is going to serve? Is the health post of the health center?

R: There is one health post which is located in a remote area and there is no transportation system. In this case, the health extension knows her program and she have remote outreach sites so he went there and took her to the place where he was going to deliver the service and he took her back to her workplace.

I: Does he serve one health post only?

R: Within these ten days he might provide service to three of the health posts out of five. He is working on this in collaboration with the health center.

I: For whom he is accountable?

R: He is accountable to the health center.

I: Is it called the Drive initiative?

R: Yes, it is, and the fuel cost is covered by the project itself.

I: As you know the higher levels of health ministry, health bureau, or [RESUPPLYING HUB] have the capacity, to be honest, what we are looking for is the lower-level health facilities and such kinds of new creatives are necessary.

R: This young boy not only addressed vaccines but also other supplies from the health center to the health post.

I: Is there any obstacle that you have seen around the health center and the health post?

R: The main challenge is the absence of the zone structure, and the inability to cascade systems Somali region has Eleven zones and 17 hospitals among these only five of them are general hospitals, and these are the hospitals that should contact the region. If the zonal structure exists we are communicating only the five general hospitals and the eleven zones, because of this the woreda, the zone, and the health centers come to the region. In the region, there might not be a responsible

person who can provide a response in that amount. When you go to the woreda lack of professionals and staff turnover are the other cases. Lack of commitment the leader is expected primarily to discharge his own responsibility and push the other professionals to act accordingly but there is a lack of such kind of commitment. There is improvement in the commitment, but still, it needs improvement.

I: The point you mentioned Drive initiative is a very good point.

R: Yes, it is. In our region, we implement this program for eleven woredas which are very challenging sights.

I: Is it functional?

R: Not yet, it will be launched and these eleven woredas are expected to select facilities, after that it will be started.

I: Is it a UNICEF program?

R: Yes, it is.

I: what kind of motor are they, is it possible to load commodities?

R: Yes, it is Yamaha Motors.

Interviewer: Is there any other transport modality from the woreda to the health center and from the health center to the health post?

R: As I have informed you earlier only the woredas that have hard geographical problems most of the woreda health office have adequate vehicles that can be moved around the town. If the woreda does not have cars at least they have a car and the health center has one ambulance. According to the Somali region, most zones have vehicles and they can manage by themselves., but the problem is the human power and the structure.

I: Is it a case of monitoring and evaluation?

R: Yes, this is the hindrance to strengthening monitoring and evaluation.

I: Yes, I understand and tried to align with that, for instance, if the supply arrives at the woreda within how many days does the woreda need to be distributed, and when the supply reaches the

health center within how many days does it transfer to the health post and the health post to the facility is obviously known that it is delivered on the fifth days, the request is taking place monthly. So, within hominy days the woreda need to address the supply five days or ten days?

R: There is no preplanned day for delivery; thus, the woreda might decide to take after the supply reaches it or the health centers send an ambulance and take the supply. There is no written thing enforced to have preplanned days.

I: You thoroughly informed us about transport issues, how do you evaluate the practicality of integrated pharmaceutical logistic system (**IPLS)** in all health institutions, health centers, health posts, and hospitals? What is your outlooking concerning issue? For instance, correctly using and filling the formats, or it might be about ordering emergency drugs and the first in first out related to the expiration day of the medicine, the system is working to strengthen general pharmaceutical activities. What do you say about this special store and dispensary?

R: IPLS is practiced in direct delivery sites. Though the reporting rate and the data quality have a problem the direct delivery sites correctly implement IPLS and use that they are receiving their requests, this is because most of the institutions have professionals. Regarding supply management, when you get to the store all of them might not use FIFO or LIFO. Even though they are not following every step they are managing the supply. At least you can see clean stores and you cannot find non-pharmaceutical things with the main drugs. In this case, things are better than the previous time. Concerning the format [RESUPPLYING HUB] was providing and now it also providing, and using softcopy they are reporting based on that they will be resupplied. The indirect is also in a similar way their woreda prepare that and they will be resupply.

I: You have mentioned one main point related to inventory management which is segregation, i.e., management of expired medicine, the second is that if there is overstock and understock supplies there should be an act of balancing them, or stock exchanging, the third is that stock status analysis, by conducting this it is necessary to identify over stock and under stock if there is the stock transfer that should be done, and managing the expired supplies. From the perspective of this, how do you see the IPLS?

R: Most of the time stock status analysis is conducted in the hospitals. In the woreda by doing stock analysis we have such consumption what can we do kind of inquiries are not there. The

minimum activity they perform is that they identify expired medicines from non-expired ones and put expired medicines off the shelf. Standard activities are performed in the hospital.

I: There is no such thing in the health center.

R: Yes, there is no. But in the hospital, this case is performed till the stock exchange. The supply that is overloaded in[Hospital]will be sent to[Hospital] or vice versa.

I: So, can consider a weak inventory-controlling system as one bottleneck?

Rt: Yes, it can be.

I: Are doing a supportive follow-up in relation to stock management at the health center, woreda, or health post? For instance, in identifying expired medicine from unexpired ones, placing, avoiding, reordering medicine based on their dates, and addressing the two uses and the medicines which have dates placing them to the back?

R: Most of the time we perform this activity in the hospital based on human power. The first thing is that we do not have a supervision budget, the hospitals are located at a far distance, so based on our efforts that we have we perform these activities in the hospitals. MNA is fully applied in hospitals and they report MNA they know their wastage rate. MNA supply chain pharmaceutical services, including biomedical management these all things are implemented in the hospitals. Now, we are going to the electronics. we start IPAFGS, In the hospitals there is very good performance according to these issues. In this case, if you have adequate human power and the structure is in the woreda you can say that I am serving and supporting all woredas properly but these things are hindrances to performing this.

I: Did the electronic logistic management information system (eLMIS) start?

R: [ELMIS] is working here.

I: Do all institutions use [ELMIS] properly?

R: [ELMIS] is working in the hospitals and this year, we started in ten health centers.

Interviewer: Do all institutions use [ELMIS] to upload their reports on the computer?

R: Yes, but there is an on/off case.

I: What is the reason for that?

R: Most of the time technical computer failure. Application, there is a networking problem unable to link and synchronize as the connection is needed. Up to the national level they tried to synchronize. No, the challenge is in the woreda and the health center.

I: So now ten woredas are using [ELMIS]?

R: In recent times they tried to customize and make the inventory. It was a very recent time that we trained and gave them a computer.

I: If the system is functional and they are using it properly, in this case, what would be the hindrance to [ELMIS] not functioning properly?

R: Technical challenges, computer failure, connection problems these are the problems.

I: How is the support related to [ELMIS]?

R: Once there was the withdrawal of the professional and the other professional was giving birth at that time we faced a great challenge to managing all sight support because of that there were on/off. Now they are coming back to the proper performance. Except for [hospital]and [hospitals] are in a challenge as they are far in a distance. The technical team in your organization was once there but they cannot go again.

I: How does it look like the health facility internal report from store to dispensary from dispensary to family planning, OPID, emergency they shift stock? There is an emergency that is taken out from the dispensary, family planning is also the same, and ART these all taken out to each department. How is the staff internal report exchange, communication, and commitment to providing services such as family planning, ART, and OPID following a designed schedule? How does staff commitment mean that certain medicines are available in some place but without carefully checking that they are not, in such cases and others, how are interdepartmental connection, and commitment?

R: A harmonized relationship within the facility works based on the standard found in the hospitals at a good performance. Managing things by the schedule, and requesting using IFRR is practiced in the hospital too. In the health center if the dispensary finishes the supply he asks by written paper and the health facility approves that and it is given to the store. The paper means the form

that the health center develops and uses for such cases. They get to their own dispensary. Most of the health centers simply request by their hand instead of IFRR.

I: DO they have model 19 ?

R: Yes, they have, and they used to take out supplies from the center.

I: Are they reporting weekly or monthly about the supply they used for instance family planning commodity, this amount is used for this week and this amount is left.

R: It is not that strong in the health center it is better in the hospitals.

I: The weak performance of the internal system can be considered as a bottleneck, is there the possibility of stockout though the supply is in the stock?

R: Yes, there might be such a case. Especially I have observed that MCH products are available. When you go to the MCH department and ask them to show you they tried to show you two steps and they said no other means. When you go to the store that product is available. Most of the time they use the method they are accustomed to. Nurses in the MCH are not ready to get into the new system and they are resistant. Only they use the one they know very well but the rest choice might not be considered by them.

I: Where do the health posts get the medicine?

R: They may get from the woreda, health center, health facility

I: As you know the new health extension packaging program is coming and the comprehensive health post is in consideration. Has the comprehensive health post started or not?

R: Is it they called second generation?

I: Yes, they are.

R: In our department, this case did not come, but there are selected health posts that have beds. They are called second-generation health posts. There is no linkage with us.

I: Since product release, harmonization and …

R: We did not do that much we have left so many things.

I: Okay, when we say facility mainly we are focusing on the health post and the health center, what is your observation about data generating, sharing, and using for decision making since decisions should be made based on the data how is this thing especially the health center?

R: It is the reporting system.

I: Reporting is one of the platforms for instance there might be MT those who evaluate should be there like the Performance management team, and quality improvement team and there may be other teams. These teams can generate, strengthen, and use the weekly, monthly, and two-week output. of each department concerning this, what is your saying?

R: Data generating, sharing, and using for innervation is again formally available in the hospitals, but no one can check the data that come from health centers. It is not strong.

I: Are there teams in the health centers?

R: No any team.

I: What about the PMT, and QIT in the health center?

R: We did not formally formulate specific teams for quality issues, and for report purposes; in the reporting process we gather reports that come from the hospitals at the same time we try to see things related to this case when there is a reviewing meeting. We do not have a team or committee formation for such an issue.

I: How do they send reports, are they simply sending what the heads compiled, is there evaluating and checking the report?

R: In the hospitals, there is a test report therefore the pharmacy goes and studies its own part, the finance and the account do their own task and they discuss together after that they report to us. What we do is we try to provide feedback by considering the previous report, for the next if they have something to improve they will consider the feedback. MNU has also gone through the same path in a quarter we tried to share with the health minister. We provide training for the ten health centers to use MNU.

I: Is the pharmacy team represented in the quality improvement team? Are they providing data, evaluating the patient satisfaction data, and stockout?

R: From the general health service they evaluate pharmacy dispensary services in relation to patient satisfaction, and it has complied with the facility report in general.

Interviewer: When you go out for supportive supervision together did you go to the health center?

R: Yes, we were there but it was a very long time that we did that. It is human commodities and budget shortages are obstacles to performing supportive supervision. If you have got a chance to visit certain health centers and provide feedback and if you are there sometime later you will see improvement in the comments that you provided this is what I have experienced. Some people are saying that supportive supervision is a motivational factor. So, I have seen this case by myself. When you tell them to do something you show them how to continue and if you go back after some time you will observe the change based on your guidance/feedback. Our communication has become better. When you disappear for a long time around the health centers the activities will be (degraded) vanishes.

I: Regarding data use there is a problem at the national level too but it shows improvement, as you mentioned data use in the health center is very weak it is better in hospitals. Most of the time we classified data use in three perspectives such as technical organizational and incentivization (behavior). So, is there any bottleneck that you know related to these three perspectives?

R: when you see in general technically they generated data and report after they report you want to be clear about the report and you request them to clarify but they did not know what they mean in the report, in the feedback unless the issue is clear by itself they did not provide specific meaning by cascading that issue, this requires working jointly with prescriber, senior manager, and pharmacy head. This is what data use means, but somehow there is a gap.

I: Does the regional bureau have a supportive system for data use, PMD, and facilities?

R: No.

I: There is a shortage of organizational support related to data use.

R: Yes.

I: What about behavior issues related to data use?

R: What do you mean by behavior?

I: Behavior means, lack of attention or attitude of the head in using the data to make decisions.

R: I have informed you that, if there is positive commitment starting from a person is required to work and improve the service. So, there is little carelessness on the side of the professionals.

I: Thank you very much, in relation to supply chain management in general, can you share with us a good experience that you have to manage a problem-solving mechanism? Which do you consider that can be a lesson or an example for others? If you have something to scale up to the future; sometimes we have performed good things but we cannot consider them as good things that can be shared with others, do you have something to say about this issue?

R: Thank you, in this case, I want to forward two points one the IPLS was very poor, and we tried to solve this case by assigning a pharmacist to each zone. The professional who assigned there is coordinates among the zone, woreda, facilities, and [RESUPPLYING HUB] professionals. We have created a WhatsApp group and each health facility posts its own report there, we communicate with [RESUPPLYING HUB], and if there is a late facility that did not report we can see it there. So, this platform creates internal competition not to be blamed and exceeded by others the reporting rate which was 52% is now getting 90%. Now a day we try to calm down the disturbance that were in the health facilities. The second is national data synchronization (dashboard) in relation to this there was competition so that the Somali region stood second at the national level. Using the same system means that we create a WhatsApp platform and by calling the names of each health facility we were commenting them to synchronize model 19th. So that all facilities synchronize the data on the due date. Even though health facilities do not have a connection. Also tried to synchronize. Some of them tried to use the regional mass media bureau, and because of this, we achieved good changes. Creating a platform is a very supportive mechanism in relation to creating positive competition. This helps you to come to the position which you are looking for.

I: thank you for your time

R: thank you

KII (21)

KII	KII -SP -18	Remark
**Place/region**	**[region]**	
**Place of work**	**[health facility]**	
**Role and responsibility of the interviewee**	**Store manager**	
**Sex**	**[sex]**	
**Educational status**	**Diploma**	
**Experiences**	**Seven years**	
**Interviewer**	**University Lecturer**	
**Total Time**	**1:52**	

I: Tell us about your role and responsibility in this health facility

R: I am the store keeper or the store man of the woreda, I request drugs quarterly for the woreda and I give medical supplies and drugs for the dispensary and other departments, as well as for 16 health posts.

I: probing: when do you request commodities usually?

R: I request from [RESUPPLYING HUB] quarterly, and I report every two month for the program and every three month for RDF. The program report doesn't go to the health office. After the [RESUPPLYING HUB] manager signed it and the finance office pay the cost and we receive the drug with model 19, the health post, and MCH and laboratory request commodities. Every week the departments from the health center request and receive commodities which is every Friday of the week. First they write down on the request paper that is Facility requisition format, they have BIN cards on the departments and they fill out or update it, then they go to the CEO of the health center, after signed and had stamped I prepared the requested commodities with issue Model and with [ELMIS] system. The supply chain management system was very low before, now after [ELMIS] system established it is currently very good.

I: How the MCH department request commodities?

R: using this request…….. (Showed us the sample), that is internal facility requisition format. Without using this requisition, I don't give any commodities for each department.

I: probing: do the departments fill out the requisition format properly?

R: I don't give them any item if there is any error in the internal reporting format what they filled out.

I: How do you see the effectiveness of the supply chain management system in this woreda?

R: the supply chain management in the woreda was very low, but currently it is very high after I got training on supply chain management system. I don't give any commodities without completing request. If they bring request, they can receive drugs. In the previous time each department don't request commodities using internal resupply format, in this case I also don't give the commodities simply. This is the problem of the health professionals in every department. And also, in the previous time the health professionals in every department request after they finished what they have on hand or zero balance, and I give them gloves or other commodities when patient is on board. Consumption was not known; they didn't update the BIN card. But now the supply chain management quality is high, there is now internal facility report and resupply system. Without internal facility resupply, I don't give any supplies. This is due to the new system.

I: probing: when this new system started?

R: It has been three years, the new supply chain management system. The [ELMIS] system is also currently functional.

I: How long the health care professionals started to request using IFRR?

R: it is with the [ELMIS] system that is starting from the last three months. Both the [ELMIS] system and IFRR started at the same time.

I: probing: what was you use before [ELMIS]?

R: we used a white paper for the requisition. But approval from the CEO is mandatory. If the commodities are available in the store, I give them. The challenge was the health care providers didn't use consumption balance. After we started to use IFRR, the health care providers use BIN card and provide me consumption balance. Now currently we get all data from the BIN card and I provide the necessary supplies after review the number of commodities they taken out from the store and the current balance on hand. Now I provided on job trainings on how to fill out IFRR about the department heads like MCH, laboratory and others. They also used BIN card previously.

I: Do you think that all necessary commodities are available in the store?

R: currently there is a problem on [RESUPPLYING HUB] side. [RESUPPLYING HUB] manager told us they are currently on system update; therefore, we are under stock out like Implanon and I called them that we are finished it and [RESUPPLYING HUB] told us to ask in emergency order.
I: what is the reason still you are waited until zero balance?
R: because of system change at [RESUPPLYING HUB]. We ordered by emergency order yesterday. The only stocked out item is Implanon. Previously we received every item every two months for program. No problem at [RESUPPLYING HUB] side. The only problem is quality request..
I: Do the [RESUPPLYING HUB] provide commodities at the facility level or not?
R: No, since it is an emergency order, I should go to [RESUPPLYING HUB] site and collect the necessary commodities. I should have also issue model for signature and to get stamp from the manager. For the regular request [RESUPPLYING HUB] come up with the commodities at facility level for quarterly level.
I: is the store sufficient?
R: it is enough
I: Some commodities are still placed on the floor; this is because of shortage of shelfs.
R: I told the health bureau about the issue. The other problem is medical supply in regarding to MCH kit, previously we got from an , but now there is shortage of MCH kits like delivery kits in the store after phased out from our woreda.
I: are there sufficient pharmacy professionals here?
R: there are enough pharmacy professionals. I think six druggist are there in the health center. Some of them have taken training. I have worked in the health center as both dispensary and store man at that time there were no enough pharmacy professionals. And I requested the woreda health office that the dispensary and the store man should be separated and professionals should be assigned for each. Now we have sufficient pharmacy professionals.
I: How health extension workers received commodities and when?
R: health posts are provided commodities for every three month. Health extension workers request using monthly report request format or by phone call or sometimes using SMS. After filling the request format which is white paper, they should get an approval from the health center CEO to take commodities from the store.

I: how about skill transfer from one professional to others or induction training for the new employed professionals?

R: that is mandatory, we give onsite training about the issue models, drugs, prices

I: How do purchase drugs for those RDF?

R: we have DTC and make quantification yearly; we have a telegram channel and make quantification for 2017. Assume we add 10% from the budget. For example, what amount of ceftriaxone needed for 2017, let say if we used 2000 above ceftriaxone for 2016, what amount of ceftriaxone is needed for 2017 including emergency order and assuming the mount needed in a quarter. Then we request [RESUPPLYING HUB] about the number of drugs we need. The cost is covered by the woreda finance office. Let say [RESUPPLYING HUB] needs six hundred thousand birr, the woreda finance office make transfer the amount of money to [RESUPPLYING HUB] account. After we received the drug from [RESUPPLYING HUB], the original model will be provided to finance office.

I: who made this purchase?

R: we, the woreda health office. In Somali region only [district] directly purchase commodities from [RESUPPLYING HUB]. For the other woredas purchasing takes place by the regional health bureau. Before some years the regional health bureau has purchased drugs, but there was lots of drug expiry because they didn't know about the cases burden, then the woreda finance office decided that they should purchase drugs for our woreda. Here is the number of expired drugs which were purchased by the regional health bureau. After we started to purchase drugs no expired drugs available now. When the regional health bureau purchased drugs, there was high expiry of drugs.

I: Do you have enough amount of budget for purchasing supplies?

R: The current budget is insufficient due to the high patient volume at our health center. Previously, there was no community health insurance like the one introduced in 2014 E.C. or 2015 E.C. However, with the implementation of community health insurance in 2016, we have seen a significant increase in client flow. In 2016 E.C., the woreda allocated 1,013,000 birr at the woreda level. We used this allocation to purchase drugs and then sought reimbursement from the health insurance. We consistently register all clients who receive services through the health insurance system and use the reimbursed funds to buy more drugs. However, there are times when we cannot obtain drugs from [RESUPPLYING HUB] and are forced to buy from private sellers, which is much more expensive. For instance, purchasing 4 cartons of drugs from a private seller costs

200,000 birr, while we could get 50 cartons from [RESUPPLYING HUB] for the same amount. In general, after community health insurance started, the drug consumption is high in our health facility.

I: what would be the solution for these issues?

R: the woreda health office should increase the budge and the health insurance also should increase the allocation budget.

I: Do RDF drugs purchased in the name of the health center or the woreda?

R: In the name of the woreda and we stocked the drugs in this store then we give issue Model for the woreda finance office. The woreda health office doesn't have model for RDF. The budget is allocated directly from the woreda finance office that is why we should submit the purchasing model to the woreda finance office. It is also important during auditing process for verification. During audit, we should show how it purchased, when it is purchased and the amount also

I: do the woreda health office has Model?

R: No, they don't have model for RDF, but they have for program only and it is published by the regional finance bureau.

I: How the health posts receive commodities from the store?

R: they/ the health extension workers use ambulance or the woreda car for transportation. If we couldn't get the ambulance, we call to the woreda administration office and request to give us car, then quarterly I provide commodities to the 16 health posts.

I: what are the challenges that hinder to transport drugs from the health center to health posts?

R: the main challenge is transportation problem. Like shortage of vehicles, and also road problem especially in rainy season we couldn't access some health posts. The farthest health post is 35 K.M away from the health center. During the rainy season we can't provide or reach out in this health post, so we should wait until the dry time. There is another option to reach out this health post but it is very distant and it is on the way to [district]... like 160 k.m. it is during rainy season like when there is rain in[regional capital].

I: when is this situation commonly occurs?

R: it happens two times in a year like January, December, and April and …….almost six month in a year. We faced this problem for one health post which is called {health post]. There are two routes to reach out this health post, the one is directly from the [health center] to [health post] which is 35 K.m far, and the second is during rainy season from [health center] to [district] then [

health pos] which is 160 K.M from here. Commonly there is stock in this health post. Even they can’t call using phone because of network problem.

I: what do you suggest to solve this problem?

R: using the alternative routes which is the longest one the other solution what I suggest is constructing bridges for the long term. Always quarterly I go to the health post to provide commodities. After giving trainings for those far health posts better to provide more commodities comparing to the usual one. Provide more months of stock since it is far and difficult to access frequently.

I: probing: How do they request?

R: they request using phone call while the network is functional and they send messages. But the challenge is I don’t verify the balance they do have on hand like beginning balance, consumption and currently on hand. There is network problem for five health posts.

I: so, if the network is challenging how they request?

R: this five health posts have an access to transport, so the health extension workers can come to the health center for request

I: do you use IPLS?

R: We currently use and update a BIN card in hard copy format. Previously, we used a duplicate copy of the BIN card, but it was impractical because it was easily displaced by the wind.

I: How long is the lead time for [RESUPPLYING HUB] to resupply commodities after request?

R: within 15 days [RESUPPLYING HUB] deliver commodities after we request. Our health center is a direct delivery site. There is no any health center other than this one in [district]. So [RESUPPLYING HUB] deliver supplies based on the schedule. Our challenge is to deliver commodities from health center to health posts.

I: How is your way to give drugs for dispensary or other departments?

R: I use first expiry first out method. I inform health care providers working in OPD to prescribe drugs which will be expired within 2 or 3 months. First I should enter drug data on [ELMIS] system then the health care providers request me using IFRR. After approval of this IFRR from CEO, I will use model 22 with three copies, the original one given to the health care provider, the second copy given to woreda finance office and the third copy stay with the pad. After we finish this model 22, letter should be written to get this model again from the woreda finance office. The processes are first the health care provider fill out IFRR, then the health center CEO approves this

IFRR, then after it comes to the store man, I calculate the balance using [ELMIS] system and enter data in the system.

I: is there an agreed schedule for requisition from different departments?

R: yes, we have schedule. Every Friday for dispensary, for MCH every Thursday, laboratory every Monday and they are comfortable for the schedule.

I: do they always keep the schedule for emergency cases?

R: no, for emergency cases I provide drugs or supplies. First, they go to CEO for approval for emergency requests also, without approval I never give them any supplies.

I: do you have any challenge in this processes?

R: no challenge currently, everybody follows the system. The first the challenge was to understand the [ELMIS] system, but now it is okay. Sometimes there is electric cut of but we have a backup system which generator

I: How functional is [ELMIS] system?

R: we are supposed to generate RRF from the [ELMIS] and send to [RESUPPLYING HUB] in the next month. Previously we use the Hard copy to send for [RESUPPLYING HUB].

I: how do you manage Expired supplies?

R: we don't have more expire drugs. But we should list out those expired drugs about Name of the drug, expired date and cost, then there is committee from police, health office and from finance office come and signed on the document shipped with car to the disposable site, which is somewhat far from here.

I: do get supportive supervision from regional health bureau?

R: yes, we got supportive supervision on January, mainly on program, RRF and request. They asked about the BIN card, I show them on the [ELMIS] system, but asked them the hard updated BIN card. They give me feedback orally, and give me on job trainings. They also tell to the CEO about the gaps. There is no written feedback from supervision team.

I: How do you manage the total inventory system? How do you manage overstock or understock?

R: before [ELMIS] system there was over stock and understock. If there is over stock I will inform the CEO of the health center and ask other woreda for transfer. We made transfer for hospital once. And we made exchange of drugs from one woreda. We also make annual inventory on June 25-30. We count all drugs and the cost. We also make inventory quarterly. And finally, we make annual inventory.

I: do you have the document?

R: this is yearly physical inventory…………here is also quarterly inventory document. The document always kept in the store.

I: what about the health posts?

R: I visit each quarter to manage supplies, keeping track of all drugs by recording the amounts collected, dispensed, and remaining. I will also check for expired drugs, counting and listing them on a sheet of paper. Afterward, I'll provide a copy of this list to the head and take photographs for documentation purposes.

I: How health posts especially the nearest request supplies?

R: health posts send reports quarterly. And they request supplies quarterly using white paper because HPMRR is not available in the health posts. Unavailability of standard reporting and requesting format in the health posts are our challenge. By the way these standard formats are not available as a region.

I: did the health extension workers received trainings about Supply chain management system?

R: only they receive on job training about how to fill BIN card, but it needs long time to understand this and to fill out. I only go to the health post quarterly, no frequent travel to the health posts to give continues on job trainings.

I: if all necessary formats are available, do you think they request properly?

R: if we give continuous training they can request on time, but the challenge is the transportation system.

I: How do you use data for decision making?

R: there is QIT and PMT in our health center, but I don't participate in these meeting at health center level. I always participate at woreda level. I am a member of woreda level PMT. We are working on especially on reports like DHIS2, we compare monthly and weekly reports and if we find discrepancies we call to the health post or health centers, and review essential drugs also which 25 item types are there. The store man usually participates on reports. The woreda PMT conduct meeting monthly and rise issues on nutrition, surveillance and MCH

I: do you rise supply chain management issues in PMT meetings?

R: commonly this issues rise in management committee meeting and review gaps on consumption, quantification, health posts, and quality of data BIN card updating

I: what about data issues in related to supply chain management system?

R: the woreda PMT meeting usually focuses on Nutrition, but no problem on supply chain management. If there is stock we list out those drugs and request the health insurance and solve the problem at woreda level. For drug purchasing in case of stock out, I write the request paper and approval from the health center administration, then I request the woreda health office, then we call to [RESUPPLYING HUB].

I: what data do present during PMT meeting at woreda level?

R: I see overstock or understock, identify problems at kebele level or health post, what is the problem are these on data quality, report or what, I list out those problems for PMT meeting. I list out the actual problems like drugs which will expire in near future…

I: what drugs are overstocked currently?

R: no, drugs are overstocked here rather there are emergency drugs which are stocked out currently which are program like ampicillin injection, it is zero balance or stocked out currently. I list outed as emergency order.

I: what are the challenges that hinder the continuous availability of commodities in this facility which is last mile delivery?

R: We need a refrigerator, as well as solutions for electricity outages and a shortage of drug shelves. The laboratory department also requires a refrigerator. Health posts are missing necessary formats, and there are issues with logistics and transportation, such as the lack of a car for training sessions. Additionally, there is a need for improved capacity building, as the current workload is leading to lower quality reports.

I: how do you transport vaccines to the health posts?

R: this is the responsibility of the cold chain person/EPI focal person.

I: thank you very much

R: thank you too!

KII Transcript (22)

**Name of Project:**	SSD
**ID Code**	KII OA 15
**Type of interview**	KII
**Type of informant**	[sex]
**Region**	[region]
**Role of the Interviewee**	[coordinator]
**Years of experience**	
**Name of Transcriber:**	[lecturer]
**Date of Transcription**	August 2024
**Length of interview**	Start time: **2: 11 PM** End time: **3: 46 PM**
**Names of Reviewers:**	

Start of interview

I: Can you describe your role and responsibilities within the WoHO?

R: I am [Logistics and Supply focal person. Eee, our main role at woreda level is availing the service and making the service provided well. There is no service available if the supply is not available. For this we have to strengthen the chain. For the supply chain, there is RRF which is reported from health centers. We will check for correctness and aggregate it and send it to [SUPPLYING HUB] (the current [SUPPLYING HUB]) up to10 (date?). I mean every two months. [SUPPLYING HUB] will check and provide based on availability. There is also IRRF which is filled by the facility and used as an intra-facility. HPMRR which is also used by the health post. They fill and send it every month. The other thing is that we provide supportive supervision. M and E, monitoring and evaluation. We go there every quarter, check it and send it to a zone. We use monthly and quarterly reports to provide continuous support. The problem is from [SUPPLYING HUB] side on availing the supplies. They say it is due to a system change. This time the shortage of family planning supply is high. The is being delayed and they (health facilities?) are requesting TB drugs. They say we bring for four months but what they brought is not enough even for two months. It's now been more than five months since they provided it. However, we are filling and sending our RRF. *(Azan from the background)* although they are not sending the products we are sending the report. Another problem is the road, which is common as a nation. We are using ambulances to prevent service interruptions. There are also places where

the donkey is used. For instance, for “[health post]” donkey is used for transportation both during the winter and summer. We are trying to overcome the problems in such a way.

I: Very good. We will discuss these issues in detail one by one. How do you see facility readiness in reporting and data quality?

R: This is an interesting question. We have health centers which are new and also which can be considered as a model for RRF use and pharmacy service. For remote facilities, there is turnover and new professionals will be assigned. For the new professionals, we will give them orientation on how to fill it. Most of them are training and how to fill it but there is a problem with data. To correct this, we also provide supportive supervision and in-service training. We also have a system called medics. Then fill it out and send it to [SUPPLYING HUB]. Before sending it to [SUPPLYING HUB] it will stay at woreda level for two days. We will check and validate or reject it. If we reject, we call them back and tell them to correct and fill this part. Now it is being corrected by using it.

I: what about the data quality?

R: Some of the data has quality problems. We are addressing this through supervision.

I: what about Coordination with resupplying hubs?

R: The hub is closer to us. When there is a family planning shortage, the woreda go and receive it. Health centers also go and receive it when it is available. We can go and collect anti malaria in less than a month when there is an emergency. Since it is closer to us we have a good relationship with the hub.

I: What about the workforce. For instance, the woreda is very wide, and has many health centers.

R: Yes

I: As you have told us, there is turnover and hiring new professionals. How do you see their capacity?

R: Practically, there are two health centers that have problems. You know what, through this support, not only the store man but anyone who works in dispensary also got the training. In addition to the pharmacy profession other health worker who work in dispensary also got the training. Sometimes the pharmacy professional may not be at the facility and other clinical might work at the dispensary. So they got the training. The facility heads also got the training. They were trained on how to fill RRF, who to fill HPMRR and how to request intra facility. We are trying to reduce the gap in such a way. We cannot say our data has quality.

I: how many health centers are there?

R: nine, there is also the called [health post] which is a comprehensive health post.

I: for how many of them program drugs directly supplied by [SUPPLYING HUB]?

R: it can differ. The season determines.

I: what do you mean?

R: they can reach eight of them if the season is normal. But now it cannot reach even five of them as the road is not good. At this time, it is rainy season and it can only reach Name of woreda and Buyo. They bring it using the big car and drop it here. We then deliver it using the ambulance. We wrote a letter stating where they can reach during the rainy season and we discussed it. [facilities] are being delivered indirectly. We wrote a letter stating four of them can be reached indirectly.

I: what about the health post

R: They are not reaching the health posts.

I: what about the comprehensive one?

R: they will get it from the health center.

I: How many of you are working here as logistic team in the zone?

R: we are two.

I: How is the supervision going? Two of you go for supervision?

R: Supervision will be conducted as a team. Supervision of logistics is difficult. It has no budget. It is only included in the plan. I also asked [partners] to take us with them when they visit health facilities. I asked them not to bring only the feedback and take me with them to visit. The bureau has nothing to arrange for it. When we do supervision as a team, we use checklists where ours is also included. I will check it only when I get a chance of filling that specific checklist. She is taking on 2 responsibilities she received training on APTS for [health facility]. Today she is working there. She is helping them with it and also providing the supplies.

I: do you think 2 people is enough?

R: although we say it is not enough. There is no position in the structure for it as a woreda. The standard is 2. It is only one person in another woreda. It is the only store man in the woreda. We feared the crime of being both the provider and store man.

I: **What are the major barriers that deter continuous availability of health commodities at health facilities and health posts? For example, what about staff commitments in terms of reporting on time?**

R: there is a problem from our side regarding support. But there is no problem with reporting. I will start calling them before 30, and they will reach here before 5. However, [SUPPLYING HUB] is not bringing it. It does ration whether you ask or not. It provides 15,000 for everyone including

for the one which has 50 thousand population. They do not see your data. I don't think they checked it. I have seen it. Last time any provided an equal number of anti-malarias for [health facility} which has 50 thousand population and geta where there is no malaria. Sometimes they also bring drugs that are about to expire. It is difficult. There is no problem to reach the health centers once they are here. The problem is that you cannot get what you have requested. Even, we have stopped talking about [SUPPLYING HUB].

I: You have told me about the orientation and on-the-job training for new staff. Is induction training provided to new staff either by calling them here or by going to the facility on RRF, IRRF, bincard?

R: When a new staff member is recruited, he will be informed of what he is supposed to do and the job description by dispensary head or facility head. This is a must, as it can affect the work.

I: you mainly get drugs and other supplies through [SUPPLYING HUB], is there any support related to the budget to purchase supplies that [SUPPLYING HUB] is not able to provide?

R: this is good question. [SUPPLYING HUB] is responsible for program drugs. Program drugs are not sold and they are not budgeted. It is free. [SUPPLYING HUB] will sell other drugs. For that, the health center has health care finances. They plan it based on health care finances. I am asking for it right now. They will plan this much for drugs, this much for medical equipment, and bring it here. We support them with 300,000. For that they will bring to us indicating what they have planned to buy. We then take it to [SUPPLYING HUB] and tell them the facility requested these commodities. [SUPPLYING HUB] will provide available things based on that. This is why we are complaining about [SUPPLYING HUB]. I asked for 150 items, but only 20 items were delivered. Most of it is gloves. If it is not available, [SUPPLYING HUB] will write stockout, and the facility will buy it from an external supplier. There is also plumpy nut which is provided by zone. We request and bring it based on the malnourished population. We also get anti-malaria from them. We bring emergency drugs and items like Vitamin A from the zone.

I: what about the availability of different tools and formats? It could be RRF or HPMRR

R: initially, [SUPPLYING HUB] has been sending the RRF. HPMRR has also sent HPMRR once. Once it stops, it is decided that facility must take the ownership. So, they are printing and using both RRF and HPMRR. Lilu and Name of woreda are now printing and using it.

I: are all of them printing and using it? Is there any shortage?

R: it is a principle. Additionally, they lose money. If they don't do it, [PARTNERS] is not going to pay them. Both the health center and health post will not get the money if the HPMRR and RRF are not filled. Everything is related to money. The facility will be included in the plan. They will include what they are going to print.

I: Can you describe the current quantification and procurement process for RMNCH commodities

R: the health center does two types of quantification. They will give us what will be included in health care financing at its price. We send that form to the health center. Health centers will send back indicating what they need. We will send that to [SUPPLYING HUB]. We request it every year but the problem is that less than 50% is provided. It is being requested twice a year. That means every six months.

I: What challenges are there, for instance, forecasting?

R: The problem with forecasting is they will not provide you with what they need on time but at the end of the year. This indicates the presence of a problem with the forecasting. That is why we ask them their plan on time. there is problem with the plan. Such a problem happens when the head of the health center is new.

I: Why did this happen? Is it due to skill or lack of commitment?

R: the first one is associated with capacity. It may depend on not knowing the population they serve. As the issue of the health not stable, some health problems may happen. For example, this

year, there was measles outbreak in Lilo. So it is unpredictable. That means there is a problem of budgeting for emergency preparedness.

I: there is a problem of emergency contingency planning

R: there is such a problem as a woreda. It is not common to plan for health emergencies. But when there is a problem we plan for it and the money will be provided through [district]. We get 100,000 from them when measle outbreak happened.

I: What about other issues other than emergencies? In terms requesting after analyzing the data, identifying their population and checking their last month consumption.

R: Most of the time their plan is related to services. There is no problem with planning. The management body from here and heads from the facilities do planning at [zone]. They just plan the money, not the details of each drug. We will call and ask them what they plan to purchase. The plan is done roughly most of the time.

I: What do facilities forecasting accuracy look like if you have checked it?

R: That's good. It is around 70. 70 percent. (*long pause*)

I: so, what is your role in quantification?

R: we know all the services they provide. If they ask for in appropriate thing, we call and ask why they planned or asked for that product. Then we discuss on it and reduce what is supposed to be reduced or add what is supposed to be more. There is vein analysis, there is DTC committee at the health center. Most of the time there is no problem if that DTC checks it. But sometimes, it is problematic when only the pharmacy professional fill and bring it without DTC committee approval. Our role is to strengthen that activity.

I: How many of the facilities have functional DTC?

R: All of them. But it might not be adequate in terms of having regular meetings.

I: Is there a skill gap in quantification skills among the professionals?

R: the problem we are facing in terms of using the tools. I have a problem filling the softcopy including using the computer. This problem is in all of the health centers. (*Long pause*)

I: you know more from experience. Can you tell us what we can do differently for strengthening the quantification. It can be related to tool and capacity building in terms of improving accuracy of quantification.

R: It is better if they plan separately for the products, they purchase themselves. Most health centers are not familiar with the tool. We face problem with the new facilities. They say what is quantification. There are facilities that send us lists of what they need just on paper. It is better if they sit down and discuss it based on Vein analysis and DTC committee before deciding the budget. It is better if they decide its importance for the health facility when the vein analysis is done. Otherwise, important things are missed and not important things become excess. This shows underutilization of vein analysis. They don't have review meetings or training. We are planning it now. It asks about it on M & E. my collogues once got training on the at Metu. She is not able to provide training to the health centers due to budget shortages. It will be difficult to make them stay here for three days without payment. So, we have planned for this year.

I: How does the transportation and distribution system for RMNCH commodities, including vaccines, function across Woreda and health facilities?

R: If we start from the hub, their car is a big one. It cannot go to [health facility] neither in winter nor summer. It is better if they use another kind of car when they plan to come such place. When it comes to our situation, the ambulance is not currently available. RDF drug which came for Geta is still here. Drugs which cost 200,000 birr and come from {region} are with me now. I have delivered to some facilities and not able to reach others due to transportation.

I: For how long is it here?

R: It came here in July. For [health facility], it was transported using donkey and motorbike. We

were trying to deliver it using the ambulance, but it was damaged on its way. The woreda needs a car. The other problem is road and it can be solved through discussion of the community and government. There is one health transformation car. It left now to deliver drugs to health facilities.

I: is it dedicate only for the drugs?

R: No, its for overall woreda activities. An ambulance is dedicated for mothers, but we are using them for transporting drugs also used. We don't have a car dedicated for transporting drugs.

I: what about the store capacity, including the one you use here and at the health facility?

R: it is better if we say there is no store here. We don't have a store. The one we are using is not safe, "Yafesal". We keep drugs in it only for a week or two weeks. Woreda is considering building it, but there is a budget shortage. Thanks to [PARTNERS], it is very good at health centers. You can go and check it. The one in the health center is the best.

I: is it enough if everything is facilitated for [SUPPLYING HUB]?

R: yes, it is enough [SUPPLYING HUB]. If [SUPPLYING HUB] is able to deliver, the health center has a place to store everything. The problem is with delivering it. If they reach there, the center has enough space and can arrange where to store it. Health centers are building blocks through support from [PARTNERS]. For instance, if they want to provide family planning services, there is no supply. Then both the health center and the community face problems.

I: You have told us as you use different means to deliver products to those health facilities that are not accessible. Is there any other alternative means of transportation to get there?

R: we use motor place where not possible for the car. The other thing is using donkey.

I: from where do you get the motorbike.

R: the health centers rent it. What other options do they have?

I**: what about transportation to the health posts?**

R: the health post themselves go and collect it. Many products are not kept there at health posts. They take it from health center, provide the services. There is no store there. Only emergency products were kept there. Vaccines may be kept in places where there is a fridge. If there is no

fridge, they collect the product, provide service on schedule and return.

I: how they take the drugs?

R: they fill the HPMRR and bring it monthly. The store man checks it and provide them. The health centers have motors and they use that one. Sometimes when someone goes for support, it will be sent to that person.

I: Do the health posts have access to transportation like car, Bajaj or motor?

R: You pay for transportation. If you want to go to Saxema you will pay for it. Only those who are near the town travel daily and they have access to transportation.

I: What are the barriers and opportunities to strengthen health post-resupply?

R: Previously there was IMNCI. The supplies have been provided through support from NGOs. Now it was stopped. The health center has supposed to buy it but it is not available now. Service is reduced in this area. Under five services are recommended to be provided for free, whether they have insurance or not. This supply is reduced at the health post. [SUPPLYING HUB] is also not bringing the supplies. Other NGO had been providing it.

I: is the vaccine provided by you?

R: yes, we have a small store specifically used for vaccines. It come directly by [SUPPLYING HUB]. [SUPPLYING HUB] drop in for Name of woreda and Kiyu. They will drop it here for all other facilities.

I: After how long after [SUPPLYING HUB] brought do the facilities collect from here?

R: it is not more than a week. It will reach there fast by any means. They come here and collect it. Or we can deliver it using our transformation car. We also use ambulances.

I: is it similar to a program drug?

R: yes, it is similar. They bring a letter to program drugs. Based on the letter they will collect the drugs after filling it on a model. As we collect it from [SUPPLYING HUB] using the model, I will provide it using the model *(laughter).* If they come when [SUPPLYING HUB] is distributing it, they will immediately collect it.

I: who come with the model? Store man?

R: yes, the store man. This happens when it is not possible to deliver directly. Most of the time the drop here and the facilities come and collect it within a week. SCTU will remain here as model 19 is with me.

I: on average, after how long does [SUPPLYING HUB] provide you following RRF you submitted.

R: in 2016, they bring drugs only twice. Once they say we are starting a new system. They brought the product and said the product is for four months. But we faced shortage in a month. We requested up to 1000, but they brought 400 for each health center. Then they disappeared for four months. Now they are saying we have started quantification. Now we are waiting for them. I called yesterday and the day before yesterday to ([SUPPLYING HUB] hub). He told me it is not available. The system itself is getting delayed and the service is being interrupted.

I: In general, how do you evaluate the capacity of [SUPPLYING HUB] with regard to direct delivery?

R: previously [SUPPLYING HUB] was good. Our complaint is that it is not bringing what we have requested. We have been getting 4 0 to 50% of what we have requested. Now we are going there and collecting it. Zones have started distributing the anti-malaria drugs collected from [SUPPLYING HUB] as they stopped direct delivery.

I: What do you do with RRF? Can you just compile it and send it?

R: We checked both the hard copy and soft copy. They will correct it if there is a problem with the report. If there is no problem we can send one copy.

I: So, you do not aggregate it.

R: Yes, we will send it as it is. [SUPPLYING HUB] will bring the products in their name for both direct and indirect. They will tag as direct and indirect.

I: How can this transportation and distribution have done differently?

R: we have to ask using accurate data. When you ask for supplies, you will ask based on population size. If everyone works according to the system, it will be corrected. Science is appropriate. The

problem is you are not getting what you have asked according to the science.

I: Is there any alternative means of transportation that you can recommend?

R: there is no alternative other than what was mentioned earlier. Cars can reach many places if there is no rain. Motorbikes can be used during rainy season. The donkey is being used for transportation in areas where it is not accessible for motorbikes. For the drugs which are purchased, they can buy the one which has a longer expiry date before the rain starts. They can purchase and store enough drugs by checking its expiry date a head of rainy season. This can be alternative.

I: Let talk about inventory management and LMIS systems? What are the main implementation challenges of IPLS in health facilities?

R: regarding the IPLS, [PARTNERS] has provided training to all health centers. Inventory is done twice in a year at health center. Health posts send it to the health center. They will do inventory of medical supplies and medicine and send it. For example, that they will send it to us in June of this year. We will record and say this amount of medicine and medical supplies will be transferred to be used in 2017. We have this data. The problem is they may face the problem with using the formats if they are new. In this situation we are providing information on how it is filled.

I: is there a situation when an emergency order happens? How do you manage those emergency orders?

R: emergency order happens when the requested order is not purchased on time. it also happens when the insurance money is not paid on time. but when it is emergency in one, it is available in other facilities in the woreda. You will see it in the softcopy of the [Name of an eLMIS}. If anti malaria is not available in Dabo, I will see which facility is available in excess. We are managing it this way. Now everyone is feeding the data. Whether it is the one they get from [SUPPLYING HUB] or RDF drugs, they feed it into mobile. So, I will see the health center with shortage. If they daily update both the softcopy and hardcopy, it is good. For example, buyo have received from Name of woreda twice in this way.

I: what mechanism do this facility used to share this method?

R: when they receive it in batch, it says the product has been transferred to this facility. It write “lose” for the facility which provided it, and it adds on to the other facility. The hospital sometimes

receives it. Once, there was many fluids in [name of health facility], but in hospital many people were admitted due to measles and other diseases, we then transferred to here. The facility will also receive other drugs at any other time. there is also a transfer form.

I: so, there is transfer SOPs?

R: Yes, it is available.

I: do you receive a copy of the transfer?

R: Yes. Most of the time, anti-malaria is available in satema, and hospitals go and receive it and they will also come and take the drug that is not available in their facility from the hospital.

I: how do they know the availability of those drugs?

R: they discuss among each other.

I: ok. They talk to each other and if it is available they write a letter. You will receive a copy of the letter and confirm the transfer.

R: yes

I: as far as I understand, the software you told us shows what is available in certain facilities and that is not available in other facility. To make that think robust, do the facilities, pharmacy head and store man fill it continuously?

R: that is the one that needs follow up. That is why I always call and tell them to make their phone active. They buy drugs and give us an invoice copy of what was purchased. Then I check whether the drugs are filled based on the invoice. If it is not filled a call and inform them to fill it.

I: you mean including for the RDF?

R: Yes, we are using it like this.

I: are they also updating the drugs they have used?

R: it is a must. For example, let's say he provided to the health post, if he is not filling it as it out is provided to the health post, he will be asked where about of that drug. He will fill "lose" on out the software. It is them who fill the forms including the RRF. We cannot fill it. I can only make

phone calls. I cannot edit or manipulate it. For instance, if they want to request for a purchase of drugs from [SUPPLYING HUB], the facility head will approve it. It works like that.

I: how many health centers are using this system?

R: all of them are using it.

I: so, you can visualize nine of the facilities. can it generate a report? RRF report?

R: Yes

I: Sure?

R: Yes. It generates, I will approve it and then they will print it out and send it to [SUPPLYING HUB]. If I cannot approve it in two days, it will pass and they will print it.

I: you approve their RRF?

R: Yes

I: who else approves it? Facility head?

R: facility head woreda approve it. But health care finance is not approved by Woreda. It is approved by the facility head and [SUPPLYING HUB]. Facility heads should approve it as he decides on the money.

I: what is the role of [SUPPLYING HUB] in health care finance?

R: [SUPPLYING HUB] role is, it sees what they have requested, and inform the products that are not available.

I: so, they take the hardcopy to there.

R: they send the hard copy, but [SUPPLYING HUB] can also see what they have requested online. EOSA was also trained on this.

I: is [SUPPLYING HUB] using (another eLMIS)?

R: Yes. They were trained on (eLMIS 1). They also use this one.

I: can you see on (eLMIS 2) the drug that [SUPPLYING HUB] has?

R: no I cannot. The health facility can see it.

I: so, medics shows the [SUPPLYING HUB] data to the health facilities.

R; yes. They check it and make an order.

I: what challenges are there with its implementation?

R: the challenge associated with the network. It is not working in rural areas. Now, from Geta bake, he is telling me he is travelling forty minutes to open it. The network is challenging. Even it cannot open here sometimes.

I: is there any other challenge?

R: it is money to buy air time used for the internet. They complain it uses more data. They just provided the phone (*laugh)*

I: Does the emergency order happen frequently?

R: no, it is not frequent.

I: what about wastage? Especially program drugs.

R: The current problem is not wastage, it's shortage. Currently, wastage is reduced highly both for program and RDF. Everyone is a member of insurance. If one person gets sick every member of the family comes for treatment. It may be damaged when it is transported using donkey.

I: what do eLMIS data accuracy look like?

R: That's what you enter. If they fill the correct data the out but it will be correct. If we take Name of (woreda) wastage rate, it shows 0.8%. When you open it, it shows the wastage rate, it shows the percentage of availability. When we talk about accuracy, it is like I have said. When entering accurate data, it shows accurate output. There is problem of adding, and subtracting the items on time. this will be addressed overtime.

I: how do you evaluate the inventory management system at woreda and facility level?

R: the inventory is good. It helps to identify functional equipment and that needs to be maintained. The inventory is being done by the committee. The DTC committee will check what equipment

and drugs are available at OPD or elsewhere. The problem here is you will be told to register it and the head will sign it and send it. We have also discussed this during our previous evaluation. Sometimes the head or staff may not know what is available in store. When inventory is done many equipment can be found.

I: Is stock out analysis done?

R: at woreda level?

I: It can be woreda or facility level?

R: yes, it is done. But it is not complete. It is done sometimes. About five facilities are doing well, but it is not enough.

I: you have told us about stock transfer and exchange. What challenges are there with the stock exchange?

R: it is good now. Previously the hospitals and health centers had been exchanging without informing the bureau. The bureau has to know what item is outside of the health center and what item the hospital has received, and then what they have received from the hospital. If one health center has to item and the other one has nothing, they will provide it the one which has nothing. The facility will replace that item another time. We are working to prevent service interruptions as they are serving the same population.

I: what has been done to prevent over stock?

R: Overstock exists. Once there is overstock, exchange will be done. The other method is to check it during procurement. So, quantification and other activities are important at this point. We can also check and reduce it. For example, we write a letter to [SUPPLYING HUB] for most of them, so we discuss it before requesting. If they say DTC decided it, we will do nothing, but we will check everything.

I: So the health extension works have no store?

R: they also take it using the model. There is no one who can take it from them using the model.

They will provide it after recording it on the register.

I: How about the internal facility report? Between the store and the units? How about commitment of the staff in terms of sticking to the schedule of using IFRR.

R: Using IFRR has been challenging for a long time. but now, it is a must. After taking the training, it is not possible to take drugs without IFRR. The units collect drugs every two weeks from the store after filling the IFRR. However, it comes be weekly based on the service provided by the unit. The practice is already there.

I: what support do you provide to the facilities on this issue?

R: When we go for supportive supervision, we check how they fill out the form and request it. We give them an advice if they are requesting without filling out the form

I: what about HPMRR? Did you visit the health posts?

R: yes, it is a must. We visit them. They were informed to do it in three copies, one copy was reported to us. This will help us to do analysis on what products they have received and utilized. They do the report in three copies. One remains with them, one for the health center and one copy comes to the woreda. When one RRF comes every two months, two HPMRR must come. We will check what they have requested and what they have received.

I: what have learned from this? I never heard that HPMRR is reported to woreda. Is it best practice? What gaps have you observed?

R: there is gap on data quality. What they do is (*laugh*), the format stays with him. When they come (for resupply), he fills it and give them one copy. The format will be kept with him. This is not the correct process. The hard copy pad would have been at lower level with them and the filled copy should be provided to him. We told them to correct this. They are doing this because of shortage of the format. We have been commenting on this. The thing that we need to know is that there is no enough HPMRR format. They have to fill it at health post level just like the older IFRR. This is the gap that I observed. The other problem is they are not getting enough supply as the health center is also not getting enough. They can only provide it if they get it from [SUPPLYING

HUB]. Since [SUPPLYING HUB] is not availing supplies, it is difficult.

I: earlier you said we have one comprehensive health post. When was it started functioning?

R: three years ago.

I: Have experienced any challenges? Do you know why I am asking you this? By default health post get supply from health centers. Is there any new thing that we can learn?

R: Even though I was there when it first opened. I stayed there for two months to make it function properly. The problem is, it has no budget. It has no budget for purchasing drugs. It provides services and the income is for that health center. But it is providing services to people who come from different area including from (name of woreda). About 5 to 6 kebeles use that health post. But it has no budget. How can the drugs be available? It waits the supply from the health center. The health center is not getting enough supply as it only receives the maximum supply provided to the health center. It would have been recognized as a better health center. The community was happy with it when it started providing services. Now, it is reduced as the service is getting reduced. Once we begged and get 100,000 from woreda. What can 100,000 buy this time? Most health care services are provided for free. Maternal service is free. Child care is free. Such things cause the facility to fail to avail supplies. Most of the drugs used to treat children are bought but, they will be informed to provide treatment for free. So, the health become weak.

I: How is supply chain data generated, shared, and utilized for decision-making, including here and health facilities?

R: obviously, data is needed for decision making. We use it to request for the supply we have finished. Now, through the support of [PARTNERS], DTC is functional. Essential drugs that help stop the service from stopping are always available. To provide the service continuously, using the data is a must. The data is found from the provided services on registry and RRF. Once they enter the data from RRF and HPMRR, the quantification will be done. Based on the quantification, we will bring the one which is received from zone and the one which is supposed purchased will be purchased accordingly. Although the data is not accurate, it is important.

I: For instance, is there a situation in which you aggregate and analyze the data that come from facility and present it to woreda management?

R: Yes, we present it. You ask for things based on the data. It is also used for decision making. Health centers also request supplies based on their data. Health center and the health post s are providing services. They are getting the supplies based on the supply they provide.

I: what about PMT?

R: PMT exists in all place. it is functional at woreda level. They are responsible for reviewing data at the facility level.

I: what is the role of pharmacy?

R: One pharmacy professional is working at the facility level and he is a member. Here, the head of our core process take part. It is called health facility support core process. We provide the data to him and present it. He also knows what we are doing.

I: Can you provide him with this data?

R: Definitely. He will also check it and discuss with us on the data. He was also given training.

I: What training?

R: On the supply chain. The core process is mainly about supply.

I: how often the facility send M and E reports?

R: it was quarterly. But starting this month they are sending it monthly.

I: what about DHIS2, are they sending it?

R: Yes.

I: did you review it?

R: yes, we had been reviewing it this week.

I: so, is there a platform you use to review the M and E, and DHIS2 reports?

R: PMT is working very well. Not only PMT, I also take part in the meeting and help them identify the missing data. Checking the report is a must. We will check the report and identify what was done and what is not done.

I**: What about giving feedback to the facility?**

R: Yes, although it is not separate feedback, we give feedback as a core process indicating the existing gap and what has to be improved regarding the pharmacy.

I: what support does the woreda need. It can be supervision or any other support. It can be a capacity building that can facilitate supply chain management. What support do you need?

R: To give supportive supervision at a lower level, it needs support.

I: what kind of support? Is that a budget?

R: yes, it is budget support. Manpower is enough. She (his colleague) who gets training on M and E. so it needs training. We don't have enough trained personnel. We are doing it from our experience. However, training is a must to know any amendments or changes. Eee… there might be things that are updated. Training is needed at both woreda and health facilities. Training on quantification is very crucial for health centers. I never get training on quantification, but I have been working on asking others who to do it. She is also not trained on it. I also never got training on M and E. I am doing it after reading the softcopy. It is also needs motorbike to go to the lower level. The current transport service is well known. Other than (woreda 1) and (woreda 2), others are very difficult to reach.

I**: do you have a regular platform where you can meet with zones and regions?**

R: We don’t have it

I: what about with [SUPPLYING HUB]?

R: we don’t have. We don’t have the budget to bring the pharmacists here to discuss.

I: What support is provided to the health facility to make them use this supply chain data?

R: it is better if one pad of HPMRR is availed at health posts. We also need a budget to call the

pharmacist every quarter for discussion. We have to go there, check the reports and then call them here and discuss on the gaps and progress from the previous quarter. To do this it need resource.

I: Do you have mobile airtime for calling the facility?

R: No. I use it from my pocket. I use my money for internet to check the data online. It is the same for those at the lower level.

I: What are the most successful examples of best practices in the supply chain? Is there anything that can be scaled? Including last mile delivery and stockout prevention.

R: one best practice is empowering the health center. For instance, Name of woreda health center has its own model pharmacy and enough pharmacists. Such things must be implemented in all health centers. Unless the health centers are independent and generate their own income, it is difficult for them to avail every drug. Name of woreda Health Center is better in terms availing supplies. On the other hand, program drugs are not available. The products are available in the market.

I: how many health centers opened model pharmacy?

R: for now, it is only Name of woreda health center. (name of health center) will open it in 2017.

I: we are here to learn how we can help the supplies reach out lower level. You are expert on that area. What should be done to make the drugs reach the right place time?

R: To make the facilities work hard to avail and provide service to the community. It can provide support and motivate them.

I: how can motivation be done?

R: It can be supportive supervision or training. It can also to avail the supplies they requested. For example, if the community goes to the health post and she tells them to go to the health center, he

cannot hear her when she teaches about the prevention method another day. So, it needs to avail drugs after doing the inventory for the drugs. Eee … there are basic things that are provided at the health post level. The health centers also started oxygen service Name of woreda, (name of woreda) and(Nearby woreda) provide the service. They need to have training and supportive supervision. Therefore, it is better if basic drugs reach a health post. The reporting format requires support at the health center. They are being informed to print everything, including HMIS formats, the cost of printing is becoming more than drugs nowadays. Things started with the support of NGOs, it left. Health centers were then informed to print and use the format. The printing cost became more than that of the drug. This is needed support. Both [SUPPLYING HUB] and zone should also work on this. There is no product, no program. TB program exists only when the products are available. The system and the supply are not linked. The pharmacist is carrying the burden. It is very challenging, especially at woreda level. When you ask for a car to bring anti malaria drugs, the car is not available. Program owners should share their responsibilities and work with commitment. We have asked for TB drugs from [SUPPLYING HUB] and they said they are not available. You cannot find it in the market. Why does the Zone keep silent when such problem happens. It cannot be effective only by pharmacy unless everyone is committed.

I: thank you for the information.

End of interview

Transcript (23)

**Name of Project:**	SSD
**ID Code**	KII-OA -19
**Type of interview**	KII

**Type of informant**	Male
**Region**	
**Role of the Interviewee**	Logistics officer
**Years of experience**	14
**Name of Transcriber:**	(University Lecturer)
**Date of Transcription**	21th August 2024
**Length of interview**	Start time: **9: 05 AM** End time: **11: 05 AM**
**Names of Reviewers:**	

Start of interview

I: Can you describe your role and responsibilities within the health supply chain system?

R: I have worked for more than 14 years with work experience including pharmacy supply chain and pharmacy services starting from the facility to here in [region] health bureau. Now I am working as a supply chain management expert in [region] health bureau.

I: How do you perceive the overall effectiveness of the supply chain management system?

R: To talk about supply chain management status in [region] region, there is one supplier as a country which is called [SUPPLYING HUB]. It works to program drug procurement, distribution, LMIS activities in government structures starting from health posts. Our facilities request for drugs, especially program drugs, then the suppliers will resupply the requested drugs according to the set schedule. We have designed inventory control system as a country and [region] region is also governed by that system. The RDF or budget drugs are not governed by this. That means the inventory control system is not like the program during that time is supplied based on the preset schedule. So, there is no strong supply chain management system for budget drugs. More or less, we have a system called IPLS that we use to manage our supply chain system in the region.

I: what about timely delivery of commodities, facility readiness in reporting and data quality?

R: As I have said earlier, the inventory control system is scheduled and they have to provide the report according to that schedule. However, we know we have pharmacy workforce capacity, competence and skill problems both at region and country level. Therefore, facility level data

quality, data generation and use has not reached maturity level. Due to these reasons, there are interruptions both for program drugs and RDF or budget drugs. There is inadequate supply at the facility level. Although there are supplies from supplier, drugs are not reaching the facility due to data quality including not reporting timely. This is only applicable for health facilities that are accessible. Our health facilities that are not accessible have the worst problems. So, the problem is inaccessibility and those which are accessible have data quality problems. Those inaccessible health facilities face more health commodities shortages. In addition to that there are security problems. Due to these reasons, it will be difficult to say the commodities are reaching health facility timely.

I: Can you tell us more about the challenges for continuous availability of health commodities at health facilities and health posts?

R: According to inventory control system, it is assumed the facility has the drugs that can be used for four months. To have this, the facility needs to have enough storage. This is not adequate for all facilities in the country. So, it is one of the problems. At the health post level, if I start with its design, there is no storage. The health facility faces problem to store to the commodities according to the inventory control system. The other is the transportation problem. This means as our suppliers face problem to reach all health facilities, our facilities are forced to collect it themselves. At this time, they face problem as there are not enough vehicles. Our facilities do not have specific vehicles that can be used for transportation of commodities. Due to this we also face shortage and interruptions.

I: what about workforce? what challenges do we have?

R: I have mentioned it earlier. There is shortage of pharmacy professionals. We don’t have adequate number of pharmacy professionals in our health facilities. In [region], it is less than 50% according the criteria set by the civil service. It says three pharmacies professional if the facility is B type and five, if the facility is A type. However, it is not fulfilled for many facilities. Secondly, there is a competence problem from the pharmacy professional side. This can be related to capacity building. Most of the time they work based on what they learned at university. They are not getting

enough on-job training. Supportive supervision and mentorship are also not enough. There is a gap in this area as compared to other health professionals. Many things remain in terms of improving supply chain competence and skill. We need to work on providing training and supportive supervision.

I: Can you tell us about performance monitoring and recognition mechanisms?

R: I have been working in this region, there has been no performance monitoring and recognition mechanism for the past 5 or 6 years. Everything is the same for those who are working better or not. This can also be a barrier for performance improvement. A person who works the improvement of supply chain must get recognition. There is no such experience so far. We have planned to do it in the future. The region will work on it at least for those who are at zonal, woreda and facility level. It can be education sponsorship or making transfers from facility to woreda office or from woreda to zone, or from zone to region based on performance. I cannot say I have done anything in this area so far.

I: Can we say staff commitment can be challenging for the availability of commodities? for example, timely request, proper recording, data use for decision making?

R: regarding the staff commitment, we can say it is in good status for some areas. But in some areas, there is low staff commitment which leads to poor data quality, so they face interruption of supplies. But in some areas like (name of a zone -1), [Name of a zone -2], [Name of a zone 3], [Name of a zone 4], the pharmacy workforce is so committed that, they have better data quality, then they have better supply chain management and better communication with the supplier. Since the supply chain and data quality have connections, they have better supplies. If the data quality is good, [SUPPLYING HUB] will not face challenges. For example, if we take [Name of zone 1] and [Name of zone -3), they properly do refill analysis and send it to [SUPPLYING HUB]. [SUPPLYING HUB] will deliver the drugs it has at hand on time without a problem. On the other hand, in areas where there is a security problem, they may not be committed as they get less motivated. So, there are zones, woredas and facilities that are doing better, while there are zones, woredas and facilities that we have to work on.

I: what about management support?

R: I can confidently say there is good management support in [region] region. Even in 2016 E.C. we got good budget and have been working on capacity building as our gap is in that area. As I have said earlier, there is a capacity gap among the pharmacy workforce. To improve supply chain management performance and activity, we have provided training for facility, woreda and zone level workforce. This is because the regional management has provided us with enough budget. We have been providing supportive supervision in collaboration with partners. Integrative supportive supervision was given to program drugs like HIV and malaria. However, there are problems in zone and woreda level management. They have no budget allocated for this purpose specifically for supply chain and pharmacy services. Regional management cascaded integrated supportive supervision and review meetings for supply chain and pharmacy services. However, it is not implemented practically. So, supply chain management support is better at region level but not good enough at zones woredas, towns and facility level. Pharmacy workforce was not included in carrying out supportive supervision. They tell us they face budget shortage to visit health facilities, even once a year. In the [region] region, we have allocated a budget and undertook supportive supervision for each zone and town. Commitment at region level is better. There is a management support gap at the zone, woreda, and facility level. [SUPPLYING HUB] provides the program drugs, there is a sense of considering it as the responsibility of others. There is a challenge with accepting and approving the RRF data that is generated. Failing to review the generated data by the performance monitoring team for its quality is a management problem. So, one of the activities we are going to do this year is to make the management support the supply chain management and use the data for decision.

I: can you say something about induction and skill transfer?

R: I remember we conducted it a year ago. The document was developed by the ministry and we adopted it and transferred to zones and woredas. However, their practice is not good enough. A lot of things are still remaining. For instance, some activities become new for them when there is turnover. But in some areas, especially those I have mentioned earlier, they are inducing and some

of them are also doing skill transfer. Some of them will be assigned to activities without getting orientation and this will create interruptions and affect the data quality. I want to say it was conducted at region level and only a few of them have practiced it. The implementation remains at the lower level.

I: what is the main reason for this?

R: It is commitment issue. As a region we called the responsible person, showed and discussed it with them. We discussed in detail with zones and town administrations. Then they are responsible to discuss with woredas and facilities, work on staff induction and share it with staff. The gap was in cascading what is done in the region to the lower level. As we cannot reach all [xxxx] health centers and [xxx] hospitals. The practice is not as expected. So, when a new professional is recruited, we need to provide orientation and training.

I: What do you recommend to be done in that area to improve supply chain management: What should be done to fill those gaps?

R: Good. We have manuals and formats like LMIS formats. First, it is better if we work on capacity building activity especially at woreda and facility level. There is a huge gap at woreda level. Secondly, making the workforce there familiar with our formats. It can be softcopy or hardcopy. We have learned that we develop SOPs and manuals, and then share them zones and towns, but they are not reaching woredas and facilities. We are distributing the updated LMIS formats to zones and towns, but our facilities are using the older LMIS formats. This is due to a communication gap and we have to work on the communication area. I hope it will have better outcome if we work on supportive supervision and communication activities by using the existing system.

I: Can you describe the current quantification and procurement process for RMNCH commodities (through program and RDF schemes)?

R: quantification of the program drug is done at the national level. At facility level, as I have said earlier, they do inventory control and report it every two months, then collect program drugs. So, they do not quantify. For the future it's better to consider it. We have also given a comment on it. It is better if the quantification for both the program and budgeted drug starts from a lower level. We are not doing quantification for program drugs at region and facility level. this is being done at the national level based on HMIS data. Our role is to collect RRF data report. Quantification of budgeted drugs have now started. Training have been provided at the national level. In our region, the training on the quantification of budgeted drugs is provided to all hospitals. There are facilities that has done quantification exercises and reported them. However, the challenge is the what they forecasted and the budget has big difference. Hospitals have experiences of doing quantification. But there is budget shortage to do procurement according to the supply plan. Overall, there is budget drugs quantification practice at the hospital level. When we talk about health centers, previously [SUPPLYING HUB] go and help them do the quantification which is not functional at this time. They didn't receive quantification exercise training. I hope in the future they will do quantification just like a hospital and get the budget. The current practice at the health center is, the woreda do the quantification for each health center once a year, then send it to us. Then based on that, the region will sign an agreement of 300,000 Birr for each health center with [SUPPLYING HUB]. Based on that [SUPPLYING HUB] will buy and distribute it. This is what is being practiced. It is questionable how 300,000 birr is enough for one health center. So, the procurement and quantification practices are different for hospitals and health centers. Hospitals do their own quantification and purchase it themselves.

I: What are the main challenges encountered? Including RMNCH commodities forecasting accuracy?

R: One of the challenges for RMNCH and other program commodities is … quantification is not done at a lower level. It is done at the national level and facilities request it to collect it. It is better if the facilities do the quantification of RMNCH products. Then, the facility informs the supply they need. Because of that, there is a capacity gap to quantify correctly. It is better if the privilege of quantification is given to hospitals and health centers. Training should be provided to improve their capacity of quantification exercise. Secondly, we also face challenges to get data that we need for the quantification. It is better if support is given at the facility level on these issues. Supportive

supervision on recording reporting of the data that we need for quantification. There is also a big problem with data quality. There is no privilege to quantify it. You will only recieve the products on request in this country. You cannot do quantification and ask for the supply of RMNCH according the current practice. So, it is better if to the quantification is done at lower level, but the capacity building is provided to them to do the quantification.

I: You have told as some point about the transportation. How is the transportation and distribution system for RMNCH commodities

R: ehhh … it is just like other program drugs since the RMNCH drugs are also transported with others in integrations. The storage is also in integration. So, any distribution and transportation problems that affect other drugs also effect it. Earlier we had said there was transportation problem. This is due to vehicle shortage at health facility, due to infrastructure problem, due to presence of man power problem. The other is budget shortages as there is no management support. If the supplier is not delivering the supplies on time, the facility needs a vehicle go and collect the commodities from suppliers. Eee it also needs fuel, it needs manpower, so there is a gap in these points. This means it affects RMNCH product delivery. Data quality is one factor for the distribution. If there is no quality data, some unnecessary products will be distributed to somewhere. So, distribution faces some problems. The factors that I have mentioned earlier can affect the distribution and transportation of RMNCH products.

I: What is the capacity of [SUPPLYING HUB] to deliver these products?

R: Okay. I have raised the problem from our facilities, woredas and zones side. The problem for the supplier side (*people talking from the background*), eee … there are problems, … there are barriers that they raise to delivering it on time. That is, the first unavailability of enough vehicles. Eee … secondly, it is ours. It our facilities are not sending the report timely. They raise the problem of data quality. On this area what we have learned is; supply chain needs immediate response. There is a problem of delivering products from the supplier side, while there is vehicles, drugs and reports that have quality. It might be due to system problem but, they do not give an immediate response. So, it creates interruption of RMNCH products. Secondly, eee … [SUPPLYING HUB] must respond to emergency delivery, according to IPLS. The one I have raised earlier is about the

routine one. If the report is submitted every two months, [SUPPLYING HUB] must deliver the drugs it has at hand within two weeks. However, it may take up to a month in some areas in the current practice. This will disturb their min-max. do you understand? The other thing is when there is an emergency, [SUPPLYING HUB] is supposed to deliver it once the facility submits the report. This is because, as we have said earlier, they have no capacity. The supplier is budgeting to deliver the products and there is no additional budget provided to the facilities for transport to collect the pharmaceuticals from the supplier. So, the big problem is the facility itself go, and collect it at the time of emergency. This happens frequently. The other is a security problem. The agreement at national and regional level is that [SUPPLYING HUB] delivers directly to accessible facilities, including for RDF drugs. [SUPPLYING HUB] delivers both programs and budget drugs to our accessible facilities. But, when we see the practical thing, the [SUPPLYING HUB] only reaches the woredas, or even zones. (long pause) they are not even reaching an accessible health facility due to fear of security. They leave it at zone, especially in [Names of six zones in a row] It is a big challenge. Here in the the [Name of a zone]. They put it at zone level and zone deliver it using ambulance after facilities experience stockout. Due to the the current country level problem associated with security,, [SUPPLYING HUB] only delivery to facilities within a radius of 50 KM from its hubs. So, zone communicate with woreda and woreda with facilities, they collect the products and use the existing transportation modality. So, this are challenges from the supplier side.

I: Can you tell us about route optimization?

R: Route optimization, …. We have also raised about it previously. For some facilities, they use the route that was designed a a long time ago. For instance, if there is hospital or health center around here and the facility not included in the map, the deliverer refuses to provide it to that facility and goes to other the woreda. Then that woreda will bring the products back to that facility. In my understanding it is better if the route optimization is revised and mapping is done again. This is the problem we are facing in [Name of tow zones]. They are reaching health facilities by passing the one which is not in the route. This should be changed to save transportation cost and deliver the products on time. To reach the facility directly and to save time, it is better if the route is revised. Other than other problems like security issues, almost all health facilities are accessible.

Now, due to this factor most of our facilities are being considered as inaccessible and the drugs are being delivered to woreda. So, it needs route optimization. Another issue, … what we have faced is, you know, [Name small town in west (region)]?

I: Yes

R: [Name of a zone] too?

I: yes

R: you see, X hospital and Y health post are found in the same town. One of them is on this side of the road and the other one is on the other side of the road toward Addis Ababa. The x hospital which is on the side closer to Addis Ababa is provided by this hub, while the other one is provided by the [Name of other hub]. This is a big loss as a country. So, route optimization should be revised to this extent.

I: What about the challenges delivering from health center to health posts?

R: I may say data quality is the main challenge. The storage capacity of the facilities can also be a challenge. (*people talking from the background*). Health post accessibility and their storage capacity is challenging. We have raised this earlier. There is no building or premises that is considered as pharmacy store at health post. So, there is a small shelf where they can store it after collecting it from the health center. Previously they had been taking products used for a month, In case their capacity of holding products is increased according to last mile delivery, they are not able to store more products. If the schedule of the last mile delivery is similar to that of IPLS, hospitals may face pharmacy store problems as it is not enough and not in a status to keep the standard storage condition. The other thing is, the data quality problem which needs to work in it. This is it if I understand your question.

I: it was mainly related with the transportation, especially challenges related with delivering at lower level. What alternative transports are there to delivery commodities. It from health center to health posts.

R: I might have raised this earlier. Since the [SUPPLYING HUB] or supplier has no enough vehicle, our facilities and woredas are forced to collect it, especially during the emergency time.

Still, this is a challenge that we can face during the last mile delivery. So, our option is at least we have ambulance at woreda and hospitals level and it can be used. But big problem is … eee, pharmaceutical supply transportation has no budget. Budget is allocated only for procurement. Maybe they can communicate with [SUPPLYING HUB] to cover the fuel and other costs to be covered by them. It can be solved in such a way as this kind of practice was previously in place for some areas. Last mile delivery can be effective by using vehicles from the hospital, woreda and health centers, while [SUPPLYING HUB] covers the fuel and other costs. Based on the previous practice, I don't think [SUPPLYING HUB] can reach hospital, health center and health post. It might work way I have mentioned now. If they work together using woreda vehicle or then they cover the cost of fuel and perdiem. (people are talking from the background). As the ambulances are also available. It is possible to delivery products to health posts by using motorbikes that are available at the health center level. (people laughing in the background). But there are health centers that have no motorbikes. It has been a while since they were provided with motorbikes. It can be challenging if it is not functional. Providing them with it may be a solution in case there is a support mechanism. It is better if motorbikes are provided to health centers for delivery pharmacy products to health posts. Other than this, if the capacity issue is related to LMIS implementation, it is possible to deliver it as we also have experience. For instance, if we take the West [names of two areas] areas where you might have also information, health post resupply activity are in good status. If the problems I have stated is addressed, (phone ringing from the background) I hope it is possible to deliver. The big problem is transportation and vehicle shortage. It needs to work continuously with woreda. Woreda and the supplier do not know each other. [SUPPLYING HUB] directly communicate with the facilities. They may discuss with the zone if they face problem. The is no communication between woreda and [SUPPLYING HUB] or supplier. But communication is back bone for supply chain. So, it is better if the supplier work with zone and woreda management body both for distribution and transportation. There is technical working group at zone level and its better if they discuss about it on this platform. I hope it is in good status at region level. As our director is member of the team, they discuss on it when there is distribution and transportation problem. Then they bring up solution and it will be communicated to the lower level. [SUPPLYING HUB] is not working with zone and woreda but facilities. The facility has no capacity, the woreda is not giving supporting on the issue, so there will be interruption. So, it needs working with woredas … to solve transportation and distribution

problem. Working on facility level infrastructure problem will also help in the implementation of last mile delivery.

I: What about deployment of third-party logistics?

R: We also has recommended such things especially for security affected woredas and security affect facilities. What we said was, outsourcing transportation service. We discussed on it at region level a year. [SUPPLYING HUB] has tried to implement it in some area but, they are not successful. The problem is, the relationship between our supplier and the transportation sector is not as such strong. They just deal with individual owner and the level of sharing the challenge was not clear. They just have a deal informal deal with individual owner and it was not successful. If they outsource the transportation service, especially in security affected area, it will support last mile delivery.

I: May be (participant name), what I want you to explain is on from woreda to health center and from health center to health post. Last week I was at (zone). As you have said, There are transportation problem, infrastructure problem and data quality. You have provided some recommendation about fuel support, motorbikes. What I saw in (zone) is there are place where it is not possible for motors too. What do you recommend to facilities where the woreda doesn't have vehicle and the [SUPPLYING HUB] cannot access the facility?

R: the trend we use so far is to use traditional transportation. We had been using traditional transportation where their problem of infrastructure and security issue. This is the last option we have. Our other recommendation was (Lough), the one which is used by many African countries. We use a drone for inaccessible facilities ya? It is used in Africa country like Kenya. Sometime it is better to think that way. Why don't we use to delivery for vital drugs and vaccines? I know it is costy, to save life, ee … it is good to plan such option as a strategy. What other option do we have? You may use manpower by covering transportation cost. Second using traditional transportation as we already have the experience, especially in [name of zone] First wee need to design how we are going to use that traditional transportation and who cover the cost. It needs developing a system. Previously it was informal. It was only used in hurry when there is problem just to delivery

the products. So, if resilient supply chain is need, we have to design it well. It should be decided on who should pay for the transportation whether it is manpower or traditional one. Drone can be used, but it is costly for us. But to save life it is possible. It is better if we think of modern transportation. we don't have it even as a country. I think Kenya use it to transport vaccine. This my recommendation for last mile delivery.

I: What are the main implementation challenges of the IPLS in health facility?

R: eee … IPLS is the system we have for supply chain. I hope it needs to be updated. As a system we have been using it for more than ten years. In some place (phone ringing form background) health facilities report monthly while it s every two months in other places. So, I think this has to be similar. Or we may update the IPLS and make it based on accessibility. By the way we are facing challenge to evaluate it. Drug max-min is two months in some place, while it four months in other places. For example, the store we need for the four month is different from that of two months. The inventory management mechanism will affect the storage, it will affect the distribution. It also affects the LMIS too. So, let us update it, the IPLS. Other than that, we have mentioned about the capacity building earlier. The training is given once they then work for five or four years. But the supply chain needs update. Even it needs to update how to request program drugs. It needs to provide training g immediately after the updated done on LMIS formats. The programs are updated, the products enter the country, bu the facility use the old formats, so you will not find the product in the facilities will product is available in the country. So, when the product is available, the supplier should communicate it and update the LMISs. When the products are updated, pharmacy professionals should get that update. How can he ask for the new product if he is updated about it? The system is there but when the is updated on program drugs for TB, Malaria, HIV the LMIS should be updated and the workforce should get training and supportive supervision.

I: Any challenge related to utilization of LMIS formats and emergency order frequency?

R: yes, I mentioned this earlier. Emergency orders have been implemented according IPLS. We have a design, we have a system for emergency orders, okay. However, there is a problem with its implementation. The facility is not filling the format and sending it on time whether it is on

[eLMIS] or hard copy. Although there are facilities that fill and send it timely, [SUPPLYING HUB] is not delivering it. Now, we can say that IPLS is totally not functional for the emergency supply chain. You have to order emergency based on the set platform, then the supplier will deliver based on that. The emergency resupply must be within 72 hours, but a week is normal now. So, the problem is facilities are not using the format of emergency request appropriately. Some facilities use telegram. They also face problem to use [eLMIS] for emergency orders. As an expert, I learned that it need investment to use [eLMIS] for an emergency orders. It requires training, monitoring and mentorship, especially by discussing with IT professionals. It needs more mentorship. On the [SUPPLYING HUB] side, they should delivery for the facilities that properly requested emergency order. Their biggest problem is responding to the emergency requests. They are responding after a week or two of occurrence of the emergency. It will be difficult to say we have a system in such a situation.

I: what about wastage rate?

R: is it wastage rate as a region?

I: yes.

R: to reduce wastage rate as a region, I can say we go a long way. What we did was, we developed a guideline on product transfer in the region three years ago. We promoted it during review meetings and now our facilities are using it. The other thing is APTS is being implemented and its main function is to reduce the wastage. More or less our hospitals have implemented APTS. Some health posts also implemented it. So there is nationally set wastage threshold, which is less than 2 %. For example, ours is 2. We are working to make it less than that. But the problem is related to some program drugs that were distributed using push mechanism, especially for COVID 19 which was distributed 3 or 4 years ago. This will increase the program commodities wastage rate. The cumulative percent region is around 2%, it is 2.1 or 2.2. But at the facility level (phone ringing in the background) we go up to 3 as there are products that are provided using push mechanisms. As a region we are working to reduce wastage by making them implement APTS, presence of stock transfer practices and not accepting support for products that has less than one month period. The stock transfer practice helped us a lot with this regard.

I: what challenges are there to implement [eLMIS] it terms of Infrastructure availability, functionality, data accuracy, support mechanisms, and data utilization?

R: the coverage has reached around xxx in the region. Out of [yyyy] health centers and [zzz]hospitals we have, we have [AAA] [eLMIS] sites. That means the computers were purchased and it was installed. For this, we have identified 139 of them functional during assessment. From this there are on-off facilities. This is what the status of [eLMIS] looks like. The problem is, the system is not friendly. We know our professionals are not good enough even in using excel as there are someone who directly joined the work immediately after graduating from school. The training was given to IT and pharmacy professional, then IT expert from center install it and provided orientation on how to use it. But, since the troubleshooting training was not given, they stopped using it when there was a problem. Close mentorship and providing remote support was needed, but this not as strong as there is shortage of manpower. Since there is no support, the service is interrupted. IT experts at zone and town level have received training but they are negligent and have no commitment. There is a problem of giving attention just like they give attention to DHIS 2. They gave more support to DHIS 2 while ignoring this one. Since the pharmacy professional does not know how to do the troubleshooting when there is a technical problem, it is getting interrupted. We have evaluated it and expect the IT professionals will provide support. To increase the coverage, there is shortage of computers. There are around 1500 health facilities including the hospitals and only 50% them have access to electricity and internet. So, 50 % is around 750 health facilities. We need to have more computers and printers to reach all these facilities. These are the challenges we have with the implementation of [eLMIS]. In collaboration with the Ministry, we plan to deploy and make the existing functional in 2017. We have discussed with people from DHA to communicate with IT professional at zone and woreda to support on stock on hand synchronization and RRF sink. I haven't heard it status at the national level. It is a big challenge by the way. To digitalize IPLS, besides the partners support, it needs government acceptance of its role in reducing wastage and increasing stock availability. Otherwise, it is difficult. (long pause) how long is it since [eLMIS] is implemented? It is not mature yet. I personally have no health facility that I can say it has implemented very well. This because government didn't take the ownership. Sadly, there is internet service in DHIS 2 room but, not available in pharmacy store which is not more than 10 meters away. You will face problem to install it in such areas as there

is no management ownership. For the presence of digitalization services there should be management ownership. We have discussed on this and hope it will be improved.

I: What are the challenges of inventory management systems in woredas, health facilities and health posts? Is the stock status analysis being carried out?

R: Eee, yes, it is done. Especially in hospitals and health centers which are doing APTS. They also receive interventions. When they do stock status analysis they are forced to receive important intervention. But it is not as satisfactory in terms of health facilities we have. Eee as I mentioned earlier, inventory management is four months maximum and two months minimum. Taking intervention based on the analysis of the stock is very challenging. We can say this practice exists in hospitals and health centers which implemented APTS. The first problem is know-how. They get the know-how of stock analysis during IPLS training. If they get training five years ago and they don't have know-how, and there is no system which supports it, doing stock status analysis will be forgotten. So, you need to develop and inform the formats used to do it, availing it, communicating what intervention the take after doing it. Nothing is done in that area. Training was given and the way they are using it should be closely monitored through mentorship.

I: How do you evaluate Understanding of HEWs on the stock out triggering points?

R: Previously, the relationship between health post and health center was very strong. Nowadays, their activity is not good in as a region and country due to different reasons. When the pharmacy professional or pharmacy store goes there to provide supportive supervision, there are many gaps. They will be resupplied every month. They are supposed to collect if there is shortage, by monitoring it using bincard. Orientation and supportive supervision have been given. The health posts have now been upgraded to a comprehensive level. The comprehensive health post has a pharmacy and laboratory and we hope it will be improved. It will trigger them if they are using bincard but, they are not using it. Now a days many communicable diseases are rising and this shows they are not working. But there are places where it is implemented well. Especially (zone) there are health posts that use the bin card properly, check their stock and report using HPMRR. There are about 7000 health posts in [region]. In this regard, we cannot say what is being done in (zone) represent all these health posts. LMIS utilization is poor at the health post level. So it needs capacity building, it needs supportive supervision, it needs availing LMIS formats, eee… it needs to update them, it needs to tell them what triggers the stockout.

I: What are the internal reporting and resupply system's challenges with the facility?

R: Okay, eee … internal reporting and resupply of the facility depends on the health facility commitment. It requires the involvement of many departments. If there is no management ownership, the departments may not stick to the schedule. The pharmacy store and management must enforce reporting on time. It is the role of CEO and PHCU director but there is negligence. They say to provide the drug if it is available. If the pharmacy store manager refuses to provide it for those who are not bringing the report, the PHCU director or the CEO force him to provide it. This is the big challenge they were raising. Eee when the management body is supportive, the pharmacy store manager should support and supervise them to fill the IFRR correctly. He has to inform them to come and collect the products based on their schedule. There is a problem with this regard. But there are areas which are using it well, where the drugs are not distributed without IFRR. Gaps are there in many places due to these reasons. Most of the time they visit the store when they are out of stock without a schedule. At this time, it will make the store busy and the management body enforce him to prevent interruption of the services. In such situations, we will not get actual consumption and it will affect the overall data quality of that facility. In this area, it needs to be discussed with directors and CEOs during the review meeting and supportive supervision. This is a big problem associated with [eLMIS]. We hear that requests are not based on the schedule. Our partners are not discussing it with the management body during their supportive supervision. They go to store manager. Zone, Woreda and facility management have no information about it. We have commented as the chain should not be in such a way. There should be management ownership to support [eLMIS], and we hope it will be corrected.

I: How is supply chain data generated, shared, and utilized for decision-making within health facilities?

R: eee (long pause) utilization of LMIS data for decision making is in infant stage even at region level. There are many gaps at the facility level in terms of generating, analyzing and using data for decision making. Most of the time they send the report and there is no practice of triangulating the data for decision making. They don't have the know-how. Since they don't have the know-how of triangulating the service delivery and supply chain data, they are not using it. As our data use culture is poor, they are not practicing it. With [implementing partner] initiative, we accepted its importance as a region, we are providing support availing LMIS formats, then checking the tallies

are done from top to lower level especially for program drugs and tracer drugs. This will help utilize the generated data used at the facility level. After utilizing it they will communicate with the stakeholders. At the region level there is pharmaceutical and medical supply performance monitoring team and, we have shared role and responsibility for each member. As a good opportunity, we have provided monitoring and evaluation training and it has also cascaded it up to woreda level. [inaudible 1:42:51] we have provided medical supply monitoring and evaluation training to all hospitals, woredas, towns and zones in 2016 E.C. We hope …. our region level pharmaceutical and medical supply performance monitoring team will evaluate the data for monitoring and evaluation of the data that was brought from the lower level in 2017. We hope there will be better data quality as it will monitor the data that come from lower level, give feedback. Previously the RRF were being filled, but its accuracy, completeness and timeliness was not checked. Other programs like TB, Malaria, and HIV are being reported after checking the data quality, but the pharmacy service was not like that. They fill and submit some format to get the drugs. There is big gab with regard to recognizing it as facility data and utilizing it.

I: what is the main reason? Is it related to behavior or, lack of motivation?

R: we can say knowledge gap, … competence, and lack of know-how. Secondly, as a health professional our data use culture is very poor. Data usage culture is not practical when it comes to supply chain data. Additionally, no one follows it. If there is no accountability and no one asks you why you are not using the data you generated, you will not be concerned. I think these are the reasons. Therefore, supply chain data is not used due to skill gaps and lack of accountability. No one asks you if you generate poor and false data. Supply chain data can cause over-stock or under-stock, and lead to service interruptions. This is our data utilization culture as a country.

I: Are performance monitoring teams at lower levels using supply chain data? What is the role of pharmacy in the team?

R: The performance monitoring team exists at the facility level. I mean starting from region to lower level. But the big problem that we recently learned is that, a performance team is not checking and, monitoring pharmacy indicators, both supply chain, pharmacy services and medical device indicators. So, at region level we have established supply chain and pharmacy service PMT

and we hope it will be established at lower level namely at zone, woreda, town and facility level this year. We are specifically working on it in [name of zone] as a in collaboration with [Name of zone}. We have started a pilot to help with the use of supply chain and pharmacy service data at facility woreda and zone level. There is a big gap but we hope we can move forward. PMT sees only other services in the facility and pharmacy indicators are not checked or reported. Despite it being the backbone of the facility, the pharmacy indicators are not reviewed in the hospitals. … the data quality is not checked and this is a big gap.

I: you have been saying a lot but can you give us recommendations on each area regarding the supply chain? Including workforce, inventory and overall supply chain improvement. Anything that can be used as best practice.

R: we were talking about the recommendation for each component. What I recommend for improvement is. The LMIS format should be consistent. I mean, it should be the same throughout the country. At the same time, follow up should be there to check if it has reached a lower level. There are different formats at different places. This will be a barrier to data utilization. If the hard copy is used, … for instance, HMIS has the owner—Ministry of Health print and distribute it. LMIS formats interrupts and the facilities will be forced to use old formats. This will be a barrier for data quality and utilization. The LMIS format should have the owner. Region can take ownership if the Ministry of Health cannot print or avail it. the region can print and distribute just like other formats. Mentorship is needed for supply chain and supply chain data use. From my understanding, supportive supervision hasn't shown change. If there is mentorship and the health professional working at that facility, coached quality data will be generated. There are many gaps in the area of capacity building. We have discussed ART, IPLS, monitoring and evaluation with DTC members during training. There is a high turnover and training gap nationally. So it is better to work more on training. Another issue is related to partners. it is better if any partner working in the supply chain area communicates with zones and woredas to bring ownership and sustainability. This is a big gap. We have support from (Names of implementing partners] To have sustainability, zone and woreda should be engaged and communicated. If they leave then area the it will be interrupted and you will never get supply chain data after that. Orientation should be given Pre refill analysis must be strengthened using the preset format. Only a few partners are working on

the quality of RRF data, but zones and woredas are unaware of that. About two zones have got the orientation. Strengthening supply chain capacity building and, availing consistent format and, mentorship. It is also better if training is given to the health center on the quantification. So far it is only provided to the hospitals. The tools should be friendly. Hospital tools are a little bit complicated. It can be found in the [eLMIS] Orientation or training can be organized for the health facility to help them use it. we hope quality supply chain data will be generated and used after that. Generally, as a nation we need to have similar digitalization. As I have said earlier, there is no ownership, it is on and off, so management at the facility level do not trust it. So, it is better if we have similar digitalization system that can be used by all just like other countries. Although we haven’t checked it, we heard about South Africa and Kenya. Their supply chain digitalization is better. I don’t know what is preventing us from being like them. If we look at the software in detail, it is not as strong. It needs a big support and investment. We need management support. In terms of workforce the is shortage and those on job are overburdened due to shortage.

I: Thank you very much. You gave us a lot of information. Thank you for your time and contribution.

End of interview
